# Ultraviolet light affects the color vocabulary: evidence from 834 languages

**DOI:** 10.3389/fpsyg.2023.1143283

**Published:** 2023-06-02

**Authors:** Dan Dediu

**Affiliations:** ^1^Department of Catalan Philology and General Linguistics, University of Barcelona, Barcelona, Spain; ^2^Universitat de Barcelona Institute of Complex Systems (UBICS), Barcelona, Spain; ^3^Catalan Institute for Research and Advanced Studies (ICREA), Barcelona, Spain

**Keywords:** color lexicon, ultraviolet light, lens brunescence, weak biases, linguistic diversity, statistics, phylogenetics

## Abstract

It has been suggested that people living in regions with a high incidence of ultraviolet light, particularly in the B band (UV-B), suffer a phototoxic effect during their lifetime. This effect, known as lens brunescence, negatively impacts the perception of visible light in the “blue” part of the spectrum, which, in turn, reduces the probability that the lexicon of languages spoken in such regions contains a word specifically denoting “blue.” This hypothesis has been recently tested using a database of 142 unique populations/languages using advanced statistical methods, finding strong support. Here, this database is extended to 834 unique populations/languages in many more language families (155 vs. 32) and with a much better geographical spread, ensuring a much better representativity of the present-day linguistic diversity. Applying similar statistical methods, supplemented with novel piecewise and latent variable Structural Equation Models and phylogenetic methods made possible by the much denser sampling of large language families, found strong support for the original hypothesis, namely that there is a negative linear effect of UV-B incidence on the probability that a language has a specific word for “blue.” Such extensions are essential steps in the scientific process and, in this particular case, help increase our confidence in the proposal that the environment (here, UV-B incidence) affects language (here, the color lexicon) through its individual-level physiological effects (lifetime exposure and lens brunescence) amplified by the repeated use and transmission of language across generations.

## 1. Introduction

The proposal that various aspects of language are influenced by non-linguistic factors has received increased attention during the last two decades (Dediu et al., 2017; Benítez-Burraco and Moran, 2018) and several such examples have been proposed, with differing degrees of support and acceptance. For example, it has been proposed that languages with small speaker populations where communication happens mostly in close-knit social networks of native speakers (“esoteric languages”) tend to be more complex than those of larger groups with a high proportion of non-native speakers (“exoteric languages”), a proposal with convincing theoretical, empirical, and modeling support (Wray and Grace, 2007; Lupyan and Dale, 2010, 2016). Other proposals concern the influence of the environment on speech sounds, including the negative effect of air dryness on linguistic tone (Everett et al., 2015, 2016) and on vowels (Everett, 2017), the influence of altitude on ejective consonants (Everett, 2013), or the link between vegetation type/density and phonological inventories (Maddieson and Coupé, 2015). Yet, other class of proposals concerns the influence of our own biology on language, such as the positive effect of a small or absent alveolar ridge prominence on the phonemic use of click consonants (Moisik and Dediu, 2017), the effect of bite on labiodentals (Blasi et al., 2019; Everett and Chen, 2021), or the influence of hard palate shape on vowel systems (Dediu et al., 2019) and on the articulation of the North American English “r” (Dediu and Moisik, 2019).

This article focuses on a particularly interesting proposal combining environment, biology, and language, namely that a particular frequency band of the incoming solar radiation (ultraviolet light, and more precisely, its band B, with wavelength between 280 and 315 nanometers) influences, across our lifetimes, the way we perceive colors (and, in particular, the “blue” part of the color spectrum) in such a way that the languages spoken in regions with high UV-B incidence tend to have a word denoting specifically the “blue” color much less often than the languages spoken under a low UV-B incidence. This hypothesis was proposed in its modern form originally by Lindsey and Brown (2002), and Josserand et al. (2021) tested it using a large database of 142 populations and advanced statistical methods which allowed the disentangling of the negative influence of UV-B from the effects of other potential predictors, finding strong evidence for a negative effect of high UV-B incidence on the presence of a specific word for “blue.” While convincing, Josserand et al. (2021) potentially suffered from the skewed nature of its database with low coverage of certain geographic regions and language families, raising the issue of its non-representativity for the present-day linguistic diversity.

Here, this database was greatly extended, not only in terms of the number of populations/languages (from 142 to 834) but also in the number of language families (from 32 to 155), as well as the languages within families and geographic macroareas; in particular, there is now a very good coverage of *Australia* and *Papunesia*, which were very under-represented in the original database. These data were then re-analyzed using the same methods as in the study by Josserand et al. (2021), and it was found that extending the database and increasing its representativity confirms the main finding of Josserand et al. (2021) and Lindsey and Brown (2002)'s hypothesis of a negative influence of UV-B incidence on the existence of a specific word for “blue.” Moreover, this relationship is linear provided the effect of subsistence strategy is also included, which accounts for the few hunter-gathering populations in high-latitude environments, and highlights the asymmetric nature of this effect: while high UV-B incidence, through its physiological effects on color perception (*lens brunescence*), generates a negative pressure against a specific word for “blue” that might “hide” the effects of other factors, low UV-B incidence is “neutral,” allowing the other factors involved in shaping the color lexicon (such as subsistence strategy) to act “freely.” Moreover, this new database contains several large families with enough languages that show variation in terms of the existence of a specific word for “blue” and of the UV-B incidence received to allow the application of phylogenetic methods designed to better capture the diachronic aspects of this influence: while the power is relatively small for individual families, there is convincing support for a negative diachronic relationship between UV-B incidence and “blue” especially when using two “global” language phylogenies.

Far from promoting a “single factor explanation” approach, this extension, just like the original, Josserand et al. (2021) makes clear that language is shaped by many factors in complex interplay, but that it is still possible, when using the right data and methods, to (partially) disentangle and study their individual effects.

## 2. Data

The data used here extends the one in Josserand et al. (2021), which is based on Josserand (2020), which, in turn, checked and expanded the data in Meeussen (2015), this last one checking and expanding the original dataset used in Brown and Lindsey (2004) (please see these respective publications for methodological details). Josserand et al. (2021) used data from 142 unique populations, each identified by the *Glottolog* (Hammarström et al., 2022) code (or *glottocode*; Hammarström and Forkel, 2021) of the main language it spoke, together with information about the presence (or not) of a *specific term for “blue”* in the vocabulary, its *geographical location*, its *elevation* from sea level, the *incidence of ultraviolet light* (or *UV* light), the *climate* (as the first three principal components of the 19 variables from *WorldClim*) and *humidity* (as yearly median and interquartile range estimated from the *NOAA* data), the *distances to the nearest lake, river and sea/ocean* (using data from *Mapzen*), the (*log* of the) *population size* (from Bentz et al., 2018), and the *subsistence strategy* (a dichotomous distinction between hunting and gathering, and food production, combining data from multiple sources: Turchin et al., 2015; Kirby et al., 2016; Bickel et al., 2017; Blasi et al., 2019) among other variables is not relevant here. These 142 languages belong to 32 language families and six macroareas (as per *Glottolog*), and Josserand et al. (2021) also estimated the putative geographical location of the proto-languages of these 32 families using various methods and heuristics (Wichmann et al., 2010; Hammarström et al., 2022), which allowed the estimation of elevation, UV light, climate, humidity, and distances to bodies of water for these as well (ofcourse, using present-day data). The UV incidence data came from the *NASA Total Ozone Mapping Spectrometer (TOMS)* for the year 1998 (see below for details), representing the amount of UV radiation that impacts the Earth surface (and the humans on it) at different wavelengths (measured in J/m^2^), of particular relevance here being the *UV-B band* (280–315 nm) considering the effects of the ozone layer, cloud cover, elevation, and the position of the sun.

The work reported here started from these data, and, because the limiting factor for testing the main hypothesis concerns the presence (or not) of a specific term for “blue” in a language's vocabulary (from now denoted as *blue*), the focus was first on collecting data that allows the estimation of *blue* for as many languages as possible. To this end, several new sources of information were used: on the one hand, Mathilde Josserand (see Section Acknowledgments) manually checked several dictionaries (especially for Australian languages) and she consulted experts in specific languages from the Laboratoire Dynamique du Langage (DDL), Université Lyon 2/CNRS, Lyon, France (see Supplementary Table 1), and, on the other hand, she collected data from the *Database of Cross-Linguistic Colexifications* (*CLICS*; Rzymski et al., 2020). For *CLICS*, she first selected all the languages having a concept for “BLUE” (https://clics.clld.org/parameters/837#1/21/1), then she selected all the languages for which the “BLUE” concept is *colexified* with the concept for “GREEN” or any other color. Based on this, she coded the variable *blue* as “yes” if and only if the concept “BLUE” is *not* colexified with the concept “GREEN”, and as “no” otherwise (please note that it was decided to not include the 20 languages, representing ≈0.25% of the *CLICS* data, for which “BLUE” was colexified with a color concept but not with “GREEN,” due to the uncertainties surrounding their interpretation here). A further source of data was represented by version 0.2 of *Lexibank* (List et al., 2022), from where the colexification of “BLUE” and “GREEN” was extracted and converted, when present, into the binary variable *blue* as described above for *CLICS*. The first three sources of data (i.e., Josserand et al., 2021, dictionaries, and *CLICS*) were concatenated, resulting in 830 datapoints, of which 83 (11.4%) are glottocodes that appear at least two times in one or more databases. For the glottocodes that appear more than once in this database, their information was aggregated by (a) picking just one entry in case of perfect duplication and (b) only for those duplicated entries with non-identical values for *blue*, by taking the means of the continuous variables [for example, for glottocode abui1241 (Abui, Timor-Alor-Pantar), CLICS has four entries with “no” for *blue* with longitudes 124.63, 124.62, 124.68, and 124.59, which were summarized in a single entry with longitude 124.63, representing their mean; please note that manual checking confirms that this is indeed meaningful]. For the remaining duplicates (i.e., the 70 glottocodes that appear with different values for *blue*), the following procedure was implemented: if the duplicates come from different databases (56 glottocodes), the entry given by Josserand et al. (2021) (if it exists) was retained preferentially, followed by the manual coding and expert opinion (if these exist), and, finally, by *CLICS* (this hierarchy reflects the subjective confidence in the reliability and validity of each database with regard to *blue*); however, there are 14 cases where the same entry appears more than once in *CLICS* (reflecting small-scale intra-linguistic variation), and it was decided to ignore these given their ambiguous interpretation. This resulted in an *aggregated database* with 728 unique datapoints (i.e., glottocodes), an apparent loss of 102 (12.3%) entries relative to the concatenated database. To this database, new datapoints from *Lexibank* were added corresponding only to glottocodes not already present in the database and which have the relevant “BLUE”/“GREEN” colexification information, representing 106 new unique datapoints. The following analyses and plots are based on this database (or subsets thereof, as appropriate to deal with missing data in specific variables) with 834 unique datapoints, comprising 503 datapoints from *CLICS*, 142 from Josserand et al. (2021), 106 from *Lexibank*, and 83 from other sources (see Supplementary Figure 1 for their distribution across the globe).

The other variables were collected and coded as in Josserand et al. (2021), with the exception of UV light incidence and population size. For the incidence of UV light, Josserand et al. (2021) used the data provided by the *NASA Total Ozone Mapping Spectrometer (TOMS;* which, unfortunately, is not available anymore at its original location, toms.gsfc.nasa.gov/ery_uv/new_uv/, but can still be found in the GitHub repository accompanying that paper at https://github.com/ddediu/colors-UV/tree/master/input_files/toms_nasa_uv), and, in particular, only the data form the year 1998 (so it could faithfully replicate the procedure in Brown and Lindsey, 2004). These data are measures of UV radiation (at several wavelengths, including the UV-B) as received by the human body taking into account the thickness of the ozone layer, the cloud cover, elevation, and the position of the sun, and is measured in J/m^2^.

This work uses the data from the *TOMS Nimbus-7 UV-B Erythemal Local Noon Irradiance Monthly* and the *TOMS Earth Probe UV-B Erythemal Local Noon Irradiance Monthly*, which show the local noon erythemal UV irradiance values (averaged per month), measured in mW/m^2^. These data are split into two datasets, the first covering the period 01/11/1978 (in the format dd/mm/yyyy) to 01/05/1993 (TOMS Science Team, Unrealeased; available from https://disc.gsfc.nasa.gov/datasets/TOMSN7L3mery_008/summary?keywords=erythemal uv as of October 2022), and the second from the period 01/08/1996 to 01/09/2003 (TOMS Science Team, 1996; https://disc.gsfc.nasa.gov/datasets/TOMSEPL3mery_008/summary?keywords=erythemal uv), covering thus a total of 22 years, with a break between 1993 and 1996. Then, the mean for all years and the standard deviation (computed over the monthly means) for each location were computed. It is important to note that these data are comparable with those used in Josserand et al. (2021) with two differences: first, the data in Josserand et al. (2021) concern, as explained above, only the year 1998, and second, the data here are measured in mW/m^2^, representing the *radiation intensity* or, equivalently, the energy per square meter received per second (vs. in J/m^2^, which is the energy received per square meter in a given time) and covers UV-B only (vs. four wavelengths, 305, 310, 320, and 380 nm, with UV-B covering the lowest two values). For completeness sake, the solar radiation (measured in kJ/m^2^ day) data from *Worldclim* were also extracted, representing the estimated average top-of-atmosphere incident solar radiation (calculated from latitude) per month for the period 1970–2000; its mean and standard deviation (across all months) for each location were computed. It is important to note one fundamental difference between the *TOMS* and *Worldclim* data, namely that while the first represents the actual UV-B incidence received by the human body out in the open taking into account various relevant factors (ozone layer, elevation, cloud cover, and sun's position), the second is an estimate of solar radiation at the top of the atmosphere obtained from the location's latitude (please note that, for consistency with the *TOMS* measures, we will also denote the *WorldClim* measures as referring to UV-B). Therefore, *a priori*, it is to be expected that the *TOMS* data are more relevant to the hypothesis tested here than the *Worldclim* data.

Concerning population size, Josserand et al. (2021) used the data from Bentz et al. (2018), in turn based on the last freely available version of the *Ethnologue* (Lewis et al., 2013). Here, these data were expanded by Mathilde Josserand and myself using two sources: given a *glottocode*, from its *Glottolog* entry, we accessed the corresponding *Multitree* (http://new.multitree.org/) metadata, where the number of speakers is provided, or the last freely accessible version of *Ethnologue* (18th edition; Lewis et al., 2015 website as provided through the WayBackMachine snapshot of 31/12/2015 at https://web.archive.org/web/20151231081912/). We always used the “total across all countries,” if available, with the exception of *Spanish, Portuguese, French*, and *English*, were she used the numbers only for Spain, Portugal, France, and the UK, respectively. The second source is represented by *Wikipedia* (https://en.wikipedia.org/wiki/Main_Page)/*Wikidata* (https://www.wikidata.org/wiki/Wikidata:Main_Page), also accessed from the *Glottolog*. If several numbers were given, we chose according to the following criteria: (a) the number that has the most references, (b) the number with the most recent source, or (c) if two numbers have the same number of references and equally recent, we chose the larger one. We used preferentially *Wikidata* over *Wikipedia*. These two sources of data were kept separate as two different population size variables. Ten languages (with glottocodes kurd1259, nepa1254, alba1267, basq1248, tzot1259, mari1278, erzy1239, rian1262, hadz1240, and saya1246) were detected for which the *Ethnologue* data contained errors, which were manually corrected using the 6th January 2013 snapshot of the 17th edition of the *Ethnologue* in the WayBackMachine.

For the statistical analyses performed (unless specified otherwise), the following continuous variables were transformed as follows: *latitude* → 1.0–*cos*(*latitude*) (so that this is 0.0 at the equator and 1.0 at the poles) and *longitude*→*cos*(*longitude*) (range between −1.0 and 1.0, corresponding to −180 and 180°, respectively); for *population size, elevation*, and *distances* to large bodies of water, *x*→*ln*(*x* + 1) (where *x* is the variable's raw value and *ln* is the natural logarithm in base *e* = 2.718282…; adding 1 avoids −∞ when *x* is 0); for *mean UV, sd UV*, and climate *PC1, PC2*, and *PC3, x* → [*x*- *mean*(*x*)]/*sd*(*x*) (i.e., the variable is *z*-scored to ensure a mean of 0 and a standard deviation of 1). The same transformations were applied to the corresponding variables at the inferred origins of the language families (if applicable).

Specifically for the phylogenetic analyses, a set of phylogenies that meet several criteria was collected: they belong to large language families for which there is enough data (the cutoff point used was of at least 10 languages with data for *blue*), for which there is enough variation in the values of *blue* and *UV-B* incidence between the leaves (the languages), and which have branch lengths (necessary for the type of phylogenetic techniques employed). With these, trees for 13 language families (Afro-Asiatic, Atlantic-Congo, Austroasiatic, Austronesian, Hmong-Mien, Indo-European, Nakh-Daghestanian, Pama-Nyungan, Sino-Tibetan, Tai-Kadai, Timor-Alor-Pantar, Turkic, and Uralic) and two “global” phylogenies (see Table 1 for details and sources) were collected. For all families, the *Glottolog* trees with three methods for imposing branch lengths (Round, 2022) were used: “original” (all branches have equal length), “exponential” (branch lengths are exponentially distributed: 1/2^*k*^ for the *kth* deepest branch), and “ultrametric” (rescaling the terminal branches so that all tips are equally distant from the root). Jäger (2018) used the ASJP database (version 17) to estimate a “global” language phylogeny (with branch length), which also provides subtrees for individual language families. Moreover, for several families, phylogenies derived from Bayesian phylogenetic methods applied to the vocabulary, either as summary (or Maximum Clade Credibility) trees or as a sample of individual posterior trees (100 or 1,000 such trees), were retrieved. Finally, Bouckaert et al. (2022) provides another “global” language phylogeny (with branch length) based on a completely different method, combining information from different sources (pre-existing language classifications, geographical location, external information for language splits, previous Bayesian analyses of several families, and genetic and archaeological data about human spreads) in a Bayesian framework.

**Table 1:** The language families for which phylogenies are available, showing the source of the phylogeny, the total number of trees with branch length provided, the number of leaves (languages) in the phylogenies, the percent of languages with a dedicated word for “blue” (i.e., a value of “yes” for the variable *blue*), and the Shannon entropy for *blue*.

**Family**	**Source**	**No. of trees**	**No. of lgs**	**% *blue***	***H*(*blue*)**
Afro-Asiatic	Glottolog (Round, 2021)	3	51	78.4	0.75
	Jäger (2018)	1	49	77.6	0.77
Atlantic-Congo	Glottolog (Round, 2021)	3	25	44.0	0.99
	Jäger (2018)	1	21	42.9	0.99
(Bantu)	Grollemund et al. (2015)	1+100	12	33.3	0.92
Austroasiatic	Glottolog (Round, 2021)	3	25	76.0	0.80
	Jäger (2018)	1	17	64.7	0.94
Austronesian	Glottolog (Round, 2021)	3	129	75.2	0.81
	Jäger (2018)	1	94	75.5	0.81
	Gray et al. (2009)	1+1,000	58	72.4	0.85
Hmong-Mien	Glottolog (Round, 2021)	3	23	43.5	0.99
	Jäger (2018)	1	11	54.6	0.99
Indo-European	Glottolog (Round, 2021)	3	80	85.0	0.61
	Jäger (2018)	1	64	90.6	0.45
	Chang et al. (2015)	1+1,000	34	97.1	0.19
Nakh-Daghestanian	Glottolog (Round, 2021)	3	31	93.6	0.35
	Jäger (2018)	1	28	92.9	0.37
Pama-Nyungan	Glottolog (Round, 2021)	3	47	19.2	0.70
	Jäger (2018)	1	29	27.6	0.85
	Bouckaert et al. (2018)	1+1,000	41	19.5	0.71
Sino-Tibetan	Glottolog (Round, 2021)	3	80	77.5	0.77
	Jäger (2018)	1	37	67.6	0.91
	Zhang et al. (2019)	1+1,000	19	73.7	0.83
Tai-Kadai	Glottolog (Round, 2021)	3	25	84.0	0.63
	Jäger (2018)	1	21	85.7	0.59
Timor-Alor-Pantar	Glottolog (Round, 2021)	3	21	38.1	0.96
	Jäger (2018)	1	16	25.0	0.81
Turkic	Glottolog (Round, 2021)	3	12	91.7	0.41
	Hruschka et al. (2015)	1+100	10	90.0	0.47
Uralic	Glottolog (Round, 2021)	3	26	96.2	0.24
	Jäger (2018)	1	23	100.0	0.00
	Honkola et al. (2013)	1+1,000	14	100.0	0.00
“Global” (1)	Jäger (2018)	1	641	66.3	0.92
“Global” (2)	Bouckaert et al. (2022)	1	703	66.0	0.92

Please note that the source “Glottolog” represents the Glottolog v4.6 trees (Hammarström et al., 2022) downloaded and preprocessed using the glottoTrees package (Round, 2021), with added branch length using the following methods (see Round, 2022 for details): “original” (all branch length are equal), “exponential” (branch lengths are exponentially distributed: 1/2^*k*^ for the *kth* deepest branch), and “ultrametric” (rescaling the terminal branches so that all tips are equally distant from the root); please note that these branch lengths do not affect the tree topology but just the lengths of the branches. Jäger (2018) provides a global phylogeny, but also individual phylogenies for each language family. Grollemund et al. (2015) provides one summary and 100 individual posterior trees for Bantu (a subgroup of Atlantic-Congo). Gray et al. (2009) provides one summary and 1,000 individual posterior trees for Austronesian. Chang et al. (2015) provides one summary and 1,000 individual posterior trees for Indo-European, but all languages have a word for “blue,” making these trees unusable for the phylogenetic methods. Bouckaert et al. (2018) provides one summary and 1,000 individual posterior trees for Pama-Nyungan. Zhang et al. (2019) provides one Maximum Clade Credibility (MCC) tree for Sino-Tibetan. Hruschka et al. (2015) provides one summary and 100 individual posterior trees for Turkic. Honkola et al. (2013) and Jäger (2018) provide one tree, and one summary and 1,000 individual posterior trees, respectively, for Uralic, but all languages have a word for “blue,” making these trees unusable for the phylogenetic methods. Both Jäger (2018) and Bouckaert et al. (2022) provide world-wide “global” phylogenies constructed using very different methods and data.

## 3. Methods

Most of the methods used here build incrementally on those used by Josserand et al. (2021), with the exception of the phylogenetic methods. First, there is the now “standard” *mixed-effects/hierarchical logistic regressions* approach, where one regresses the binary dependent variable *blue* (i.e., does the language have a specific word for “blue”?) on various (combinations of) predictors (such as the mean UV-B incidence), with controlling for “Galton's problem” and language contact by having language family and macroarea as random effects (Jaeger et al., 2011; Ladd et al., 2015; Josserand et al., 2021). These regressions were preferentially performed in a Bayesian framework (using brms in R; Bürkner, 2018), but also using a frequentist approach (using glmer; Bates et al., 2015) in some cases. In both frameworks, model comparison (which of two models should be considered “better”?), model simplification (starting from a “full” model containing a set of potential predictors, removing the predictors that do no contribute “significantly,” and retaining only those that do), and variable selection (does an individual predictor “significantly” help predicting the dependent variable?) were performed. In the frequentist framework, the *p*-values reported by glmer() for individual predictors (based on the Wald *Z*-test) and the *p*-values reported by anova() (based on the likelihood ratio test) and Δ*AIC* (difference in Akaike Information Crierion scores) for model comparison were used throughout (the α-level was 0.05 and the threshold for Δ*AIC* was 3). In the Bayesian framework, model comparison was based on BFs (Bayes factors), WAIC (the Widely Applicable Information Criterion or the Watanabe-Akaike Information Criterion), LOO (Leave-One-Out cross-validation), and KFOLD (*k*-fold cross-validation, with *k* = 10) as implemented by bayes_factor() in brms and by loo_compare() in loo (Vehtari et al., 2022). For BFs, the cutoff was $\frac{1}{3}$, while for the others, the cutoff was 4.0 points. Please note that there might be differences between BFs, on the one hand, and WAIC/LOO/KFOLD, on the other, due to the default use of improper priors (see, for example, here) and to intrinsic differences in what these indices capture (McElreath, 2020), such that the decisions here were based on a combination of these indices. For model simplification and variable selection, the posterior distribution of the predictor of interest *vis-à-vis* 0.0 (judged jointly from the posterior plot and the 95% Highest Density Interval) and formal hypothesis tests against 0 [either directional, when a direction is *a priori* hypothesized, or punctual; please note that this is the posterior probability that the variable is in the given relationship with 0 or the posterior probability that the variable is not 0, respectively as given by hypothesis() in brms], supplemented by model comparison (as described above), were used. To control for “Galton's problem,” the family as a random effect (most models) was included, but also a model where the “global” language phylogeny of Jäger (2018) and the associated phylogenetic variance-covariance matrix were as a grouping term (using brms's gr() syntax, and ape's vcv.phylo() function; Paradis and Schliep, 2019) was run. Likewise, to control for contact, macroarea as a random effect (most models) was included, but also a model where a 2D Gaussian process (one per macroarea, using brms's grouping gr() function by longitude, latitude, and macroarea), as suggested in McElreath (2020) and Naranjo and Becker (2022), was run. Moreover, one extra model was fitted, where both the “global” language phylogeny of Jäger (2018) and the 2D Gaussian process, as described above, were included.

Second, *mediation analyses* were conducted, which can quantify the direct and the indirect (or mediated) effects of a *treatment* (*T*) on an *outcome* (*O*) possibly mediated by a *mediator* (*M*). Thus, there is a *direct effect* (with strength *a*, represented as $T\overset{a}{\underset{}{\to }}O$) and an *indirect effect* “flowing” through *M* ($T\overset{b}{\underset{}{\to }}M\overset{c}{\underset{}{\to }}O$, with two components of strengths *b* and *c*, respectively), with the *total effect* (i.e., the overall influence of *T* on *O*, $T\overset{↗M↘}{\underset{}{\to }}O$) of strength *a* + *b* × *c*. These are estimated here by fitting the two mixed-effects regressions (with family and macroarea as random effects) to the data jointly (using R's notation):


$$\begin{array}{l} M\sim T+\left(1|family\right)+\left(1|macroarea\right) \end{array}$$

$$\begin{array}{l} O\sim T+M+\left(1|family\right)+\left(1|macroarea\right) \end{array}$$

These were fitted in a Bayesian framework (using brms), estimating, for each individual component (*T*→*O*, *T*→*M*, and *M*→*O*), its strength (*a*, *b*, and *c*, respectively), as well as their 95% HDIs, and their “significance” was judged based on the inclusion of 0 in the 95% HDI; for the effects (total, direct, and indirect), their strength (*a* + *b* × *c*, *a*, and *b* × *c*, respectively) was estimated, as well as their 95% HDIs, and their “significance” was judged based on the inclusion of 0 in the 95% HDI and the posterior probability of the hypothesis *p*(estimate = 0) (using hypothesis() in brms). These mediation models were also fitted using piecewise Structural Equation Models in a frequentist framework (using package piecewiseSEM in R; Lefcheck, 2016), which allows not only the estimation of the total, direct, and indirect effects (with bootstrapping 95% CIs and *p*-values) and of *a*, *b*, and *c* (with standard errors and *p*-values) but also to test the existence of the direct effect using d-separation (within Judea Pearl's causality framework; Lefcheck, 2016; Pearl and Mackenzie, 2018). Please note that only those mediation models that make sense theoretically and where the three components (*T*→*O*, *T*→*M*, and *M*→*O*) were individually “significant” (as regressions), or when they were of particular *a priori* theoretical importance were actually estimated.

Third, *path analysis* (Wright, 1934) models were fitted using lavaan (Rosseel, 2012), which model those relationships that are theoretically important (see Josserand et al., 2021 for details) to the primary hypothesis. While this method allows the simultaneous modeling of multiple influences (paths) between several variables (which the mediation approach does not), it cannot (at the moment) control for the effects of family and macroarea (as the mediation models do); moreover, this was fitted in a frequentist framework. To address some of these limitations, path analysis was also conducted in a piecewise Structural Equation Models framework where the individual regressions composing the model are fitted simultaneously either in a frequentist (using piecewiseSEM) or Bayesian (using brms) approach, which allow the inclusion of family and macroareas as random effects and the use of generalized linear models (in particular, of logistic regression; *N.B*., piecewiseSEM does currently have some limitations that might affect the use of dichotomous variables). However, it can be argued that some of the predictors are, in fact, indirect measurements of the unmeasured latent variables that presumably play the causal role, in particular *UV-B incidence* (captured by its mean and standard deviation), “*cultural complexity”* (partly captured by subsistence and population size), and possibly *climate* (captured by various climate PCs and humidity). Therefore, Structural Equation Models with latent variables were also implemented using lavaan with the aforementioned limitations, with the partial exception of also conducting a multi-group analysis using macroarea as a grouping factor, which allows the estimation of separate parameters for each macroarea.

Fourth, various techniques were employed to check which of the many potential predictors of *blue* do, in effect, predict it. For all these techniques, the full dataset was split randomly into a training (80% of datapoints) and testing (remaining 20%) datasets, 100 times (this allows the testing of how well the model generalizes to new “unseen” data). Then, *Bayesian multiple logistic regression* with manual model simplification (as implemented by brms), *conditional inference trees* (as implemented by ctree() in package partykit; Hothorn and Zeileis, 2015), *random forests* (as implemented by randomForest() in package randomForest; Liaw and Wiener, 2002), *conditional random forests* (as implemented by cforest() in package partykit), and Support Vector Machines [SVMs, as implemented by fit(…,model=~svm~) in the rminer package; Cortez, 2020] were fitted.

Finally, several *phylogenetic analyses* were performed, as follows. The *phylogenetic signal* of *blue* was estimated using three methods: the Fritz and Purvis (2010)' *D*, as implemented by phylo.d() in package caper (Orme et al., 2018), which provides a numeric estimate *D* of the phylogenetic signal and also two *p*-values associated with the hypotheses (*D* = 0 that the character is “clumped,” evolving on the phylogeny under a Brownian motion model and *D* = 1 that the character is random relative to the phylogeny, respectively). The remaining two methods are based on performing the logistic phylogenetic regression of *blue* with no predictors, as implemented by binaryPGLMM() in package ape [Paradis and Schliep, 2019; which gives the “phylogenetic signal measured as the scalar magnitude of the phylogenetic variance-covariance matrix s2 * V” (denoted here as *s2*) and the *p*-value of the “likelihood ratio test of the hypothesis H0 that *s2* = 0”], and by phyloglm() in package phylolm [Ho and Ane, 2014; using Ives and Garland, 2009's method, which uses “alpha to estimate the level of phylogenetic correlation” (denoted here as α); this might come with a warning if α is too close to its limits, in which case, this probably means that the phylogenetic signal is, in fact, negligible]. Then, *ancestral state reconstruction* for *blue* was performed (estimating the probability that a proto-language had a dedicated word for “blue”) using two methods: the one implemented by ace() in package ape and based on Pagel (1994) (both the single-rate, ER, equivalent in this case to the symmetric, SYM, model, and the all-rates, ARD, model were used; both estimate the appropriate transition rate(s), 1 for ER and 2 for ARD, and the probability of a “0,” i.e., the absence of “blue,” at the root; furthermore, the two methods were compared, using the Likelihood Ratio test and AIC, retaining the one best fitting the data), and the one implemented by rerootingMethod() in package phytools and based on Yang et al. (1995) (which estimates the marginal ancestral state estimates by re-rooting the tree; this works only for symmetric models, in this case ER, and gives the transition rate and the probability of a “0” at the root). Also, the *correlated evolution* of *blue* with all its potential predictors was estimated using two methods: one implemented by fitPagel() in package phytools based on Pagel (1994) (this only works for binary characters, so the continuous predictors were dichotomized using median split, i.e., all values <the median → “0,” all others → “1”), and the threshold model as implemented by threshBayes() also in package phytools and based on Felsenstein (2012) (this is a Bayesian method which works with both discrete and continuous characters). Finally, *phylogenetic regression* of *blue* on all its potential predictors of interest was performed, using three methods: the *Phylogenetic Generalized Linear Mixed Model for Binary Data* as implemented by binaryPGLMM() in package ape, the *Phylogenetic Generalized Linear Model* as implemented by phyloglm() in package phylolm (implementing the phylogenetic logistic regression of Ives and Garland, 2009 with both an optimized GEE approximation to the penalized likelihood of the logistic regression and the maximization of the penalized likelihood of the logistic regression methods), and the *Bayesian logistic regression controlling for phylogeny* as implemented in package brms, using gr(glottocode, cov = A,) where A is the phylogenetic variance-covariance matrix of the language family (in this case, the “flat” logistic regressions which completely disregard the phylogenetic information was also estimated, providing a baseline test of the relationship between *blue* and the considered predictor while ignoring Galton's problem).

## 4. Results

### 4.1. The languages

**Figure 1:**
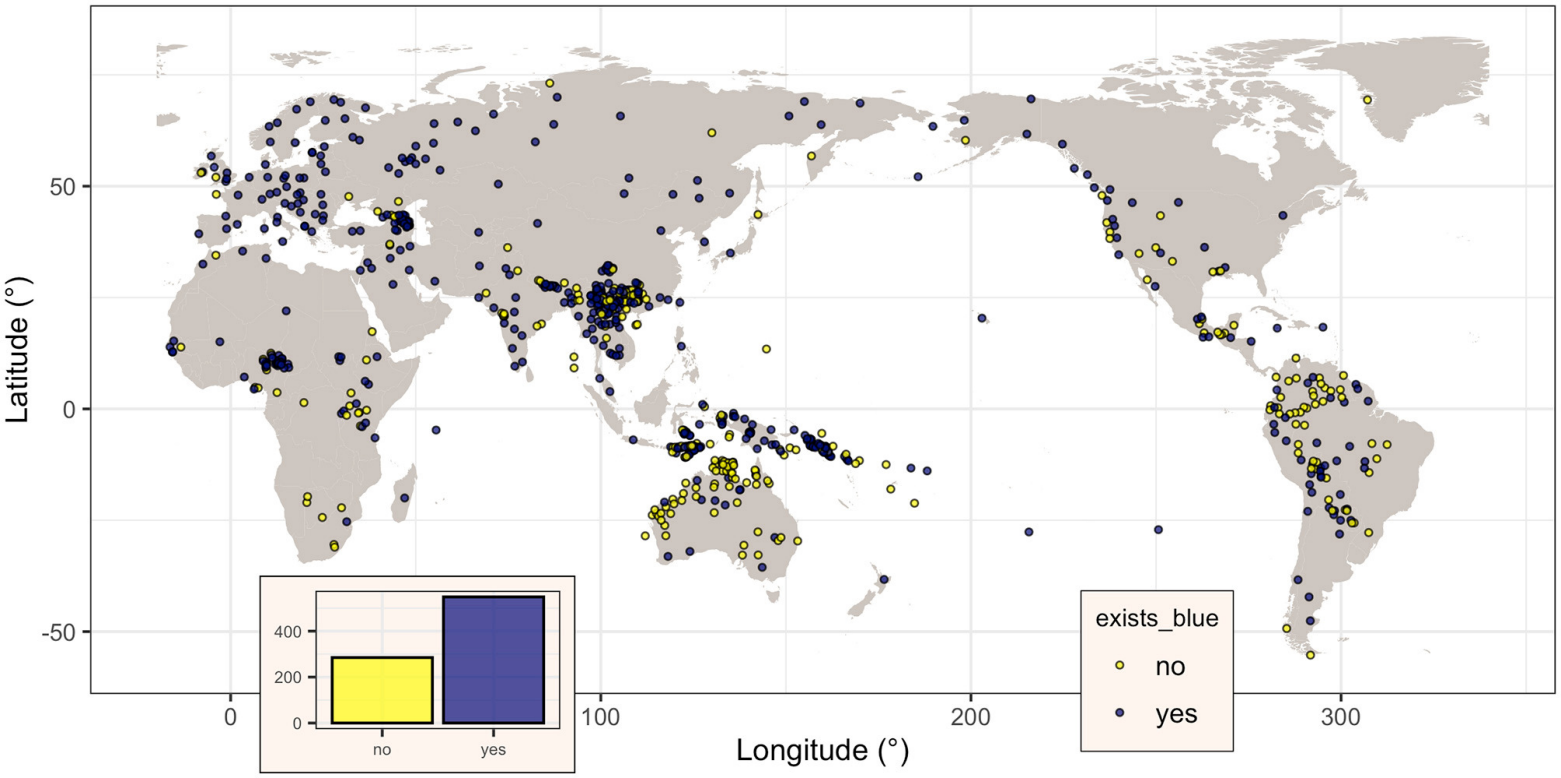
Map of the languages in the dataset, showing, for each, if there is a specific term for “blue” in the languages (dark magenta dots) or not (yellow dots). Figure generated using R version 4.2.3 (2023-03-15) and packages ggplot2 (version 3.4.1) and maps (version 3.4.1), using public domain data from the Natural Earth project as provided by the R package maps.

There are 834 unique glottocodes, distributed as shown in Figure 1. They belong to 155 unique *language families* (as per *Glottolog*, Hammarström et al., 2022), but the distribution is highly skewed, with most languages belonging to the *Austronesian* (glottocode *aust1307*; 134 languages), *Indo-European* (glottocode *indo1319*; 86 languages), *Sino-Tibetan* (*sino1245*; 85), *Afro-Asiatic* (*afro1255*; 51), and *Pama-Nyungan* (*pama1250*; 48), while 10 have only three languages, 8 have just two languages, and 110 only one, reflecting by and large the actual distribution of languages across families. Likewise, the distribution of the languages across the six *Glottolog* macorareas is uneven: ordered by decreasing number of languages, there are 350 languages in *Eurasia*, 187 in *Papunesia*, 88 in *South America*, 86 in *Africa*, 74 in *Australia*, and 49 in *North America*. Therefore, this extension of the database, from 142 unique datapoints (i.e., unique glottocodes) in 32 unique families to 834 unique datapoints in 155 unique families, resulted in a 5.87 times (or 487.3%) overall increase, both in terms of new language families added (123, of which most contain less than five languages but three are rather large: Nakh-Daghestanian, 32 languages, Timor-Alor-Pantar, 25, and Hmong-Mien, 25) as well as by adding new languages to existing families (mostly with just a few new languages, with the exception of Austronesian, 134 vs. 9; Sino-Tibetan, 8 vs. 9; Pama-Nyungan, 48 vs. 1; Indo-European, 86 vs. 41; Afro-Asiatic, 51 vs. 13; Tai-Kadai, 25 vs. 1; Austroasiatic, 25 vs. 3; and Uralic, 28 vs. 9). All macroareas have now many more languages, with the most dramatic increases for Australia (74 vs. 2 or an 3600.0% increase) and Papunesia (187 vs. 9, 1977.8%), followed by South America (88 vs. 12, 633.3%), North America (49 vs. 9, 444.4%), Eurasia (350 vs. 79, 343.0%), and Africa (86 vs. 31, 177.4%).

### 4.2. The variables considered

#### 4.2.1. Is there a dedicated word for “blue”?

The binary variable *blue*, coding the presence (“yes”) or not (“no”) of a dedicated word for “blue” in a given language, was coded for all the 834 languages, of which 549 (65.8%) do have such a word (i.e., *blue* is “yes”) and the remaining 285 (34.2%) do not. Visually (Figure 1), their distribution seems to be spatially non-random, with the majority of languages without a word for “blue” seemingly clustered closer to the equator. However, this impression can be misleading due to various confounding factors (Ladd et al., 2015), paramount being “Galton's problem” (Mace and Holden, 2005) and language contact. The first refers to the fact that related languages (i.e., languages from the same family) are not independent, as they may inherit some of their characteristics from the family's proto-language, while the second refers to the fact that languages in contact may come to share characteristics as well.

#### 4.2.2. UV-B incidence

**Figure 2:**
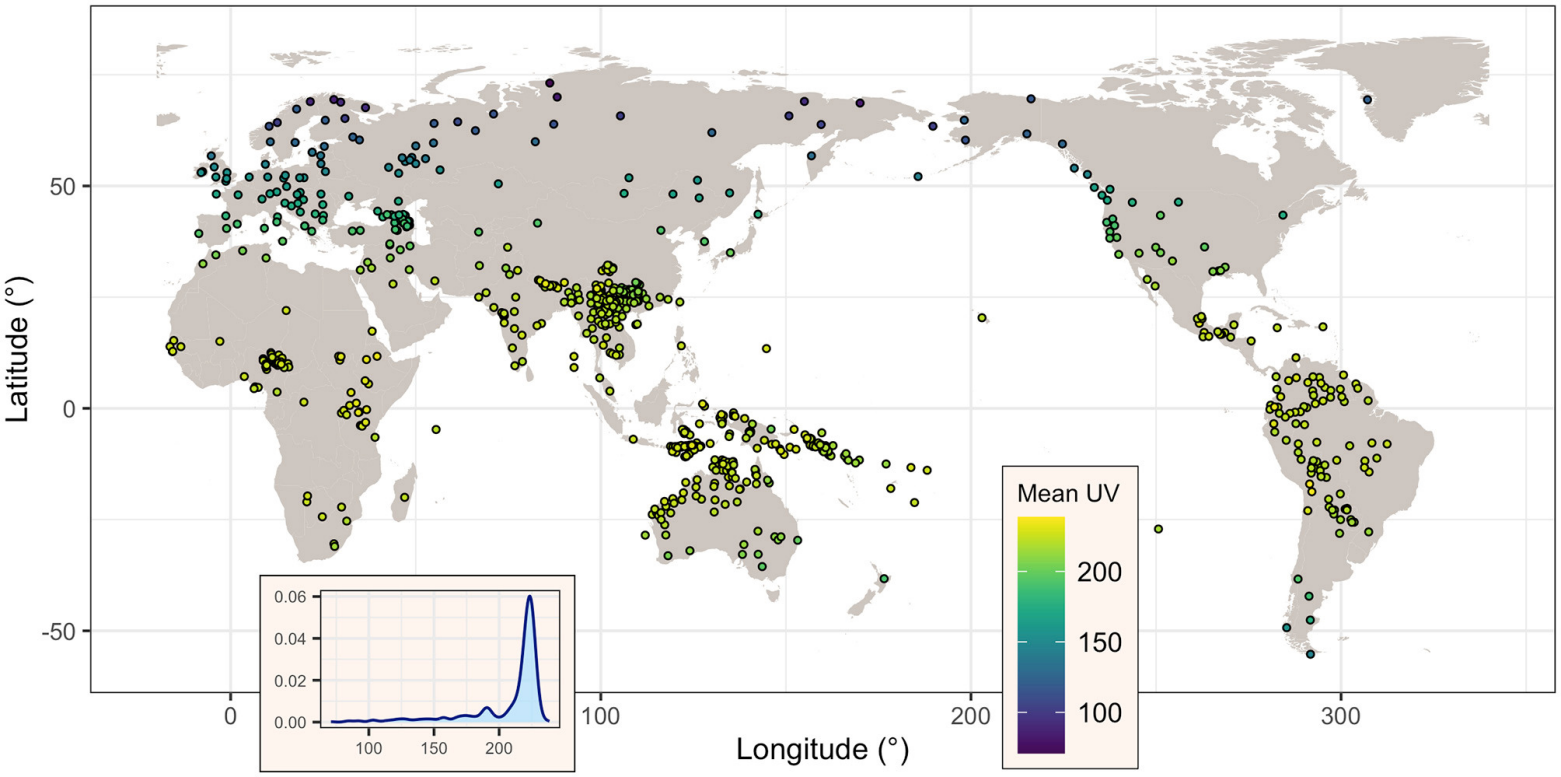
Map of the languages showing, for each, its mean UV-B incidence (as given by *TOMS*, in mW/m^2^), as well the overall distribution of this variable across all languages (inset). Figure generated using R version 4.2.3 (2023-03-15) and packages ggplot2 (version 3.4.1) and maps (version 3.4.1), using public domain data from the Natural Earth project as provided by the R package maps.

When using *TOMS* as a source of data, information was recovered for all 834 (100%) languages. The mean UV-B incidence (denoted here *UV*_*mT*_) varies between a minimum of 70.9 mW/m^2^ and a maximum of 238.6 mW/m^2^, with a mean of 208.5 mW/m^2^ and a median of 221 mW/m^2^, and a standard deviation of 29.0 mW/m^2^ and an inter-quartile range (IQR) of 16.1 mW/m^2^. As can be seen in Figure 2, *UV*_*mT*_ is sharply skewed toward high values, reflecting the relatively small number of languages at very high latitudes.

The standard deviation of the UV-B incidence (*UV*_*sT*_) varies between a minimum of 1.1 mW/m^2^ and a maximum of 71.2 mW/m^2^, with a mean of 16.4 mW/m^2^ and a median of 7.6 mW/m^2^, and a standard deviation of 17.5 mW/m^2^ and an IQR of 12.9 mW/m^2^. As can be seen in Supplementary Figure 2, *UV*_*sT*_ is sharply skewed toward low values, essentially because most languages in the dataset have a low seasonal variation in UV-B incidence.

When using *WorldClim* as a source of data, information was recovered for 829 (99.4%) languages. The mean UV-B incidence (*UV*_*mW*_) varies between a minimum of 6,780 kJ/m^2^day and a maximum of 22,681 kJ/m^2^day, with a mean of 16,499 kJ/m^2^day and a median of 17,045 kJ/m^2^day, and a standard deviation of 3470.2 kJ/m^2^day, and an IQR of 5,270 kJ/m^2^day. Its standard deviation (*UV*_*sW*_) varies between a minimum of 448.5 kJ/m^2^day and a maximum of 8,625 kJ/m^2^day, with a mean of 3,460 kJ/m^2^day and a median of 2,490 kJ/m^2^day, and a standard deviation of 2156.6 kJ/m^2^day and an IQR of 3981.4 kJ/m^2^day. Please see Supplementary Figures 3, 4.

There is a negative correlation between the mean and sd of the UV-B incidence (in *TOMS*: Pearson's *r* = −0.96, *p* = 0; Spearman's ρ = −0.75, *p* = 1.26·10^−149^; and in *WorldClim*: *r* = −0.55, *p* = 7.13·10^−67^, ρ = −0.43, *p* = 1.26·10^−38^) as expected due to the relationship between latitude and seasonality. As hinted by Pearson's *r* and Spearman's ρ and shown in Supplementary Figure 5, this relationship is clearer for *TOMS*, and, for both databases, it is non-linear.

As expected, the two databases are positively correlated with each other (for mean UV-B incidence: *r* = 0.78, *p* = 5.00·10^−170^, ρ = 0.64, *p* = 4.78·10^−96^; for sd UV-B incidence: *r* = 0.86, *p* = 1.64·10^−249^, ρ = 0.75, *p* = 2.49·10^−148^), but the relationship is far from perfect and is non-linear (see Supplementary Figure 6), suggesting that the two databases do not capture the same information about UV-B light incidence.

#### 4.2.3. Elevation, climate, and distance to large bodies of water

Elevation was available for all 834 (100%) languages and was heavily skewed toward low altitudes (see Supplementary Figure 7), ranging between −6 and 5,161 m, with a mean of 652.1 m, a median of 336 m, a standard deviation of 823.4 m, and an IQR of 746.8 m.

Climate data from WorldClim were available for all but three languages. The first Principal Component, PC_1_, of the climate variables, explains 53.9% of the variance and its high values reflect low seasonality, wet and hot climates (see Supplementary Figure 8). PC_2_ explains 23.3% of the variance (see Supplementary Figure 9), while PC_3_ explains only 7.1% of the variance (see Supplementary Figure 10), and they are harder to interpret.

For specific humidity (measured in grams of vapor per kilogram of air), data were available for all 834 (100%) languages. The mean of yearly medians (shortened as *median humidity* or *hum*_*m*_) ranges from 0.0014 to 0.02, with a mean of 0.012, median of 0.013, standard deviation of 0.005, and an IQR of 0.01 (see Supplementary Figure 11). The mean of yearly IQRs (shortened as *median variation* or *hum*_*v*_) ranges from 0.00035 to 0.012, with a mean of 0.0044, median of 0.0041, standard deviation of 0.003, and an IQR of 0.005 (see Supplementary Figure 12).

The distances to the nearest large bodies of water are measured in kilometers (km) as the crow flies, and are subdivided in the distance to the nearest lake (*dist2lake*, or *d2l*), to the nearest river (*dist2river* or *d2r*), to the nearest sea or ocean (*dist2ocean* or *d2o*), and the minimum between the three (i.e., the distance to the nearest large body of water irrespective of its type, denoted *dist2water* or *d2w*). These data were available for all 834 (100%) languages. The *dist2lake* is heavily skewed to the left with a few extreme outliers, and ranges between 0.5 and 2,770 km, with a mean of 40.4 km, a median of 21.4 km, a standard deviation of 115.0 km, and an IQR of 37.2 km (see Supplementary Figure 13). The *dist2river* is also heavily skewed to the left with a few extreme outliers, and ranges between 0.9 and 3,527 km, with a mean of 81.1 km, a median of 39.7 km, a standard deviation of 176.9 km, and an IQR of 68.5 km (see Supplementary Figure 14). The *dist2ocean* is less skewed and ranges between 0.6 and 2,194 km, with a mean of 317.7 km, a median of 146.5 km, a standard deviation of 354.8 km, and an IQR of 549.4 km (see Supplementary Figure 15). The *dist2water* is skewed to the left and ranges between 0.5 and 238.6 km, with a mean of 19.8 km, a median of 13.1 km, a standard deviation of 24.6 km, and an IQR of 17.3 km (see Supplementary Figure 16).

#### 4.2.4. Population size and subsistence strategy

For population size collected from both sources, data were missing only for 63 languages (covering thus 771 or 92.4% of the languages) in the *Ethnologue* and 78 (covering 756 or 90.6% of the languages) in the Wikipedia. The data primarily derived from the *Ethnologue* (measured in tens of thousands of speakers to reduce the order of magnitude of the numbers displayed) are heavily skewed toward small languages, and ranges between 0 (for 45 languages, including 41 reported as recently extinct) and 84,091, with a mean of 465.6, a median of 1.0, a standard deviation of 3,480, and an IQR of 29.1 (see Supplementary Figure 17). The data primarily derived from *Wikidata*/*Wikipedia* (also measured in tens of thousands of speakers), is also heavily skewed toward small languages, and ranges between 0 (for 46 languages, including 42 reported as recently extinct) and 92,000, with a mean of 663.6, a median of 1.0, a standard deviation of 4,257.1, and an IQR of 30.9 (see Supplementary Figure 18). There is a strong positive and 1:1 linear relationship between the two sources (Pearson's *r* = 0.98 and Spearman's ρ = 0.98, both with *p* < 2.2·10^−16^; see Supplementary Figure 19), with a few languages where the two estimates differ, in most cases due to the year of the estimate (very important for extremely endangered languages) or on the different type of categories of people considered (native speakers only, including L2 speakers as well, or even ethnicity).

Subsistence data were available only for 712 (85.4%) languages, and many more (553, 77.7%) practice subsistence modes centered around food production (“agriculture”) than those (159, 22.3%) whose subsistence mode is based on hunting, fishing, gathering, and/or foraging (“hunter-gatherers”). As expected, the latter tend to be found in marginal lands, being present mainly (in this dataset) in Australia, South America, and northern Eurasia (see Supplementary Figure 20).

#### 4.2.5. Language vs. origin-of-family measurements

For 15 variables, their value at the putative origin of the language families were also available. However, these values come with several caveats: first, the putative geographic origins are in most cases very controversial and come with probably very large errors; second, the value of the variables are present-day values, which might differ from their values at the time the proto-languages were spoken (ranging from hundreds to thousands of years, and usually now known with certitude). For each of these variables, the origin of family-level values versus the language-level values was plotted, their Pearson and Spearman correlations were computed, and their VIF (variance inflation factor) when used (as fixed effects) to predict *blue* in a mixed-effects logistic model with family and macroarea as random effects were estimated (see Table 2).

**Table 2:** The relationship between the language-level and the family-origin-level values for the 15 variables (1st column) for which the latter could be estimated.

**Variable**	**Pearson's *r***	**Spearman's ρ**	**VIF**
Longitude	*r* = 0.88, *p* = 6.7·10^−276^	ρ = 0.86, *p* = 3.9·10^−248^	1.7
Latitude	*r* = 0.86, *p* = 7.8·10^−246^	ρ = 0.78, *p* = 3.7·10^−170^	2.4
UV-B (mean; TOMS)	*r* = 0.83, *p* = 1.8·10^−214^	ρ = 0.67, *p* = 3.0·10^−111^	2.4
UV-B (sd; TOMS)	*r* = 0.87, *p* = 4.5·10^−258^	ρ = 0.71, *p* = 2.3·10^−129^	2.7
UV-B (mean; WorldClim)	*r* = 0.69, *p* = 1.1·10^−116^	ρ = 0.57, *p* = 4.1·10^−73^	1.5
UV-B (sd; WorldClim)	*r* = 0.78, *p* = 1.4·10^−172^	ρ = 0.68, *p* = 7.8·10^−115^	1.8
Climate PC1	*r* = 0.68, *p* = 1.2·10^−114^	ρ = 0.58, *p* = 2.4·10^−76^	1.5
Climate PC2	*r* = 0.56, *p* = 7.5·10^−70^	ρ = 0.55, *p* = 1.2·10^−66^	1.2
Climate PC3	*r* = 0.36, *p* = 1.7·10^−26^	ρ = 0.40, *p* = 5.5·10^−33^	1.1
Humidity (median)	*r* = 0.75, *p* = 1.7·10^−154^	ρ = 0.72, *p* = 3.9·10^−133^	1.7
Humidity (IQR)	*r* = 0.53, *p* = 5.0·10^−62^	ρ = 0.40, *p* = 1.3·10^−32^	1.2
Dist. to lakes	*r* = 0.13, *p* = 0.00017	ρ = 0.15, *p* = 8.1·10^−6^	1.0
Dist. to rivers	*r* = 0.03, *p* = 0.373	ρ = 0.02, *p* = 0.547	1.0
Dist. to oceans/seas	*r* = 0.65, *p* = 8.2·10^−101^	ρ = 0.61, *p* = 9.9·10^−86^	1.3
Dist. to water	*r* = 0.30, *p* = 2.9·10^−19^	ρ = 0.27, *p* = 5.9·10^−15^	1.1

Shown are: their Pearson's (2nd column) and Spearman's (3rd column) columns, as well as the variance inflation factor (VIF, 4th column) of a mixed-effects logistic regression of *blue* having the language-level and the family-origin-level values as fixed effects, and family and macroarea as random effects.

It can be seen, first, that the geographical locations of the present-day languages and of the putative origin of language families are very highly correlated, which is to be expected. However, there are a few families which show a very large spread among their daughter languages (Table 3), of particular interest here being those with a large spread in latitude, as latitude is the main driver of UV-B incidence as well as having a strong influence on climate.

**Table 3:** Languages families which have a standard deviation of latitude among their languages ≥ the median standard deviations across families of 2.3 (4th column), ordered decreasingly by this column.

**Family**	**Glottocode**	**sd (longitude)**	**sd (latitude)**	**No. of lgs**
Athabaskan-Eyak-Tlingit	atha1245	19.1	15.0	4
Indo-European	indo1319	58.4	15.0	86
Atlantic-Congo	atla1278	17.5	14.7	25
Tupian	tupi1275	5.1	11.7	7
Arawakan	araw1281	6.3	10.2	8
Afro-Asiatic	afro1255	12.2	9.6	51
Chukotko-Kamchatkan	chuk1271	9.3	8.4	2
Nuclear-Macro-Je	nucl1710	2.1	8.3	5
Turkic	turk1311	25.2	8.0	12
Tungusic	tung1282	11.0	7.7	5
Eskimo-Aleut	eski1264	45.9	6.5	6
Pama-Nyungan	pama1250	11.7	6.5	48
Austronesian	aust1307	24.7	6.4	134
Uralic	ural1272	21.4	6.1	28
Austroasiatic	aust1305	5.0	5.3	25
Uto-Aztecan	utoa1244	7.1	4.6	3
Tai-Kadai	taik1256	3.9	4.2	25
Dravidian	drav1251	2.6	3.8	5
Sino-Tibetan	sino1245	6.9	3.3	85
Nilotic	nilo1247	1.6	2.9	4
Mongolic-Khitan	mong1349	35.6	2.7	3
Pano-Tacanan	pano1259	3.0	2.4	8
Yukaghir	yuka1259	3.0	2.3	2

The standard deviation of longitude (3rd column), the number of languages with data in the family (5th column), the family name (1st column), and glottocode (2nd column).

Given these, it is no surprise that most variables show high correlations between the language-level and family-origin-level values (except for the distances to lakes and to rivers, the latter being the only non-significant one, given their high dependence on small-scale details of geography and climate), but it is also interesting to note that the highest VIF is ≈2.7, which is well below the usual cutoff of 5, and suggests that the family-origin-level values do not carry the same information as the language-level values.

### 4.3. Should the family and macroarea be modeled as random effects?

*A priori*, it is extremely important to control for Galton's problem, and for language contact (Ladd et al., 2015) so, it was also checked if, on these data, including language family and macroarea as random effects in a mixed-effects regression model is statistically justified or not. For this, the *null model*, *m*0 (i.e., in which *blue* is regressed only on the intercept, without any predictors), with both family and macroarea as random effects [in R's notation, *m*0 = *blue*~1+(1|*family*) + (1|*macroarea*)] and the null models that miss one of these random effects [*m*0_−*f*_ = *blue*~1+(1|*macroarea*) and *m*0_−*m*_ = *blue*~1+(1|*family*)] were compared (in both the frequentist and Bayesian frameworks). It was found that *m*0 has an Intraclass Coefficient Coefficient, *ICC* (which can be interpreted as the proportion of the variation explained by the grouping of observations as given by the random effects, ranging from 0%, when the random structure doe not explain anything, to 100%, when the random structure is enough by itself to explain the data), of 27.3%. Removing family significantly drops the fit (*m*0 vs *m*0_−*f*_: LR model comparison's *p* = 2.57·10^−6^, ΔAIC = 20.1, *BF* = 16059.0, ΔLOO = 14.5, ΔWAIC = 15.1, ΔKFOLD = 12.8), as does removing macroarea (*p* = 2.45·10^−5^, ΔAIC = 15.8, *BF* = 1108.7, ΔLOO = 4.8, ΔWAIC = 3.0, ΔKFOLD = 10.0). Thus, both random effects will be systematically included in the following models.

### 4.4. The potential predictors of *blue* considered individually

Both frequentist and Bayesian logistic mixed-effects regressions of *blue* on each of the following predictors of potential interest individually were performed: *UV-B incidence* (mean and sd, separately from TOMS and WorldClim), *latitude, subsistence* strategy, *elevation, climate* (PC1, PC2, and PC3), *humidity* (median and IQR), *distance to large bodies of water* (separately for distance to lakes, rivers, seas/oceans, and any type of large body of water), and *population size* (separately from the Ethnologue and Wikipedia/Wikidata). For the numeric predictors (all except *subsistence*), the process began with the quadratic model [*blue*~1+*x*+*x*^2^+(1|*family*) + (1|*macroarea*)], while for discrete predictors (only *subsistence*), it started with the linear model. Then, they were (automatically) simplified by dropping first the quadratic effect (if it exits), then the linear effect, and retaining the simplest model (which could well be the null model) that explains the data equally well as the most complex model. With this, it was found that the predictors which seem to have an individual effect on *blue* with various degrees of confidence are (see Table 4): UV-B as measured at the location of the languages (clearly negative for the mean, either quadratic [TOMS] or linear [WorldClim], and clearly positive for sd, probably linear [TOMS] and [WorldClim]), UV-B at the origins of the language families (suggestive linear, negative for the mean [TOMS], and positive for sd [TOMS] and [WorldClim]), latitude at the location of the languages (clearly linear positive), latitude at the origins of the language families (linear positive), climate PC1 at the origins of the language families (possibly negative linear), humidity median and at the origins of the language families (possibly negative linear), and distance to lakes (probably negative linear). The clearest signals are thus for UV-B incidence (negative for their mean and positive for their standard deviation) and latitude (at the language and family origins, positive). It is interesting to note that, in general, the frequentist and Bayesian estimates are in very good numeric agreement, but that the Bayesian approach tends to be more conservative.

**Table 4:** The predictors that individually seem to help predict *blue* in a mixed-effects logistic regression.

**Predictor**	**Approach**	**Formula**
UV-B (mean; TOMS)	Frequentist	−0.20 ± 0.08*x*^2^−0.93 ± 0.24*x*
	Bayesian	−0.52[−0.83, −0.21]*x*
UV-B (sd; TOMS)	Frequentist	0.57 ± 0.14*x*
	Bayesian	0.58[0.30, 0.89]*x*
UV-B (mean; WorldClim)	Frequentist	−0.36 ± 0.14*x*
	Bayesian	{−0.36[−0.64, −0.09]*x*}
UV-B (sd; WorldClim)	Frequentist	0.32 ± 0.13*x*^2^+0.07 ± 0.16*x*
	Bayesian	{0.28[0.02, 0.57]*x*}
UV-B (mean fam.; TOMS)	Frequentist	−0.31 ± 0.15*x*
	Bayesian	{−0.31[−0.63, 0.00]*x*}
UV-B (sd fam.; TOMS)	Frequentist	0.31 ± 0.14*x*
UV-B (sd fam.; WorldClim)	Frequentist	0.33 ± 0.15*x*
	Bayesian	{0.32[0.03, 0.65]*x*}
Latitude	Frequentist	3.07 ± 1.00*x*
	Bayesian	2.68[0.84, 4.77]*x*
Latitude (fam.)	Frequentist	2.93 ± 1.08*x*
	Bayesian	2.41[0.29, 4.52*x*]
Elevation	Frequentist	−0.06 ± 0.02*x*^2^+0.62 ± 0.23*x*
PC1 (fam.)	Frequentist	−0.37 ± 0.13*x*
	Bayesian	{−0.37[−0.64, −0.09]*x*}
Humidity (median)	Frequentist	−67.32 ± 25.12*x*
Humidity (median fam.)	Frequentist	−87.21 ± 31.69*x*
Dist. lakes	Frequentist	−0.20 ± 0.09*x*
	Bayesian	{−0.20[−0.38, −0.02]*x*}

It shows the predictor, the type of regression (frequentist, i.e., using glmer, or Bayesian, using brms), and the (essential) regression formula. For this last column, it uses the following conventions: for frequentist regressions, it gives the point estimate ± standard error, while for the Bayesian regressions, it gives the point estimate (i.e., the mean posterior) [95% HDI]. For both, it gives either the quadratic or the linear formula, as appropriate. If the Bayesian linear model is not formally better than the null (but marginally so) and the 95% HDI does not contain 0, it still gives the linear model but enclosed within { and }; please note that the global intercept α nor its variation by the random effect structure are shown, as these are not relevant for establishing the direction and relative strength of the predictor's effect. So, as an example, the first row is interpreted as a quadratic model (frequentist) with coefficient −0.20 ± 0.08 for the quadratic term and −0.93 ± 0.24 for the linear term, while the last row is a Bayesian linear regression that is not formally better than the null model, but where the slope, −0.20, is very probably negative, as 0∉[−0.38, −0.02].

Comparing the two UV-B incidence databases, TOMS and WorldClim, in terms of their capacity to predict *blue* when using UV-B mean in a mixed-effects logistic regression with family and macroarea as random effects, we suggests that TOMS is a better predictor [glmer: Δ*AIC* = 6.4, Δ*BIC* = 6.4; brms: *BF* = 23.0, Δ*LOO* = 2.4(2.6), Δ*WAIC* = 2.3(2.6), Δ*KFOLD* = 3.5(3.4)]. Likewise, fitting a mixed-effects logistic regression of *blue* as above, but with UV-B mean and sd from both databases as fixed effects simultaneously found high VIFs for UV-B mean (8.6) and sd (12.2) from TOMS, and low VIFs for the mean (2.2) and sd (2.9) from WorldClim, suggesting that the two databases contain highly overlapping information. Taken together with the substantive difference between the two databases in terms of what they actually mean in terms of UV-B incidence, it was decided to only use the TOMS data in the reminder of the article.

### 4.5. Mediation analyses

Several mediation models having *blue* as outcome were fitted (see Supplementary Figures 21–39), and it was found that, first, the significant positive total effect of *latitude* on *blue* (Bayesian: *TE* = 3.6[1.4, 5.9], piecewiseSEM: *TE* = 0.03[0.01, 0.05], *p* = 0.0001; please note that the effects are standardized but not the regression coefficients) is composed of a non-significant negative direct effect (Bayesian: *DE* = −5.7[−12.5, 1.1]), piecewiseSEM: no estimate) and a significant positive indirect effect (Bayesian: *IE* = 9.3[2.4, 16.1], piecewiseSEM: *IE* = 0.03[0.01, 0.05], *p* = 0.0001), the latter mediated through *UV-B mean* and composed of a significant negative effect of *latitude* on *UV-B mean* (Bayesian: β_*T*→*M*_ = −7.1[−7.2, −6.9], piecewiseSEM: β_*T*→*M*_ = −7.1 ± 0.1, *p* = 0) and a significant negative effect of *UV-B mean* on *blue* (Bayesian: β_*M*→*O*_ = −1.3[−2.3, −0.4], piecewiseSEM: β_*M*→*O*_ = −0.5 ± 0.1, *p* = 0.0007; see Supplementary Figure 21). When using *UV-B sd* instead, the results are similar, but suggest that *UV-B mean* is a better mediator of the relationship: the significant positive total effect of *latitude* on *blue* (Bayesian: *TE* = 3.1[0.1, 5.2], piecewiseSEM: *TE* = 0.01[0.00, 0.02], *p* = 0.0006) is composed of a significant negative direct effect (Bayesian: *DE* = −8.9[−15.6, −2.4], piecewiseSEM: *DE* = −0.02[−0.03, −0.00], *p* = 0.009) and a significant positive indirect effect (Bayesian: *IE* = 12.0[5.8, 18.6], piecewiseSEM: *IE* = 0.03[0.01, 0.04], *p* = 0.0003), the latter mediated through *UV-B sd* and composed of a significant positive effect of *latitude* on *UV-B sd* (Bayesian: β_*T*→*M*_ = 6.6[6.4, 6.8], piecewiseSEM: β_*T*→*M*_ = 6.6 ± 0.1, *p* = 0) and a significant positive effect of *UV-B sd* on *blue* (Bayesian: β_*M*→*O*_ = 1.8[0.9, 2.8], piecewiseSEM: β_*M*→*O*_ = 1.5 ± 0.4, *p* = 0.0003, see Supplementary Figure 22). *Climate PC1, population size*, and *distance to lakes* do not mediate the relationship between *latitude* and *blue*, but *subsistence* might (piecewiseSEM: *IE* = −0.01[−0.01, −0.00], *p* = 0, β_*T*→*M*_ = −0.6 ± 0.1, *p* = 0, β_*M*→*O*_ = 1.1 ± 0.3, *p* = 0, Supplementary Figures 23–25, 34). *Subsistence* seems to mediate some of the relationship between *UV-B* (*mean* and *sd*) and *blue* (Supplementary Figures 28, 29), but *population size* does not (Supplementary Figures 30, 31). Focusing on *distance to lakes* suggests that its negative effect on *blue* is, in fact, mediated by *latitude* and *UV-B* incidence (Supplementary Figures 33–39).

### 4.6. Path analysis and structural equation models

#### 4.6.1. Path analysis

I fitted various path analyses models that reflect to various degrees our causal beliefs connecting *blue, UV-B, latitude*, and other predictors using three different techniques, each with its own advantages and disadvantages: “classical” variance-based SEM (as implemented by lavaan; Rosseel, 2012), frequentist piecewise SEM (piecewiseSEM; Lefcheck, 2016), and Bayesian piecewise SEM (using brms; Bürkner, 2018).

**Figure 3:**
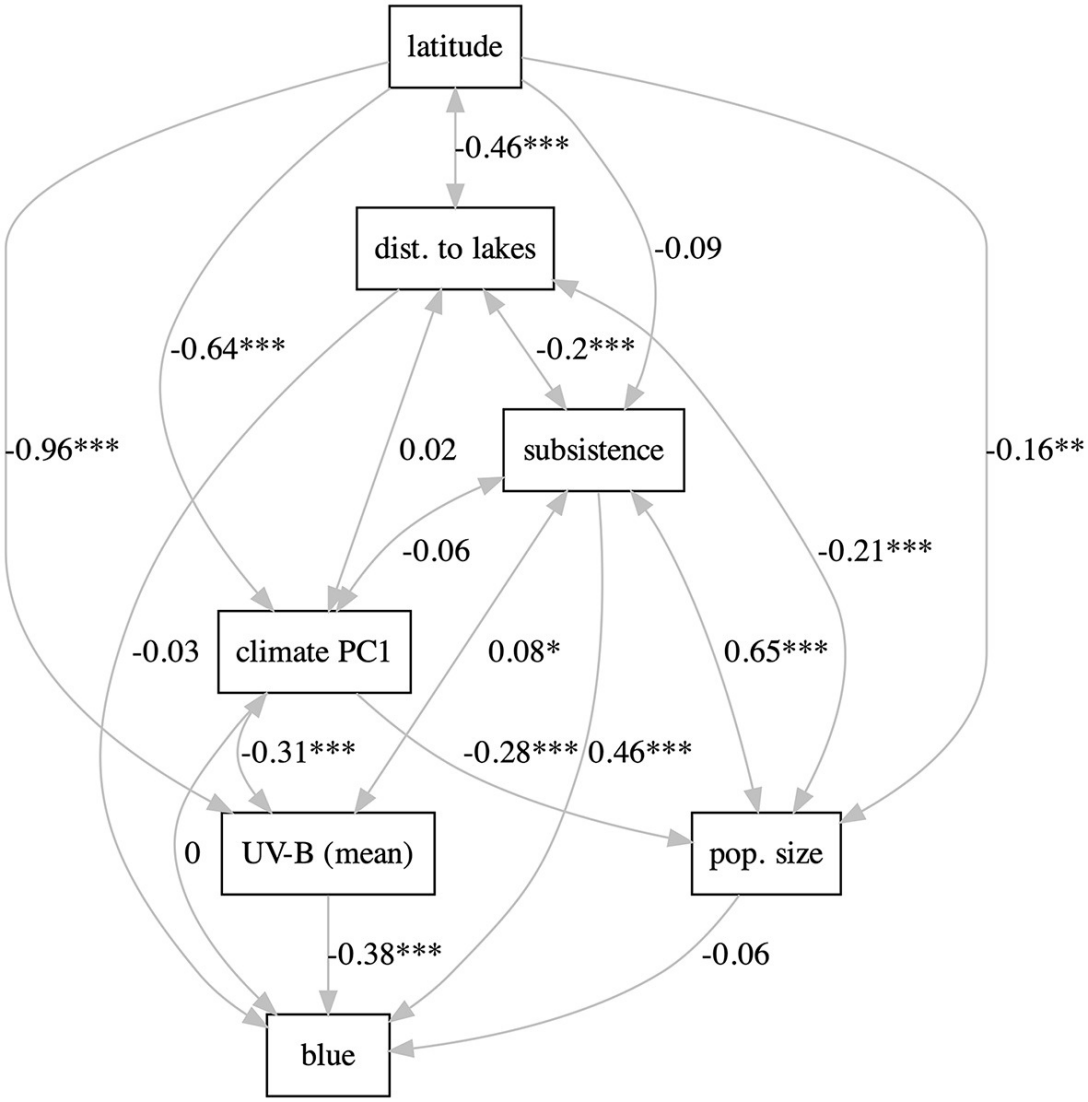
The “relaxed” path model using *UV-B mean*. The labels on the path are the path coefficients; stars represent significance (* ≤ 0.05, ** ≤ 0.01, and *** ≤ 0.001). Figure generated using R version 4.2.3 (2023-03-15) and package lavaanPlot (version 0.6.2). For all the path models, see Supplementary Figures 40–48.

With lavaan, two types of path models were fitted, as in Josserand et al. (2021). The “full” models include all the potentially relevant variables (*latitude, UV-B incidence, distance to lakes, climate PC1, subsistence, population size*, and *blue*) and most paths are directional (except for *UV-B* ↔*climate PC1*, and *UV-B* ↔ *distance to lakes*, which are modeled as correlations). Three such models were fitted: one including *UV-B mean* (Supplementary Figure 40), one including *UV-B sd* (Supplementary Figure 41), and one including both *UV-B mean* and *UV-B sd* (modeled as correlated; Supplementary Figure 42). All these models fit the data rather well and are equivalent in terms of fitting [for all: $\chi_{\left(1\right)}^{2}=0.1$, *p* = 0.74>0.05, *CFI* = 1.0, *TLI* = 1.02, *NNFI* = 1.02, and *RMSEA* = 0.00], suggesting that using the mean or the sd of UV-B incidence are equivalent from this pint of view. Using only the mean results in a negative but non-significant path *UV-B*→*blue*, using only the sd results in a significant positive path, and when including both, only the positive path from sd remains significant; the positive path *subsistence*→*blue* is significant in all models. The “relaxed” models kept the direction of the effect only in those cases for which there are strong *a priori* reasons (Figure 3 and Supplementary Figures 43, 44). This results in very good fits to the data and now the three models have slightly different fits as well [mean: $\chi_{\left(3\right)}^{2}=0.3$, *p* = 0.96>0.05, *CFI* = 1.00, *TLI* = 1.01, *NNFI* = 1.01, *RMSEA* = 0.00; sd: $\chi_{\left(3\right)}^{2}=0.1$, *p* = 0.99>0.05, *CFI* = 1.00, *TLI* = 1.01, *NNFI* = 1.01, *RMSEA* = 0.00; both: $\chi_{\left(5\right)}^{2}=0.3$, *p* = 0.99>0.05, *CFI* = 1.00, *TLI* = 1.01, *NNFI* = 1.01, *RMSEA* = 0.00]. As above, including individually the mean and sd of *UV-B* incidence results in significant paths to *blue* of similar strengths (negative and positive, respectively), but including both makes their paths to *blue* non-significant. Likewise, *subsistence*→*blue* is significant and positive in all models.

In contrast with lavaan, piecewiseSEM allows the inclusion of family and macroarea as random effects, and I fitted two path models corresponding to the “full” models above separately for mean and sd *UV-B incidence* (see Supplementary Figures 45, 46). Including the mean results in a much smaller AIC than not including it (Δ*AIC* = −263.9), the model fits the data very well [$\chi_{\left(7\right)}^{2}=141.0$, *p* = 0, and Fisher's *C*(14) = 167.5, *p* = 0], but the negative effect of mean(UV-B) is not significant (standardized β = −0.40, *p* = 0.065). Likewise, the standard deviation results in a Δ*AIC* = −221.5, the model fits the data very well [$\chi_{\left(7\right)}^{2}=45.0$, *p* = 0, and Fisher's *C*(14) = 69.0, *p* = 0], and the positive effect of sd(UV-B) is highly significant (standardized β = 0.73, *p* = 0.0003). In both models, subsistence (AGR) has a significant positive effect on *blue*.

While more flexible than lavaan, piecewiseSEM still has certain restrictions that may affect the results, prompting me to also implement piecewise SEM using brms to fit the two models described above (see Supplementary Figures 47, 48). The model including the mean is overwhelmingly better than the one without it [*BF* = 1.2·^42^, Δ*LOO* = 121.2(24.0), Δ*WAIC* = 123.7(23.6), and Δ*KFOLD* = 120.0(25.6)] and finds a clear negative effect of mean(UV-B) on *blue* [β = −1.10[−1.99, −0.23], posterior *p*(β < 0) = 0.98]. Likewise, the model including the standard deviation is overwhelmingly better than the one without it [*BF* = 2.5·^16^, Δ*LOO* = 70.9(37.9), Δ*WAIC* = 71.7(37.8), and Δ*KFOLD* = 66.3(40.2)] and finds a clear positive effect of sd(UV-B) on *blue* (β = 1.93[1.09, 2.79], posterior *p*(β > 0) = 1.00). Both models find a significant positive effect of *subsistence* (AGR) on *blue* and there may be hints of a negative effect of *distance to lakes* and a positive effect of *population size*.

#### 4.6.2. Modeling latent variables

However, it is arguably incorrect to include simultaneously both the mean and standard deviation of *UV-B incidence* as they are highly correlated and causally linked, being two connected aspects of the same unmeasured construct capturing the *UV-B incidence* received by a geographic location in a year. Likewise, *subsistence* and *population size* are, arguably, proxies for an unmeasured “*cultural complexity”* that might affect *blue*. Therefore, I also implemented a series of Structural Equation Models that explicitly model the latent variables *UV-B incidence*, measured by mean(UV-B) and sd(UV-B), and *cultural complexity*, measured by *subsistence* and *population size* (climate is captured by *climPC1*). However, currently only lavaan allows latent constructs and, given the complexities of fitting such models, I start with the main hypothesis and I subsequently added other factors to the model. First, the model implementing the main hypothesis (see Supplementary Figure 49) that *blue* is influenced by the latent *UV-B incidence* which is affected by *latitude* fits the data [$\chi_{\left(1\right)}^{2}=1.9$, *p* = 0.17 > 0.05, *CFI* = 1.00, *TLI* = 0.99, *NNFI* = 0.99, *RMSEA* = 0.032] and finds a significant negative effect of *UV-B incidence* on *blue* (standardized β = −0.83, *p* = 0.008); this latent loads approximately equally but with opposed signs on the mean (loading fixed to 1.0) and sd (loading −1.003, *p* = 0). Adding the climate (*climPC1*) improves the fit [$\chi_{\left(3\right)}^{2}=1.2$, *p* = 0.75 > 0.05, *CFI* = 1.00, *TLI* = 1.00, *NNFI* = 1.00, *RMSEA* = 0.00] and does not alter the relationship among *blue, UV-B incidence*, and *latitude*. Further adding the latent *cultural complexity* (see Supplementary Figure 50) makes the model to not formally fit the data anymore [$\chi_{\left(9\right)}^{2}=19.1$, *p* = 0.025 ≤ 0.05] but the fit indices are still very good (*CFI* = 0.99, *TLI* = 0.99, *NNFI* = 0.99, *RMSEA* = 0.04); the relationship among *blue, UV-B incidence*, and *latitude* remains the same (with a slightly weaker β_*UVB*→*blue*_ = −0.64, *p* = 0.002), and there is now a significant positive relationship between *cultural complexity* (mainly loading positively on *subsistence* but also on *population size*) and *blue* (β_*culture*→*blue*_ = 0.46, *p* = 0.0). However, adding the *distance to lakes* makes the model not fit the data and degrades its fit indices as well [$\chi_{\left(14\right)}^{2}=144.0$, *p* = 0 ≤ 0.05, *CFI* = 0.94, *TLI* = 0.88, *NNFI* = 0.88, *RMSEA* = 0.12] suggesting that we should not put too much weight on it, but it introduces a significant negative effect of this variable on *blue* and does not alter the previous relationships of interest.

Finally, while lavaan does not currently handle random effects, I attempted to control for the effect of *macroarea* by modeling it as a grouping factor in the first model that embodies the main hypothesis, estimating the models' parameters for each macroarea (*N.B*., this is fundamentally different from a random effects approach and cannot be applied to the language family due to the large number of families and generally very low number of languages per family). This fits the data well enough [$\chi_{\left(6\right)}^{2}=11.9$, *p* = 0.065 > 0.05, *CFI* = 0.97, *TLI* = 0.90, *NNFI* = 0.90, *RMSEA* = 0.08] and finds the following estimates of β_*UVB*→*blue*_ (with 95% CIs and *p*-values) per macroareas: Africa (−2.15[−4.24, −0.06], *p* = 0.044), Eurasia (−3.24[−5.82, −0.65], *p* = 0.014), Australia (1.55[−2.04, 5.13], *p* = 0.40), Papunesia (−3.39[−6.51, −0.28], *p* = 0.033), North America (0.40[−2.54, 3.35], *p* = 0.79), and South America (−1.74[−3.99, 0.51], *p* = 0.13).

### 4.7. Predicting *blue*

#### 4.7.1. Bayesian mixed effects regression

A Bayesian mixed effects logistic regression with family and macroarea as random effects, using all potential predictors as fixed effects fits well the full dataset (76.6% accuracy, 77.5% sensitivity, 74.1% specificity, 88.7% precision, and 77.5% recall). When randomly splitting the dataset into 80% training/20% testing subsets 100 times, using all the potential predictors, a good fit on the testing subsets was obtained (70.1 ± 2.9% accuracy, 74.7 ± 3.2% sensitivity, 59.4 ± 6.6% specificity, 81.7 ± 3.9% precision, and 74.7 ± 3.2% recall). Manual simplification retains the following three predictors [estimate, 95% HDI and *p*(ROPE)]: *UV-B sd* (β = 0.81[0.49, 1.11], *p*(*ROPE*) = 0.00), *subsistence* (β = 0.87[0.2, 1.52], *p*(*ROPE*) = 0.0003), and *distance to oceans* (family) (β = 0.24[0.02, 0.47], *p*(*ROPE*) = 0.29); this model still fits the full data well (76.0% accuracy, 76.7% sensitivity, 74.0% specificity, 89.2% precision, and 76.7% recall).

#### 4.7.2. Conditional inference trees

A conditional inference tree using all the potential predictors fits the full dataset well (72.9% accuracy, 72.0% sensitivity, 79.2% specificity, 96.2% precision, and 74.1% recall) and seems to make a distinction among the African, Eurasian, North American, and Papunesian languages, on the one hand, and the South American and Australian languages, on the other; for the former split, *UV-B (sd)* has a positive effect on *blue*, while for the second, the longitude of the family has a positive effect (see Supplementary Figure 51). When randomly splitting the dataset into 80% training/20% testing subsets 100 times, using all the potential predictors, good fits on the testing subsets were obtained (70.8 ± 3.5% accuracy, 74.3 ± 4.0% sensitivity, 62.2 ± 8.4% specificity, 85.0 ± 5.9% precision, and 74.3 ± 4.0% recall).

#### 4.7.3. (Conditional) random forests

Both random forests and conditional random forests fit the dataset well (72.3 ± 0.5% accuracy, 75.5 ± 0.4% sensitivity, 64.9 ± 0.8% specificity, 83.3 ± 0.5% precision, and 75.5 ± 0.4% recall; and 81.4 ± 0.3% accuracy, 81.2 ± 0.3% sensitivity, 81.9 ± 0.6% specificity, 93.3 ± 0.2% precision, and 81.2 ± 0.3% recall, respectively). Various measures of variable importance suggest the following top five predictors: *UV-B (mean), distance to oceans* (family), *UV-B (sd), latitude*, and *macroarea* (accuracy-based predictor importance from random forests); *UV-B (mean), UV-B (sd), latitude, population size*, and *elevation* (Gini-index-based predictor importance from random forests); and *macroarea, latitude* (family), *climate PC1* (family), *UV-B (mean)*, and *UV-B (sd)* (unconditional predictor importance from conditional random forests).

#### 4.7.4. Support vector machines (SVM)

An SVM using all potential predictors fits well the full dataset (74.7% accuracy, 74.3% sensitivity, 76.0% specificity, 91.7% precision, and 74.3% recall), and the top five predictors by importance are as follows: *distance to lakes* (family), *macroarea, elevation* (family), *subsistence*, and *climate PC1* (family). When randomly splitting the dataset into 80% training/20% testing subsets 100 times, using all the potential predictors, very good fits on the testing subsets were obtained (71.5 ± 2.9% accuracy, 71.8 ± 3.4% sensitivity, 70.7 ± 7.4% specificity, 89.9 ± 3.0% precision, and 71.8 ± 3.4% recall).

#### 4.7.5. Regressions controlling for phylogeny and contact

Furthermore, the Bayesian logistic regression of *blue* on the full set of potential predictors with manual simplification in brms were fitted, using (a) a 2D Gaussian process to model the spatial relationships between the languages (McElreath, 2020; Naranjo and Becker, 2022), which should better capture the continuous dependency of the probability and/or intensity of language contact on geographical space within macroareas (as opposed to the categorical use of macroareas as a random effect) while still including family as a random effect; (b) the “global” language phylogeny in Jäger (2018) to model the detailed “vertical” historical relationships between languages (as opposed to the categorical approach of using language family as a random effect) while still including macroarea as a random effect; and (c) combining both (a) and (b) in a single model where a 2D Gaussian process models the within-macroarea continuous language contact and the “global” phylogeny to model the detailed “vertical” historical relationships between languages. However, given that these models are very computationally expensive, they were not generalized to each individual predictor nor to the mediation models. Their findings clearly support the *a priori* hypothesis: after manual simplification, the model retained for (a) includes a negative effect of *UV-B mean* (β = −0.64[−0.98, −0.29], *p*(β = 0) = 0.03, *p*(β < 0) = 1.00), of *subsistence* (agriculture: β = 1.18[0.51, 1.86], *p*(β = 0) = 0.03, *p*(β > 0) = 1.00), and negative of *distance to lakes* (β = −0.20[−0.40, 0.02], *p*(β = 0) = 0.83, *p*(β < 0) = 0.97). The models retained in (b) and (c) both include only the negative effect of *UV-B mean* (β = −0.83[−1.90, 0.06], *p*(β = 0) = 0.36, *p*(β < 0) = 0.99, and β = −0.73[−1.39, −0.07], *p*(β = 0) = 0.30, *p*(β < 0) = 0.99, respectively).

### 4.8. Phylogenetic analyses

#### 4.8.1. Language families and trees with branch lengths

A total of 4,259 trees with branch length for 13 language families (Supplementary Figures 52–83) and two “global” trees (not shown due to their size) were collected (see Table 1 for summaries). The number of languages with data in a family varies between 10 (Turkic; Hruschka et al., 2015) and 129 (Austronesian; Round, 2021), with 641 and 703 languages in the two “global” trees (Jäger, 2018; Bouckaert et al., 2022, respectively). The percent of languages with a dedicated word for “blue” varies between ≈19% (Pama-Nyungan; Bouckaert et al., 2018; Round, 2021) and 100% (Uralic; Honkola et al., 2013; Jäger, 2018), with ≈66% for the two “global” trees. The corresponding Shannon entropy varies between an uninformative 0.00 (when “blue” is at 100%) to 0.99 (e.g., Atlantic-Congo; Jäger, 2018); for the two “global” trees, it is a very high 0.92.

#### 4.8.2. Phylogenetic signal and ancestral state reconstruction for *blue*

The phylogenetic signal of *blue* independently in each of the available trees was estimated and it was found, in summary, that there seems to be a significant phylogenetic signal at least in Austronesian, Indo-European, possibly Hmong-Mien, and the two “global” trees, but the results are rather patchy and seem to depend on the particular tree and method used (see Supplementary Table 2 for details). This probably reflects the need for large trees, as the signal for the two very large “global” trees is quite strong and consistence across methods.

Given this, it is not surprising that the ancestral state reconstruction of *blue* seems to depend on the particular tree and method used, but the following families seem to have had a specific word for “blue” in their proto-languages: Afro-Asiatic, Austroasiatic, Austronesian, Indo-European, Nakh-Daghestanian, Sino-Tibetan, Tai-Kadai, Turkic, and Uralic, while proto-Pama-Nyungan seems not to have had it. The “global” tree of Jäger (2018) seems to have had a specific word for “blue” at its root, but the other “global” tree (Bouckaert et al., 2022) is uninformative. See Supplementary Table 3 for details.

#### 4.8.3. Correlated evolution of *blue* with individual predictors

The correlated evolution between *blue* and each of its potential predictors was estimated separately. Focusing on UV-B incidence, there seems to be some evidence for correlated evolution between *blue* and *UV-B mean* and between *blue* and *UV-B sd* in a few families and trees (Austroasiatic and Hmong-Mien, and Austronesian, Hmong-Mien and Pama-Nyungan, respectively), as well as in both “global” trees (see Supplementary Tables 4, 5). For the other predictors, there is some evidence for correlated evolution with *blue* in some families as well as in one or both “global” trees, but is inconsistent—please see the full analysis report for details.

#### 4.8.4. Phylogenetic regression of *blue* on individual predictors

The phylogenetic logistic regression of *blue* on each of the potential predictors separately was performed using two non-Bayesian and one Bayesian approach, and for each, the corresponding non-phylogenetic logistic regression was also fitted as a baseline comparison which ignores “Galton's problem” (Mace and Holden, 2005). The results are presented in the Supplementary Figures 85–92 (see Supplementary Figure 84 for the full caption and interpretation key) and summarized in Supplementary Table 6. Several predictors show suggestive signals of coherent association with *blue* across multiple families and the two “global” phylogenies, including *UV-B mean* (negative), *UV-B sd* (positive), *longitude* (positive), *population size* (positive), *climate PC3* (negative), and *distance to lakes* (negative), while *climate PC1* varying between families and *subsistence* (agriculture) seems to have a positive effect blurred by being constant in so many families.

#### 4.8.5. The effect of *UV-B* incidence on *blue* in a phylogenetic context

Putting all these results together and focusing on the *a priori* main hypothesis of a negative effect of UV-B incidence on the existence of a dedicated word for “blue,” it was found that: first, there is significant correlated evolution for Austroasiatic and Hmong-Mien using the Bayesian approach, and using both methods. On the other hand, the logistic phylogenetic regression finds a significant negative effect only in a few cases (14 or 5.6% trees belonging to Indo-European, Uralic, and Sino-Tibetan and the two “global” phylogenies), but the estimated β's are negative in the majority of cases (≈65% when including the posterior trees, which give a very strong influence to the few families with such trees, and ≈75% when excluding them, which gives a much more balanced view); importantly, there is a significant strong negative effect for all methods in the two “global” phylogenies.

Second, there is also a signal of correlated evolution between *UV-B sd* and *blue*, and there is a significant positive effect for 11 (4.4%) cases and a positive β for ≈60 and ≈65% of cases, respectively; there is a strong positive signal in both “global” phylogenies.

Third, plotting the relationship between *blue* and UV-B incidence in each family separately (see Supplementary Figures 85–92 and the full analysis report) suggests that, first, only two families (Atlantic-Congo and Tai-Kadai) do not show any effect of UV-B incidence on *blue*, nine (sub)families show a signal consistent with the hypothesis of an effect of UV-B on *blue*, but three families show an effect in the opposite direction to that predicted(for *UV-B mean*: Uralic, for *UV-B sd*: Timor-Alor-Pantar, and for both: Turkic). However, it is clear that for Trukic and Uralic, this is driven by one outlier each in the north, while for Timor-Alor-Pantar, there is very little variation in UV-B incidence (The case of Indo-European is interesting as the MCMC summary and posterior trees seem to show an opposite effect to the expected one, while the Glottolog trees show an effect in the expected direction, with the Jäger (2018) tree showing essentially a null effect). Importantly, both “global” phylogenies show a clear and significant effect in the expected direction for both the mean and standard deviation of UV-B incidence.

### 4.9. The shape of the relationship between UV-B incidence and *blue*

Josserand et al. (2021), based on the original hypothesis by Brown and Lindsey (2004), only tested a linear negative effect of mean UV-B incidence on the probability of having a specific word for “blue.” However, the actual shape of the relationship might not be strictly linear and its particular shape might give hints as to the details of the causal mechanisms involved.

To help better understand these relations, the *z*-scored values of UV-B incidence (mean and sd) back-map to their raw values as follows. For *UV-B mean*: 0.0 → 208.5*mW*/*m*^2^, −4.75 → 70.89*mW*/*m*^2^, and 1.04 → 238.6*mW*/*m*^2^; in general, $UV_{raw}\left[mW/m^{2}\right]=208.5\left[mW/m^{2}\right]+UV_{z}\cdot 29.0\left[mW/m^{2}\right]$. For *UV-B sd*: 0 → 16.4*mW*/*m*^2^, −0.87 → 1.13*mW*/*m*^2^, and 3.13 → 71.2*mW*/*m*^2^; in general, $UV_{raw}\left[mW/m^{2}\right]=16.4\left[mW/m^{2}\right]+UV_{z}\cdot 17.5\left[mW/m^{2}\right]$. Supplementary Figures 93–95 show the relationship between UV-B incidence (mean and sd) and the presence of a specific word for “blue” globally and per macroarea.

**Figure 4:**
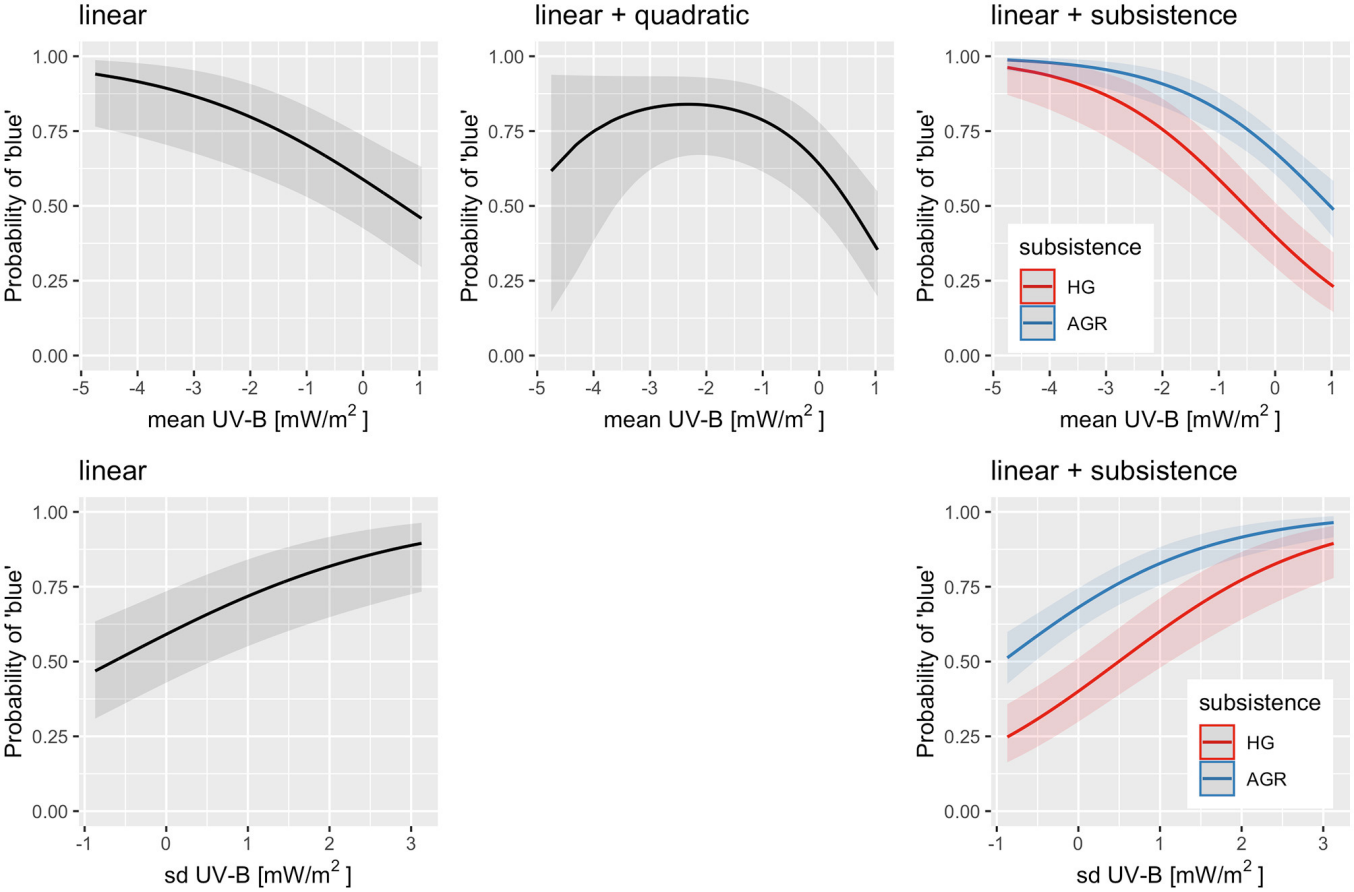
The predictions generated by the fitted regression models of the probability of a dedicated word for “blue” on UV-B mean **(top row)** or UV-B sd **(bottom row)** by themselves (first two columns) or with subsistence (third column). There is no quadratic model for UV-B sd as it fitted slightly worse than the linear one. The horizontal axis represents the *z*-scored UV-B incidence (mean or sd) while the vertical axis the predicted probability of “blue.” The solid curves are the estimates and the shaded areas their 95% CIs. When subsistence is included (third column) the two curves represent hunter-gatherers (HG; red) and the agriculturalists (AGR; blue). Figure generated using R version 4.2.3 (2023-03-15) and package ggplot2 (version 3.4.1).

Polynomial logistic regression up to degree 3 in the fixed effect were conducted (both Bayesian and non-Bayesian) while controlling for family and macroarea as random effects, and the results are extremely similar. For *UV-B mean*, the linear model finds a clear negative relationship, where going from the minimum mean UV-B incidence of 70.9*mW*/*m*^2^ to the maximum of 238.6*mW*/*m*^2^ is associated with a drop in the probability of “blue” from about 94% (with a 95%CI of [77%, 99%]) to about 46% (with a 95%CI of [30%, 63%]). However, the model with both linear and quadratic effects fits the data marginally better [vs. linear: $\chi_{\left(1\right)}^{2}=4.93$, *p* = 0.026, Δ*AIC* = 2.9, Δ*BIC* = −1.8], which suggests that the relationship might not be linear (or even monotonic) at low mean UV-B incidences (the confidence interval is very wide), and instead the probability of “blue” might plateau (or reach a maximum) at about 140*mW*/*m*^2^ of about 84% [67, 93%] and falls off to 35% [20, 55%] for the maximum mean UV-B incidence, and also (but see the very wide 95%CI!) toward 62% [15, 94%] for the minimum mean UV-B incidence. However, the “dip” at lower mean UV-B incidences (higher latitudes) could be an artifact of hunter–gatherer populations whose languages tend to lack a word for “blue.” Therefore, the same polynomial regression but also including all the interactions with *subsistence* was also fitted. With these, manual model simplification suggests that the best model (Δ*AIC* = 123.6, Δ*BIC* = 124.4) actually comprises the linear effect of UV-B mean and the independent contribution of subsistence (i.e., with no interaction between the two). For *UV-B sd*, the linear and the quadratic models fit equally well [$\chi_{\left(1\right)}^{2}=0.84$, *p* = 0.36, Δ*AIC* = −1.2, Δ*BIC* = −5.9], so we will use the linear model when going from the minimum sd UV-B incidence of 1.1*mW*/*m*^2^ to the maximum of 71.2*mW*/*m*^2^ is associated with an increase in the probability of “blue” from about 47% [31, 63%] to about 90% [73, 96%]. Adding the independent contribution of subsistence results in an even better fit (Δ*AIC* = 129.7, Δ*BIC* = 125.7). Figure 4 shows the predictions of these models: it can be seen that including subsistence removes the need for a quadratic effect in UV-B mean and highlights the overall lower probability of a specific word for “blue” in hunter–gatherer languages but no detectable interaction (within the limits of the dataset) between subsistence and UV-B incidence.

## 5. Discussion and conclusion

This extension of the database resulted in a massive increase in the language families covered, and in the languages within families and macroareas. A slight majority of the languages in the extended database do have a specific word for “blue” and are spread across a wide range of UV-B incidences. The other potentially relevant variables were also extended to all of or to a sizable proportion of the data. This resulted in a much better coverage of small families and isolates, and of Australia and Papunesia, offering a much more representative sample of present-day linguistic diversity and increased statistical power relative to the original study (Josserand et al., 2021). Moreover, by increasing the available data for several large families, it made possible the application of various phylogenetic methods as well as the addition of piecewise path analysis and of Structural Equation Models with latent variables.

Overall, the large set of diverse methods used overwhelmingly supports the *a priori* hypothesis of a negative effect of mean UV-B incidence on the probability that a language has a specific word for “blue.” First, this negative effect is found in the individual logistic regression of “blue” on the mean UV-B incidence. Second, it also appears in the mediation analysis, where mean UV-B incidence fully mediates the overall effect of latitude on “blue,” and in the various path and Structural Equation models. Third, the phylogenetic methods provide some evidence of correlated evolution and of a negative phylogenetic effect in several large families and in two “global” language phylogenies. Moreover, it emerged that the annual variation in UV-B incidence is strongly negatively correlated with mean UV-B incidence (as expected due to astronomic considerations) and, in most models, both tend to explain very similar variation, resulting in one “removing” the other from the model when included simultaneously (most often, the variation is retained and the mean is “dropped”). With this in mind, variation in UV-B has a clear positive effect on “blue” in the individual logistic regression, in the mediation, path and Structural Equation analyses, and in the suggestive signal in the phylogenetic analyses. Moreover, modeling the mean and variation in UV-B incidence as indicators of the latent UV-B incidence recovers the expected effect of this latent variable on “blue.” Thus, while most techniques do find a “significant” overall negative effect of mean UV-B on “blue,” there is none among the remaining techniques that supports an overall positive effect, and even among the techniques that suggest no effect, this seems to be due to overlapping variance with other predictors. While “global” language phylogenies have serious issues and it is unclear to what degree they reflect the “vertical” historical connections between languages (especially beyond the level of established language families), the fact that two such “global” phylogenies, constructed using widely different methods and datasets, find overwhelming support for the negative effect of mean UV-B on “blue” after controlling for “Galton's problem” at this global scale is more than encouraging. The fact that a phylogenetic effect was detected for certain families suggests that the effect is indeed diachronic and may play out at the time-scale of within-family divergence (i.e., thousands to hundreds of years). It is important, however, to point out that the measurements used here for UV-B incidence (but also for climate, humidity and distances to bodies of water) are present-day measurements that, on the one hand, may deviate quite strongly from their values during the periods of interest (presumably more so for some regions than for others) and, on the other, represent a snapshot of a variable timeseries (again, in a region- and time-dependent manner). In particular, the UV-B incidence used may not accurately reflect historical values due to the human-induced ozone layer depletion and its depletion and its slow recovery following the “Montreal Protocol” from 1978 (see https://en.wikipedia.org/wiki/Montreal_Protocol), very probably with strong variation across geographic regions (e.g., Australia), but it is unclear how we can extrapolate its values back to the pre-industrial period globally and with the required spatio-temporal resolution (Lindfors et al., 2007; den Outer et al., 2010; Čížková et al., 2018).

Climate and humidity seem to have a much less clear and consistent effect in this larger dataset. The previously found negative effect of distance to lakes on *blue*, which Josserand et al. (2021) were careful not to over-interpret, is much weaker but still arguably discernible at least as a trend in this extended dataset, especially when using mediation, path analyses, latent variable SEM, and even phylogenetic regression, but the mediation analyses conducted specifically with this variable in mind seem to suggest that this might be due to it being related to latitude and UV-B incidence (this relationship probably reflects the vagaries of the current disposition of landmasses on Earth, on the one hand, and the causal links among latitude, climate, and the density of lakes and UV-B incidence, on the other).

However, there might be an overall weak effect of subsistence (practicing agriculture increases the probability of “blue”) and possibly of population size. Nevertheless, given that both are far-from-perfect proxies for the unmeasured (and arguably extremely hard to measure) cultural complexity and capture different aspects thereof (Josserand et al., 2021), the fact that there is a “switch” in their contribution to “blue” between Josserand et al. (2021) and this study should not be taken too literally and, coupled with the results of Structural Equation Modeling including a latent “cultural complexity”, gives extra support to the positive influence of cultural complexity on “blue.” Interestingly, subsistence seems to be required to properly explain the shape of the relationship between UV-B incidence and “blue,” as it helps account for the few northern populations who do not have a specific term for “blue.” It turns out that these apparent exceptions do, in fact, support the *a priori* hypothesis which states that high UV-B incidence generates a negative pressure against a specific term for “blue,” but, in contrast, low UV-B incidence does not induce any specific bias for or against “blue” and, instead, allows other factors to “play freely,” as it were. And indeed, this is what it was found: the relationship between UV-B incidence and “blue” is negative linear overall if one accounts for hunter–gatherer populations living with low UV-B incidence but do not have a dedicated word for “blue.” This is highly similar to other cases reported in the literature, in particular concerning the positive effect of a small or absent alveolar ridge prominence on click consonants (Moisik and Dediu, 2017) and the negative effect of an edge-to-edge bite on labiodentals (Blasi et al., 2019), where the bias is effectively asymmetric. Moreover, the finding that the relationship is very probably linear should help guide the search for the detailed causal mechanisms involved, suggesting an additive effect of UV-B incidence on the perception of blue as well as an additive effect on language across time. Nevertheless, as highlighted in Josserand et al. (2021), we must keep in mind that this is very likely a multi-factorial complex causal process involving multiple temporal and organizational scales, ranging from the intra-individual physiological lens brunescence and the associated perceptual and cognitive mechanisms of compensating and adapting to it to the large-scale presumably cross-generational and inter-individual language change in structured communities reflecting the decreased perception of “blue” among its most affected (older) members. While many of these components are still in need of thorough study and require inter-disciplinary and methodologically diverse approaches, the conversation has already started (see, for example, Josserand et al., 2021, the recent technical comment to it in Hardy et al., 2023 and our response in Josserand et al., 2023, touching on these aspects).

In conclusion, enlarging the database using primary and secondary sources of data vastly increased its representativity of the world's linguistic diversity and allowed the application of phylogenetic methods to investigate the diachronic component of the negative influence of UV-B incidence on the existence of a specific word for “blue.” It can only be highlighted that such extensions are an essential component of science and that, in this case, it supports and refines the previous findings of Josserand et al. (2021) and the original proposal (Lindsey and Brown, 2002) of a negative effect of UV-B incidence on the probability that a language has a specific word for “blue.”

## Data availability statement

The data and code needed to reproduce the results reported here, as well as the full analysis report can be found at: https://github.com/ddediu/colors-UV-update.

## Author contributions

DD designed the research, checked, corrected, collected data, performed the analyses, and wrote the study.
