# Supplementary material for: Ultraviolet light affects the color vocabulary: evidence from 834 languages
Source: Front Psychol. 2023 Jun 2;14:1143283. doi: 10.3389/fpsyg.2023.1143283 (PMC10273402; doi:10.3389/fpsyg.2023.1143283)
Supplement: Supplementary file 1 [file Data_Sheet_1.PDF]

Supplementary Material for “Ultraviolet light affects the color vocabulary: evidence from 834 languages”

Please note that the full results can be found in the **full `HTML` report** available in the GitHub repository ddediu/colors-UV-update. This report can be generated by running the `Rmarkdown` script available from the same GitHub repository ddediu/colors-UV-update.

1 SUPPLEMENTARY DATA

All the data are available in open formats (`CSV`, `text`) with detailed explanations in the associated GitHub repository.

2 SUPPLEMENTARY TABLES AND FIGURES

2.1 Supplementary Tables

Table S1: The list of languages (gives by their glottocodes and names) for which dictionaries (given by their reference) and expert opinions (expert name and expertise; at the time of data collection, all were located at the Laboratoire Dynamique du Langage, Université Lyon 2/CNRS, Lyon, France) were used. For help with the resources (dictionaries, word lists) concerning the Australian languages, thanks to Jayden Macklin-Cordes.

Glottocode	Language name	Source	’blue’?
here1252	*Herero*	Roberson et al. (2004)	no
sgaw1245	*S’gaw Karen*	Karl Seifen (*Tai-Kadai*)	yes (✓)
nucl1310	*Burmese*	-”-	yes (✓)
cent1989	*Khmer*	-”-	yes (✓)
monn1252	*Mon*	-”-	yes (✓)
viet1252	*Vietnamese*	-”-	no
mlab1235	*Mlabri*	-”-	no
laoo1244	*Lao*	-”-	yes (✓)
thai1261	*Central Thai*	-”-	yes (✓)
shan1277	*Shan*	-”-	yes (✓)
soca1235	*Swo (Cameroon)*	Tessa Vermeir (*Swo*)	no
halh1238	*Halh Mongolian*	Rigele Na (*Solon Evenki*)	yes (✓)
solo1263	*Tungusic*	-”-	yes (✓)
-pending-	*Black Hmong*	Lea Mouton (*Black Hmong*)	no
ngal1292	*Ngalkbun*	Evans et al. (2004)	no
alya1239	*Alyawarr*	Green (1992)	no
remb1249	*Rembarrnga*	Saulwick et al. (2003)	no

Continued on next page

Table S1 – continued from previous page

Glottocode	Language name	Source	'blue'?
gami1243	*Yuwaalaraay-Gamilaraay*	Lissarrague et al. (2003)	yes (✓)
kayt1238	*Kaytetye*	Turpin and Ross (2012)	yes (✓)
pint1250	*Pintupi-Luritja*	Hansen and Hansen (1992)	no
guda1242	*Wambaya-Gudanji*	Nordlinger (1998)	no
wikm1247	*Wik-Mungkan*	Kilham et al. (1986)	no
yuul1239	*Yuulngu*	Zorc (1996)	no
adny1235	*Adnyamathanha*	McEntee and McKenzie (1992)	no
alaw1244	*Alawa*	Sharpe (2001)	yes (✓)
badi1246	*Badimaya*	Marmion and Yamadji Language Centre (1995)	no
bard1255	*Bardi*	Aklif (1999)	no
bayu1240	*Bayungu*	Austin (n.d.)	no
bili1250	*Bilinarra*	Meakins et al. (2013)	no
buna1275	*Bunaba*	Kimberley Language Resource Centre (2010)	no
bura1267	*Burarra*	Glasgow (1994)	no
burd1238	*Burduna*	Burgman (2007a)	no
darl1243	*Paakantyi*	Hercus (1993)	no
dhal1245	*Dhalandji*	Austin (1992a)	no
dhan1270	*Dhangu*	Zorc and Bowern (2004)	no
dhar1247	*Dhargari*	Austin (1992c)	no
djau1244	*Jawoyn*	Merlan and Jacq (2005)	no
djin1253	*Djinang*	Waters (1988)	no
djiw1241	*Djiwarli*	Austin (1992b)	no
dyaa1242	*Dyaabugay*	Robertson and Sommer (1997)	no
erre1238	*Erre*	Birch (2006)	no
gami1243	*Yuwaalaraay-Gamilaraay*	Ash et al. (2003)	no
gupa1247	*Gupapuyngu*	Lowe and Lowe (1976)	no
gura1252	*Guragone*	Green and Nimbadja (2015)	no
gurd1238	*Gurdjar*	Black and Gilbert (1988)	no
guri1247	*Gurindji*	Meakins et al. (2013 2013)	no
iwai1244	*Iwaidja*	Pym and Larrimore (2011)	no
kara1476	*Karadjeri*	McKelson (1989)	no
kuka1246	*Kukatja*	Peile and Valiquette (1993)	yes (✓)
kuku1273	*Kuku-Yalanji*	Hershberger and Hershberger (1982)	no
lara1258	*Laragia*	Harvey (2004)	no
lard1243	*Lardil*	Hale and Leman (1997)	no
mang1382	*Mangerr*	Birch (2006)	no
mara1385	*Marra*	Heath (1981)	no
maun1240	*Mawng*	Singer et al. (2015)	no
muru1266	*Muruwari*	Oates (1992)	yes (✓)
naka1260	*Nakara*	Eather et al. (2005)	no
			Continued on next page

Table S1 – continued from previous page

Glottocode	Language name	Source	'blue'?
ngad1258	*Ngadjunmaya*	Wangka Maya Pilbara Aboriginal Language Centre (2008)	yes (✓)
ngan1295	*Ngandi*	Heath (1978)	no
ngar1284	*Ngarinyin*	Coate and Elkin (1974)	yes (✓)
ngar1296	*Ngarla*	Brown and Geytenbeek (1991)	no
nugu1241	*Nugunu*	Hercus (1992a)	no
nyam1271	*Nyamal*	Burgman (2007b)	no
nyan1301	*Nyangumarta*	Geytenbeek and Geytenbeek (1991)	no
pini1245	*Lake Carnegie Western Desert*	Blyth (2001)	no
thay1249	*Thayore*	Foote (1992)	yes (✓)
tiwi1244	*Tiwi*	Lee (2013)	no
urni1239	*Urningangg*	Birch (2006)	no
wadj1254	*Wadjiginy*	Ford (1997)	no
wage1238	*Wageman*	Wilson and Harvey (2001)	no
waja1257	*Wajarri*	Mackman (2012)	no
walm1241	*Walmajarri*	Hudson and Richards (1993)	no
wand1263	*Wandarang*	Heath (1980)	no
wany1247	*Wanyi*	Laughren (2016)	yes (✓)
wari1262	*Wariyangga*	Austin (1992d)	no
warl1254	*Warlpiri*	Schwartz (1996)	yes (✓)
wemb1241	*Wemba Wemba*	Hercus (1992b)	yes (✓)
wikn1245	*Wik-Ngathana*	Sutton (1995)	no
wong1246	*Wangkumara*	Robertson (1985)	no
yann1237	*Nhangu*	James and Milingimbi School Literature Production Centre (2003)	no
yany1243	*Yanyuwa*	Bradley (n.d.)	no
yayg1236	*Yaygir*	Morelli (2012)	no
yind1247	*Yinggarda*	Anderson and Richards (n.d.)	no
yinh1234	*Yinhawangka*	Wangka Maya Pilbara Aboriginal Language Centre (2008)	no
yiry1245	*Yir-Yoront*	Alpher (1991)	no
yuwa1242	*Yuwaalaraay*	Ash et al. (2003)	no
yuwa1243	*Yuwaalayaay*	Ash et al. (2003)	no

Table S2: The phylogenetic signal estimates for *blue*. The 1st column gives the language family, the 2nd the actual tree(s) used ("Go" = Glottolg original, "Ge" = Glottolg exponential, "Gu" = Glottolg ultrametric, "summary" = three summary or MCC tree, "posterior" = the sample of posterior trees). The 3rd, 4th and 5th columns give the estimate of Fritz and Purvis' D (Fritz and Purvis, 2010) as well as the p-values of the hypotheses that the estimate is 0 or 1, respectively. The 6th and 7th columns give the estimate and p-value of the phylogenetic variance-covariance matrix $s2$. The last two columns give the estimate of Ives and Garland (2009)'s α and if there are any warning pointing to a negligible phylogenetic signal. One or two stars (* or **) represent significance versus the standard α-levels 0.05 and 0.01 respectively (without any multiple testing correction). Please note that for the posterior samples of trees, instead the mean of the estimates, the percent of significant p-values and the percent of α warnings across the whole sample are given. Some families and trees cannot be used as *blue* is constant across their languages. **Bold** is used to highlight "significant" results.

**Family**	**Tree(s)**	D	$p(D\text{=}0)$	$p(D\text{=}1)$	$s2$	$p(s2\text{=}0)$	α	$W(\alpha)$
Afro-Asiatic	Go	1.13	0.003**	0.65	0.00	0.5	6.39	Y
	Ge	1.00	0**	0.45	0.01	0.49	54.57	N
	Gu	0.56	0.3	0.28	0.33	0.39	0.23	N
	Jäger (2018)	0.47	0.22	0.077	0.98	0.28	6.62	N
Atlantic-Congo	Go	0.33	0.34	0.092	1.50	0.16	0.45	N
	Ge	0.62	0.07	0.11	0.81	0.12	57.79	Y
	Gu	0.73	0.34	0.37	0.63	0.27	0.20	N
	Jäger (2018)	0.80	0.21	0.38	0.36	0.39	26.63	N
	summary	1.58	0.12	0.66	0.44	0.42	0.00	N
	posterior	2.6	99.0%	0.0%	2.8e-09	0.0%	0.012	78.0%
Austroasiatic	Go	1.34	0.085	0.67	0.00	0.5	1.16	N
	Ge	0.91	0.058	0.36	0.00	0.5	62.57	Y
	Gu	0.44	0.42	0.37	0.20	0.45	0.82	N
	Jäger (2018)	1.97	0.053	0.77	0.29	0.44	139.61	N
**Austronesian**	**Go**	**0.33**	0.14	**0.001****	**3.13**	**0.00018****	0.29	N
	**Ge**	**0.57**	0.001**	**0****	**8.60**	**1.3e-07****	55.71	Y
	**Gu**	**-0.03**	0.57	**0****	**1.60**	**0.00076****	0.08	N
	**Jäger (2018)**	**0.22**	0.3	**0****	**3.95**	**0.00041****	12.25	N
	**summary**	**0.37**	0.25	**0.031***	1.32	0.03*	0.66	N
	posterior	0.41	0.0%	33.9%	1.4	99.5%	0.87	0.0%
**Hmong-Mien**	**Go**	**0.05**	0.51	**0.033***	4.09	0.097	0.42	N
	**Ge**	**0.31**	0.27	**0.033***	6.64	0.11	21.60	N
	**Gu**	**-1.13**	0.82	**0.027***	1.26	0.18	0.24	N
	Jäger (2018)	1.27	0.15	0.55	0.80	0.39	135.28	N
**Indo-European**	**Go**	**-0.18**	0.67	**0****	**6.39**	**8.7e-06****	0.17	N
	**Ge**	**0.11**	0.38	**0****	**8.97**	**1.2e-08****	33.72	N
	**Gu**	**-1.53**	0.95	**0****	**1.91**	**0.002****	0.04	N
	**Jäger (2018)**	**0.18**	0.43	**0.046***	**5.04**	**0.03***	2.74	N
	summary	4.48	0.031*	0.88	0.00	0.5	0.00	N

Continued on next page

Table S2 – continued from previous page

Family	Tree(s)	*D*	*p*(*D*=0)	*p*(*D*=1)	*s*2	*p*(*s*2=0)	α	*W*(α)
	posterior	4.2	79.5%	0.0%	0.0029	0.0%	0.00098	0.1%
Nakh-Daghestanian	Go	1.38	0.27	0.56	0.30	0.47	8.99	N
	Ge	1.08	0.21	0.36	0.00	0.5	38.11	N
	Gu	0.32	0.46	0.35	0.03	0.5	0.70	N
	Jäger (2018)	0.64	0.3	0.41	0.00	0.5	44.77	N
Pama-Nyungan	Go	1.46	0.011*	0.82	0.00	0.5	1.06	N
	Ge	1.14	0.001**	0.69	0.00	0.5	57.38	Y
	Gu	0.71	0.33	0.4	0.00	0.5	0.83	N
	Jäger (2018)	0.74	0.18	0.33	0.00	0.5	302.86	Y
	summary	1.16	0.037*	0.6	0.00	0.5	0.00	N
	posterior	1.2	79.8%	0.0%	8.1e-08	0.0%	0.0011	1.2%
**Sino-Tibetan**	**Go**	**0.52**	0.12	**0.022***	**1.64**	**0.048***	0.84	N
	Ge	0.81	0.001**	0.08	0.00	0.5	54.96	Y
	Gu	0.57	0.28	0.23	0.45	0.24	2.86	Y
	Jäger (2018)	0.38	0.32	0.16	1.53	0.16	244.56	N
	summary	0.14	0.48	0.16	2.05	0.13	0.00	N
Tai-Kadai	Go	0.95	0.13	0.43	0.70	0.34	5.91	Y
	Ge	1.08	0.027*	0.5	0.36	0.36	59.29	Y
	Gu	-0.22	0.57	0.17	0.00	0.5	0.51	N
	Jäger (2018)	0.52	0.38	0.28	0.00	0.5	16.04	N
Timor-Alor-Pantar	Go	0.17	0.42	0.094	2.50	0.19	0.58	N
	Ge	0.42	0.21	0.074	2.58	0.11	59.68	Y
	Gu	-0.72	0.72	0.067	0.72	0.29	6.61	N
	Jäger (2018)	0.30	0.46	0.26	0.00	0.5	-	N
Turkic	Go	-1.81	0.71	0.17	0.00	0.5	0.47	N
	Ge	0.39	0.61	0.075	0.74	0.48	1.10	N
	Gu	-4.76	0.63	0.08	2.07	0.3	0.05	N
	summary	-3.47	0.7	0.075	2.97	0.21	0.00	N
	posterior	1.2e+02	0.0%	55.0%	1.2	0.0%	0.026	70.0%
**Uralic**	Go	-32.01	0.82	0.17	0.00	0.5	2.30	N
	**Ge**	**-4.54**	0.81	**0****	5.98	0.11	0.15	N
	**Gu**	**-14.71**	0.89	**0****	2.38	0.23	0.01	N
**“global” (1)**	**Jäger (2018)**	**0.29**	0.015*	**0****	**2.81**	**7.8e-19****	10.62	N
**“global” (2)**	**Bouckaert et al. (2022)**	**0.62**	0**	**0****	**2.39**	**2.3e-19****	0.42	N

Table S3: Ancestral state reconstruction for *blue*. The ACE column gives the probability of *blue* at the root and the best fitting rate model (ER = equal rates, ARD = all rates) as estimated by the ace method (Pagel, 1994). The ReR column gives the probability of *blue* at the root as estimated by the re-routing method (Yang et al., 1995). For the posterior sample, the percent of trees for which the ER is the best-fitting model, and the average probability of *blue* at the root for the ACE and ReR methods are given, respectively. Other conventions as in Table S2. **Bold** highlights the cases where the estimated probability at the root deviates from the uninformative 50%:50% by at least 25%. Some trees could not be used as *blue* is constant.

Family	Tree(s)	ACE	ReR
**Afro-Asiatic**	**Go**	ARD: 50%	**96%**
	Ge	ARD: 50%	50%
	**Gu**	**ER: 100%**	**100%**
	**Jäger (2018)**	**ER: 96%**	**96%**
**Atlantic-Congo**	**Go**	**ER: 76%**	50%
	Ge	ER: 50%	50%
	Gu	ER: 41%	50%
	Jäger (2018)	ER: 46%	46%
	summary	ER: 50%	50%
	posterior	100%: 50%	50%
**Austroasiatic**	**Go**	**ER: 84%**	**79%**
	Ge	ER: 53%	53%
	**Gu**	**ER: 95%**	**93%**
	Jäger (2018)	ER: 50%	50%
**Austronesian**	**Go**	**ER: 98%**	**98%**
	Ge	ARD: 50%	50%
	**Gu**	**ER: 98%**	**99%**
	**Jäger (2018)**	ARD: 38%	**91%**
	**summary**	**ER: 92%**	**92%**
	**posterior**	0%: 50%	**75%**
Hmong-Mien	Go	ER: 41%	41%
	Ge	ER: 50%	50%
	Gu	ER: 48%	50%
	Jäger (2018)	ER: 50%	50%
**Indo-European**	**Go**	**ARD: 93%**	**100%**
	Ge	ARD: 50%	74%
	**Gu**	**ARD: 0%**	**100%**
	**Jäger (2018)**	**ER: 100%**	**100%**
	**summary**	ARD: 50%	**100%**

Continued on next page

Table S3 – continued from previous page

Family	Tree(s)	ACE	ReR
	**posterior**	0%: 50%	**100%**
**Nakh-Daghestanian**	**Go**	**ER: 100%**	**100%**
	**Ge**	ARD: 50%	**95%**
	**Gu**	**ER: 100%**	**100%**
	**Jäger (2018)**	**ER: 100%**	**100%**
**Pama-Nyungan**	**Go**	ARD: 54%	**3%**
	Ge	ARD: 50%	50%
	**Gu**	**ER: 2%**	**1%**
	**Jäger (2018)**	**ER: 7%**	**7%**
	**summary**	ARD: 50%	**0%**
	**posterior**	0%: 50%	**2%**
**Sino-Tibetan**	**Go**	**ER: 99%**	**99%**
	Ge	ARD: 50%	50%
	Gu	ARD: 50%	50%
	Jäger (2018)	ER: 65%	65%
	summary	ER: 50%	71%
**Tai-Kadai**	**Go**	ARD: 50%	**100%**
	Ge	ARD: 50%	50%
	**Gu**	**ER: 100%**	**100%**
	**Jäger (2018)**	**ER: 96%**	**96%**
**Timor-Alor-Pantar**	**Go**	**ER: 19%**	**18%**
	Ge	ER: 50%	50%
	Gu	ER: 50%	50%
**Turkic**	**Go**	**ER: 100%**	**100%**
	**Ge**	**ER: 96%**	**96%**
	**Gu**	**ER: 99%**	**100%**
	**summary**	ARD: 50%	**98%**
	**posterior**	0%: 50%	**97%**
**Uralic**	**Go**	**ARD: 0%**	**91%**
	**Ge**	**ER: 99%**	**99%**
	**Gu**	**ER: 100%**	**99%**
	**Jäger (2018)**	**ER: 100%**	**100%**
	**summary**	**ER: 100%**	**100%**
	**posterior**	**100%: 100%**	**100%**
**“global”** (1)	**Jäger (2018)**	**ER: 100%**	**100%**
“global” (2)	Bouckaert et al. (2022)	ARD: 50%	50%

Table S4: Correlated evolution between *blue* and *UV-B mean*. For Pagel's method (Pagel, 1994) it shows the ΔAIC(independent - dependent) and the LR test's *p*-value. For threshBayes (Felsenstein, 2012), it shows the mean evolutionary correlation, its 95%HDI and its pROPE. It shows a * (and use **bold**) when there is some evidence for correlated evolution, provided either as ΔAIC > 3, LR test's $p < 0.05$, or $0 \notin$ the 95%HDI. For the posterior sample it shows the average estimates and the percent significant *p*-values. Other conventions as in Table S2. Some trees could not be used as *blue* is constant.

Family	Tree(s)	Pagel	threshBayes
Afro-Asiatic	Go	$-0.3\ p = 0.1$	$-0.15\ [-0.50, 0.24]\ p = 0.2$
	Ge	$-4.5\ p = 0.48$	$0.05\ [-0.32, 0.44]\ p = 0.28$
	Gu	$-2.0\ p = 0.2$	$-0.06\ [-0.51, 0.25]\ p = 0.36$
	Jäger (2018)	$-3.3\ p = 0.32$	$-0.04\ [-0.53, 0.36]\ p = 0.24$
Atlantic-Congo	Go	$-4.7\ p = 0.5$	$0.01\ [-0.41, 0.50]\ p = 0.27$
	Ge	$-2.9\ p = 0.28$	$-0.27\ [-0.77, 0.29]\ p = 0.18$
	Gu	$-5.2\ p = 0.59$	$-0.08\ [-0.64, 0.39]\ p = 0.27$
	Jäger (2018)	$-4.6\ p = 0.5$	$-0.08\ [-0.58, 0.42]\ p = 0.26$
	summary	$-6.6\ p = 0.84$	$-0.20\ [-0.83, 0.42]\ p = 0.18$
	posterior	$-7.76\ p : 0.0\%$	$-7.76\ p : 0.0\%$
**Austroasiatic**	**Go**	$-3.5\ p = 0.34$	**−0.52 [−0.89, −0.12] *p* = 0.021***
	Ge	$-5.1\ p = 0.58$	$-0.50\ [-0.79, 0.04]\ p = 0.052$
	Gu	$-6.3\ p = 0.79$	$-0.40\ [-0.76, 0.03]\ p = 0.061$
	**Jäger (2018)**	$-7.1\ p = 0.93$	**−0.55 [−0.87, −0.00] *p* = 0.045***
Austronesian	Go	$-9.7\ p = 1$	$-0.06\ [-0.27, 0.28]\ p = 0.43$
	Ge	$-3.0\ p = 0.29$	$-0.02\ [-0.12, 0.07]\ p = 0.93$
	Gu	$-0.4\ p = 0.11$	$-0.06\ [-0.32, 0.27]\ p = 0.49$
	Jäger (2018)	$-6.6\ p = 0.85$	$-0.03\ [-0.27, 0.24]\ p = 0.47$
	summary	$-6.2\ p = 0.77$	$0.07\ [-0.60, 0.71]\ p = 0.18$
	posterior	$-2.47\ p : 0.0\%$	$-2.47\ p : 0.0\%$
**Hmong-Mien**	**Go**	$-3.3\ p = 0.32$	**−0.63 [−0.94, −0.23] *p* = 0.0079***
	**Ge**	$-4.6\ p = 0.5$	**−0.65 [−0.98, −0.17] *p* = 0.01***
	**Gu**	$-5.3\ p = 0.61$	**−0.52 [−0.86, −0.04] *p* = 0.031***
	Jäger (2018)	$-4.5\ p = 0.47$	$-0.50\ [-0.93, 0.08]\ p = 0.089$
Indo-European	Go	$-2.4\ p = 0.23$	$-0.21\ [-0.55, 0.10]\ p = 0.24$
	Ge	$1.3\ p = 0.053$	$-0.04\ [-0.42, 0.39]\ p = 0.35$
	Gu	$-0.9\ p = 0.13$	$-0.21\ [-0.55, 0.13]\ p = 0.25$
	Jäger (2018)	$-5.7\ p = 0.68$	$-0.26\ [-0.72, 0.12]\ p = 0.17$
	summary	$-6.4\ p = 0.8$	$0.14\ [-0.32, 0.62]\ p = 0.28$
	posterior	$-6.43\ p : 0.0\%$	$-6.43\ p : 0.0\%$
Nakh-Daghestanian	Go	$-5.2\ p = 0.59$	$-0.20\ [-0.67, 0.27]\ p = 0.23$
	Ge	$-6.2\ p = 0.76$	$0.04\ [-0.67, 0.60]\ p = 0.21$
			Continued on next page

Table S4 – continued from previous page

Family	Tree(s)	Pagel	threshBayes
	Gu	$-5.8\ p = 0.71$	$-0.03\ [-0.62, 0.57]\ p = 0.21$
	Jäger (2018)	$-5.5\ p = 0.65$	$-0.06\ [-0.60, 0.53]\ p = 0.22$
Pama-Nyungan	Go	$-3.4\ p = 0.33$	$-0.08\ [-0.44, 0.32]\ p = 0.28$
	Ge	$-2.1\ p = 0.21$	$-0.08\ [-0.45, 0.35]\ p = 0.42$
	Gu	$-5.4\ p = 0.62$	$-0.24\ [-0.48, 0.12]\ p = 0.17$
	Jäger (2018)	$-5.6\ p = 0.67$	$-0.11\ [-0.58, 0.31]\ p = 0.27$
	summary	$-7.6\ p = 0.99$	$-0.11\ [-0.48, 0.25]\ p = 0.3$
	posterior	$-7.56\ p : 0.0\%$	$-7.56\ p : 0.0\%$
Sino-Tibetan	Go	$-6.6\ p = 0.84$	$-0.01\ [-0.40, 0.29]\ p = 0.4$
	Ge	$-5.3\ p = 0.6$	$-0.03\ [-0.16, 0.11]\ p = 0.82$
	Gu	$-6.3\ p = 0.79$	$-0.11\ [-0.44, 0.20]\ p = 0.44$
	Jäger (2018)	$-2.8\ p = 0.27$	$-0.26\ [-0.70, 0.15]\ p = 0.2$
	summary	$-6.9\ p = 0.89$	$-0.18\ [-0.77, 0.41]\ p = 0.18$
Tai-Kadai	Go	$-5.4\ p = 0.63$	$-0.12\ [-0.68, 0.37]\ p = 0.28$
	Ge	$-2.4\ p = 0.23$	$-0.47\ [-0.91, 0.31]\ p = 0.068$
	Gu	$-3.5\ p = 0.35$	$-0.14\ [-0.62, 0.46]\ p = 0.24$
	Jäger (2018)	$-5.8\ p = 0.71$	$-0.00\ [-0.59, 0.54]\ p = 0.24$
Timor-Alor-Pantar	Go	$-4.3\ p = 0.44$	$-0.04\ [-0.54, 0.48]\ p = 0.2$
	Ge	$-6.5\ p = 0.83$	$0.01\ [-0.55, 0.64]\ p = 0.22$
	Gu	$-6.2\ p = 0.78$	$-0.02\ [-0.55, 0.47]\ p = 0.26$
	Jäger (2018)	$-3.0\ p = 0.29$	$-0.07\ [-0.60, 0.33]\ p = 0.33$
Turkic	Go	$-5.7\ p = 0.67$	$0.53\ [-0.10, 0.91]\ p = 0.071$
	Ge	$-5.7\ p = 0.67$	$0.55\ [-0.19, 0.99]\ p = 0.076$
	Gu	$-5.3\ p = 0.61$	$0.31\ [-0.31, 0.92]\ p = 0.12$
	summary	$-6.7\ p = 0.87$	$0.58\ [-0.05, 0.93]\ p = 0.058$
	posterior	$-6.43\ p : 0.0\%$	$-6.43\ p : 0.0\%$
Uralic	Go	$-3.7\ p = 0.37$	$0.47\ [-0.04, 0.85]\ p = 0.05$
	Ge	$-7.0\ p = 0.91$	$0.35\ [-0.23, 0.84]\ p = 0.12$
	Gu	$-4.0\ p = 0.41$	$0.31\ [-0.28, 0.80]\ p = 0.16$
**“global” (1)**	**Jäger (2018)**	**12.0 $p = 0.00051$***	**$-0.12\ [-0.23, -0.08]\ p = 0.41$***
**“global” (2)**	**Bouckaert et al. (2022)**	**9.1 $p = 0.0019$***	**$-0.01\ [-0.02, -0.01]\ p = 1$***

Table S5: Correlated evolution between *blue* and *UV-B sd*.
Same conventions as in Table S4.

Family	Tree(s)	Pagel	threshBayes
Afro-Asiatic	Go	$-6.1\ p = 0.75$	$0.01\ [-0.43, 0.41]\ p = 0.18$
	Ge	$-6.3\ p = 0.78$	$0.09\ [-0.50, 0.44]\ p = 0.22$
	Gu	$-7.1\ p = 0.92$	$0.06\ [-0.34, 0.45]\ p = 0.32$

Continued on next page

Table S5 – continued from previous page

Family	Tree(s)	Pagel	threshBayes
	Jäger (2018)	$-4.8\ p = 0.53$	$0.02\ [-0.41, 0.43]\ p = 0.35$
Atlantic-Congo	Go	$-4.9\ p = 0.53$	$-0.18\ [-0.62, 0.38]\ p = 0.27$
	Ge	$-6.0\ p = 0.73$	$-0.12\ [-0.59, 0.45]\ p = 0.28$
	Gu	$-6.3\ p = 0.8$	$-0.08\ [-0.55, 0.35]\ p = 0.31$
	Jäger (2018)	$-7.5\ p = 0.98$	$-0.15\ [-0.72, 0.35]\ p = 0.25$
	summary	$-6.5\ p = 0.83$	$-0.19\ [-0.81, 0.50]\ p = 0.14$
	posterior	$-7.98\ p : 0.0\%$	$-7.98\ p : 0.0\%$
Austroasiatic	Go	$-3.1\ p = 0.29$	$0.33\ [-0.12, 0.70]\ p = 0.11$
	Ge	$-4.7\ p = 0.52$	$0.11\ [-0.36, 0.59]\ p = 0.28$
	Gu	$-6.2\ p = 0.76$	$0.27\ [-0.19, 0.72]\ p = 0.16$
	Jäger (2018)	$-4.8\ p = 0.52$	$0.25\ [-0.39, 0.78]\ p = 0.13$
**Austronesian**	**Go**	**$7.2\ p = 0.0042$***	$0.33\ [-0.13, 0.69]\ p = 0.15$
	**Ge**	**$4.4\ p = 0.015$***	$0.06\ [-0.18, 0.21]\ p = 0.6$
	**Gu**	**$2.6\ p = 0.031$***	$0.32\ [-0.06, 0.73]\ p = 0.15$
	Jäger (2018)	$-5.4\ p = 0.63$	$0.23\ [-0.02, 0.50]\ p = 0.17$
	summary	$-4.3\ p = 0.45$	$0.28\ [-0.18, 0.79]\ p = 0.12$
	posterior	$-1.77\ p : 0.0\%$	$-1.77\ p : 0.0\%$
**Hmong-Mien**	**Go**	$-3.9\ p = 0.4$	**$0.46\ [0.04, 0.89]\ p = 0.047$***
	Ge	$-5.5\ p = 0.65$	$0.39\ [-0.12, 0.82]\ p = 0.12$
	Gu	$-5.8\ p = 0.69$	$0.37\ [-0.11, 0.73]\ p = 0.11$
	Jäger (2018)	$-6.6\ p = 0.85$	$0.29\ [-0.39, 0.82]\ p = 0.14$
Indo-European	Go	$-2.4\ p = 0.23$	$0.39\ [-0.00, 0.73]\ p = 0.092$
	Ge	$1.3\ p = 0.053$	$0.09\ [-0.40, 0.44]\ p = 0.32$
	Gu	$-0.9\ p = 0.13$	$0.18\ [-0.09, 0.51]\ p = 0.27$
	Jäger (2018)	$-5.7\ p = 0.68$	$0.09\ [-0.26, 0.43]\ p = 0.46$
	summary	$-6.3\ p = 0.8$	$-0.04\ [-0.48, 0.45]\ p = 0.23$
	posterior	$-6.28\ p : 0.0\%$	$-6.28\ p : 0.0\%$
Nakh-Daghestanian	Go	$-4.8\ p = 0.53$	$0.42\ [-0.21, 0.87]\ p = 0.082$
	Ge	$-5.8\ p = 0.69$	$-0.03\ [-0.55, 0.53]\ p = 0.26$
	Gu	$-4.1\ p = 0.42$	$0.11\ [-0.40, 0.72]\ p = 0.29$
	Jäger (2018)	$-5.5\ p = 0.65$	$0.01\ [-0.53, 0.59]\ p = 0.24$
**Pama-Nyungan**	**Go**	$-6.6\ p = 0.84$	**$0.26\ [0.02, 0.67]\ p = 0.2$***
	Ge	$-6.8\ p = 0.89$	$0.16\ [-0.13, 0.47]\ p = 0.29$
	Gu	$-4.2\ p = 0.44$	$0.33\ [-0.04, 0.61]\ p = 0.097$
	Jäger (2018)	$-3.2\ p = 0.31$	$0.33\ [-0.10, 0.66]\ p = 0.1$
	summary	$-3.8\ p = 0.38$	$0.22\ [-0.21, 0.57]\ p = 0.27$
	posterior	$-4.90\ p : 0.0\%$	$-4.90\ p : 0.0\%$
Sino-Tibetan	Go	$-4.8\ p = 0.53$	$0.15\ [-0.15, 0.51]\ p = 0.34$
	Ge	$1.3\ p = 0.054$	$0.01\ [-0.16, 0.19]\ p = 0.82$
	Gu	$-4.0\ p = 0.41$	$0.27\ [-0.20, 0.65]\ p = 0.12$
	Jäger (2018)	$-3.4\ p = 0.33$	$-0.01\ [-0.47, 0.42]\ p = 0.32$
	summary	$-5.2\ p = 0.59$	$0.18\ [-0.40, 0.73]\ p = 0.23$

Continued on next page

Table S5 – continued from previous page

**Family**	**Tree(s)**	**Pagel**	**threshBayes**
Tai-Kadai	Go	$-2.0\ p = 0.2$	$0.19\ [-0.42, 0.68]\ p = 0.17$
	Ge	$-1.5\ p = 0.17$	$0.17\ [-0.39, 0.68]\ p = 0.22$
	Gu	$-1.9\ p = 0.19$	$0.21\ [-0.40, 0.77]\ p = 0.12$
	Jäger (2018)	$-2.9\ p = 0.28$	$0.22\ [-0.41, 0.67]\ p = 0.17$
Timor-Alor-Pantar	Go	$-2.3\ p = 0.22$	$-0.43\ [-0.88, 0.17]\ p = 0.082$
	Ge	$-4.5\ p = 0.48$	$-0.42\ [-0.86, 0.11]\ p = 0.13$
	Gu	$-2.9\ p = 0.28$	$-0.47\ [-0.89, 0.04]\ p = 0.073$
	Jäger (2018)	$-3.0\ p = 0.29$	$-0.08\ [-0.60, 0.46]\ p = 0.25$
Turkic	Go	$-5.7\ p = 0.67$	$-0.47\ [-0.94, 0.17]\ p = 0.097$
	Ge	$-5.7\ p = 0.67$	$-0.61\ [-0.99, 0.07]\ p = 0.055$
	Gu	$-5.3\ p = 0.61$	$-0.50\ [-0.94, 0.07]\ p = 0.063$
	summary	$-6.7\ p = 0.87$	$-0.44\ [-0.96, 0.27]\ p = 0.094$
	posterior	$-6.43\ p : 0.0\%$	$-6.43\ p : 0.0\%$
Uralic	Go	$-3.5\ p = 0.34$	$-0.03\ [-0.66, 0.57]\ p = 0.29$
	Ge	$-6.5\ p = 0.83$	$-0.04\ [-0.69, 0.80]\ p = 0.13$
	Gu	$-4.9\ p = 0.54$	$0.04\ [-0.65, 0.63]\ p = 0.26$
**“global” (1)**	**Jäger (2018)**	**11.3 *p* = 0.0007***	$0.03\ [-0.03, 0.13]\ p = 0.85$
**“global” (2)**	**Bouckaert et al. (2022)**	**9.3 *p* = 0.0017***	$0.01\ [-0.01, 0.02]\ p = 1$

Table S6: Phylogenetic regression of *blue* on various potential predictors considered independently (one per row). 1st column gives the predictor, 2nd and 3rd columns give, respectively, the number (and %) of significant trees and methods and the % of trees and methods with an estimated negative relationship, $\beta < 0$, between *blue* and the predictor (irrespective of it being significant or not) when not including the full posterior samples. Columns 4, 5 and 6 give, respectively, the number (and %) of significant trees and methods, to which family(ies) they belong, and the % of trees and methods with $\beta < 0$. Please note that implicitly the % of trees and methods with a positive relationship, $\beta > 0$, is 100 - columns 3 and 6, as appropriate, and we are interested in predictors for which the ratio $\beta < 0$: $\beta > 0$ deviates from the even split 50%:50%, suggesting a tendency across families, trees and methods.

**Predictor**	**With MCMC trees**		**Without MCMC trees**		
	**# (%) signif.**	**%β <0**	**# (%) signif.**	**Families**	**%β <0**
UV-B (mean)	14 (0.1%)	65.9%	14 (5.6%)	Indo-European, Sino-Tibetan, Uralic, “global” (1), “global” (2)	75.8%
UV-B (sd)	11 (0.1%)	37.4%	11 (4.4%)	Indo-European, Sino-Tibetan, “global” (1), “global” (2)	33.6%
latitude	12 (0.1%)	65.5%	12 (4.8%)	Austronesian, Sino-Tibetan, Uralic, “global” (1), “global” (2)	57.6%
longitude	26 (0.2%)	21.9%	26 (9.9%)	Sino-Tibetan, Tai-Kadai, Turkic, “global” (1), “global” (2)	30.3%
pop. size	26 (0.2%)	21.9%	26 (9.9%)	Sino-Tibetan, Tai-Kadai, Turkic, “global” (1), “global” (2)	30.3%
subsistence	2 (0.0%)	98.3%	2 (0.8%)	“global” (1), “global” (2)	40.9%
climate PC1	108 (0.6%)	80.8%	8 (3.2%)	Austronesian, Pama-Nyungan, Sino-Tibetan, Turkic, “global” (2)	43.5%
climate PC2	16 (0.1%)	7.3%	16 (6.3%)	Afro-Asiatic, Pama-Nyungan, Sino-Tibetan, “global” (2)	52.2%
climate PC3	10 (0.1%)	98.7%	10 (4.0%)	Indo-European, Pama-Nyungan, Turkic, Uralic, “global” (2)	71.0%
humidity (median)	8 (0.0%)	63.4%	8 (3.2%)	Uralic, “global” (1), “global” (2)	55.8%

Continued on next page

Table S6 – continued from previous page

Predictor	With MCMC trees		Without MCMC trees		
	# (%) signif.	%β <0	# (%) signif.	Families	%β <0
humidity (IQR)	10 (0.1%)	47.7%	10 (4.0%)	Hmong-Mien, Indo-European	40.3%
dist. to lakes	17 (0.1%)	71.8%	17 (6.7%)	Afro-Asiatic, Hmong-Mien, Sino-Tibetan, “global” (1), “global” (2)	68.4%
dist. to rivers	5 (0.0%)	63.2%	4 (1.6%)	Austronesian, Tai-Kadai	56.9%
dist. to oceans	0 (0.0%)	17.1%	0 (0.0%)	–	41.1%
dist. to water	12 (0.1%)	11.0%	12 (4.8%)	Sino-Tibetan, “global” (2)	61.7%

2.2 Supplementary Figures

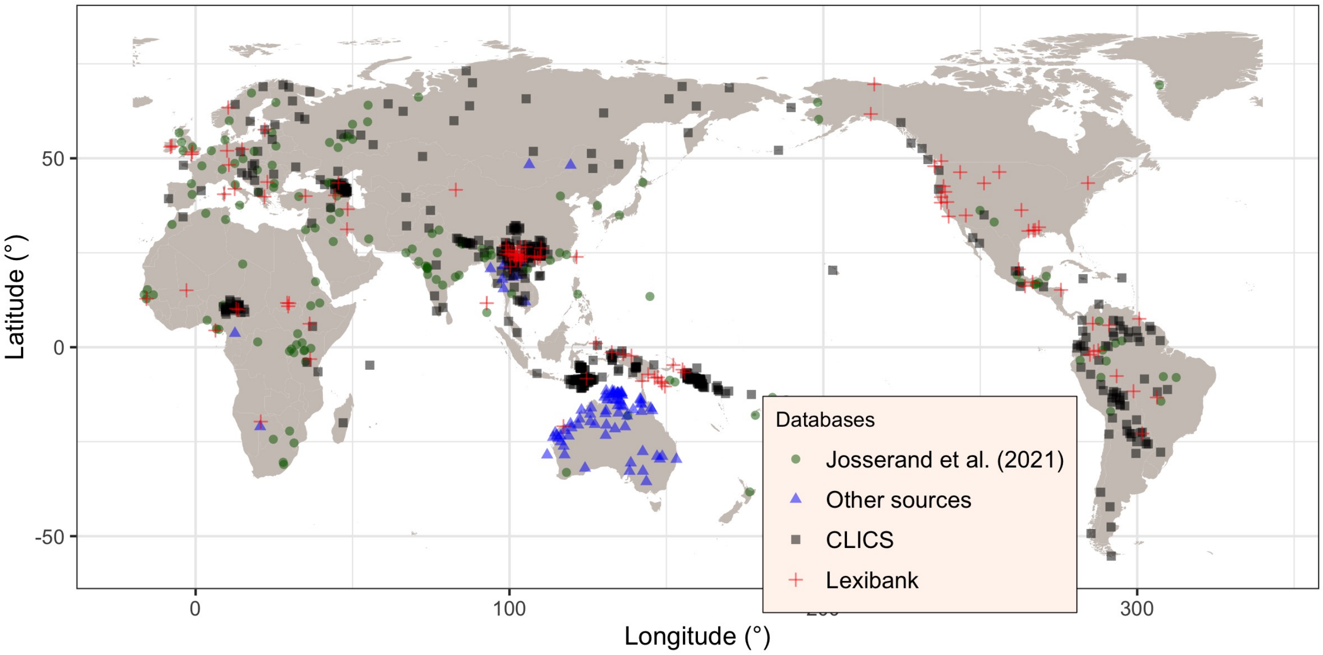

Figure S1. Map showing the datapoints (languages) used in this paper and their source ("Other sources" represent grammars, dictionaries, word lists and expert opinions). Figure generated using `R` version 4.2.3 (2023-03-15) and packages `ggplot2` (version 3.3.6) and `maps` (version 3.4.0), using public domain data from the Natural Earth project as provided by the `R` package `maps`.

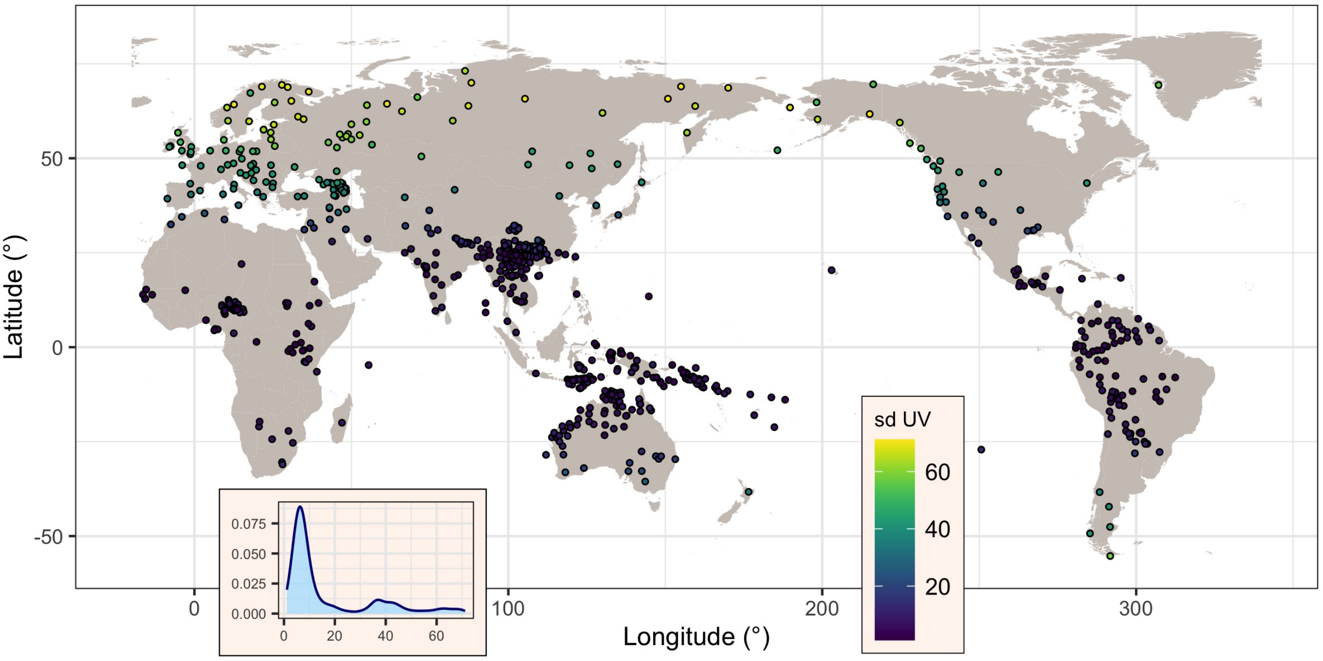

Figure S2. Map of the languages showing, for each, the standard deviation of its UV-B incidence (as given by *TOMS*, in mW/m^2), as well the overall distribution of this variable across all languages (inset). Figure generated using `R` version 4.2.3 (2023-03-15) and packages `ggplot2` (version 3.3.6) and `maps` (version 3.4.0), using public domain data from the Natural Earth project as provided by the `R` package `maps`.

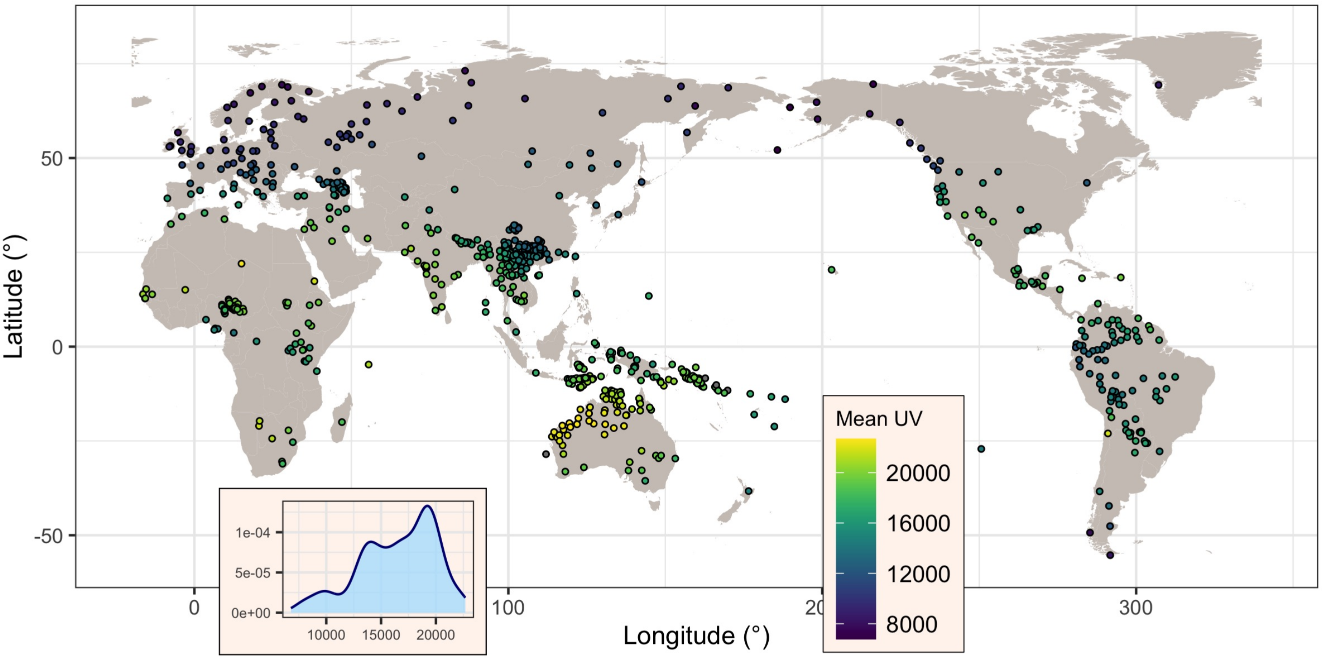

Figure S3. Map of the languages showing, for each, its mean UV-B incidence (as given by *WorldClim*, in kJ/m^{2}day), as well the overall distribution of this variable across all languages (inset). Figure generated using `R` version 4.2.3 (2023-03-15) and packages `ggplot2` (version 3.3.6) and `maps` (version 3.4.0), using public domain data from the Natural Earth project as provided by the `R` package `maps`.

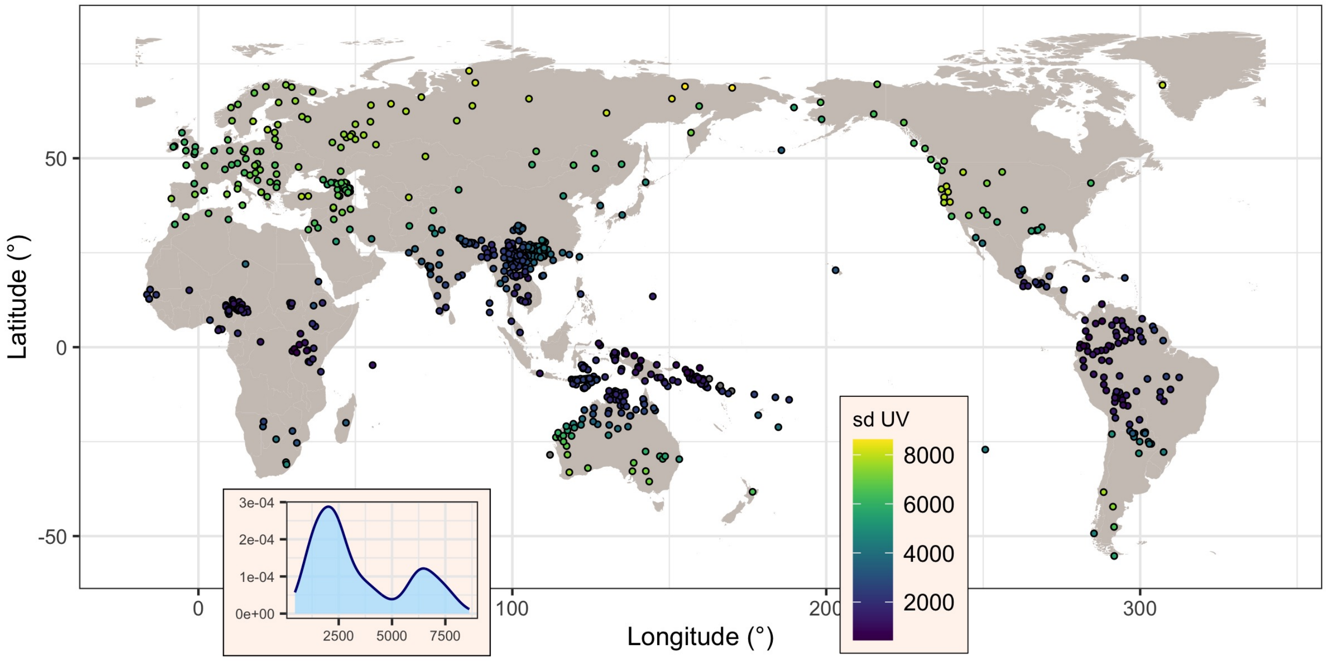

Figure S4. Map of the languages showing, for each, the standard deviation of its UV-B incidence (as given by *WorldClim*, in kJ/m^{2}day), as well the overall distribution of this variable across all languages (inset). Figure generated using `R` version 4.2.3 (2023-03-15) and packages `ggplot2` (version 3.3.6) and `maps` (version 3.4.0), using public domain data from the Natural Earth project as provided by the `R` package `maps`.

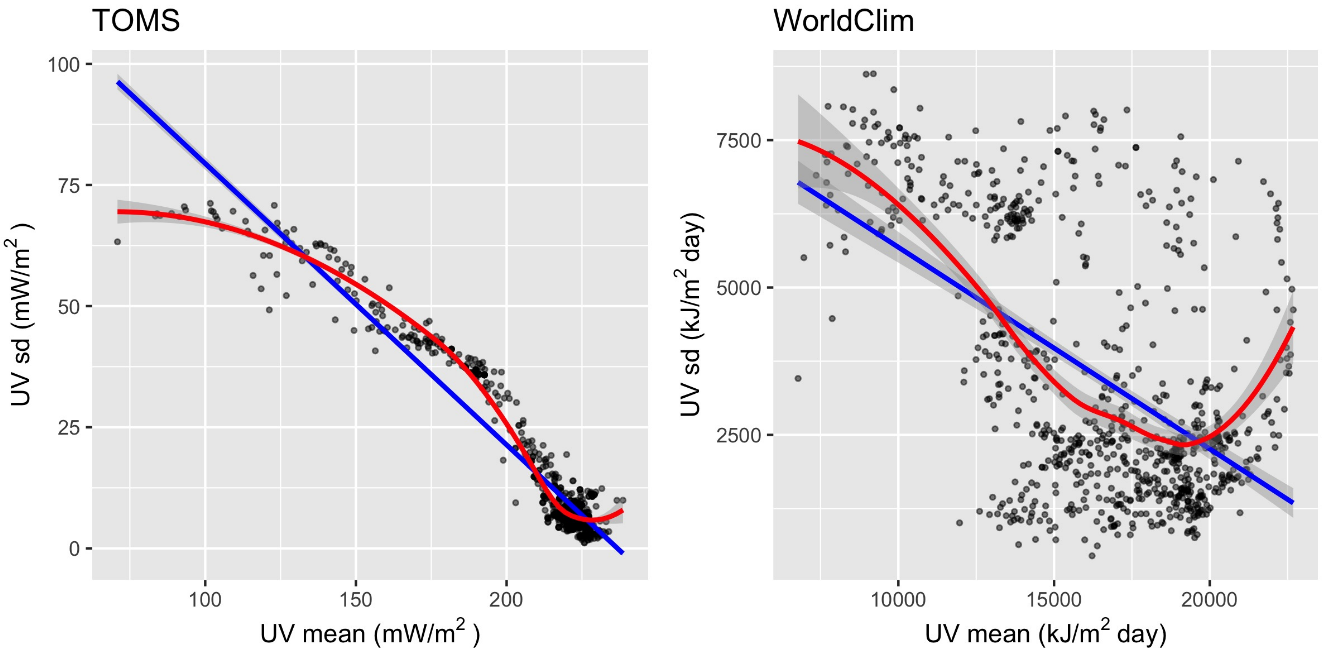

Figure S5. Relationship between the mean (horizontal axis) and sd (vertical axis) of UV-B incidence from *TOMS* (left) and *WorldClim* (right). It can be seen that both are overall negative but non-linear. Figure generated using `R` version 4.2.3 (2023-03-15) and `ggplot2` (version 3.3.6).

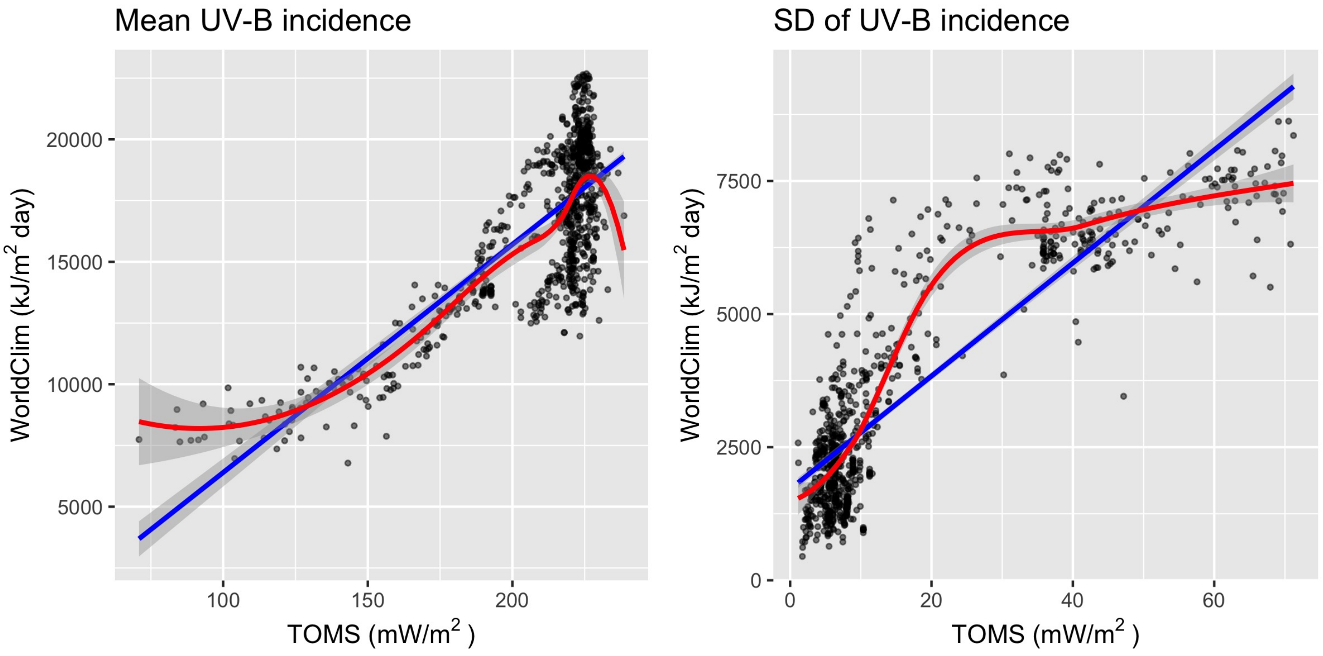

Figure S6. Relationship between the mean (left) and sd (right) of UV-B incidence from *TOMS* (horizontal axis) vs from *WorldClim* (vertical axis). It can be seen that both are overall positive but non-linear. Figure generated using `R` version 4.2.3 (2023-03-15) and `ggplot2` (version 3.3.6).

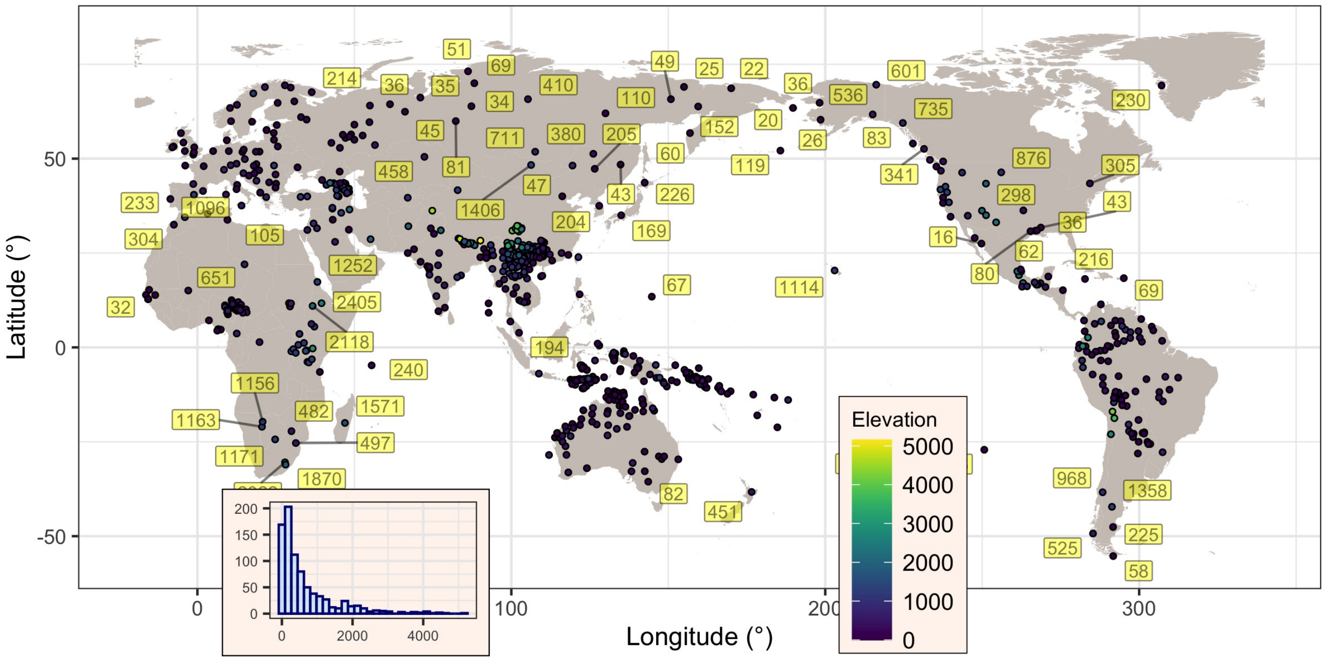

Figure S7. Map of the languages showing, for each, its elevation (altitude), in meters (m), as well the overall distribution of this variable across all languages (inset). The labels show the rounded elevations for a subset of languages, for better orientation. Figure generated using `R` version 4.2.3 (2023-03-15) and packages `ggplot2` (version 3.3.6) and `maps` (version 3.4.0), using public domain data from the Natural Earth project as provided by the `R` package `maps`.

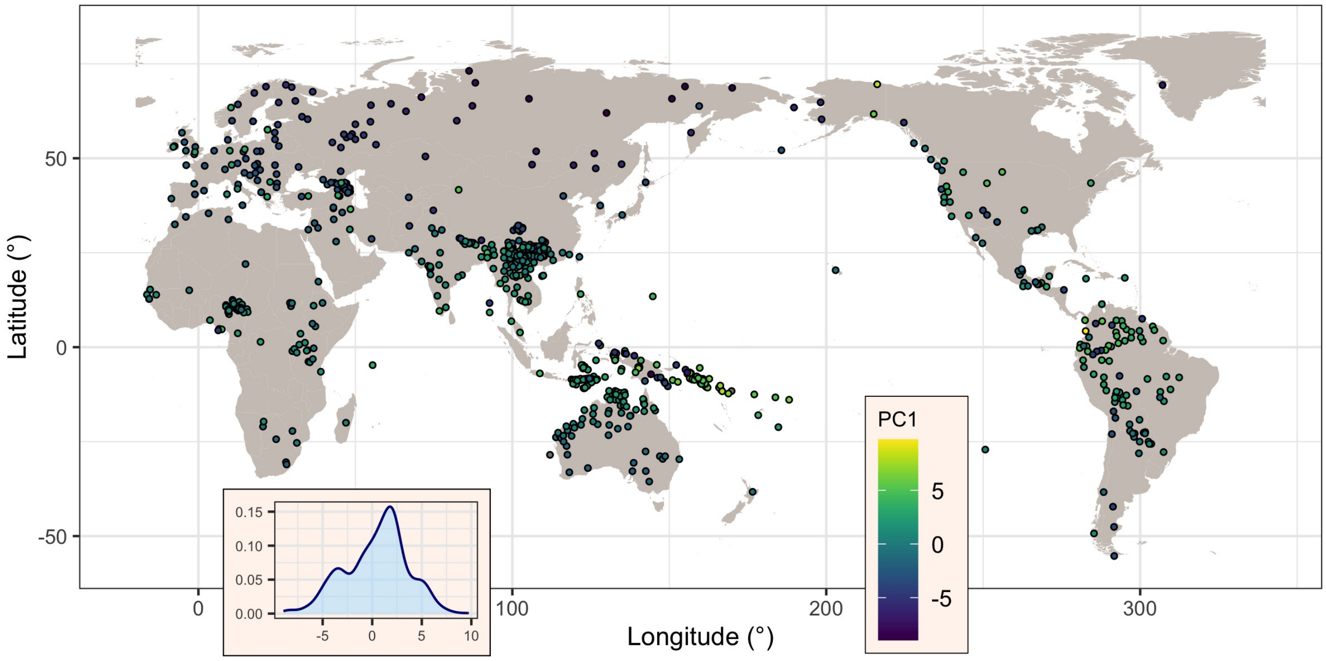

Figure S8. Map of the languages showing, for each, its climate PC1 (explaining 53.9% of the variance), as well the overall distribution of this variable across all languages (inset). Figure generated using `R` version 4.2.3 (2023-03-15) and packages `ggplot2` (version 3.3.6) and `maps` (version 3.4.0), using public domain data from the Natural Earth project as provided by the `R` package `maps`.

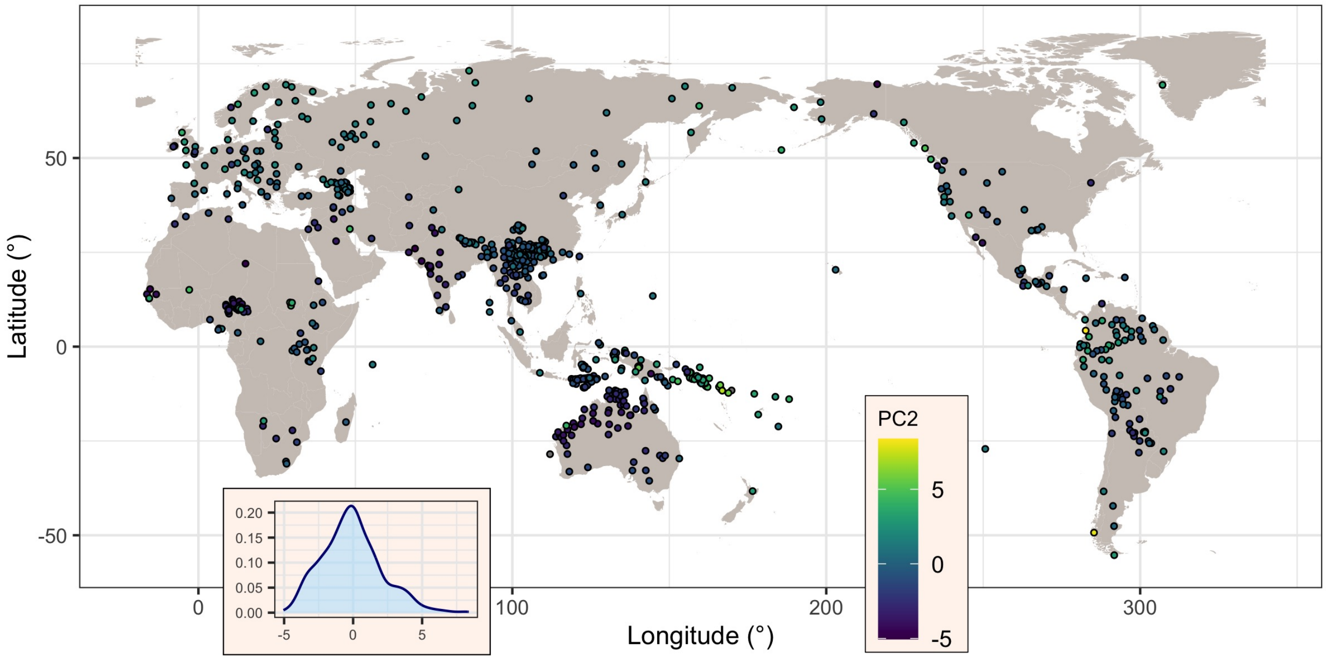

Figure S9. Map of the languages showing, for each, its climate PC2 (explaining 23.3% of the variance), as well the overall distribution of this variable across all languages (inset). Figure generated using `R` version 4.2.3 (2023-03-15) and packages `ggplot2` (version 3.3.6) and `maps` (version 3.4.0), using public domain data from the Natural Earth project as provided by the `R` package `maps`.

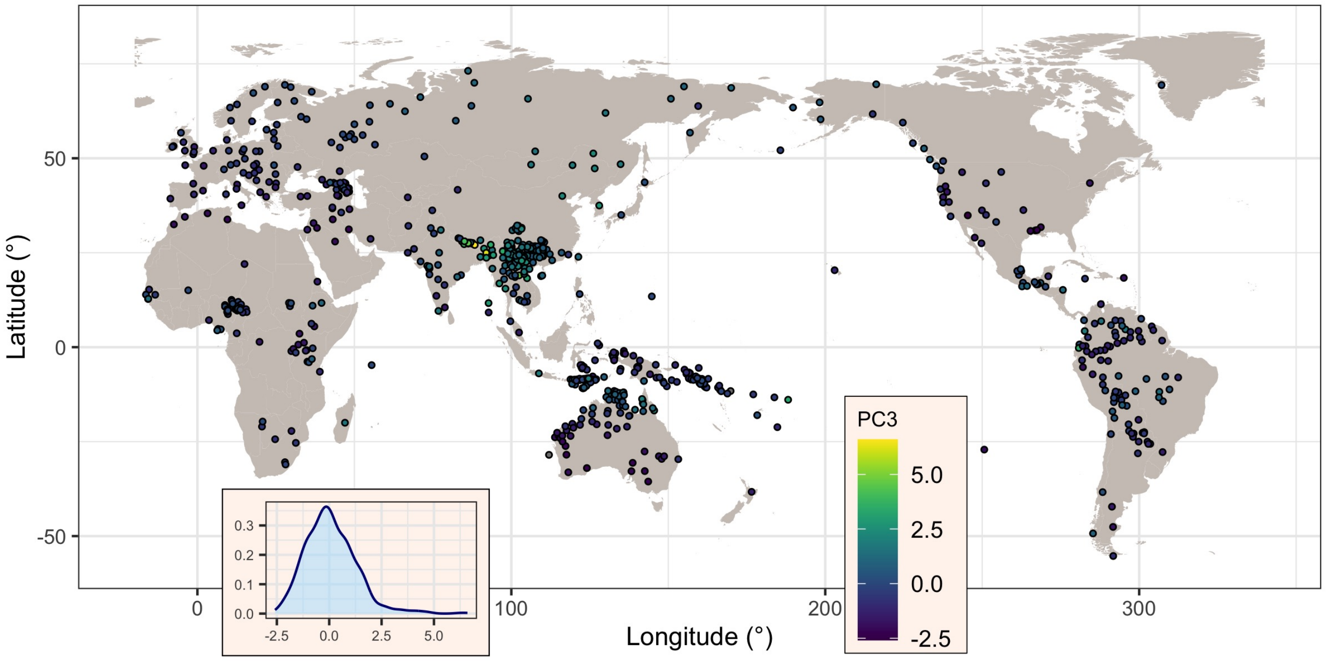

Figure S10. Map of the languages showing, for each, its climate PC3 (explaining 7.1% of the variance), as well the overall distribution of this variable across all languages (inset). Figure generated using `R` version 4.2.3 (2023-03-15) and packages `ggplot2` (version 3.3.6) and `maps` (version 3.4.0), using public domain data from the Natural Earth project as provided by the `R` package `maps`.

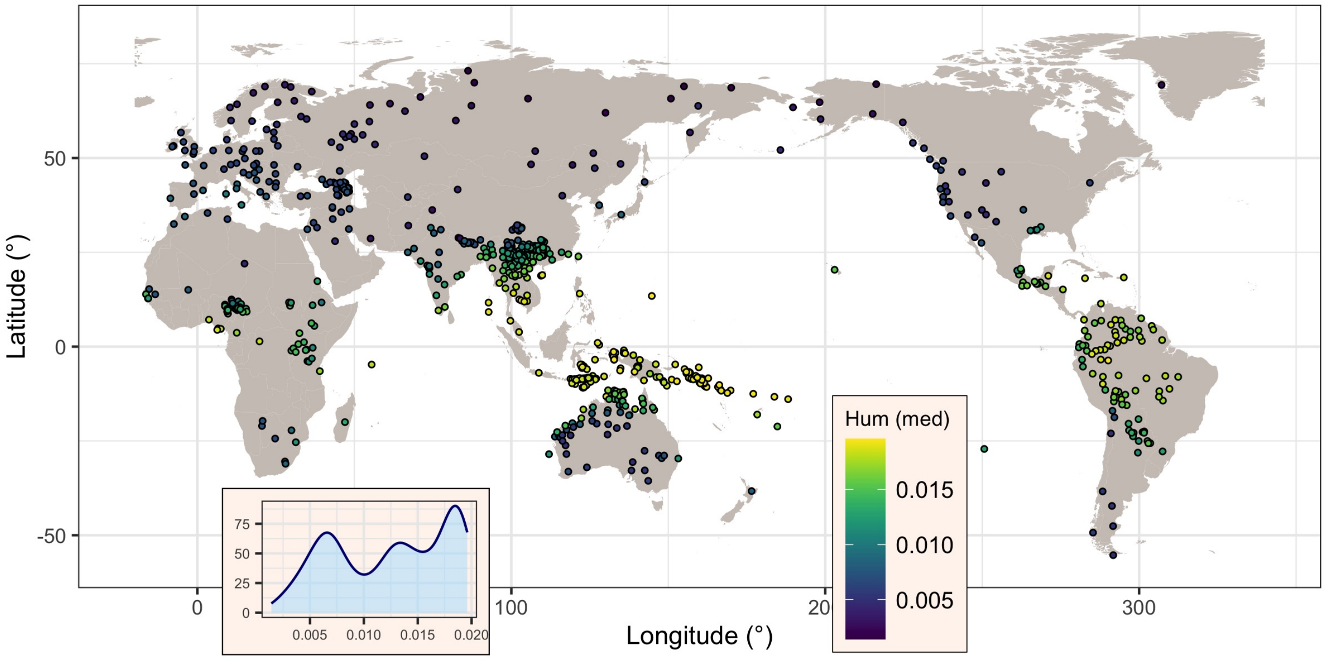

Figure S11. Map of the languages showing, for each, the mean of yearly medians (aka median humidity) measured in grams of vapor per kilogram of air, as well the overall distribution of this variable across all languages (inset). Figure generated using `R` version 4.2.3 (2023-03-15) and packages `ggplot2` (version 3.3.6) and `maps` (version 3.4.0), using public domain data from the Natural Earth project as provided by the `R` package `maps`.

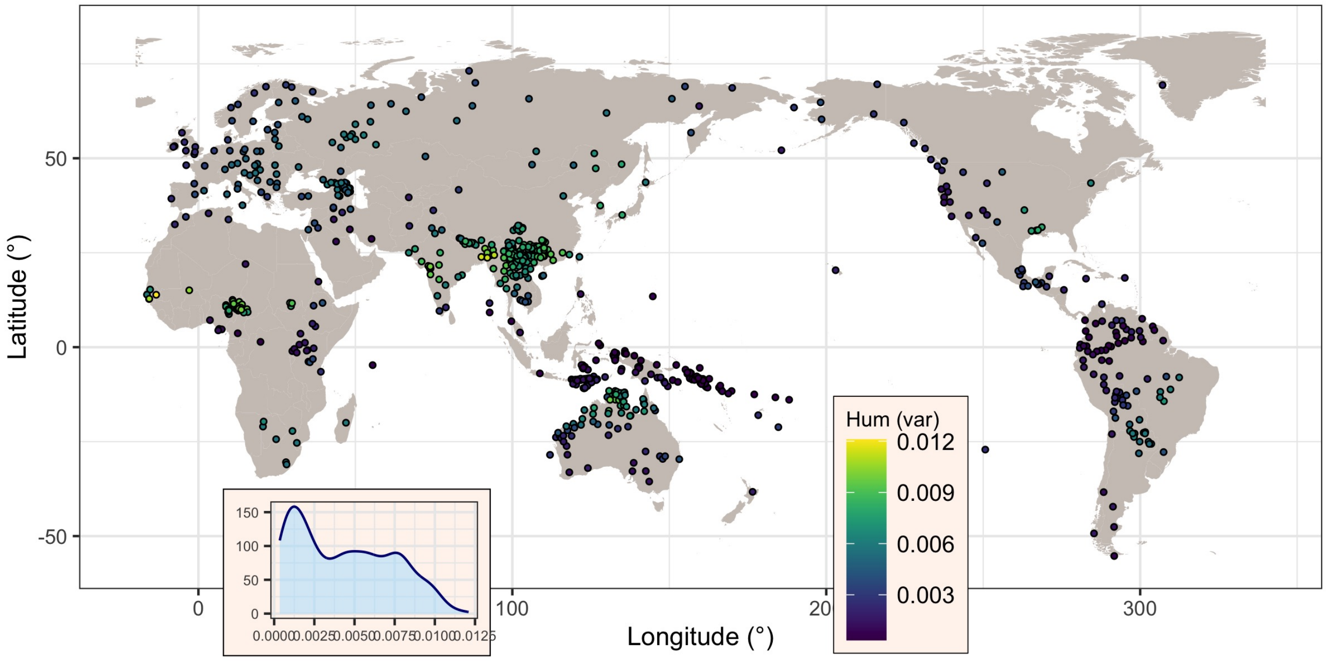

Figure S12. Map of the languages showing, for each, mean of yearly IQRs (aka median variation) measured in grams of vapor per kilogram of air, as well the overall distribution of this variable across all languages (inset). Figure generated using `R` version 4.2.3 (2023-03-15) and packages `ggplot2` (version 3.3.6) and `maps` (version 3.4.0), using public domain data from the Natural Earth project as provided by the `R` package `maps`.

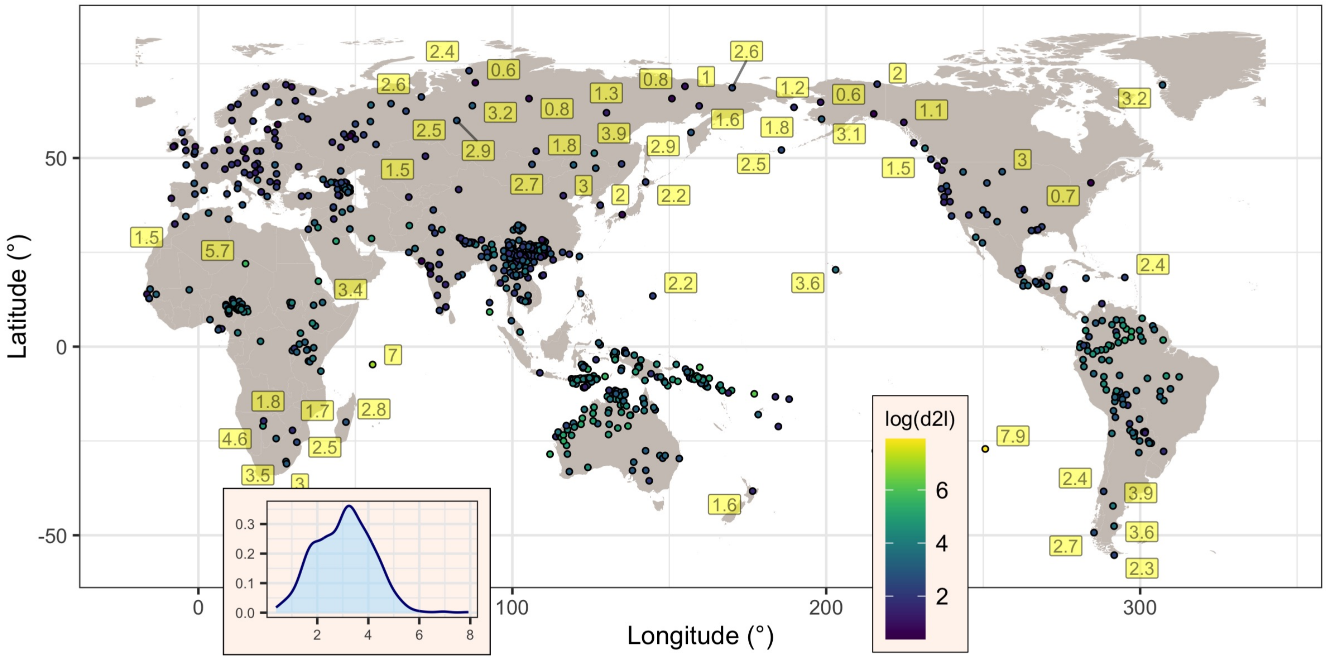

Figure S13. Map of the languages showing, for each, the *log*(distance to the nearest lake), as well the overall distribution of this variable across all languages (inset). The labels show the value (rounded to one decimal place) for a subset of languages, for better orientation. Figure generated using `R` version 4.2.3 (2023-03-15) and packages `ggplot2` (version 3.3.6) and `maps` (version 3.4.0), using public domain data from the Natural Earth project as provided by the `R` package `maps`.

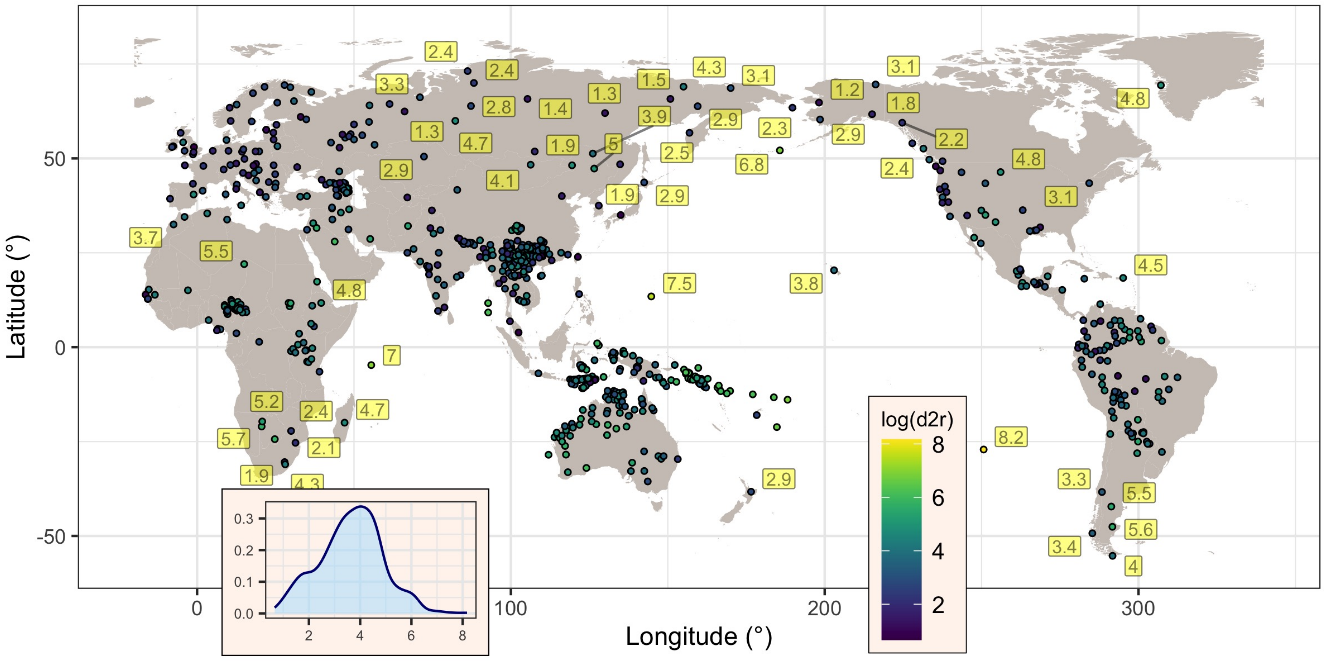

Figure S14. Map of the languages showing, for each, the *log*(distance to the nearest river), as well the overall distribution of this variable across all languages (inset). The labels show the value (rounded to one decimal place) for a subset of languages, for better orientation. Figure generated using `R` version 4.2.3 (2023-03-15) and packages `ggplot2` (version 3.3.6) and `maps` (version 3.4.0), using public domain data from the Natural Earth project as provided by the `R` package `maps`.

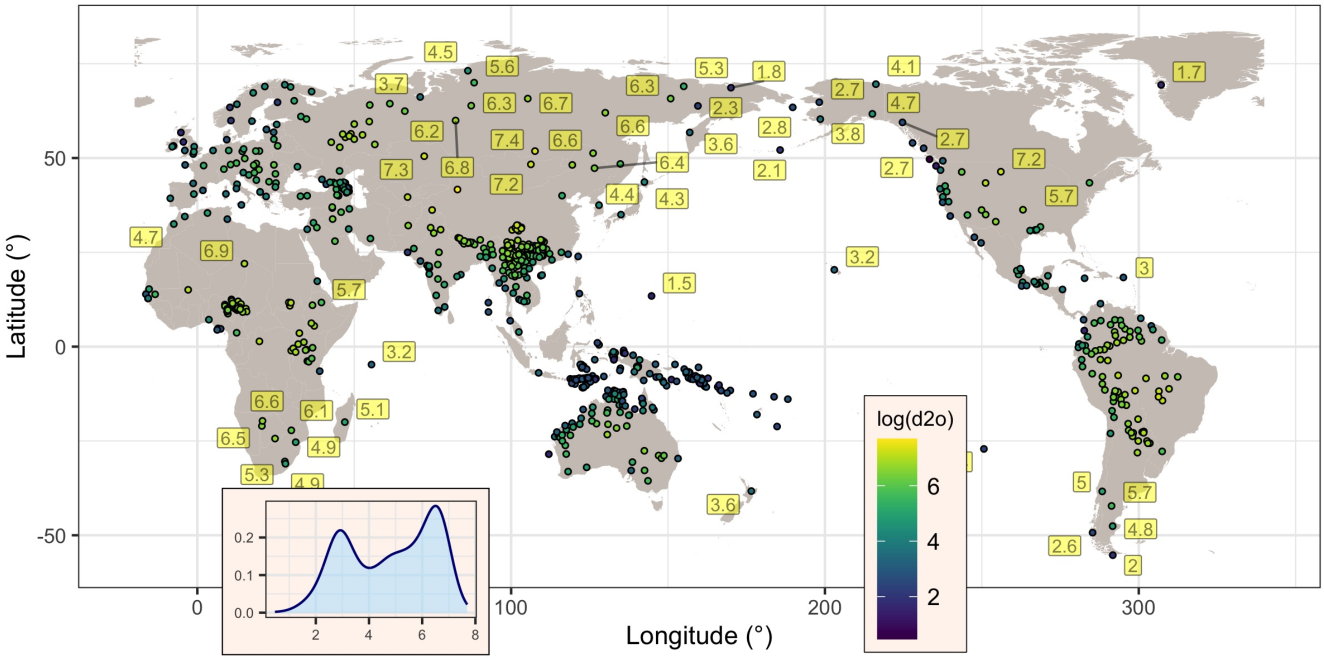

Figure S15. Map of the languages showing, for each, the *log*(distance to the nearest ocean or sea), as well the overall distribution of this variable across all languages (inset). The labels show the value (rounded to one decimal place) for a subset of languages, for better orientation. Figure generated using `R` version 4.2.3 (2023-03-15) and packages `ggplot2` (version 3.3.6) and `maps` (version 3.4.0), using public domain data from the Natural Earth project as provided by the `R` package `maps`.

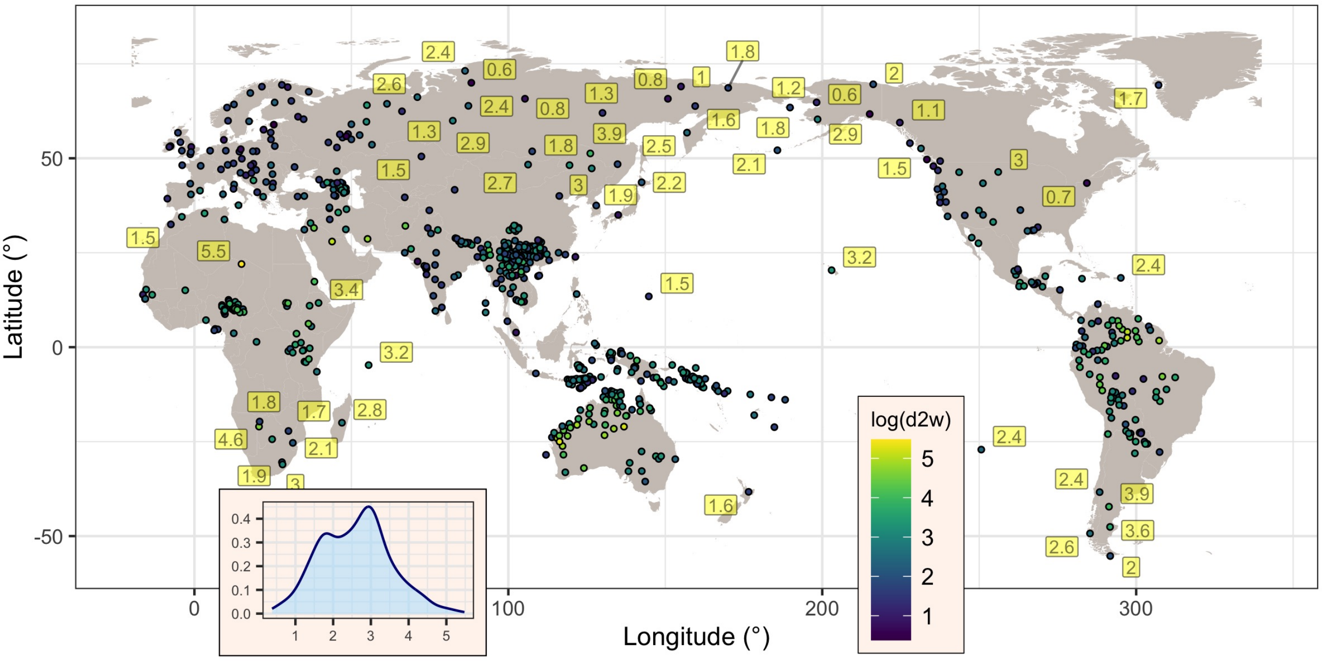

Figure S16. Map of the languages showing, for each, the *log*(distance to the large body of water of any type), as well the overall distribution of this variable across all languages (inset). The labels show the value (rounded to one decimal place) for a subset of languages, for better orientation. Figure generated using `R` version 4.2.3 (2023-03-15) and packages `ggplot2` (version 3.3.6) and `maps` (version 3.4.0), using public domain data from the Natural Earth project as provided by the `R` package `maps`.

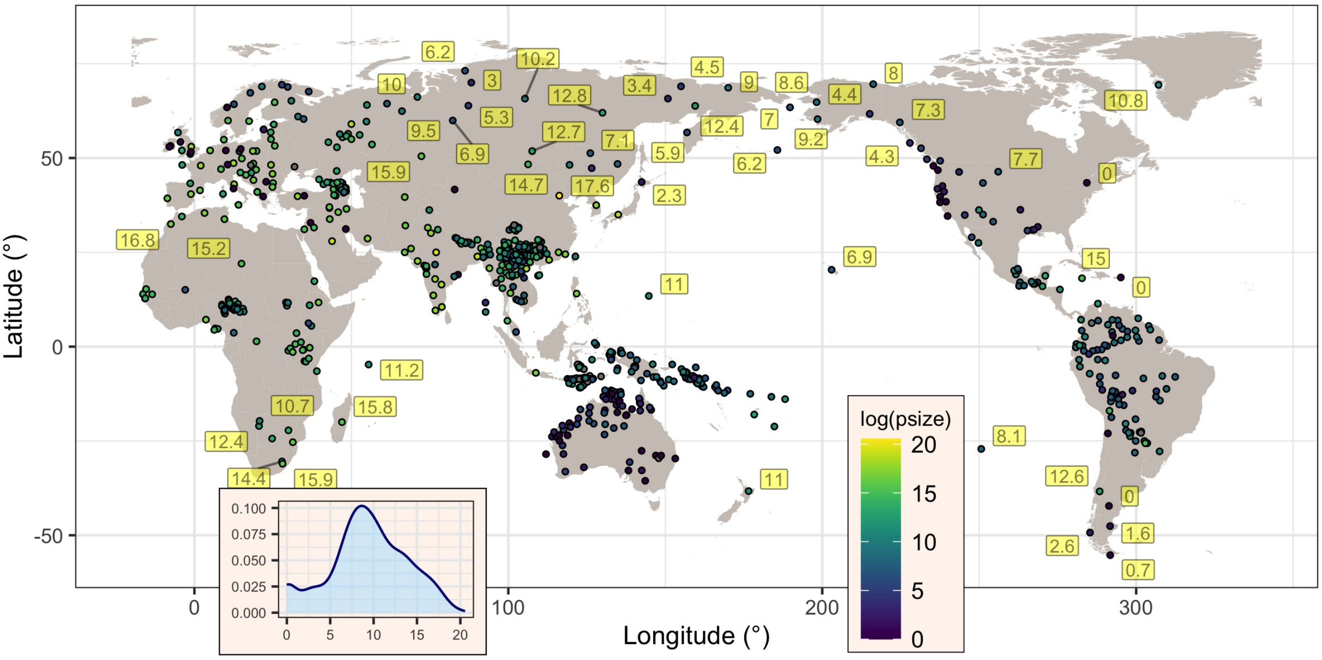

Figure S17. Map of the languages showing, for each, the *log*(population size) as given, essentially, by the *Ethnologue*, as well the overall distribution of this variable across all languages (inset). The labels show the value (rounded to one decimal place) for a subset of languages, for better orientation. Figure generated using `R` version 4.2.3 (2023-03-15) and packages `ggplot2` (version 3.3.6) and `maps` (version 3.4.0), using public domain data from the Natural Earth project as provided by the `R` package `maps`.

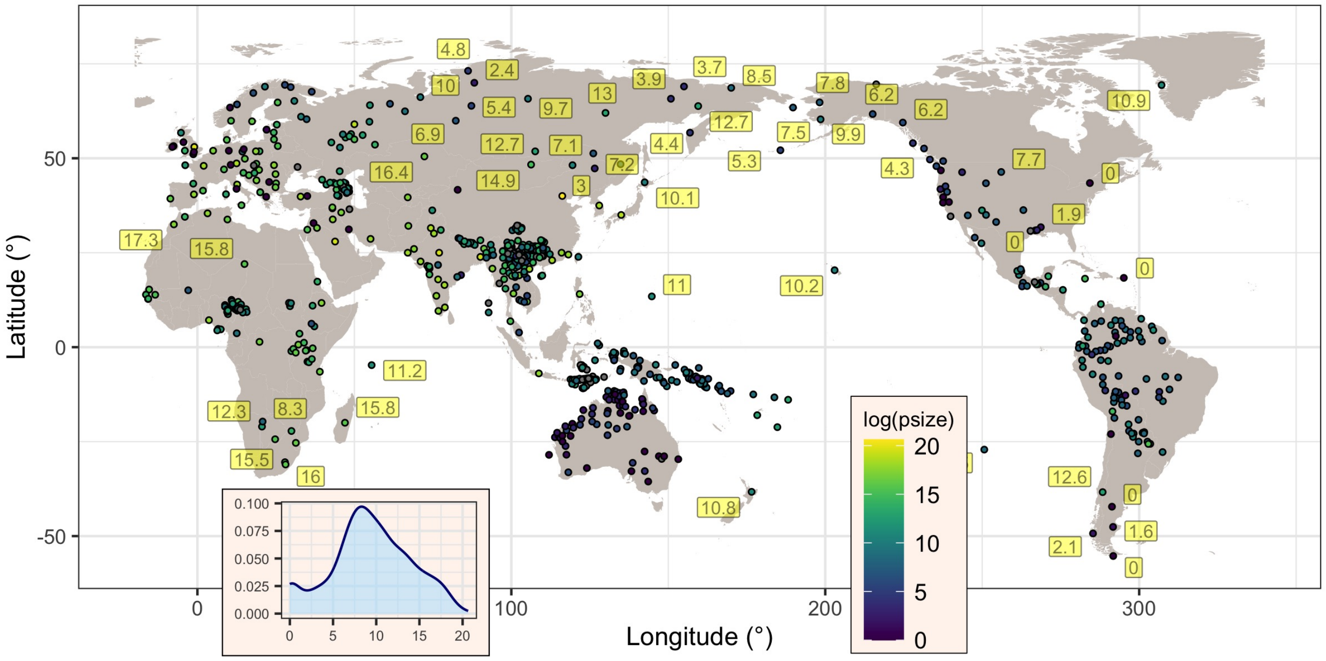

Figure S18. Map of the languages showing, for each, the *log*(population size) as given by *Wikidata*/*Wikipedia*, as well the overall distribution of this variable across all languages (inset). The labels show the value (rounded to one decimal place) for a subset of languages, for better orientation. Figure generated using `R` version 4.2.3 (2023-03-15) and packages `ggplot2` (version 3.3.6) and `maps` (version 3.4.0), using public domain data from the Natural Earth project as provided by the `R` package `maps`.

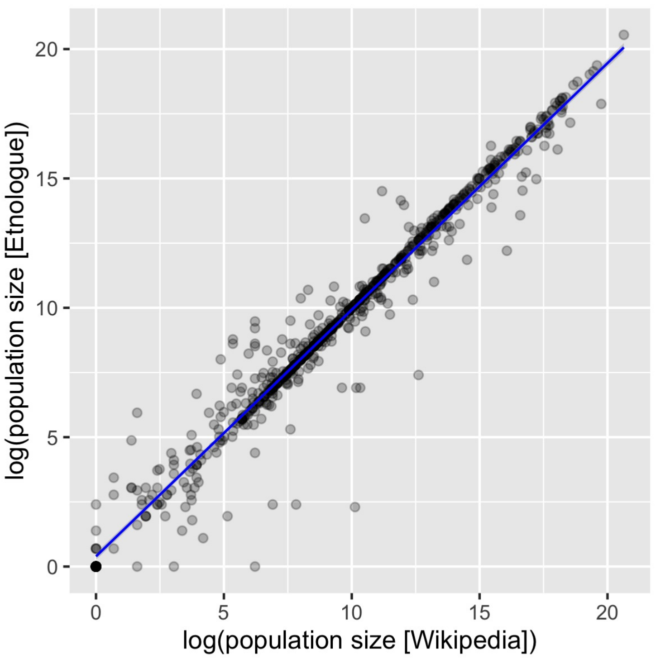

Figure S19. Relationship between log(population size) as given ultimately by the *Ethnologue* (horizontal axis) vs as given by *Wikidata*/*Wikipedia* (vertical axis). Figure generated using `R` version 4.2.3 (2023-03-15) and `ggplot2` (version 3.3.6).

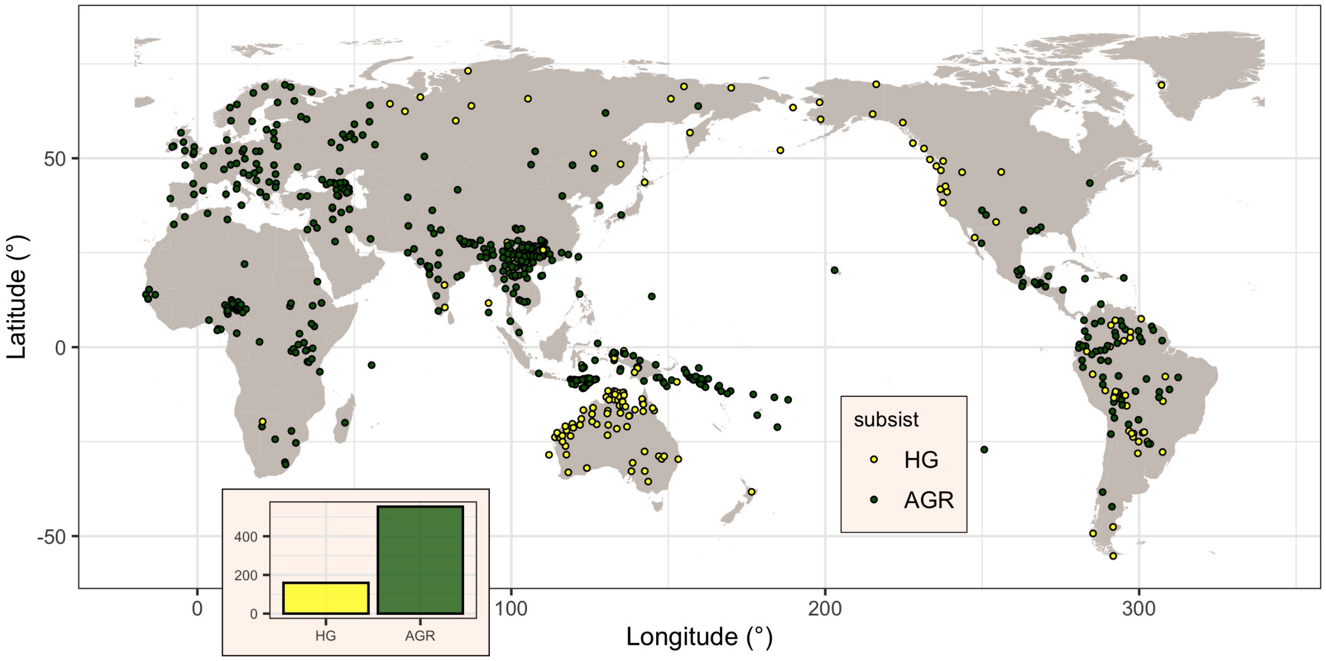

Figure S20. Map of the languages showing, for each, the type of subsistence practiced, as well the overall distribution of this variable across all languages (inset). Figure generated using `R` version 4.2.3 (2023-03-15) and packages `ggplot2` (version 3.3.6) and `maps` (version 3.4.0), using public domain data from the Natural Earth project as provided by the `R` package `maps`.

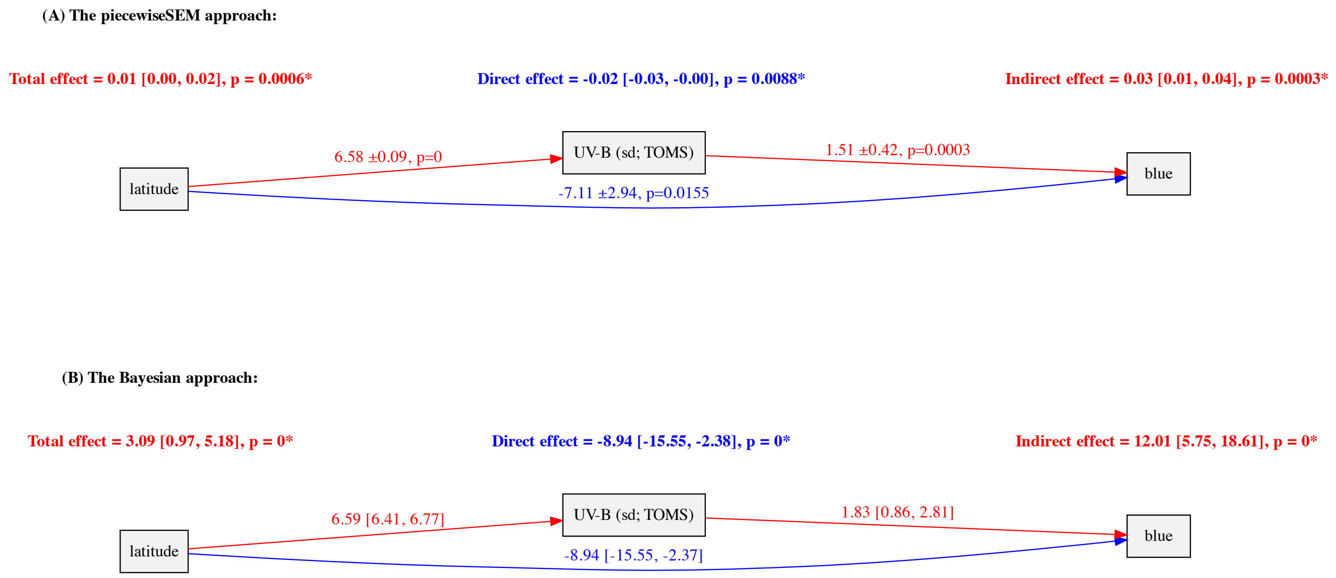

Figure S21. Mediation analysis of *latitude* $\longrightarrow$ *UV-B (mean)* $\longrightarrow$ *blue*, showing the total, direct and indirect effects, as well as the actual regression coefficients, using (top panel, **A**) frequentist piecewise Structural Equation Modeling (package `piecewiseSEM`) and (bottom panel, **B**) a Bayesian implementation (with `brms`). For **A**, the effects (standardized) are shown with their bootstrap 95%CI and p-value (and a star * if $p < 0.05$), and the regression coefficients with their standard error and p-value; the arrow, regression coefficient and effect color reflects their significance (light gray if not significant) and, if significant, the direction of the relationship (blue for negative and red for positive); if model comparison and the d-separation test suggest there is no direct effect, this is represented as a gray colored "[no direct effect]". For **B**, the effects (unstandardized) are shown with their 95%HDIs, p(ROPE) and a star * if p(ROPE) <0.05, and regression coefficients with their 95%HDIs; the arrow, regression coefficient and effect color reflects their "significance" (light gray if 0 is included in the 95%HDI or the effect's p(ROPE) > 0.05) and, if "significant", the direction of the relationship (blue for negative and red for positive). Please note that because the outcome (*blue*) is binary, the direct and indirect effects may be on different scales. Figure generated using `R` version 4.2.3 (2023-03-15) and package `DiagrammeR` (version 1.0.9).

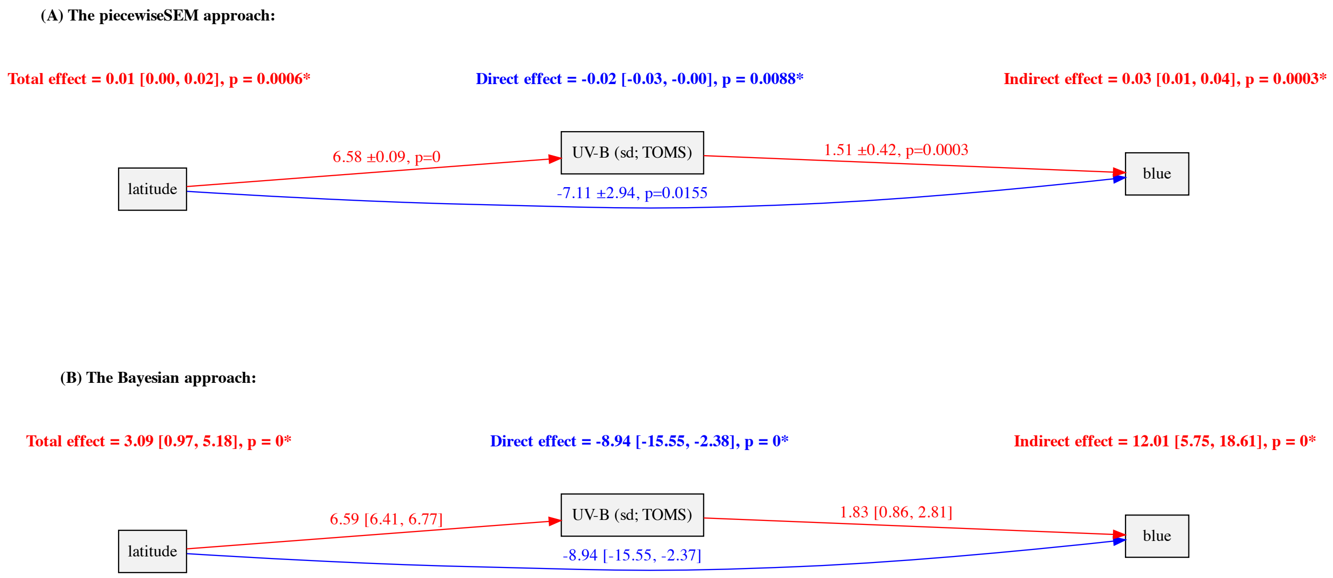

Figure S22. Mediation analysis of *latitude* ⟶ *UV-B (sd)* ⟶ *blue*; same conventions as in Figure S21.

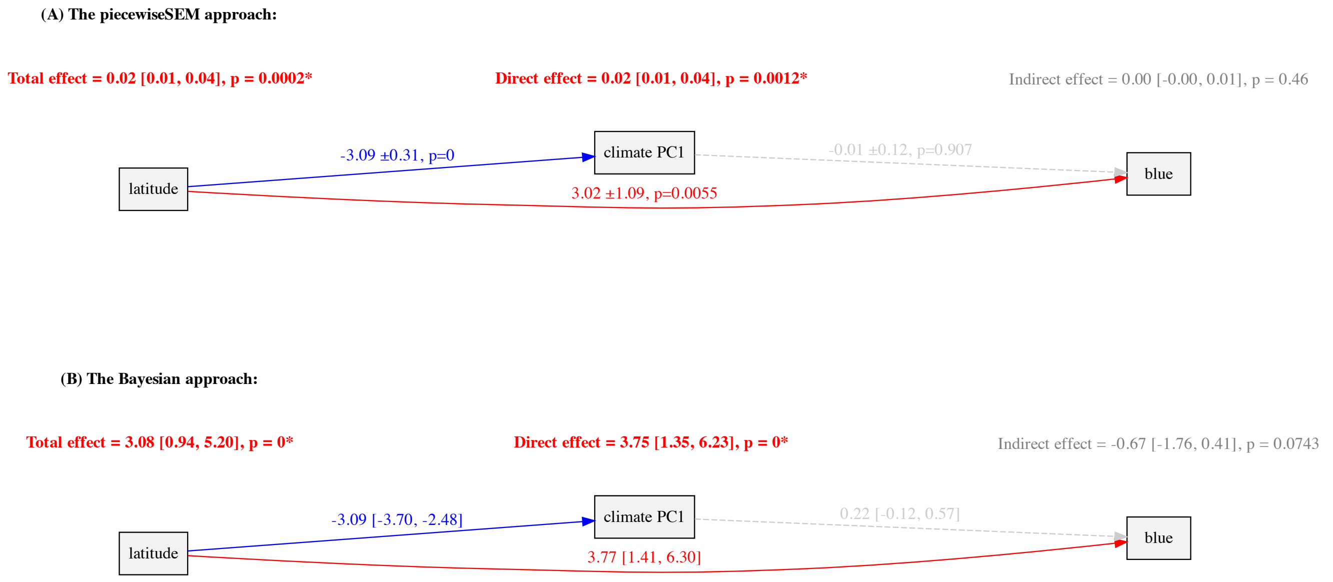

Figure S23. Mediation analysis of *latitude* ⟶ *climate PC1* ⟶ *blue*; same conventions as in Figure S21.

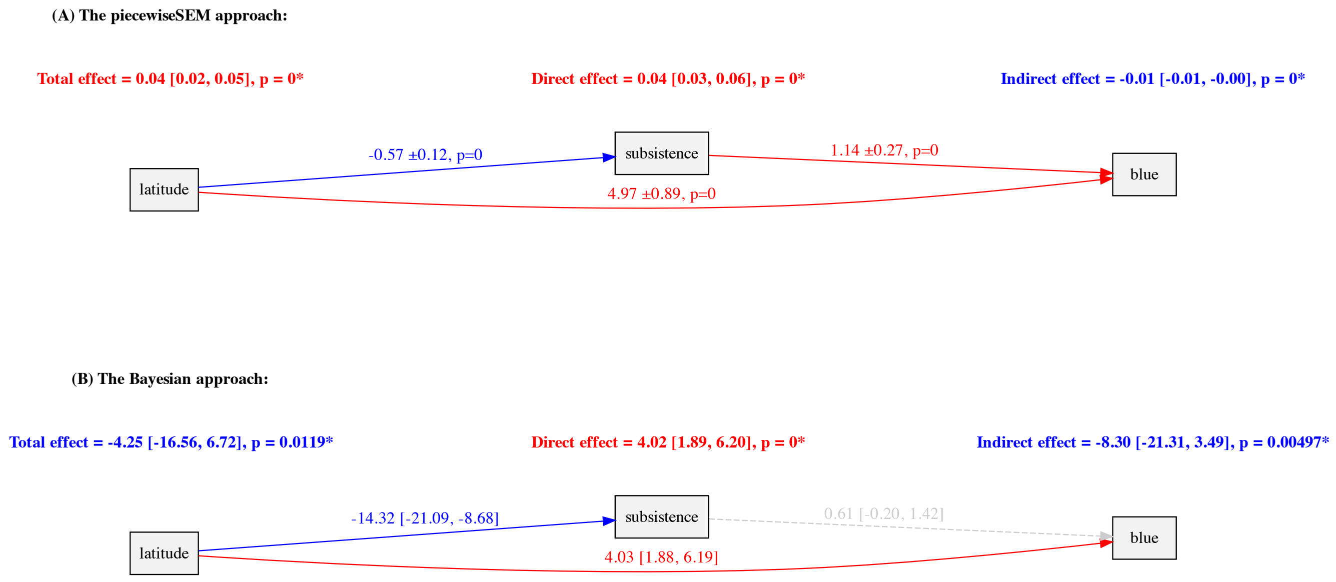

Figure S24. Mediation analysis of *latitude* ⟶ *subsistence* ⟶ *blue*; same conventions as in Figure S21.

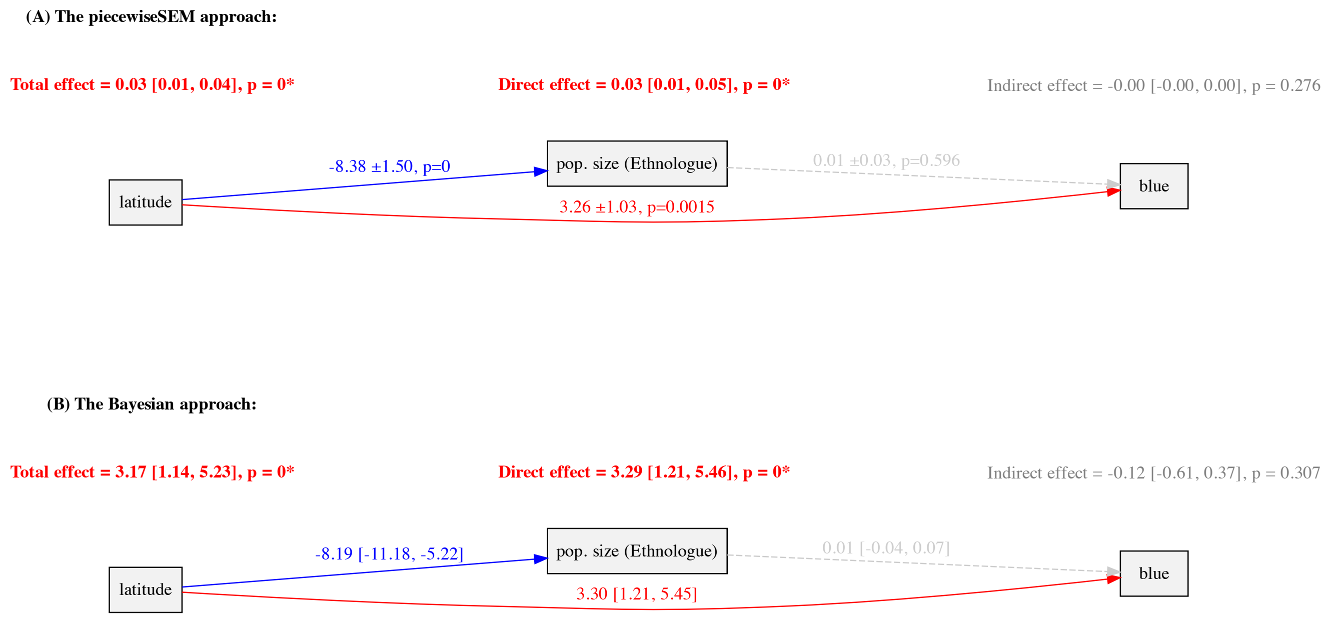

Figure S25. Mediation analysis of *latitude* ⟶ *population size* ⟶ *blue*; same conventions as in Figure S21.

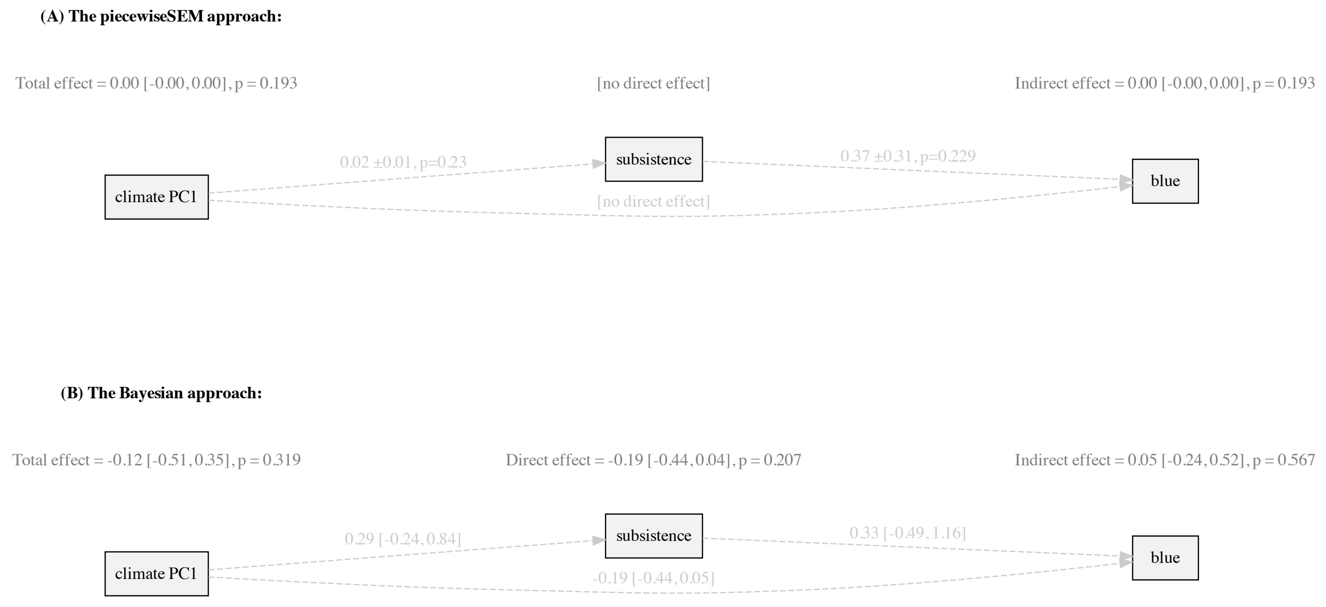

Figure S26. Mediation analysis of *climate PC1* ⟶ *subsistence* ⟶ *blue*; same conventions as in Figure S21.

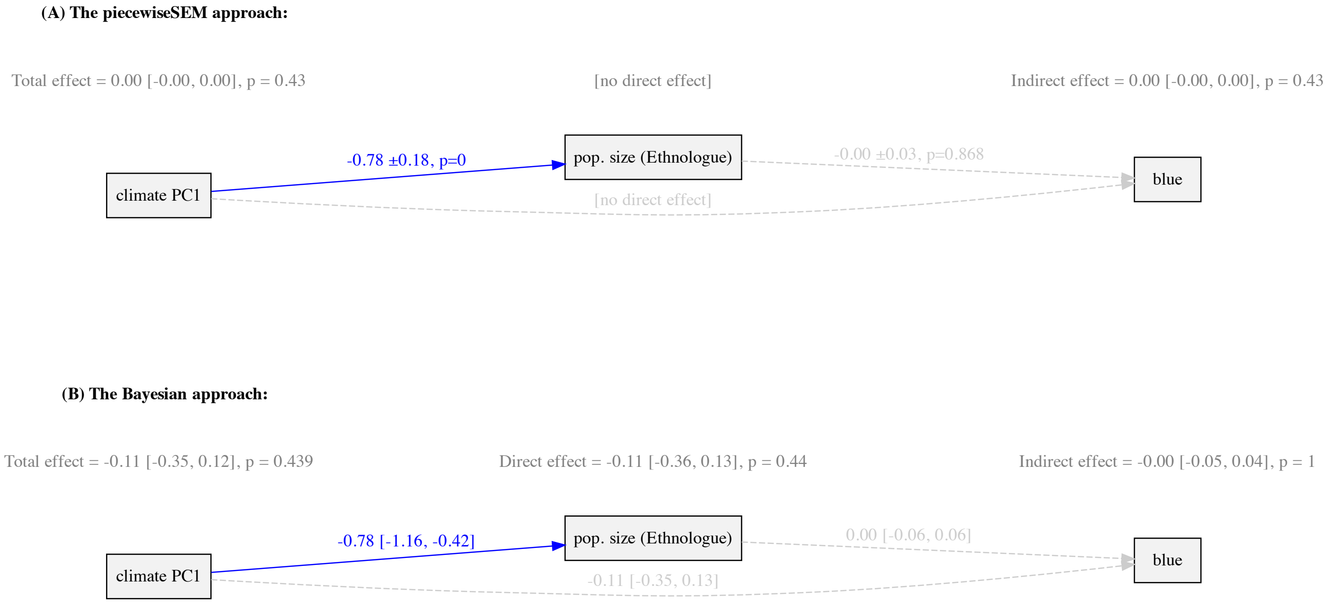

Figure S27. Mediation analysis of *climate PC1* ⟶ *population size* ⟶ *blue*; same conventions as in Figure S21.

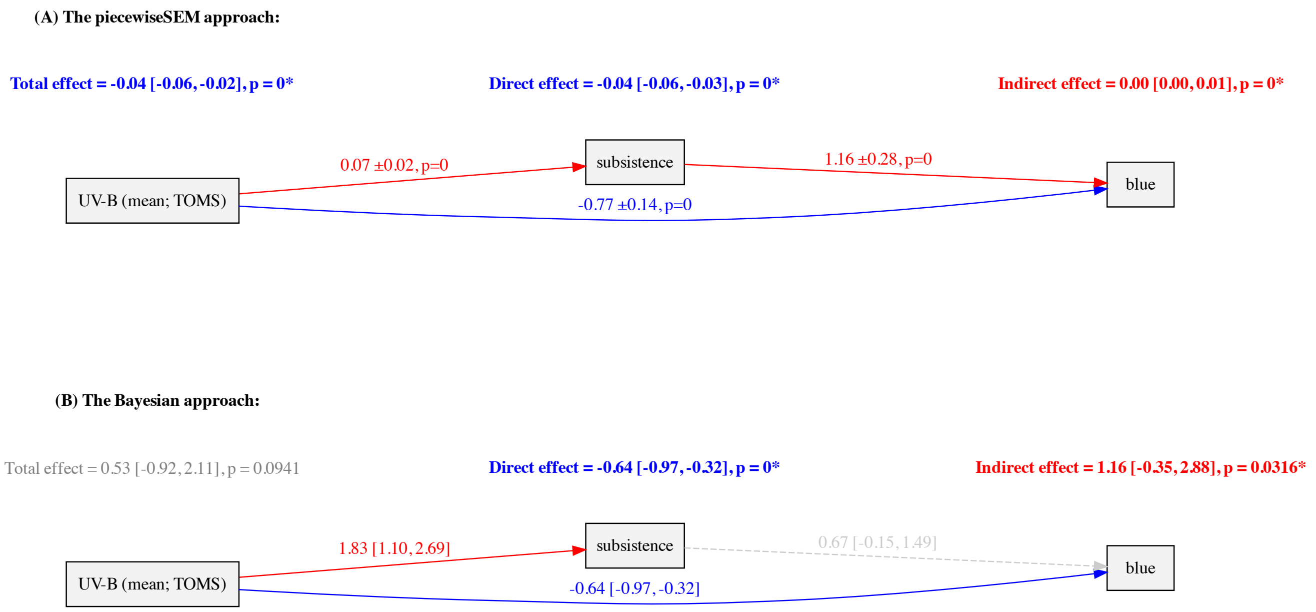

Figure S28. Mediation analysis of *UV-B (mean)* $\longrightarrow$ *subsistence* $\longrightarrow$ *blue*; same conventions as in Figure S21.

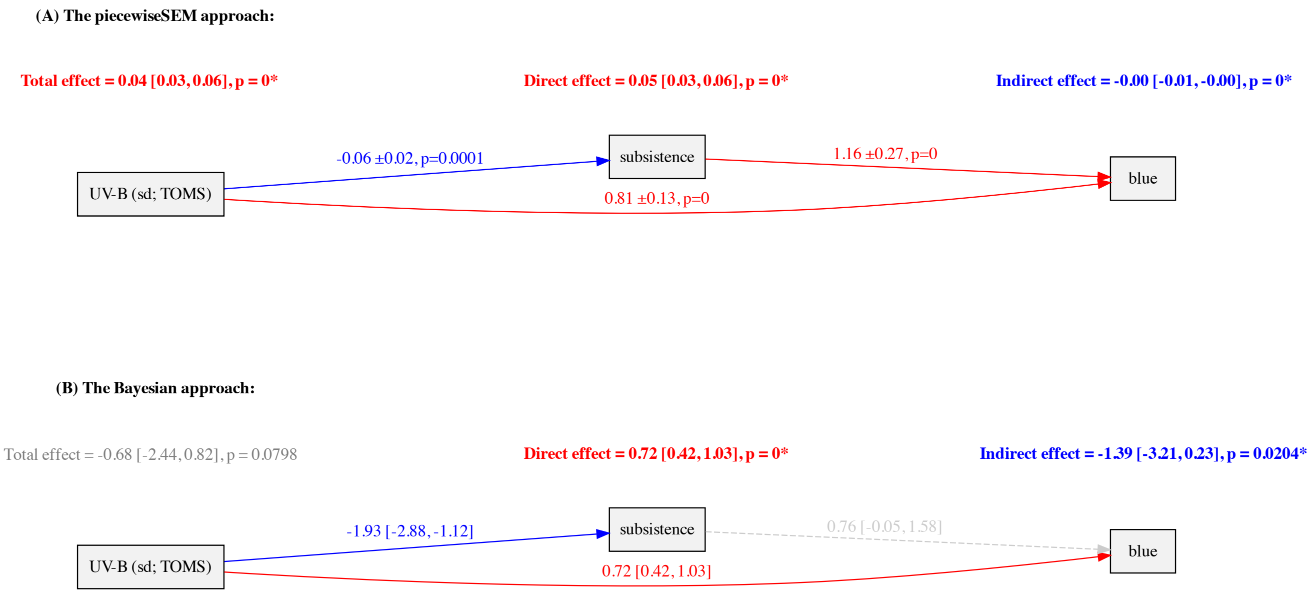

Figure S29. Mediation analysis of *UV-B (sd)* $\longrightarrow$ *subsistence* $\longrightarrow$ *blue*; same conventions as in Figure S21.

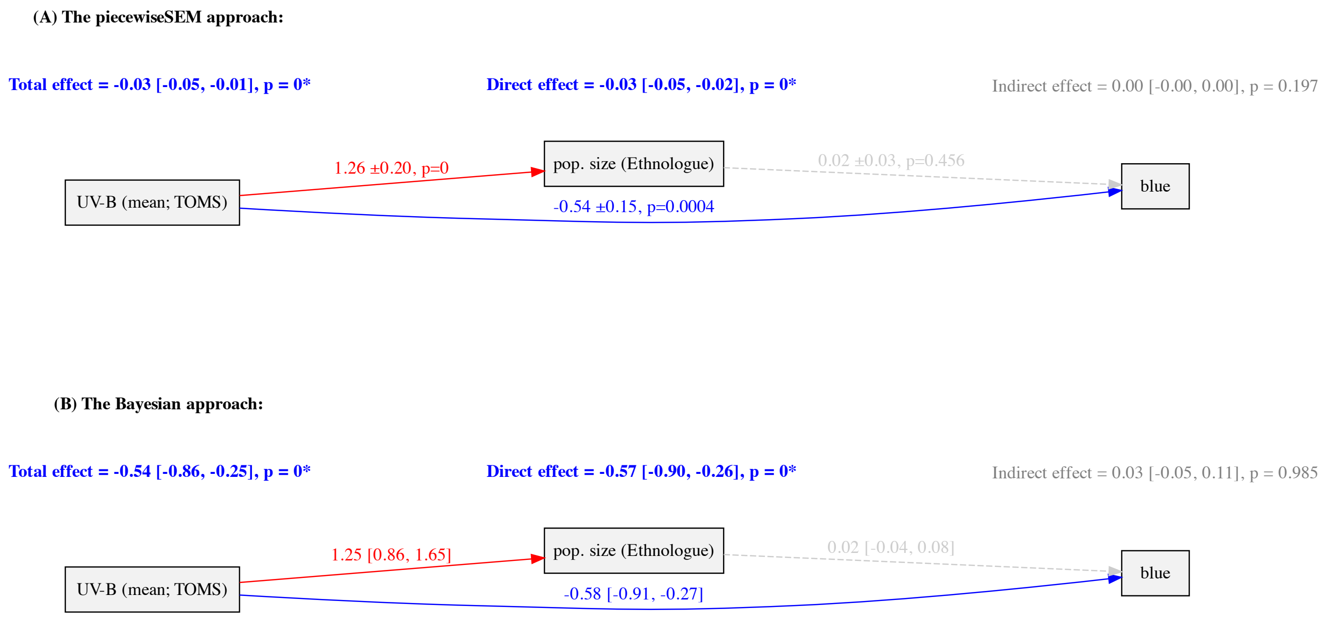

Figure S30. Mediation analysis of *UV-B (mean)* ⟶ *population size* ⟶ *blue*; same conventions as in Figure S21.

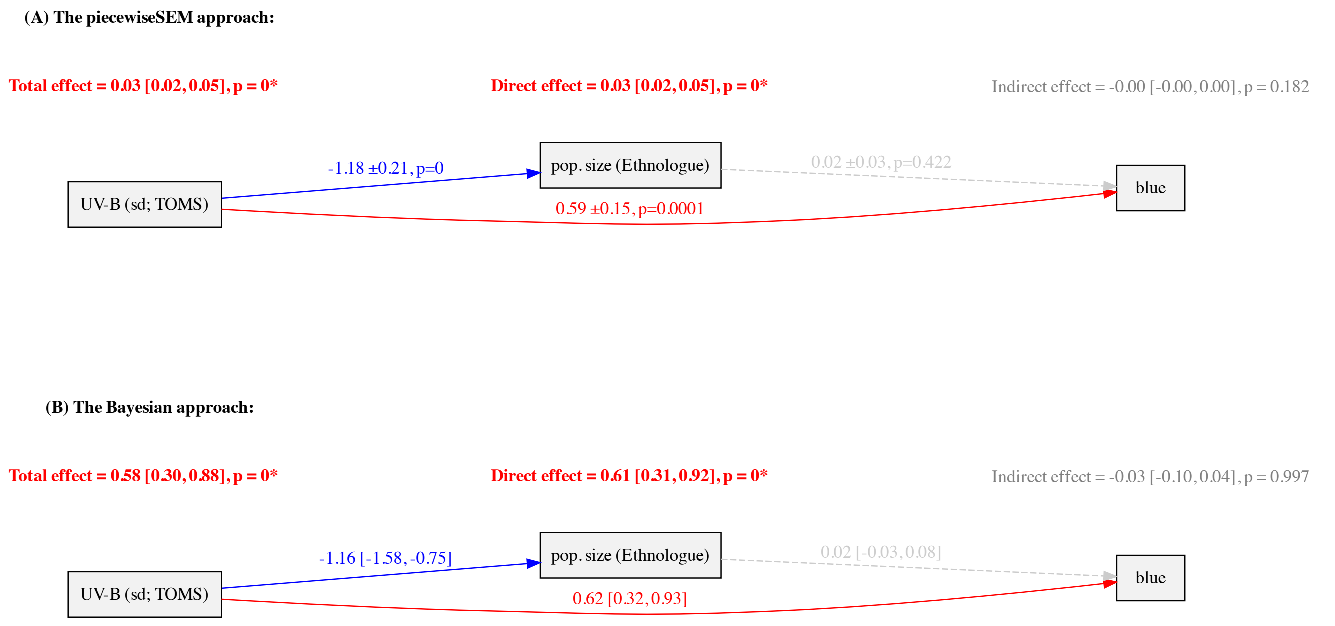

Figure S31. Mediation analysis of *UV-B (sd)* ⟶ *population size* ⟶ *blue*; same conventions as in Figure S21.

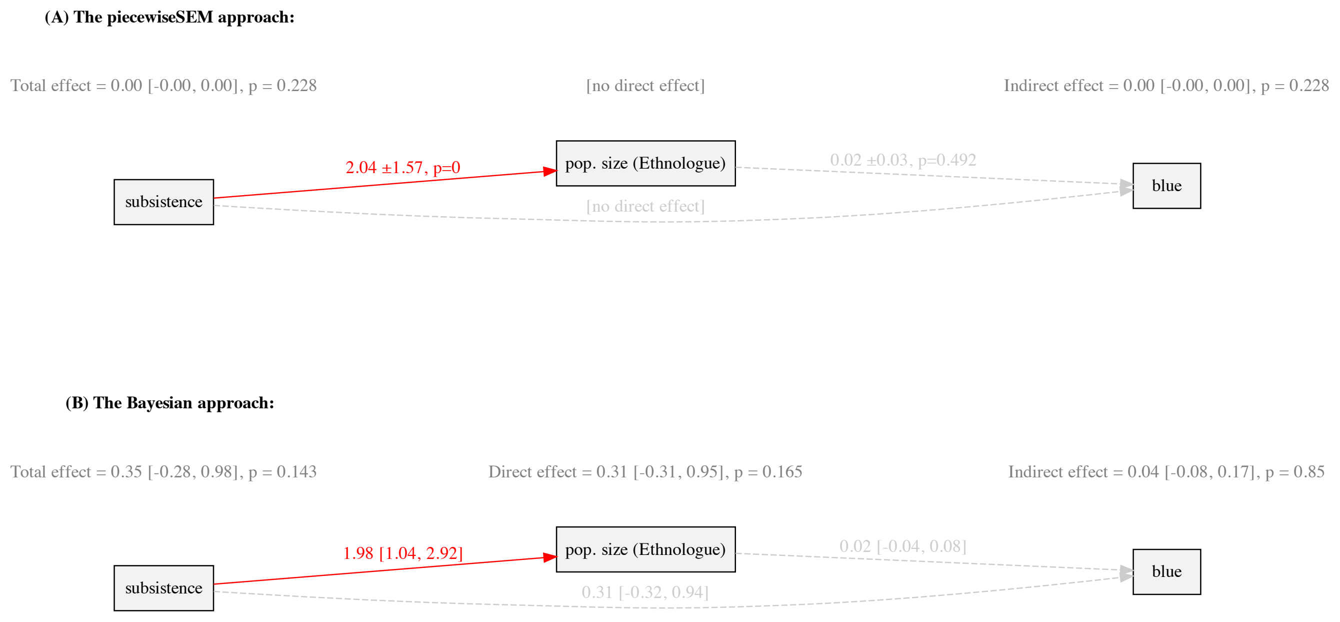

Figure S32. Mediation analysis of *subsistence* ⟶ *population size* ⟶ *blue*; same conventions as in Figure S21.

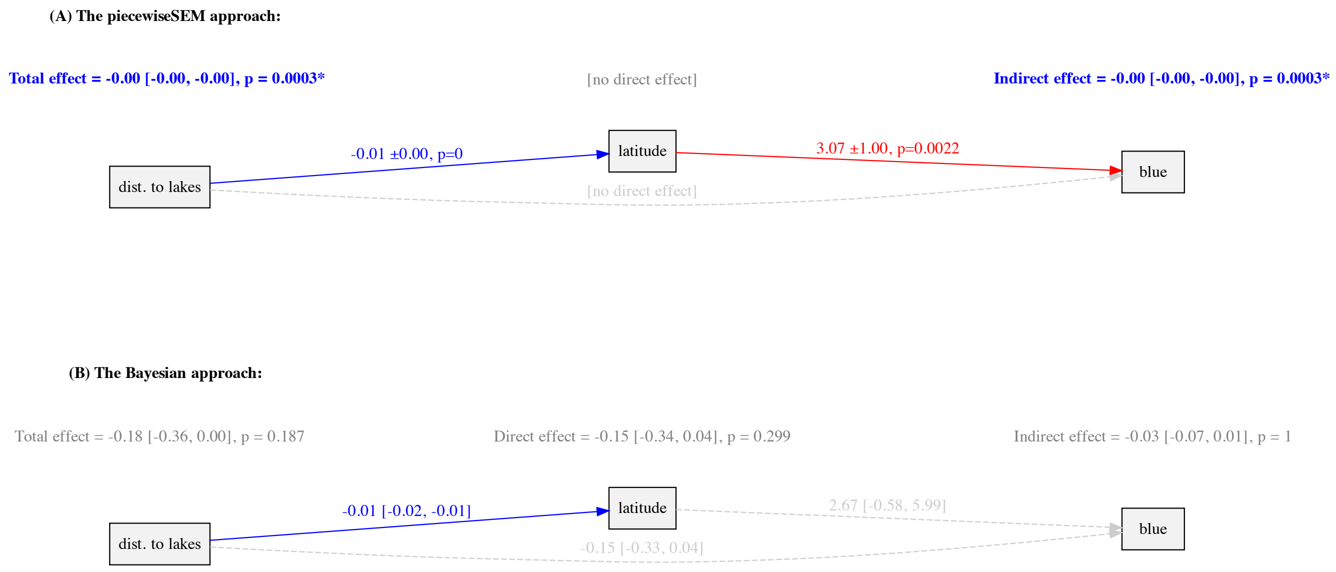

Figure S33. Mediation analysis of *dist2lakes* ⟶ *latitude* ⟶ *blue*; same conventions as in Figure S21.

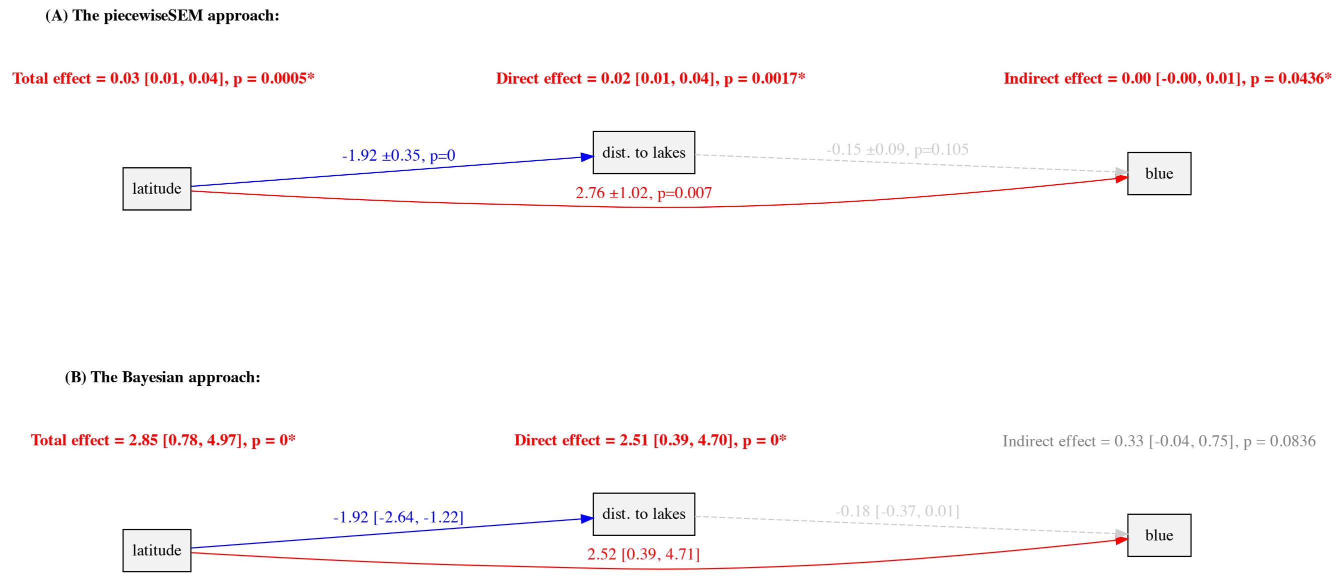

Figure S34. Mediation analysis of *latitude* ⟶ *dist2lakes* ⟶ *blue*; same conventions as in Figure S21.

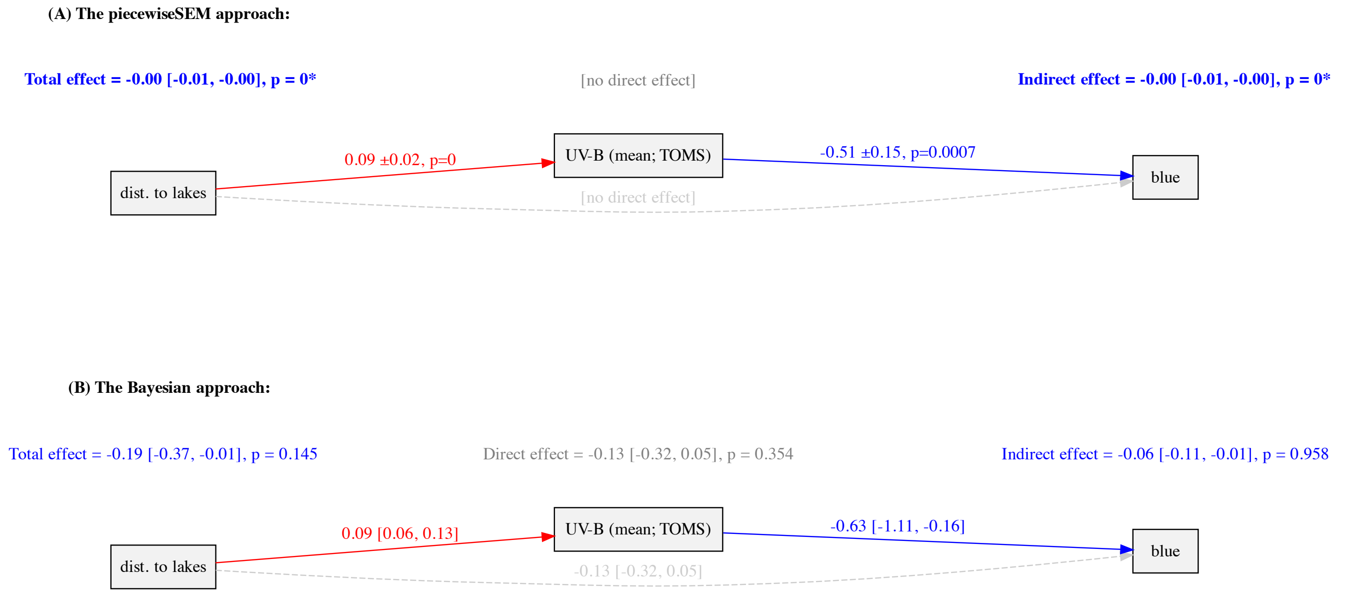

Figure S35. Mediation analysis of *dist2lakes* ⟶ *UV-B (mean)* ⟶ *blue*; same conventions as in Figure S21.

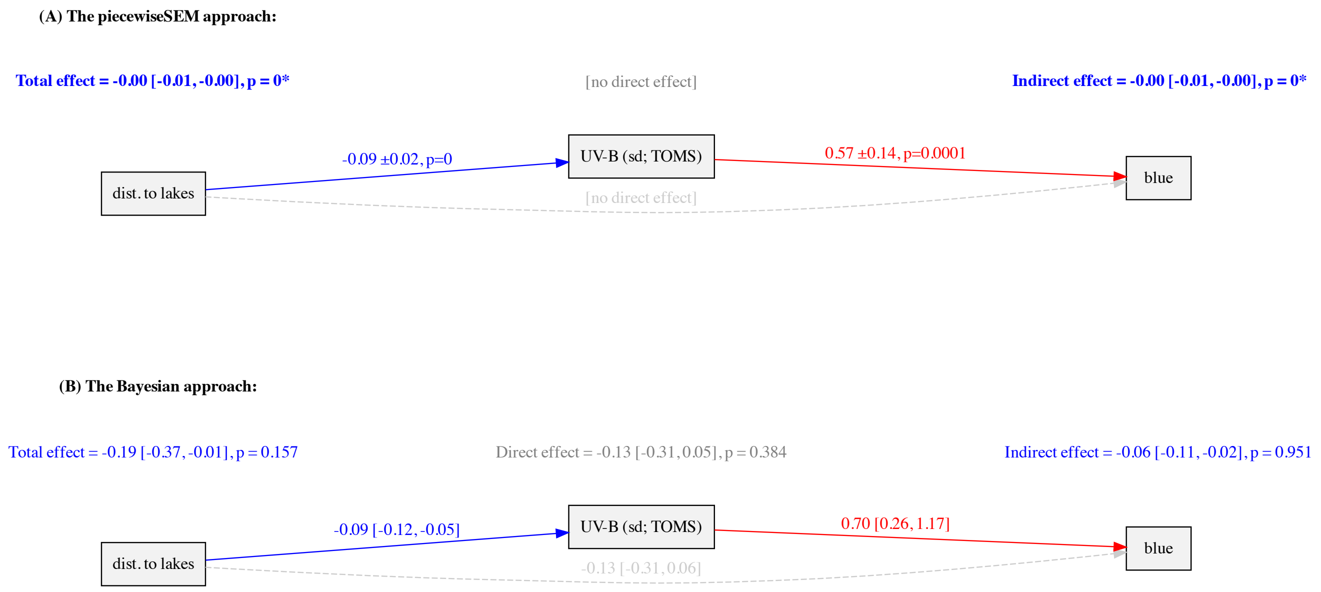

Figure S36. Mediation analysis of *dist2lakes* ⟶ *UV-B (sd)* ⟶ *blue*; same conventions as in Figure S21.

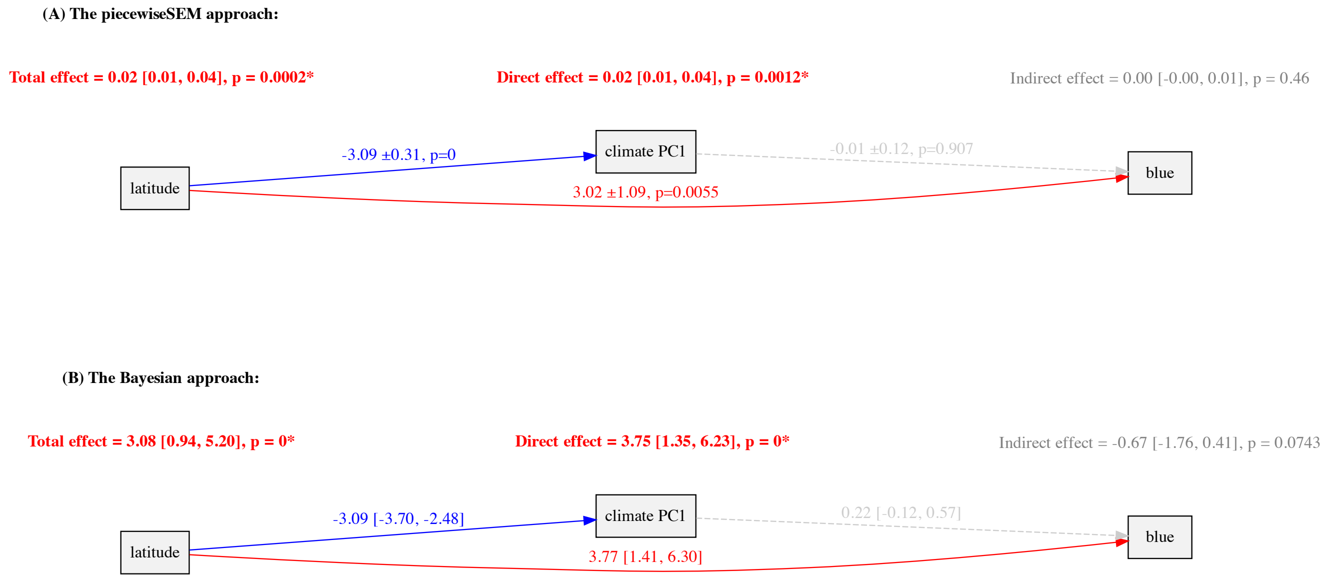

Figure S37. Mediation analysis of *dist2lakes* ⟶ *climate PC1* ⟶ *blue*; same conventions as in Figure S21.

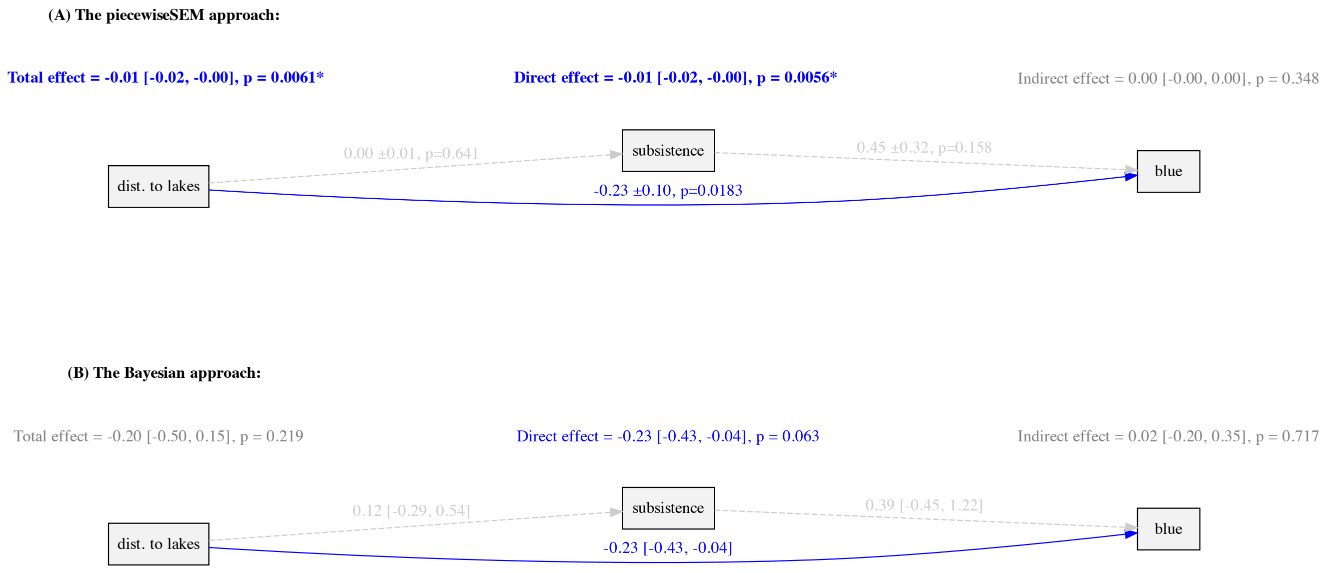

Figure S38. Mediation analysis of *dist2lakes* $\longrightarrow$ *subsistence* $\longrightarrow$ *blue*; same conventions as in Figure S21.

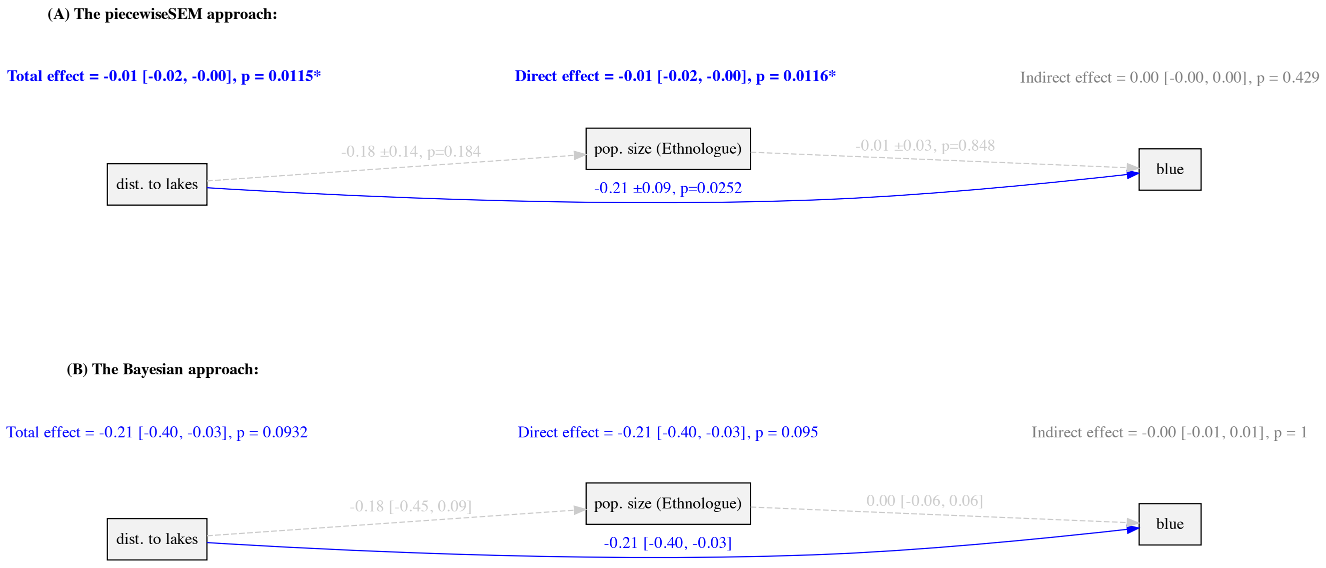

Figure S39. Mediation analysis of *dist2lakes* $\longrightarrow$ *population size* $\longrightarrow$ *blue*; same conventions as in Figure S21.

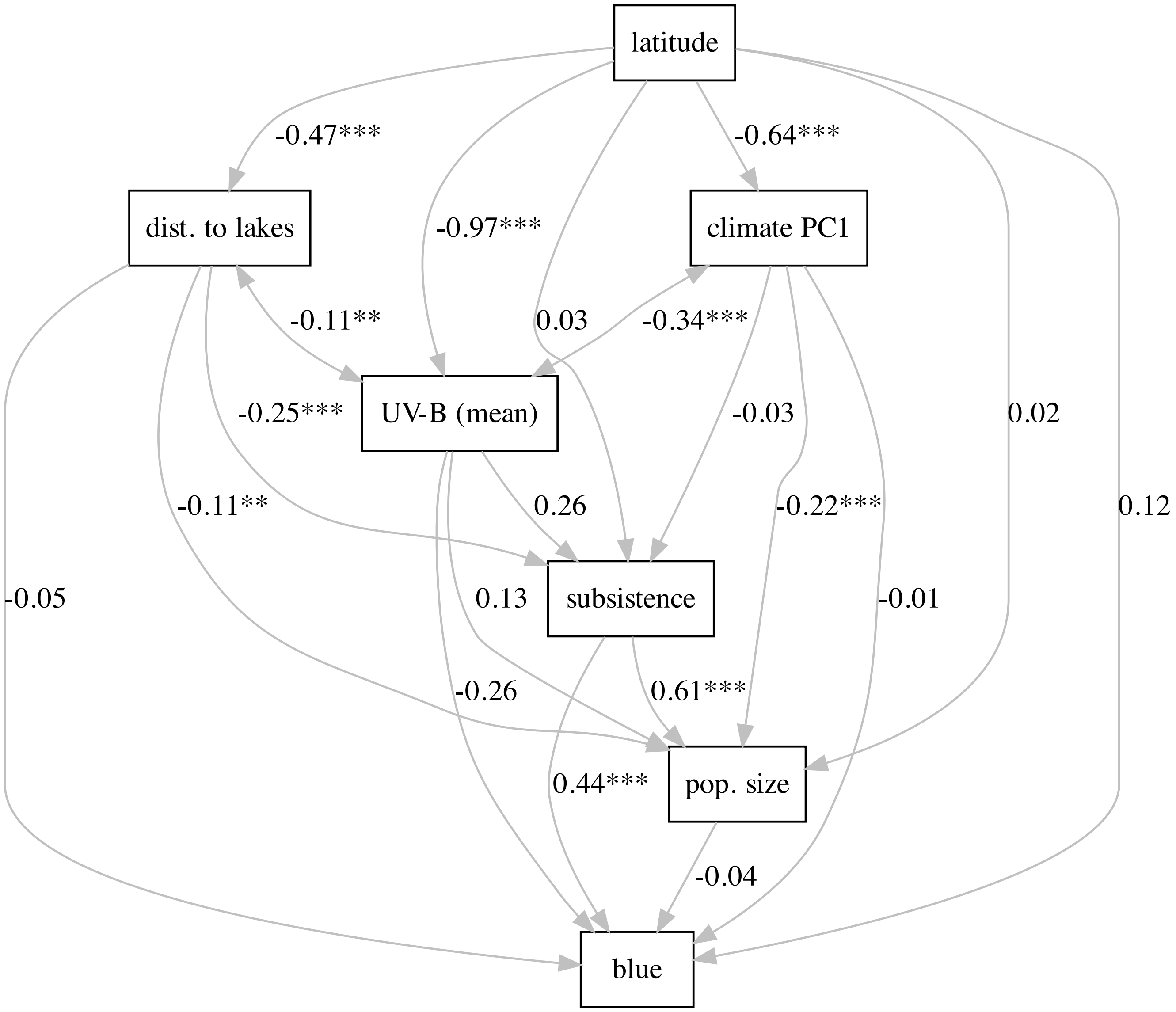

Figure S40. The "full" path model using *UV-B mean*. The labels on the path are the path coefficients; stars represent significance. Figure generated using `R` version 4.2.3 (2023-03-15) and package `lavaanPlot` (version 0.6.2).

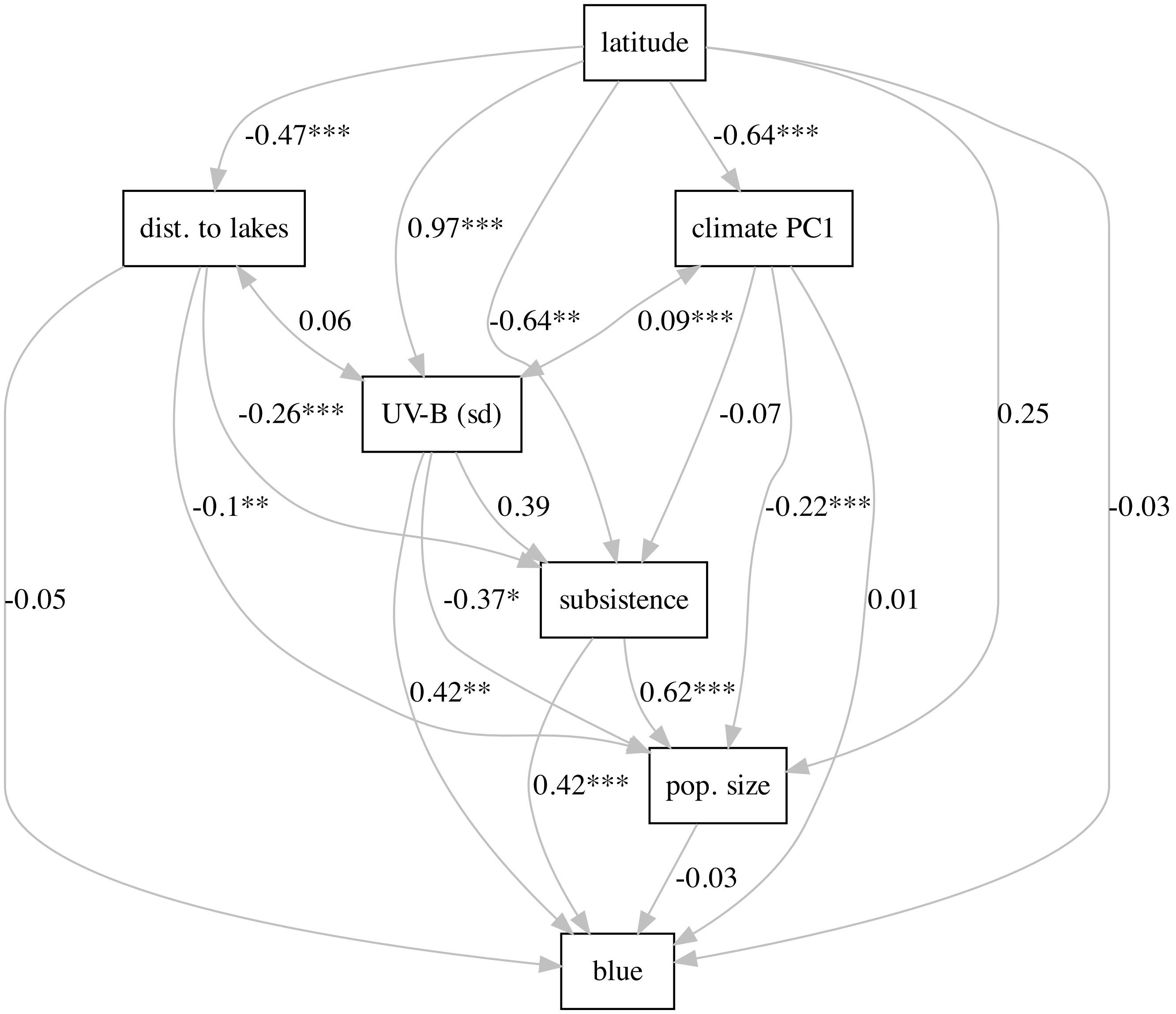

Figure S41. The "full" path model using *UV-B sd*. The labels on the path are the path coefficients; stars represent significance. Figure generated using `R` version 4.2.3 (2023-03-15) and package `lavaanPlot` (version 0.6.2).

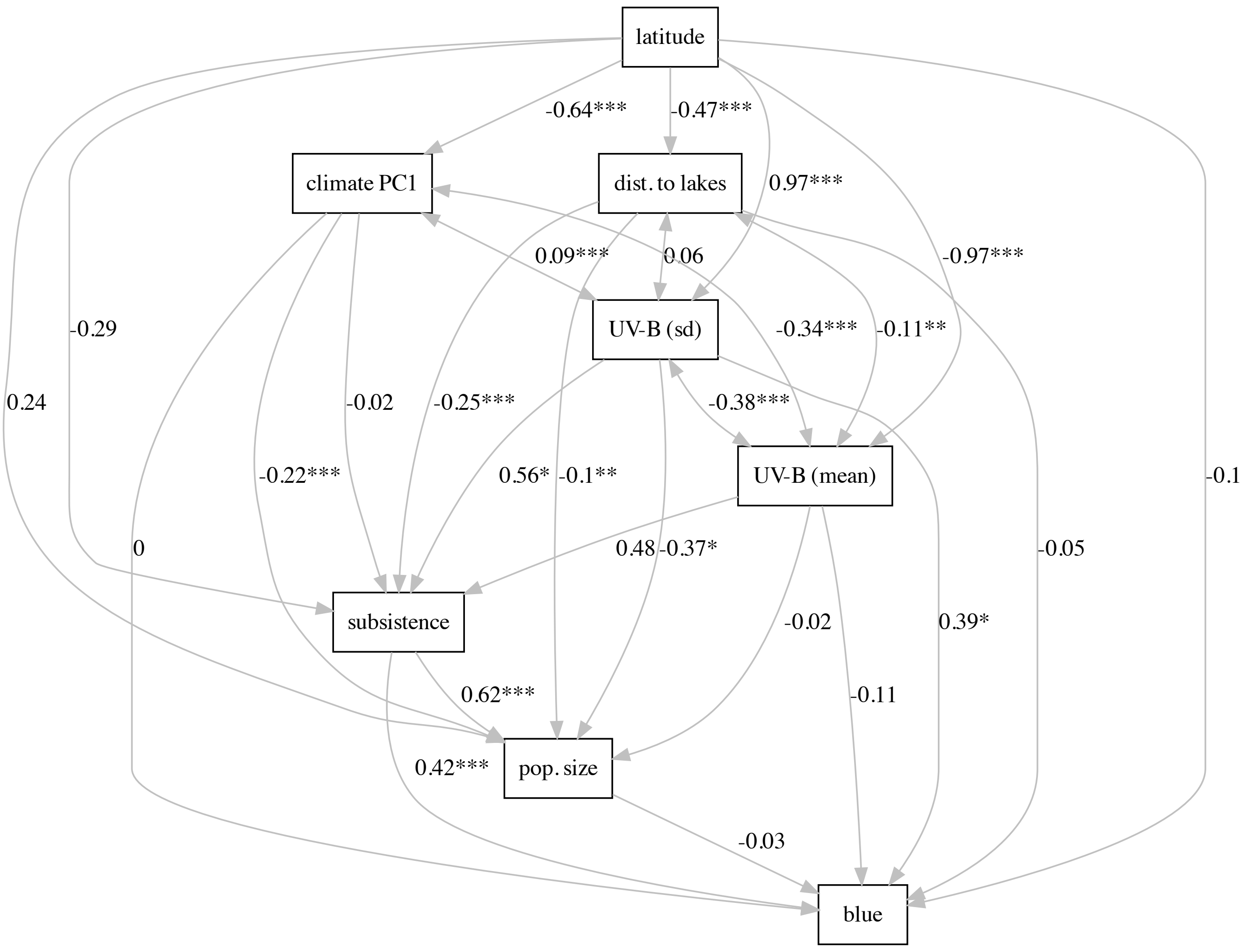

Figure S42. The "full" path model using both *UV-B mean* and *UV-B sd*. The labels on the path are the path coefficients; stars represent significance. Figure generated using `R` version 4.2.3 (2023-03-15) and package `lavaanPlot` (version 0.6.2).

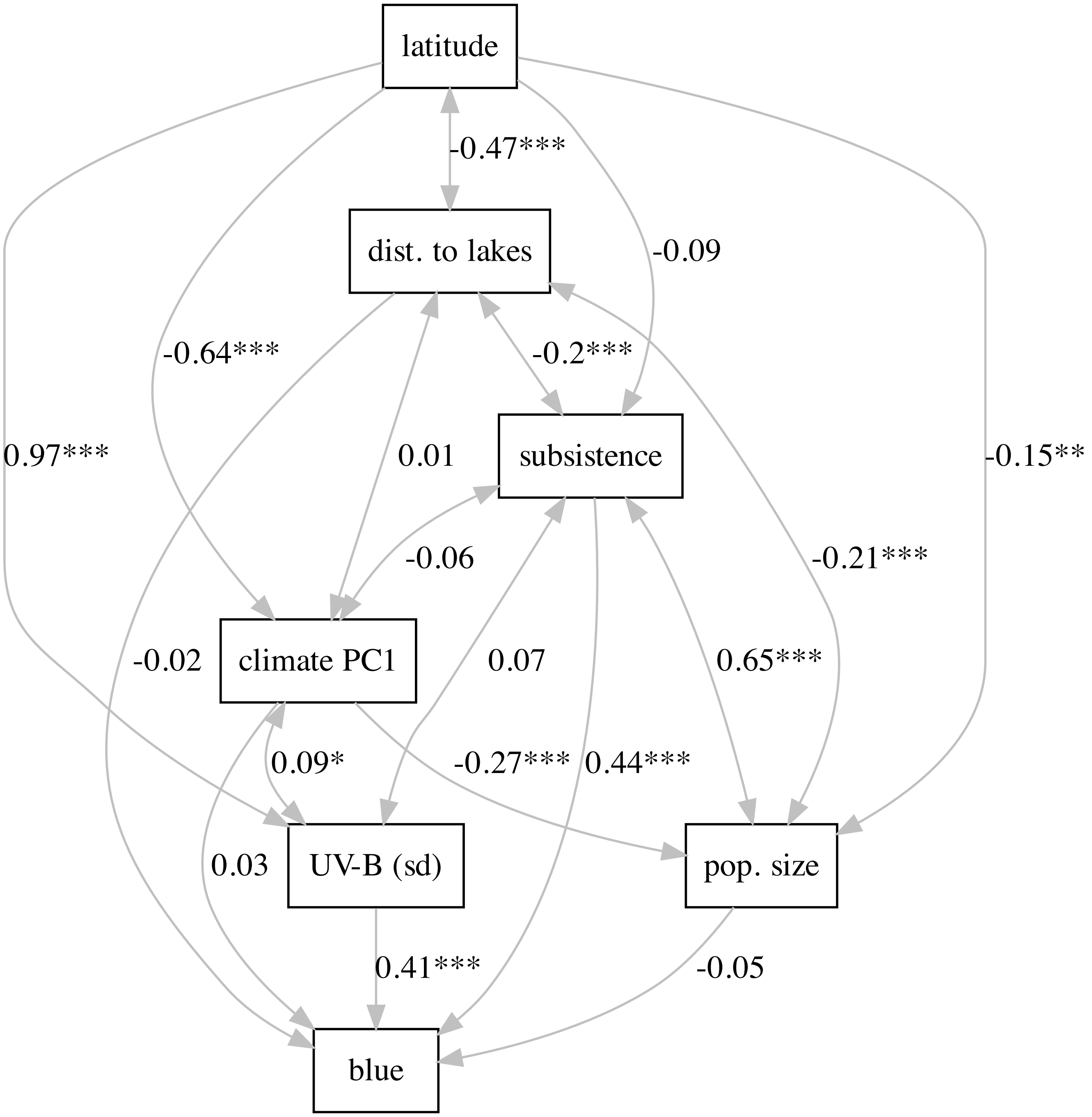

Figure S43. The “relaxed” path model using *UV-B sd*. The labels on the path are the path coefficients; stars represent significance. Figure generated using `R` version 4.2.3 (2023-03-15) and package `lavaanPlot` (version 0.6.2).

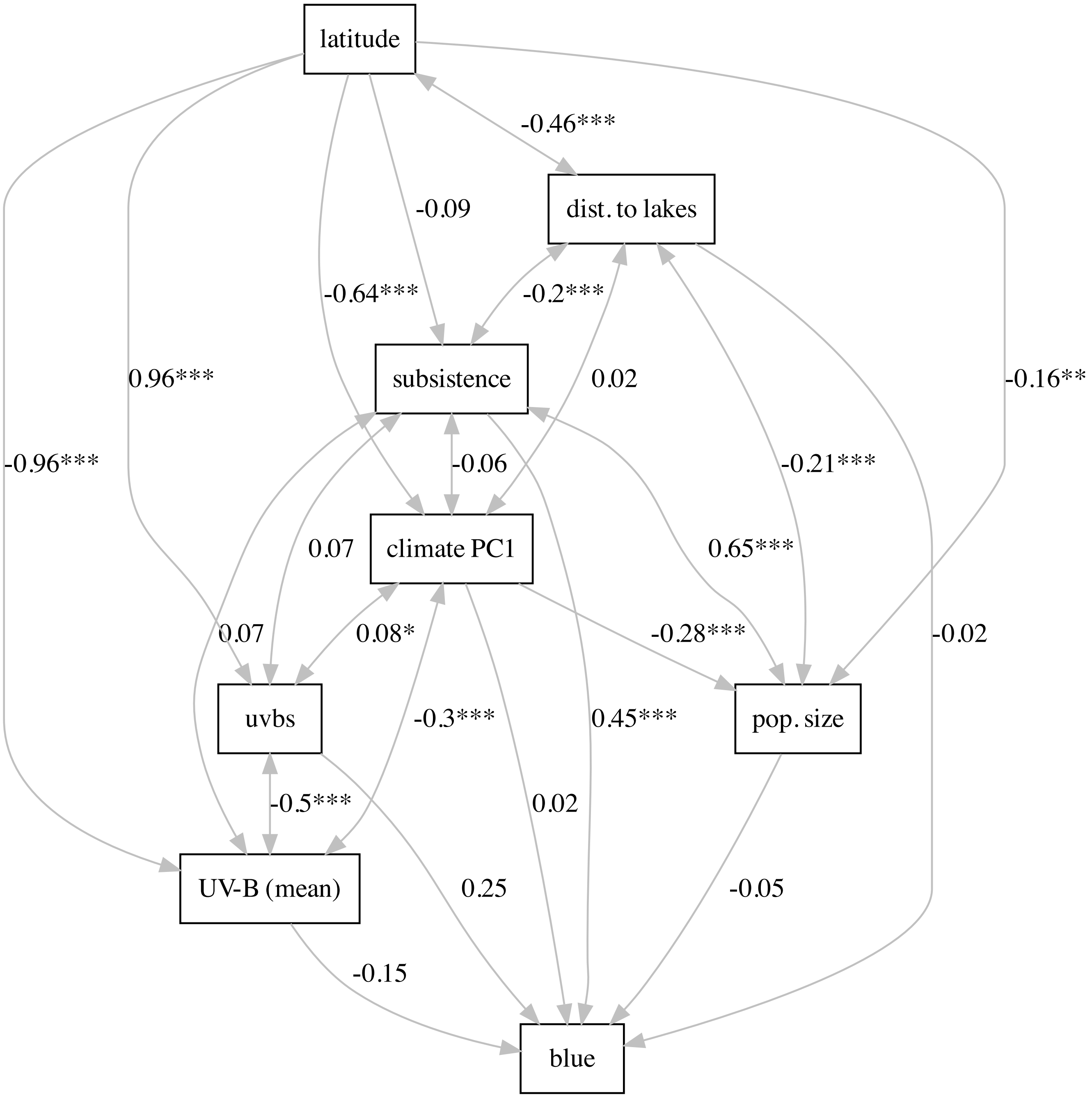

Figure S44. The "relaxed" path model using both *UV-B mean* and *UV-B sd*. The labels on the path are the path coefficients; stars represent significance. Figure generated using `R` version 4.2.3 (2023-03-15) and package `lavaanPlot` (version 0.6.2).

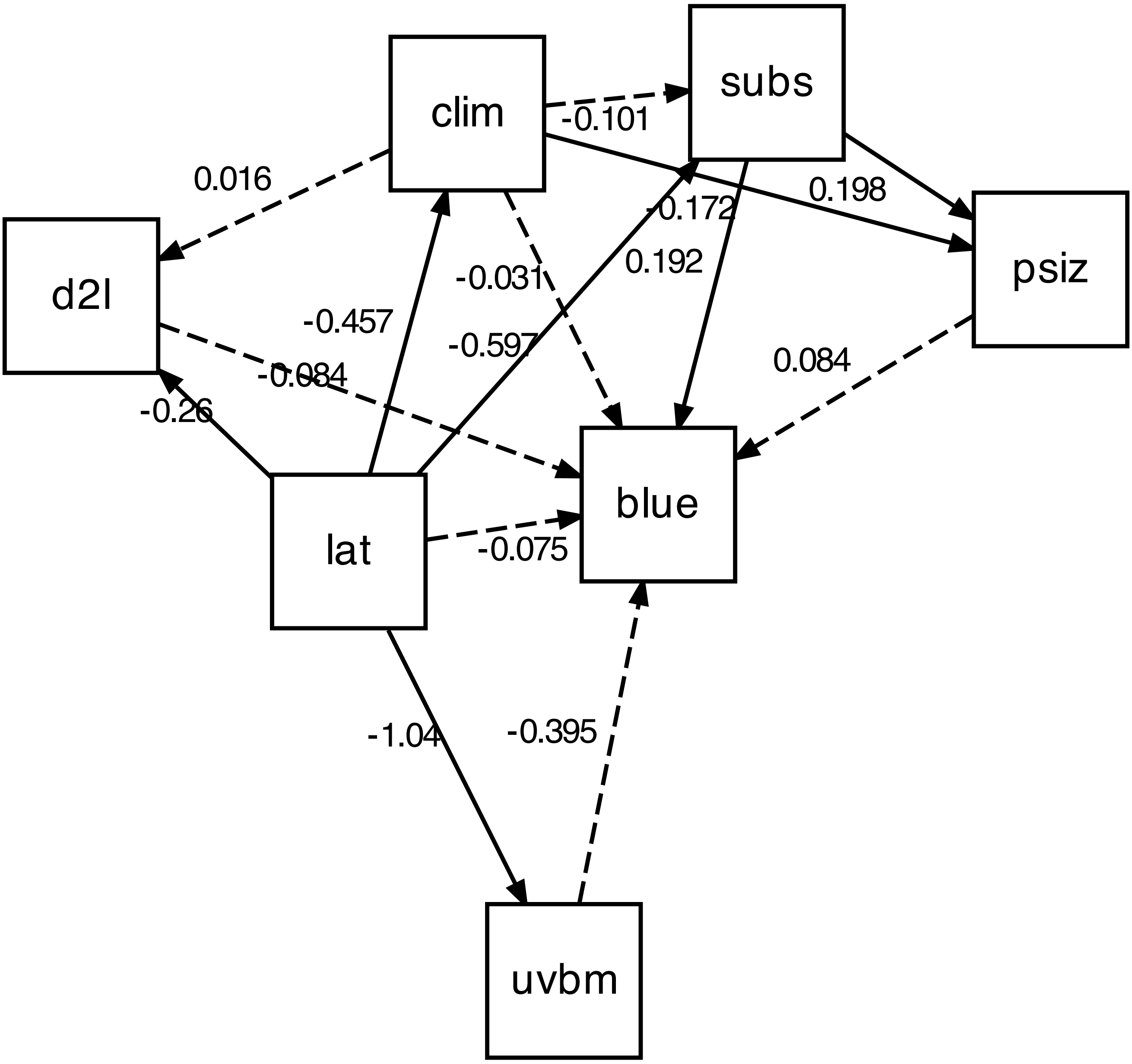

Figure S45. The piecewise model tested with `piecewieseSEM` for mean(UV-B) showing the regression coefficients; the edges are solid if the regression coefficient is significantly different from 0. Figure generated using `R` version 4.2.3 (2023-03-15) and package `lavaanPlot` (version 0.6.2).

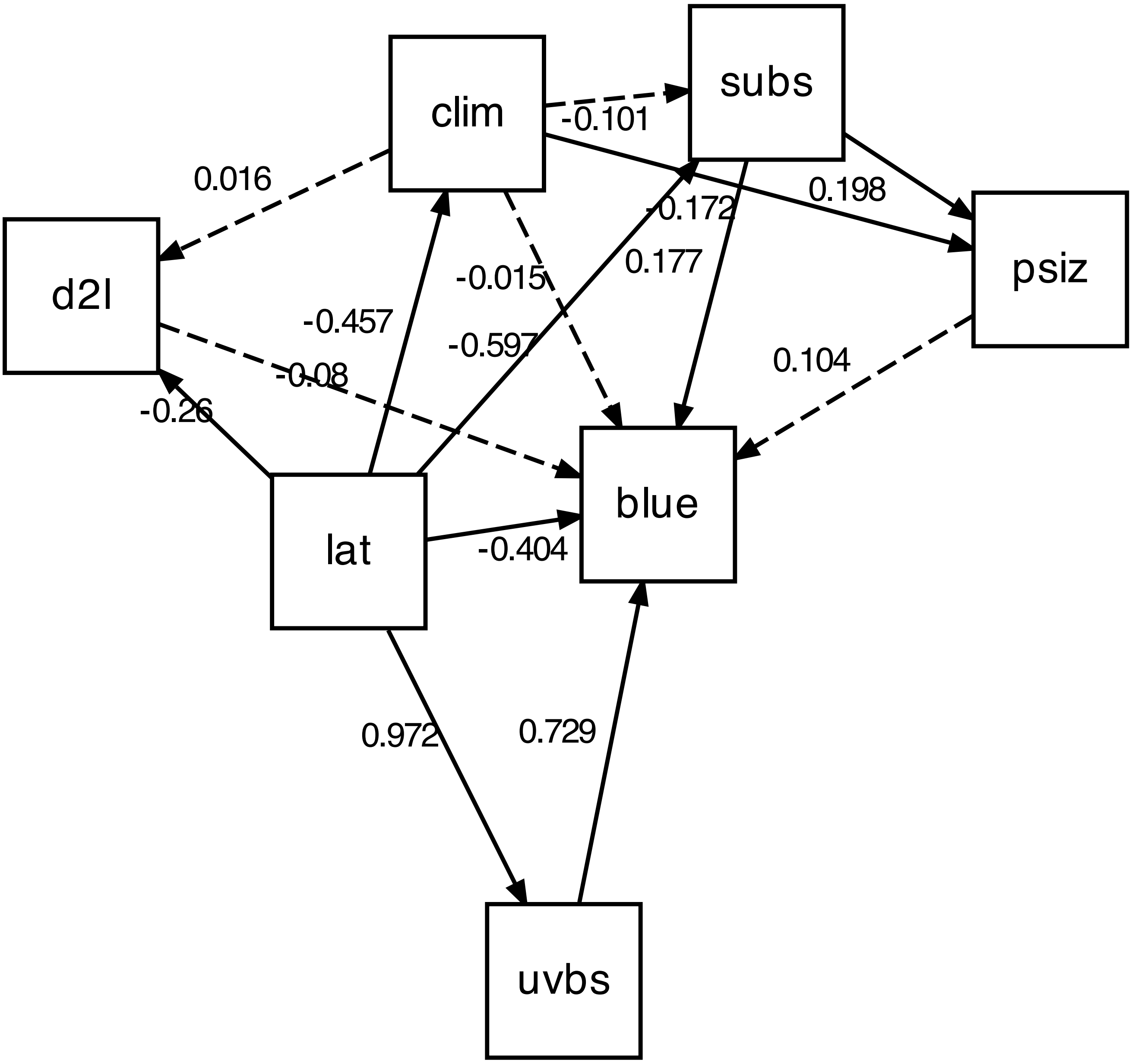

Figure S46. The piecewise model tested with `piecewieseSEM` for sd(UV-B) showing the regression coefficients; the edges are solid if the regression coefficient is significantly different from 0. Figure generated using `R` version 4.2.3 (2023-03-15) and package `lavaanPlot` (version 0.6.2).

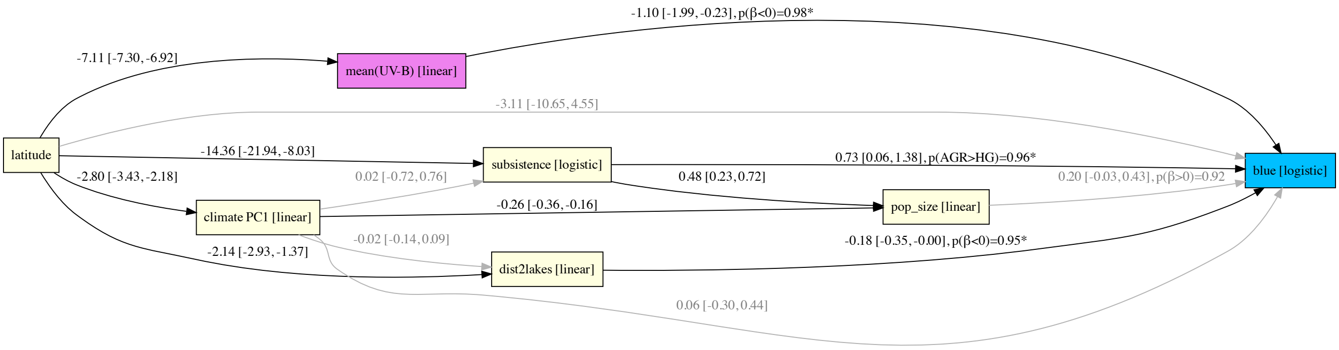

Figure S47. The piecewise `brms` for mean(UV-B) showing the regression coefficients with their 95%HDIs, if 0 is contained within the 95%HDI (by color) and, for those with *a priori* hypotheses, the posterior probability of the hypothesis. Figure generated using `R` version 4.2.3 (2023-03-15) and package `lavaanPlot` (version 0.6.2).

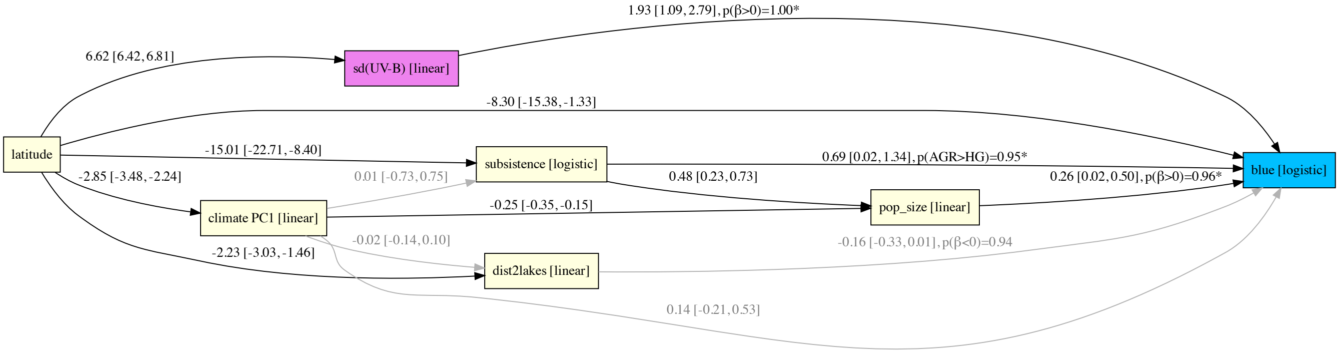

Figure S48. The piecewise `brms` for sd(UV-B) showing the regression coefficients with their 95%HDIs, if 0 is contained within the 95%HDI (by color) and, for those with *a priori* hypotheses, the posterior probability of the hypothesis. Figure generated using `R` version 4.2.3 (2023-03-15) and package `lavaanPlot` (version 0.6.2).

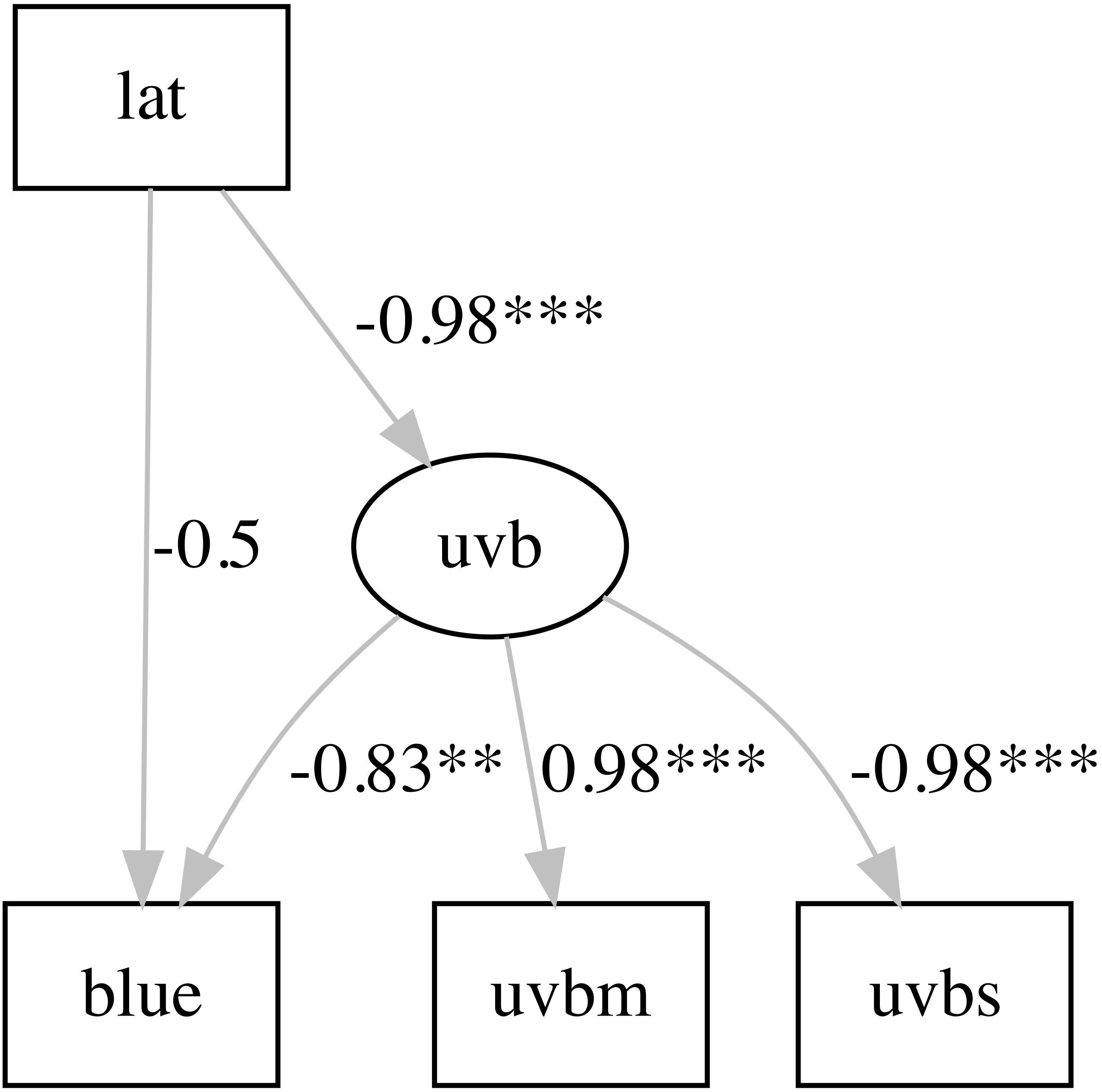

Figure S49. SEM model of the main hypothesis with a latent UV-B incidence with standardized coefficients, showing all path estimates with significance. Single-headed arrows represent regressions, while double-headed arrows represent covariance. Figure generated using `R` version 4.2.3 (2023-03-15) and package `lavaanPlot` (version 0.6.2).

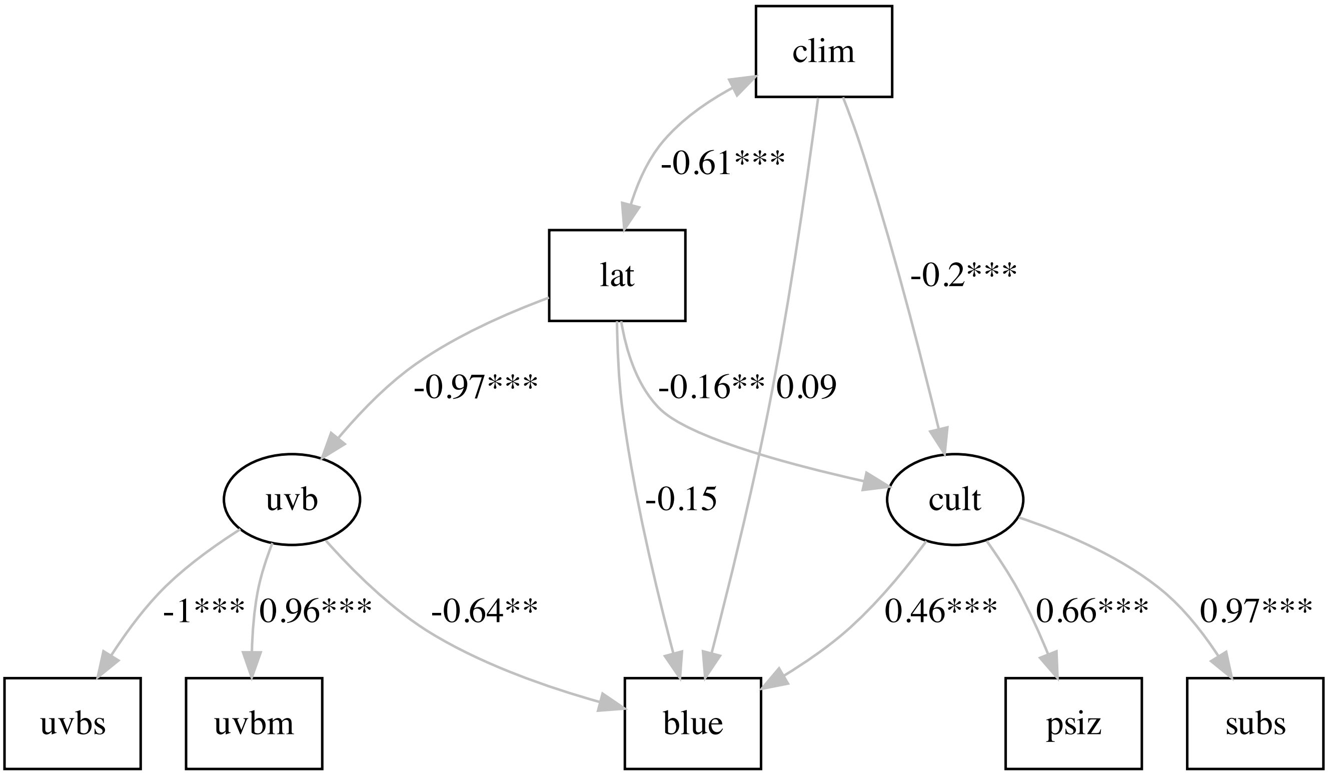

Figure S50. ... adding climate and "cultural complexity" with standardized coefficients, showing all path estimates with significance. Single-headed arrows represent regressions, while double-headed arrows represent covariance. Figure generated using `R` version 4.2.3 (2023-03-15) and package `lavaanPlot` (version 0.6.2).

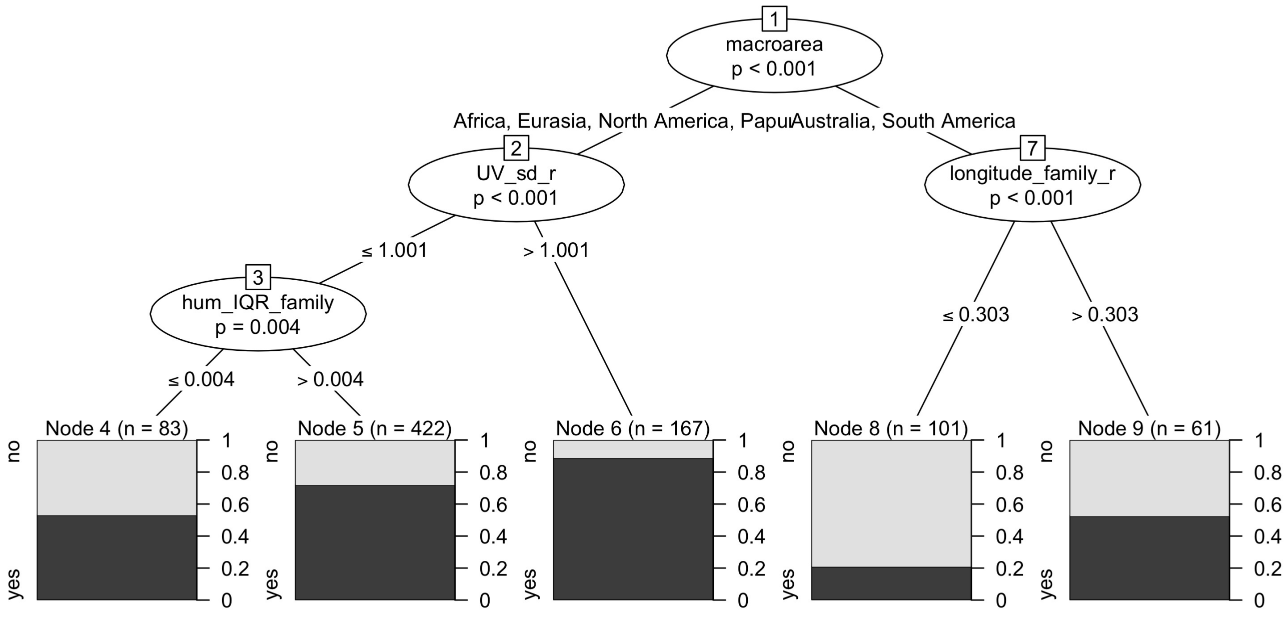

Figure S51. Conditional inference tree for predicting *blue* using all the potential predictors on the full dataset. Please note that some predictors are transformed (the "_r" suffix), and that, for interpretability, a maximum of 4 predictors and a maximum depth of 3 were allowed. Figure generated using `R` version 4.2.3 (2023-03-15) and package `partykit` (version 1.2.16).

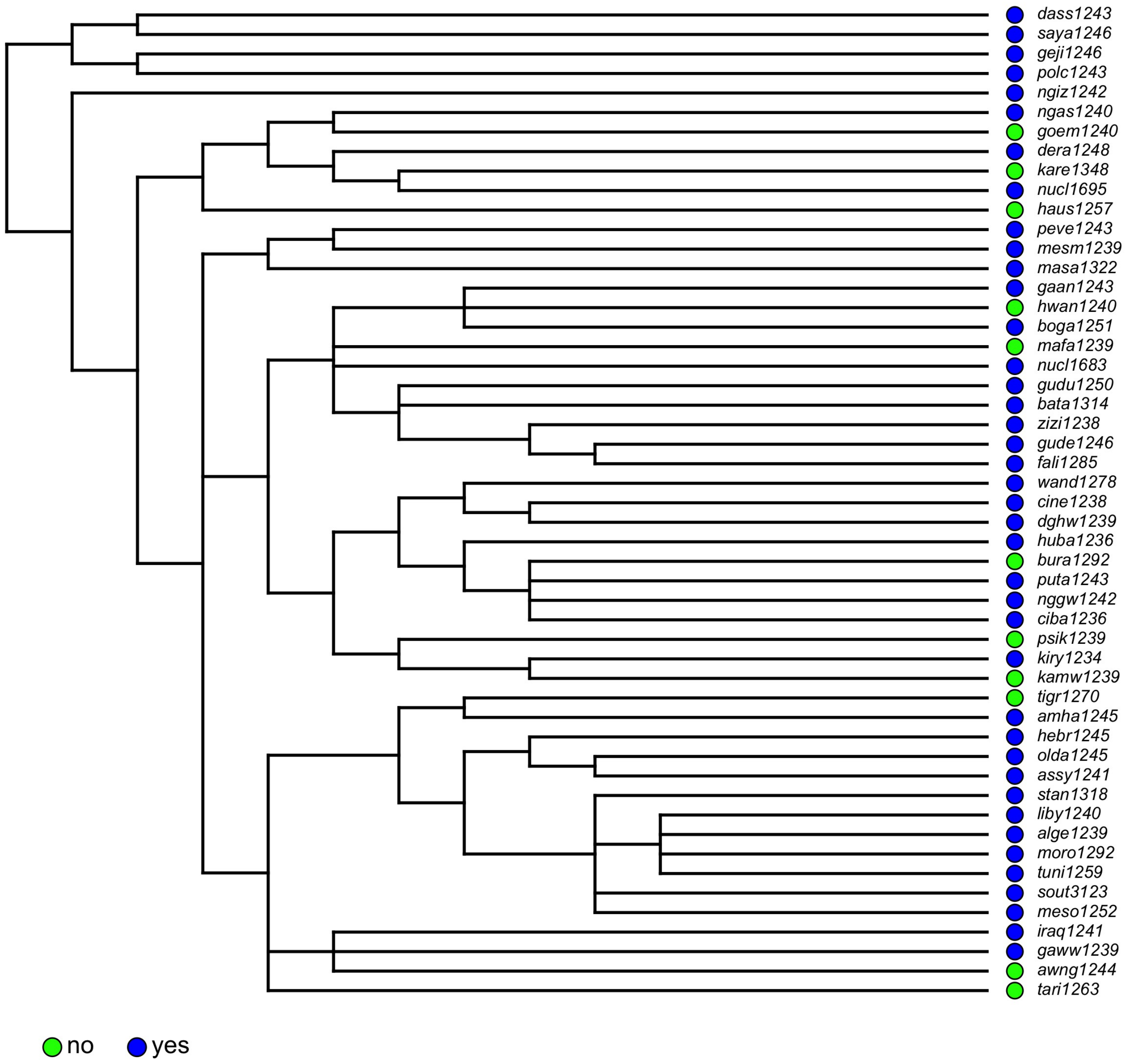

Figure S52. The phylogeny for Afro-Asiatic from Glottolog (ultrametric) (Round, 2021), also showing the languages that have (blue dots) and don't have (green dots) a dedicated word for 'blue'. The languages are represented by their glottocodes (Hammarström and Forkel, 2021). Figure generated using `R` version 4.2.3 (2023-03-15) and package `partykit` (version 1.2.16) and package `phytools` (version 1.2).

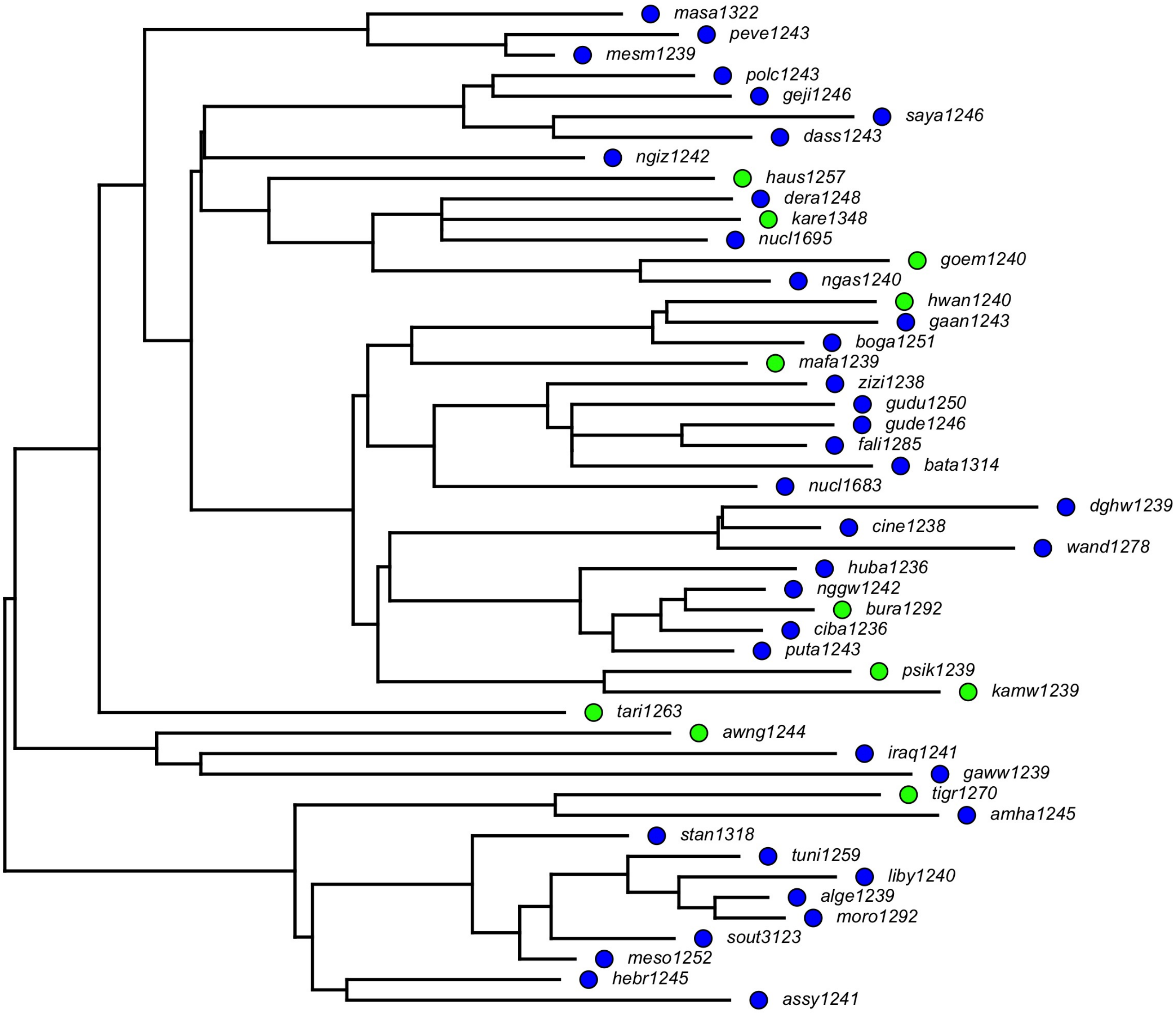

Figure S53. The phylogeny for Afro-Asiatic from Jäger (2018). Conventions as in Figure S52.

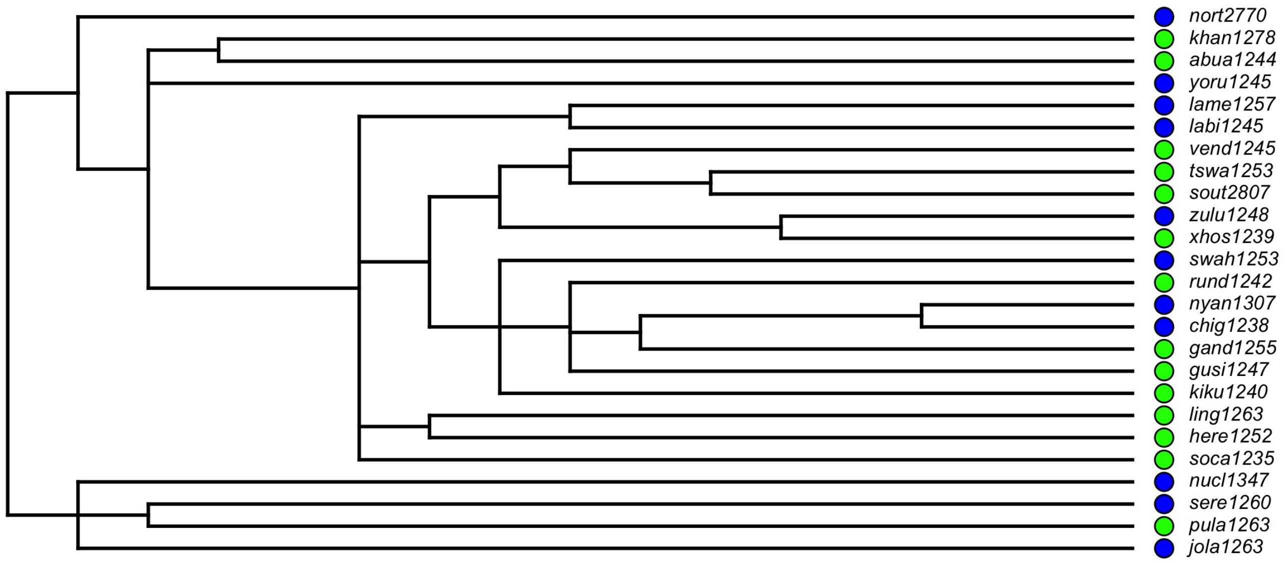

Figure S54. The phylogeny for Atlantic-Congo from Glottolog (ultrametric) (Round, 2021). Conventions as in Figure S52.

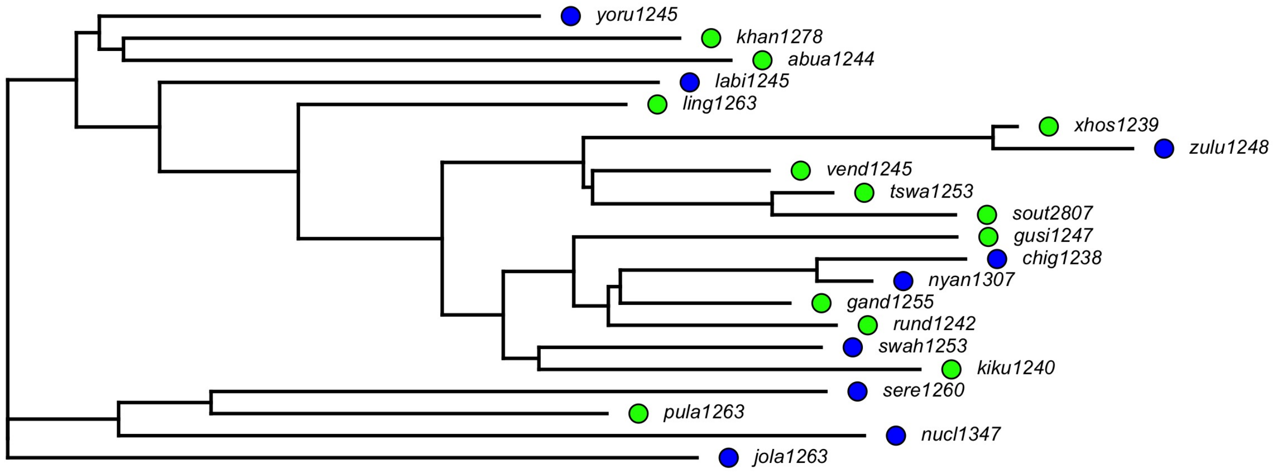

Figure S55. The phylogeny for Atlantic-Congo from Jäger (2018). Conventions as in Figure S52.

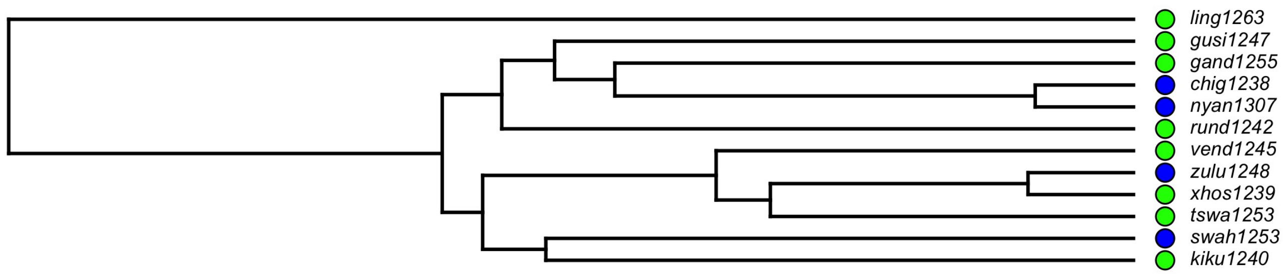

Figure S56. The phylogeny for Bantu (sub-tree of Atlantic-Congo, the MCMC summary tree) from Grollemund et al. (2015). Conventions as in Figure S52.

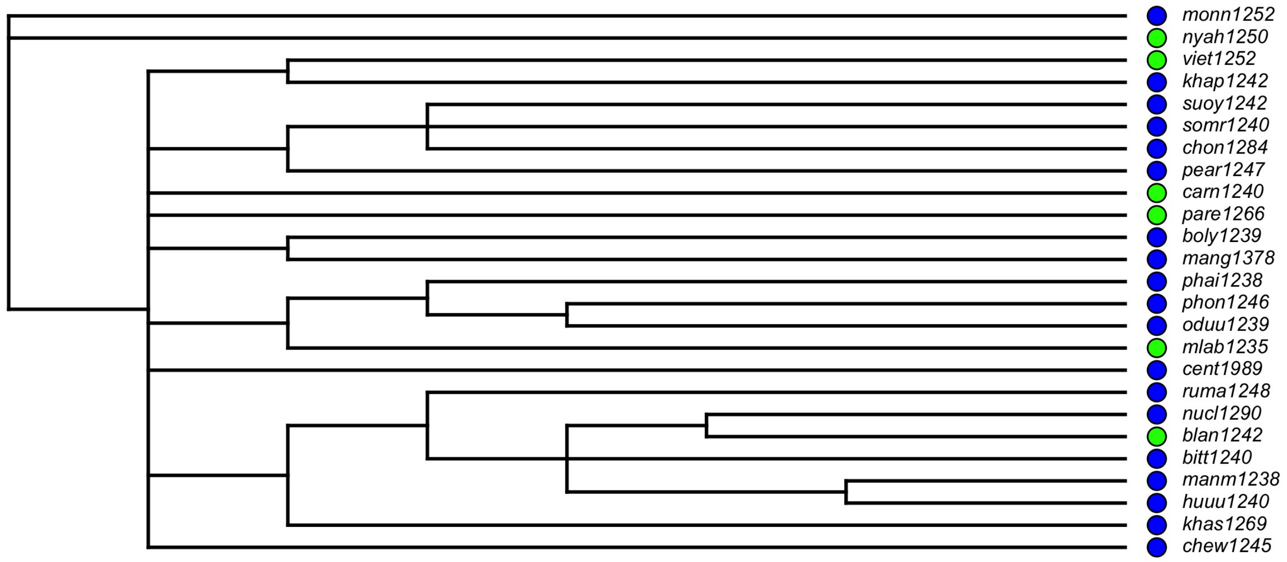

Figure S57. The phylogeny for Austroasiatic from Glottolog (ultrametric) (Round, 2021). Conventions as in Figure S52.

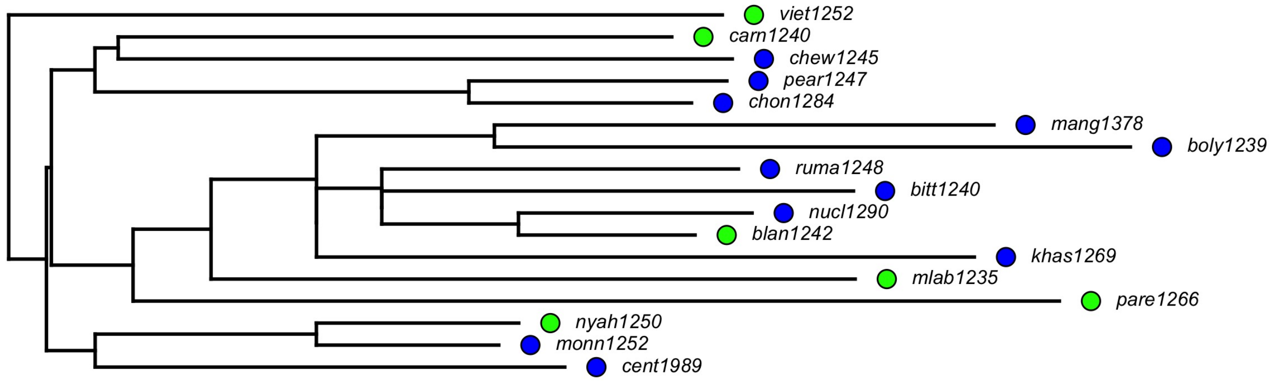

Figure S58. The phylogeny for Austroasiatic from Jäger (2018). Conventions as in Figure S52.

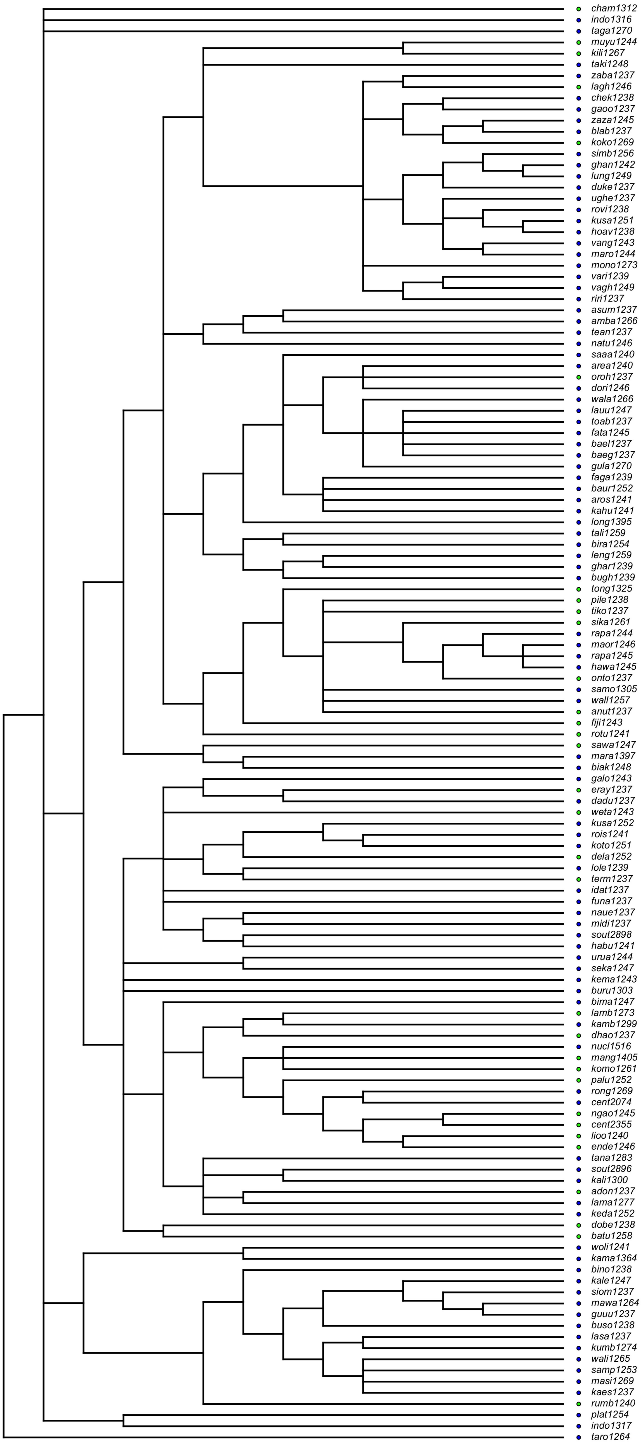

Figure S59. The phylogeny for Austronesian from Glottolog (ultrametric) (Round, 2021). Conventions as in Figure S52. The image size was reduced so as to fit the page.

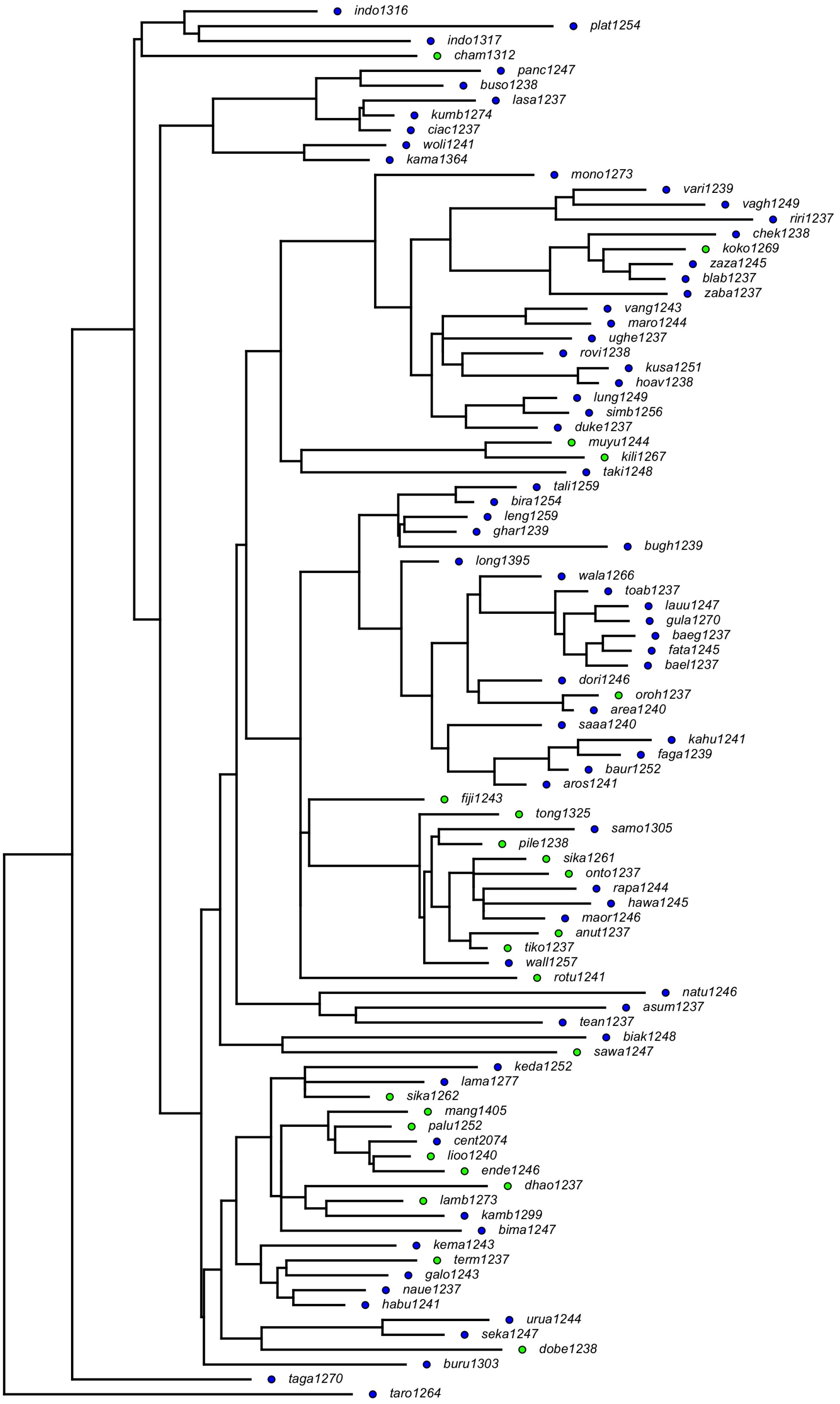

Figure S60. The phylogeny for Austronesian from Jäger (2018). Conventions as in Figure S52. The image size was reduced so as to fit the page.

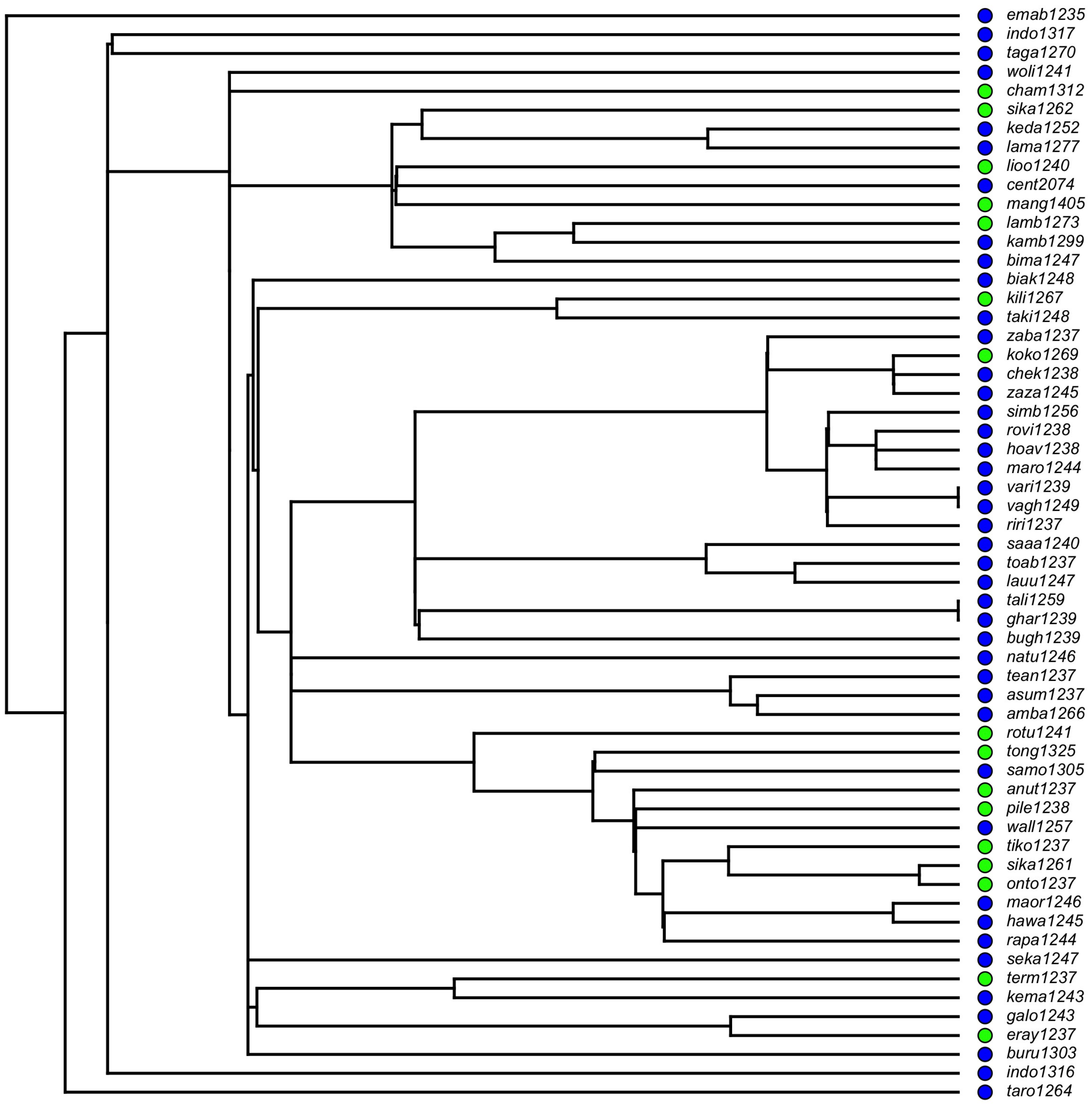

Figure S61. The phylogeny for Austronesian (the MCMC summary tree) from Gray et al. (2009). Conventions as in Figure S52.

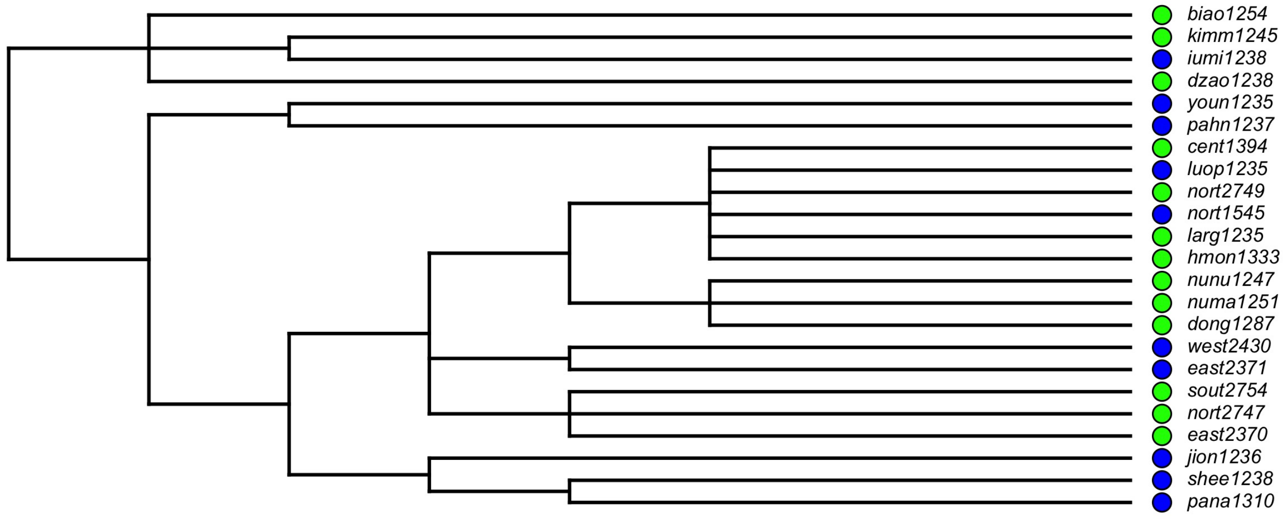

Figure S62. The phylogeny for Hmong-Mien from Glottolog (ultrametric) (Round, 2021). Conventions as in Figure S52.

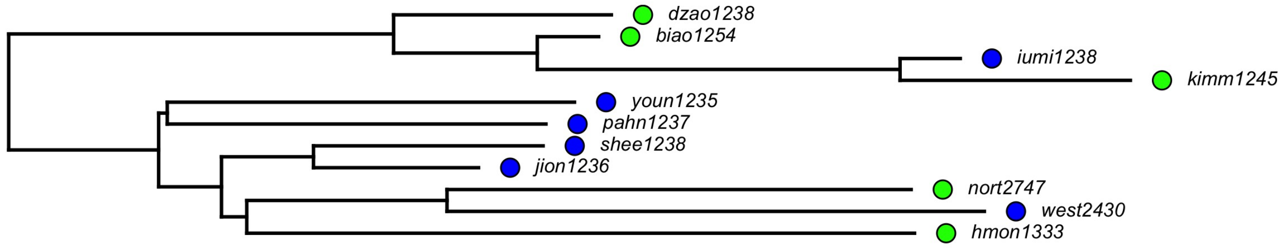

Figure S63. The phylogeny for Hmong-Mien from Jäger (2018). Conventions as in Figure S52.

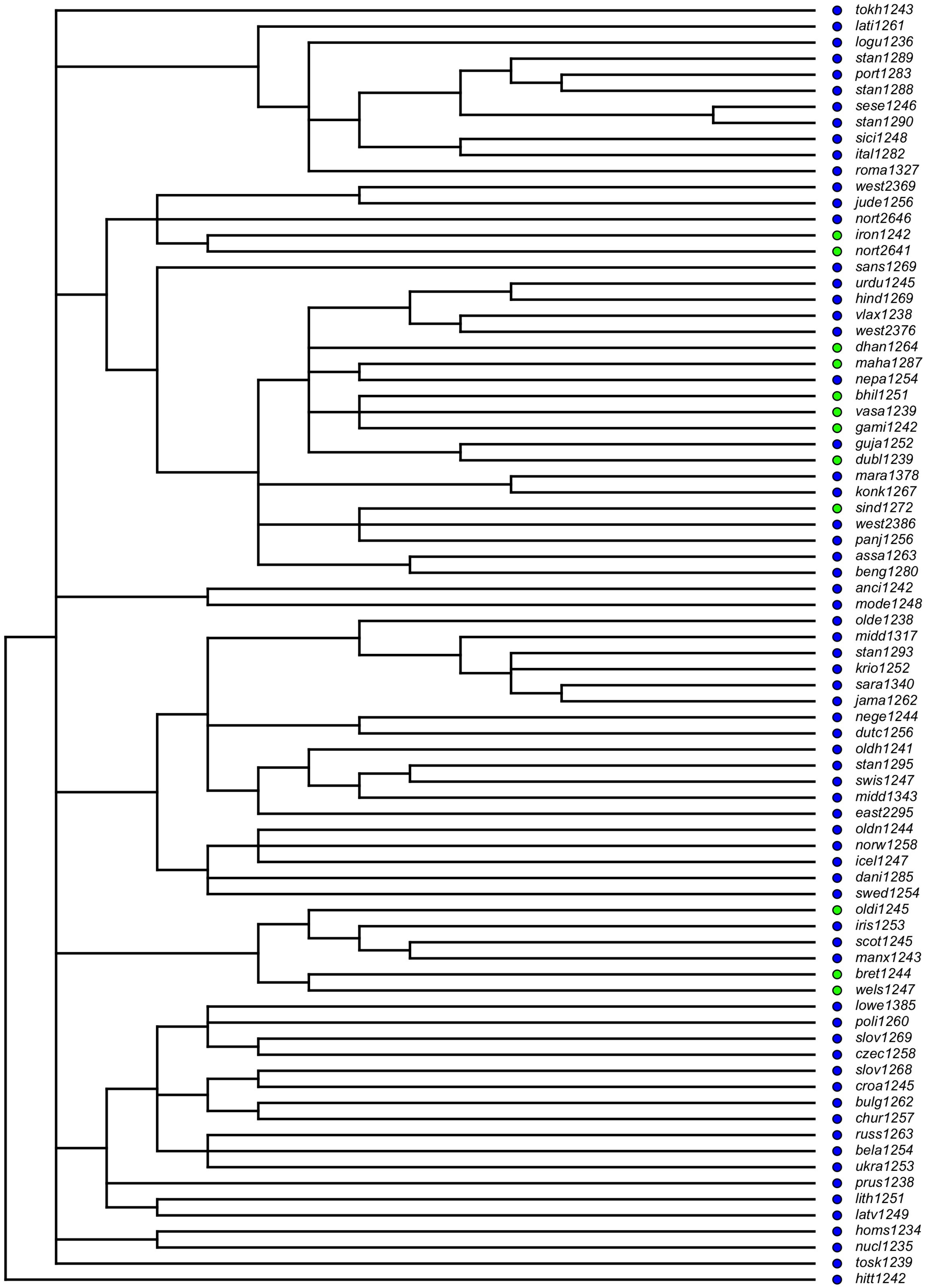
tokh1243
lati1261
logu1236
stan1289
port1283
stan1288
sese1246
stan1290
sici1248
ital1282
roma1327
west2369
jude1256
nort2646
iron1242
nort2641
sans1269
urdu1245
hind1269
vlax1238
west2376
dhan1264
maha1287
nepa1254
bhil1251
vasa1239
gami1242
guja1252
dubl1239
mara1378
konk1267
sind1272
west2386
panj1256
assa1263
beng1280
anci1242
mode1248
olde1238
midd1317
stan1293
krio1252
sara1340
jama1262
nege1244
dutc1256
oldh1241
stan1295
swis1247
midd1343
east2295
oldn1244
norw1258
icel1247
dani1285
swed1254
oldi1245
iris1253
scot1245
manx1243
bret1244
wels1247
lowe1385
poli1260
slov1269
czec1258
slov1268
croa1245
bulg1262
chur1257
russ1263
bela1254
ukra1253
prus1238
lith1251
latv1249
homs1234
nucl1235
tosk1239
hitt1242

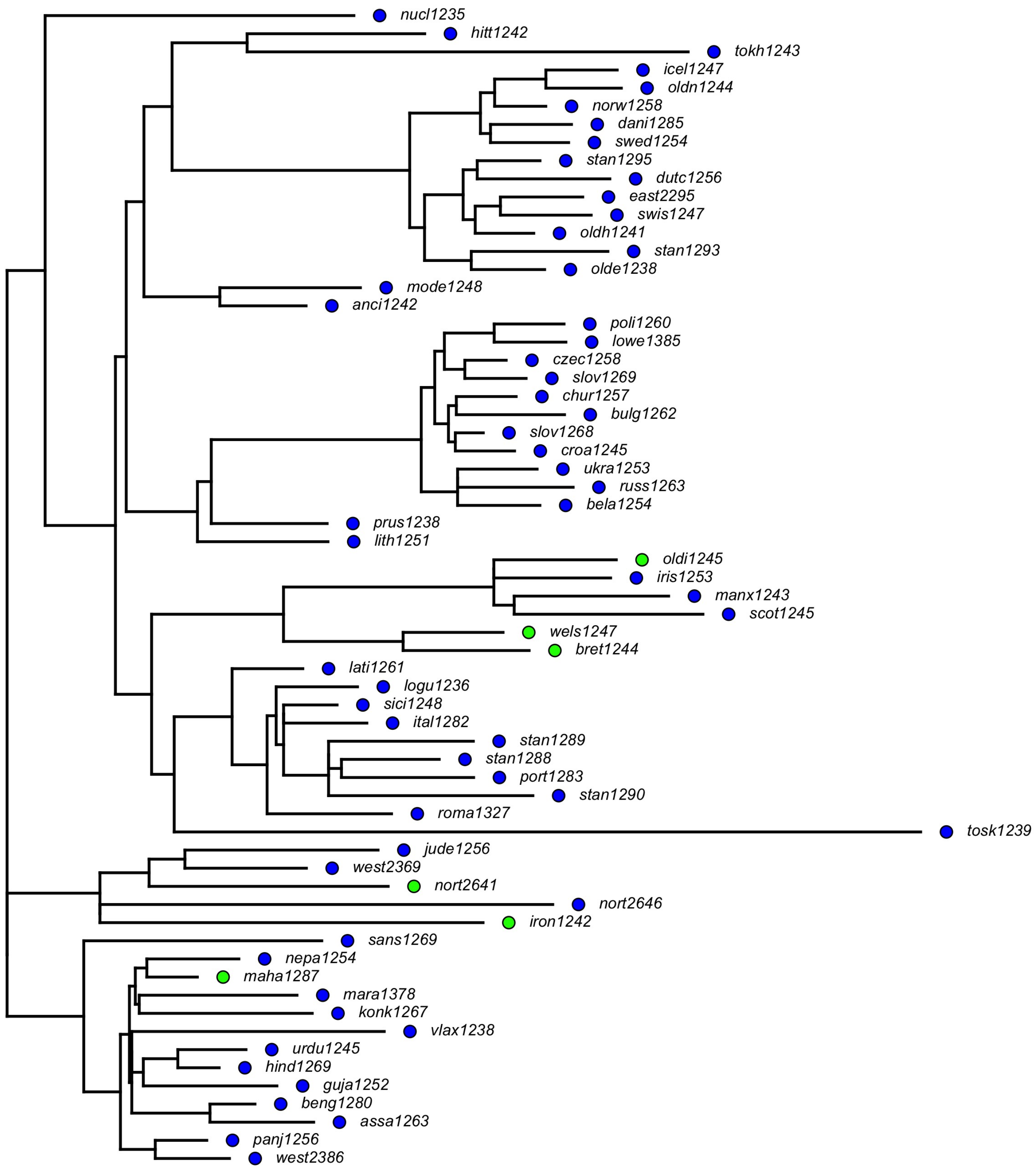

Figure S65. The phylogeny for Indo-European from Jäger (2018). Conventions as in Figure S52.

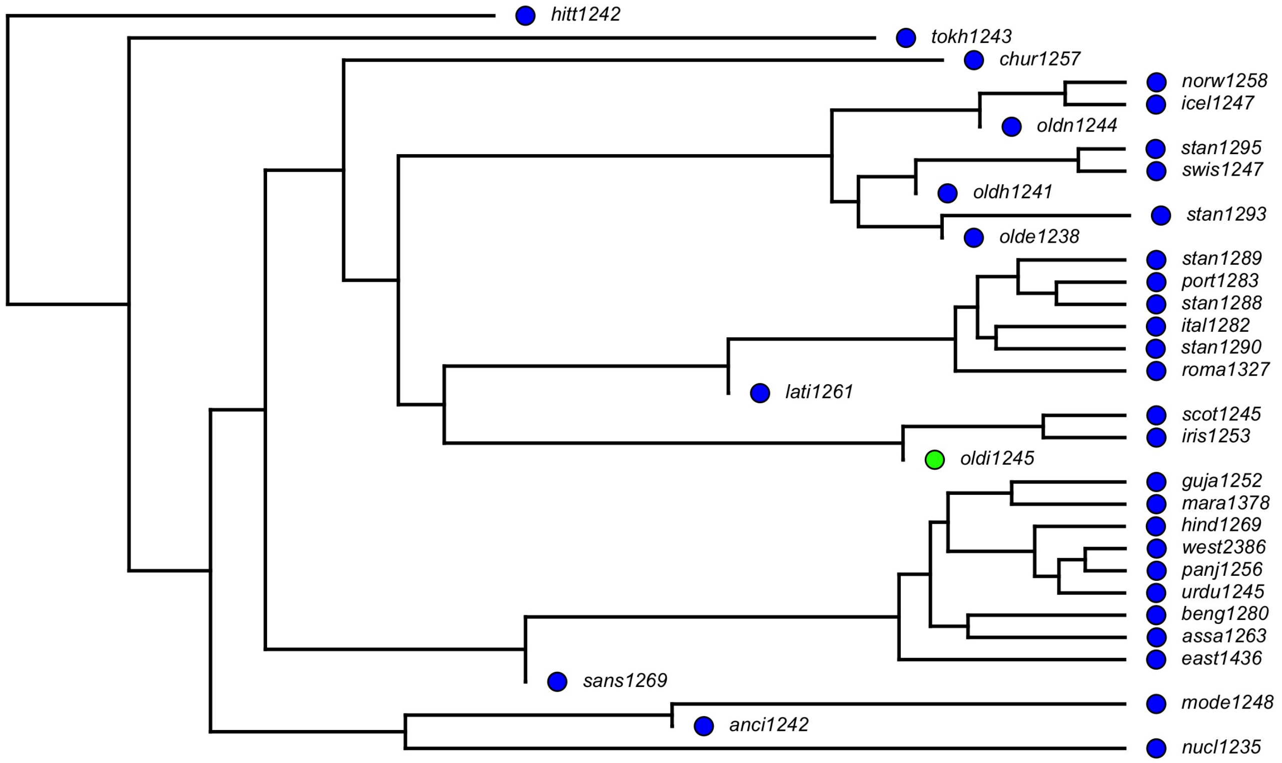

Figure S66. The phylogeny for Indo-European (the MCMC summary tree) from Chang et al. (2015). Conventions as in Figure S52.

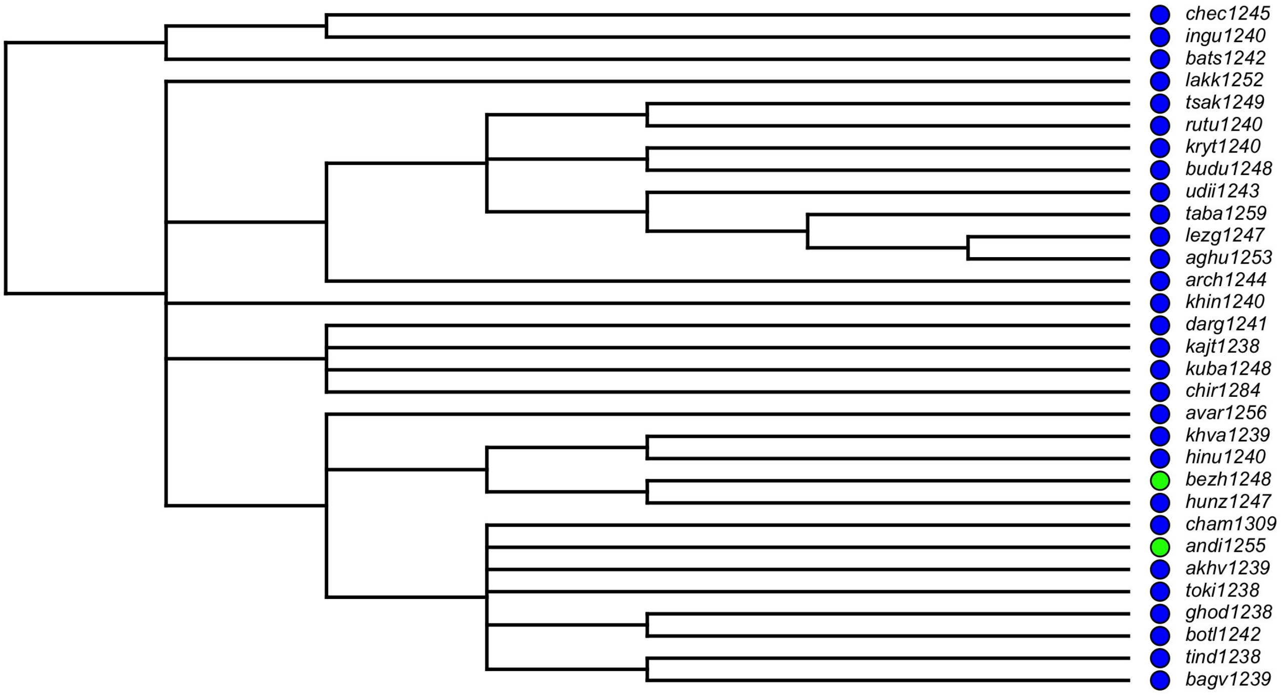

Figure S67. The phylogeny for Nakh-Daghestanian from Glottolog (ultrametric) (Round, 2021). Conventions as in Figure S52.

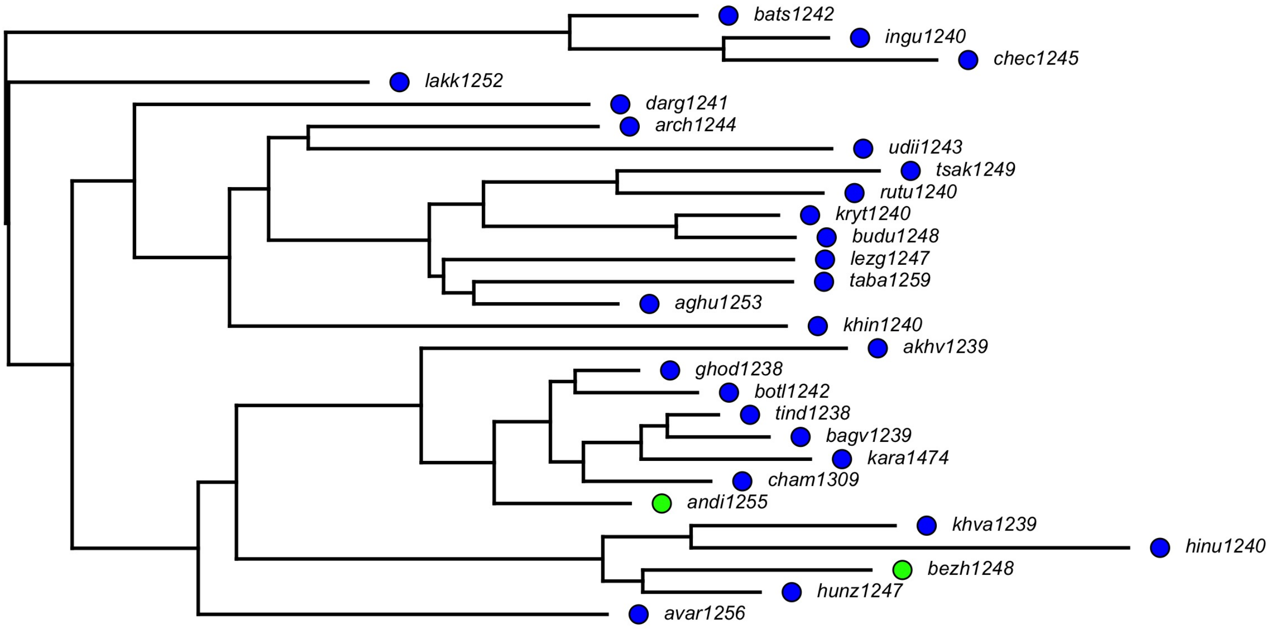

Figure S68. The phylogeny for Nakh-Daghestanian from Jäger (2018). Conventions as in Figure S52.

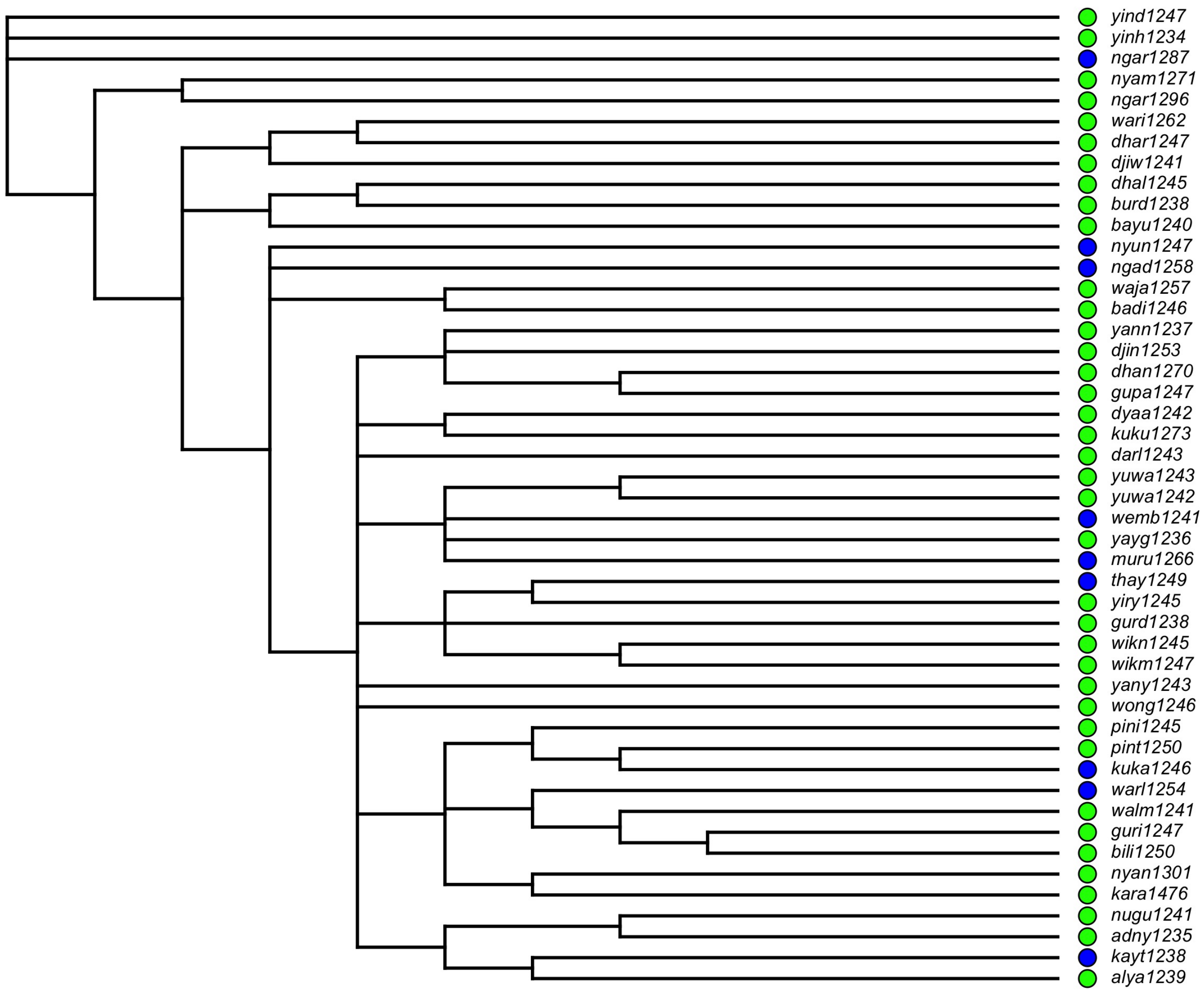

Figure S69. The phylogeny for Pama-Nyungan from Glottolog (ultrametric) (Round, 2021). Conventions as in Figure S52.

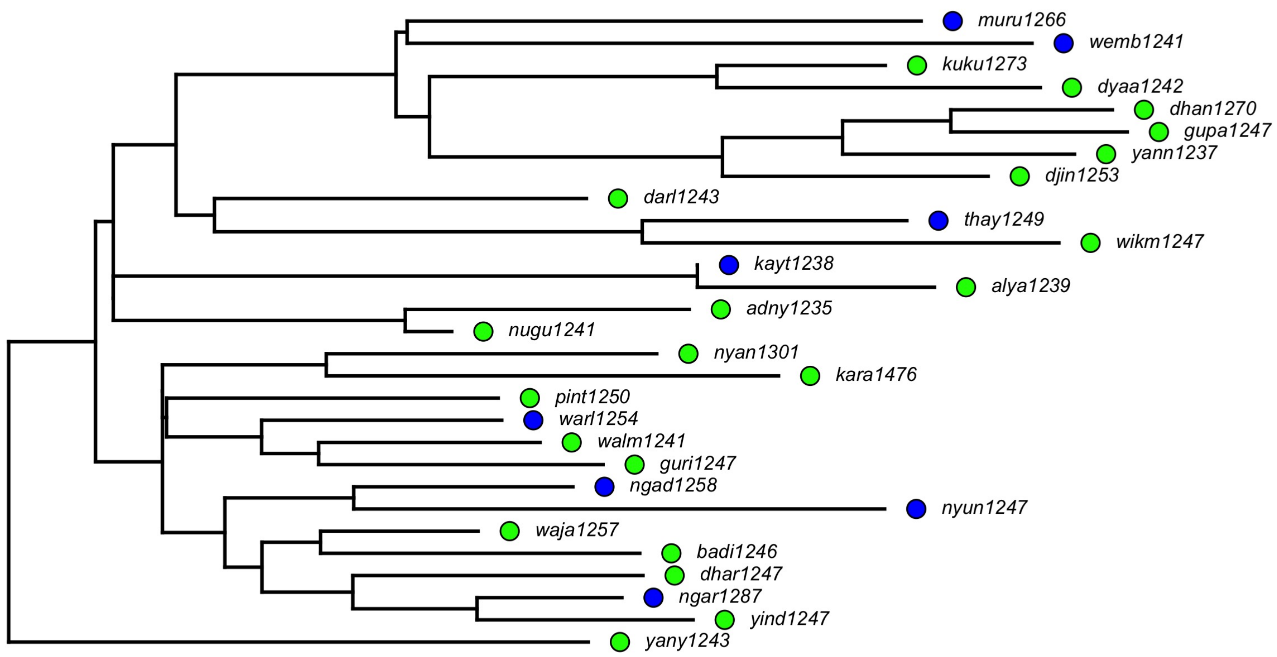

Figure S70. The phylogeny for Pama-Nyungan from Jäger (2018). Conventions as in Figure S52.

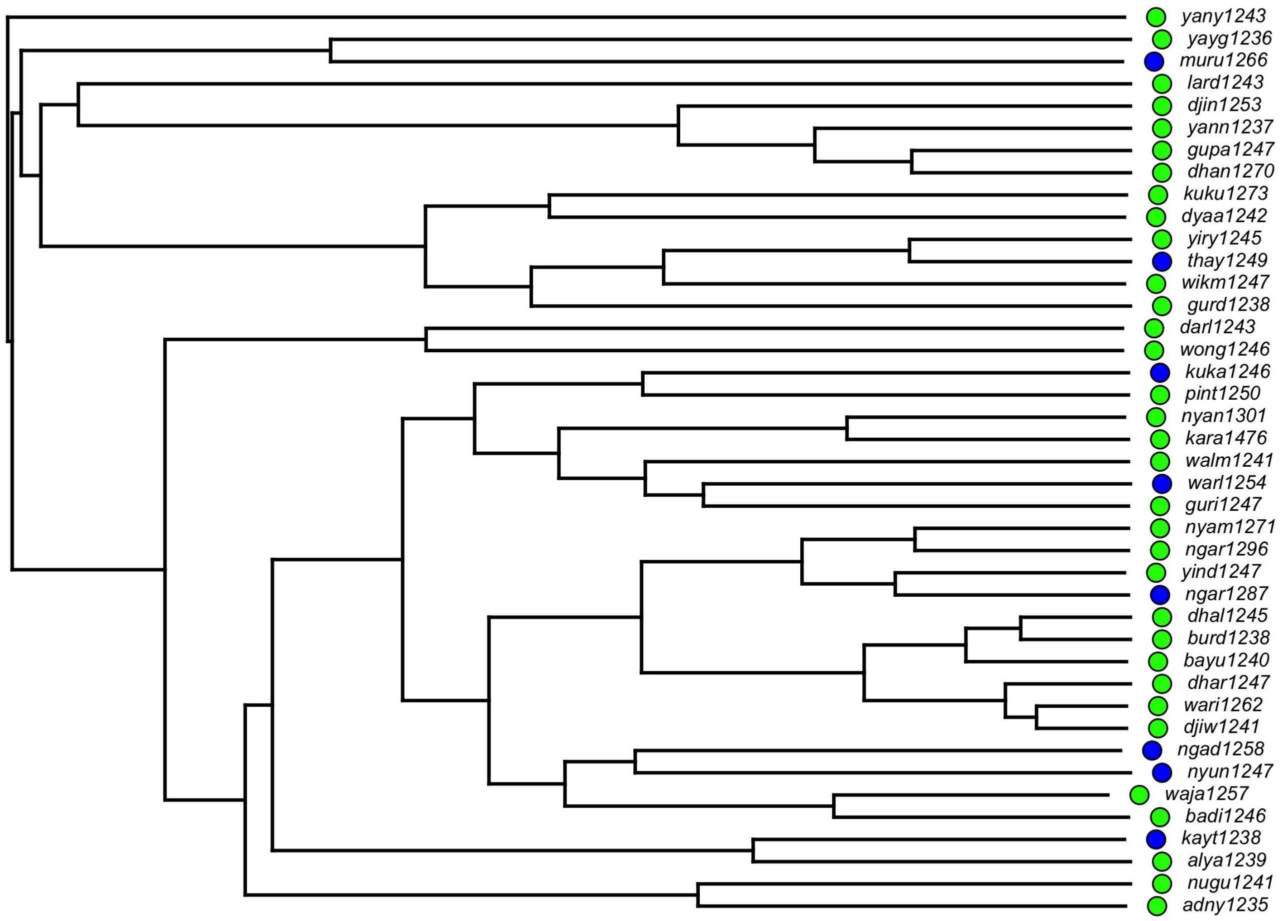

Figure S71. The phylogeny for Pama-Nyungan (the MCMC summary tree) from Bouckaert et al. (2018). Conventions as in Figure S52.

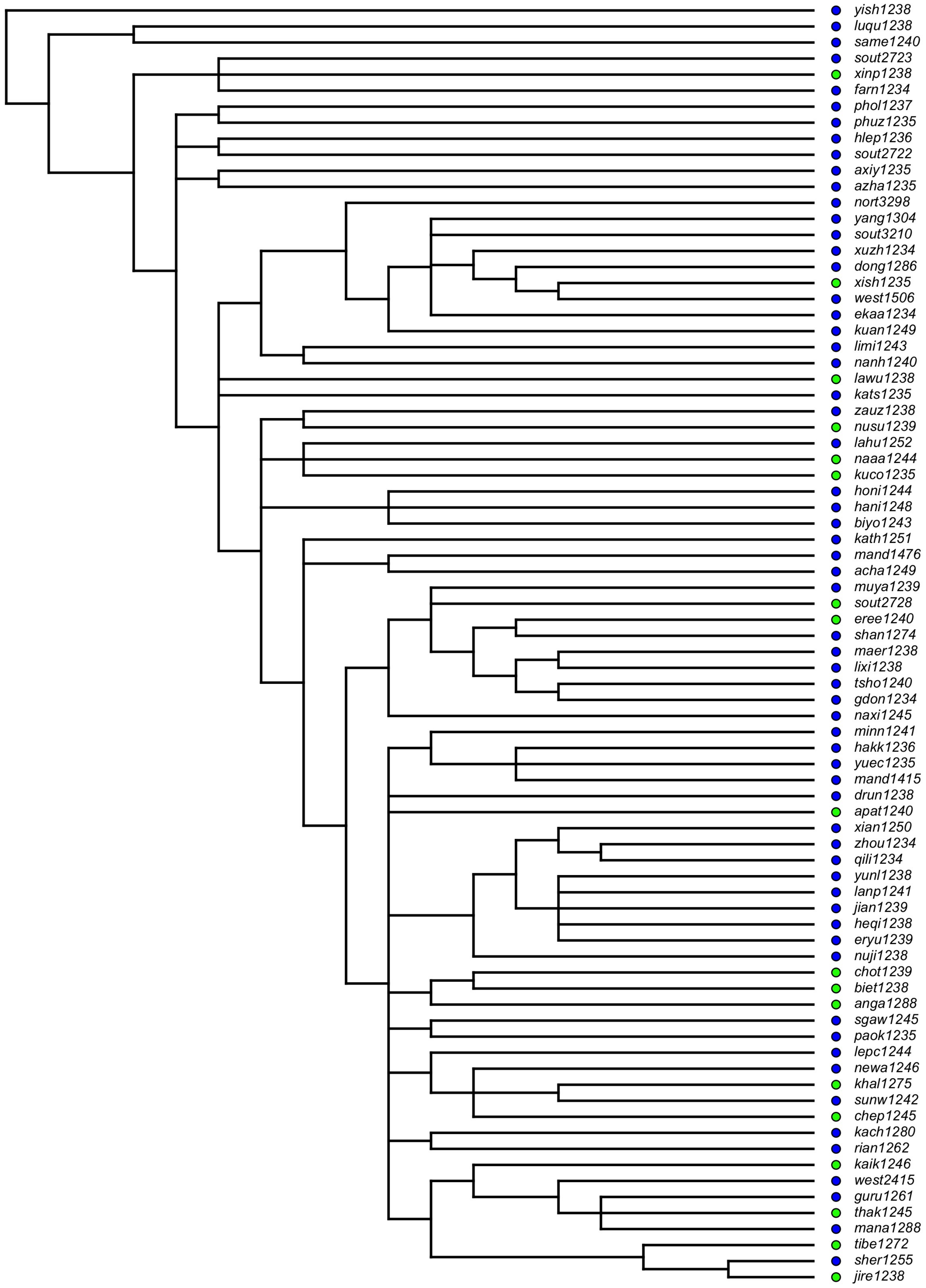

yish1238
luqu1238
same1240
sout2723
xinp1238
farn1234
phol1237
phuz1235
hlep1236
sout2722
axiy1235
azha1235
nort3298
yang1304
sout3210
xuzh1234
dong1286
xish1235
west1506
ekaa1234
kuan1249
limi1243
nanh1240
lawu1238
kats1235
zauz1238
nusu1239
lahu1252
naaa1244
kuco1235
honi1244
hani1248
biyo1243
kath1251
mand1476
acha1249
muya1239
sout2728
eree1240
shan1274
maer1238
lixi1238
tsho1240
gdon1234
naxi1245
minn1241
hakk1236
yuec1235
mand1415
drun1238
apat1240
xian1250
zhou1234
qili1234
yunl1238
lanp1241
jian1239
heqi1238
eryu1239
nuji1238
chot1239
biet1238
anga1288
sgaw1245
paok1235
lepc1244
newa1246
khal1275
sunw1242
chep1245
kach1280
rian1262
kaik1246
west2415
guru1261
thak1245
mana1288
tibe1272
sher1255
jire1238

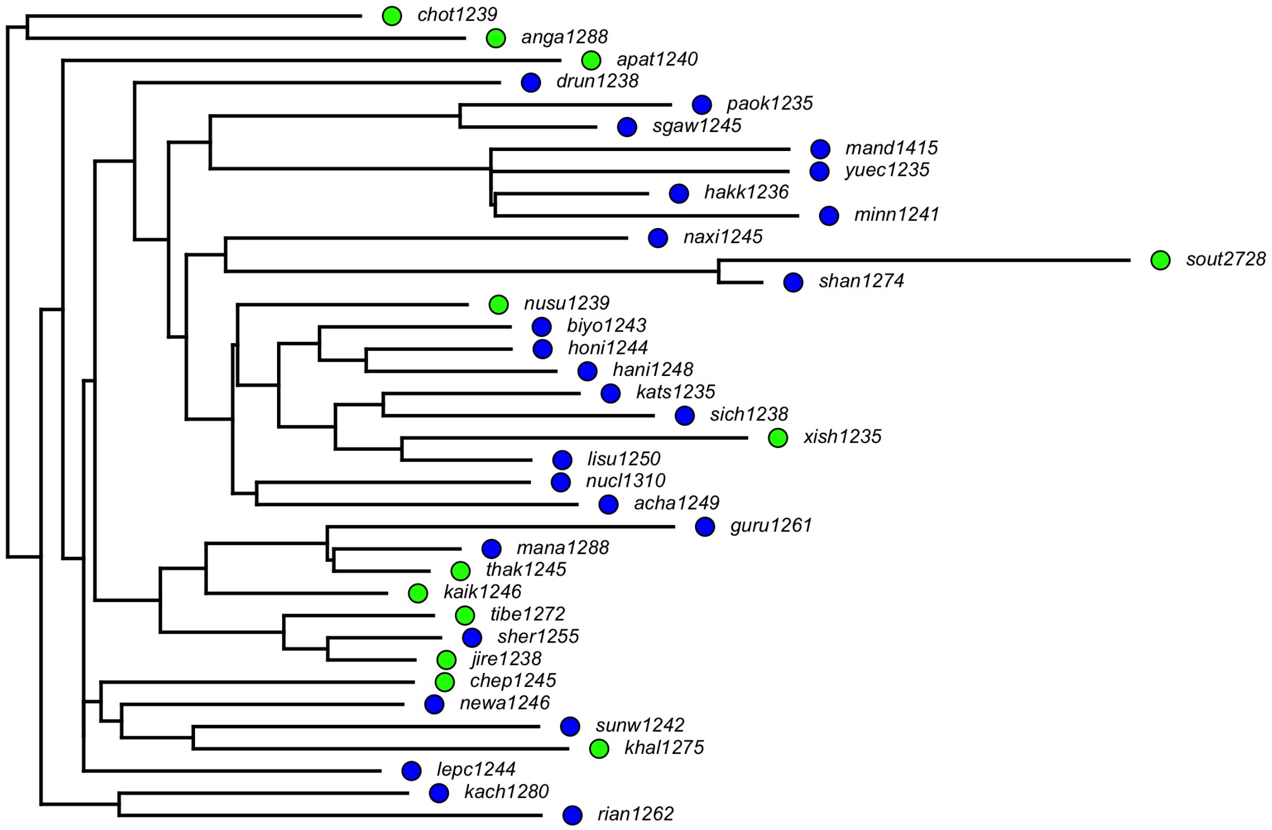

Figure S73. The phylogeny for Sino-Tibetan from Jäger (2018). Conventions as in Figure S52.

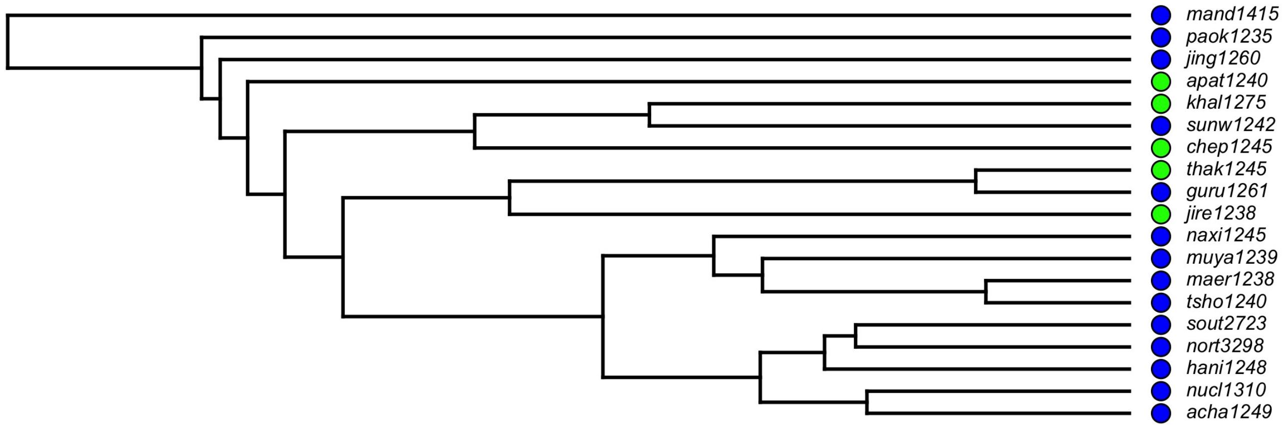

Figure S74. The phylogeny for Sino-Tibetan (the MCMC summary tree) from Zhang et al. (2019). Conventions as in Figure S52.

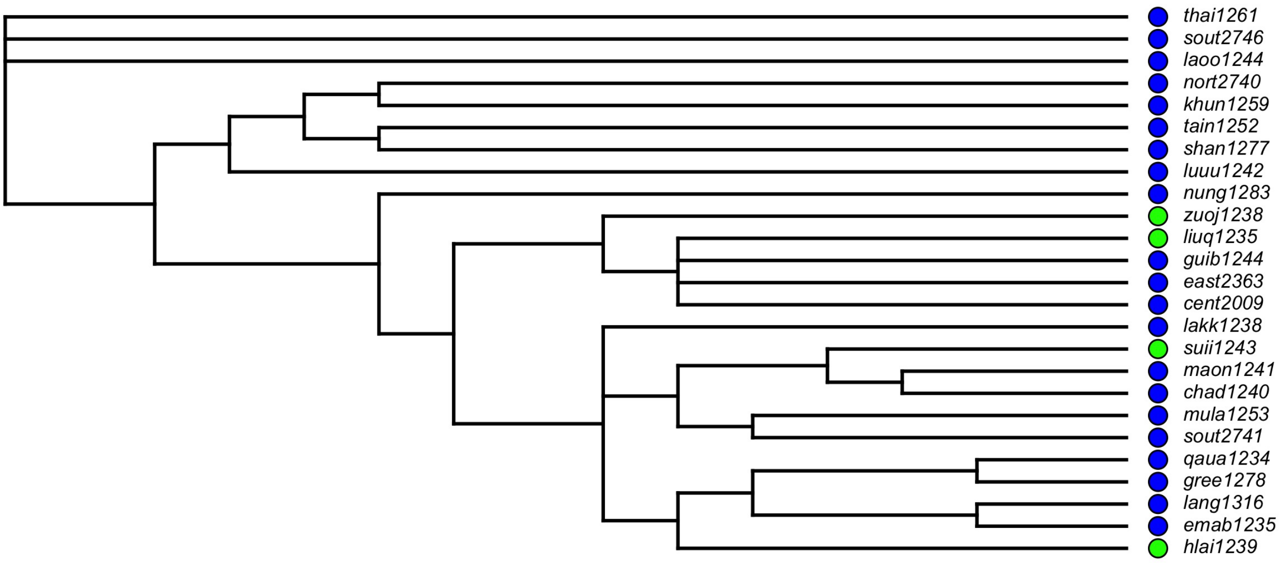

Figure S75. The phylogeny for Tai-Kadai from Glottolog (ultrametric) (Round, 2021). Conventions as in Figure S52.

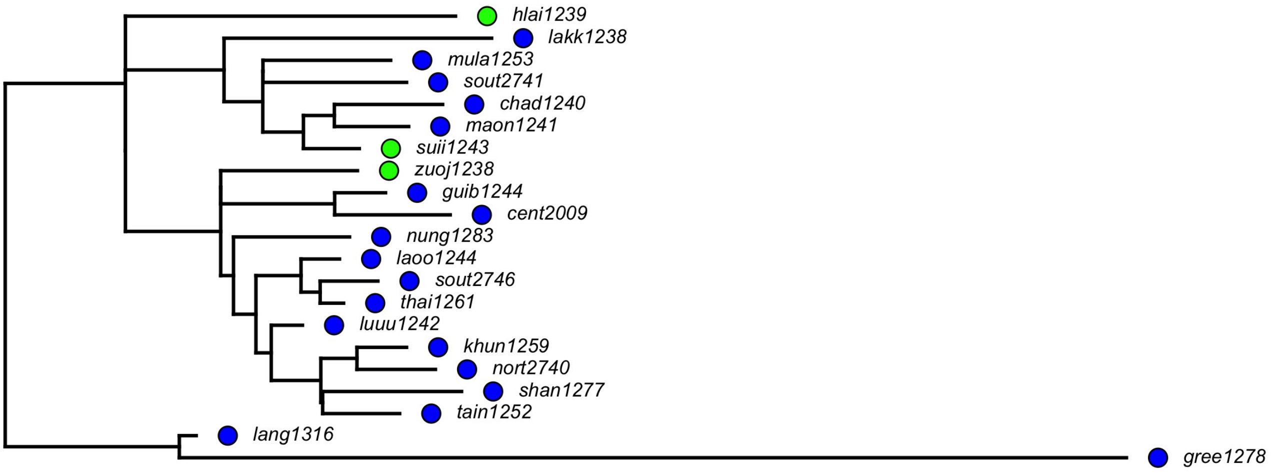

Figure S76. The phylogeny for Tai-Kadai from Jäger (2018). Conventions as in Figure S52.

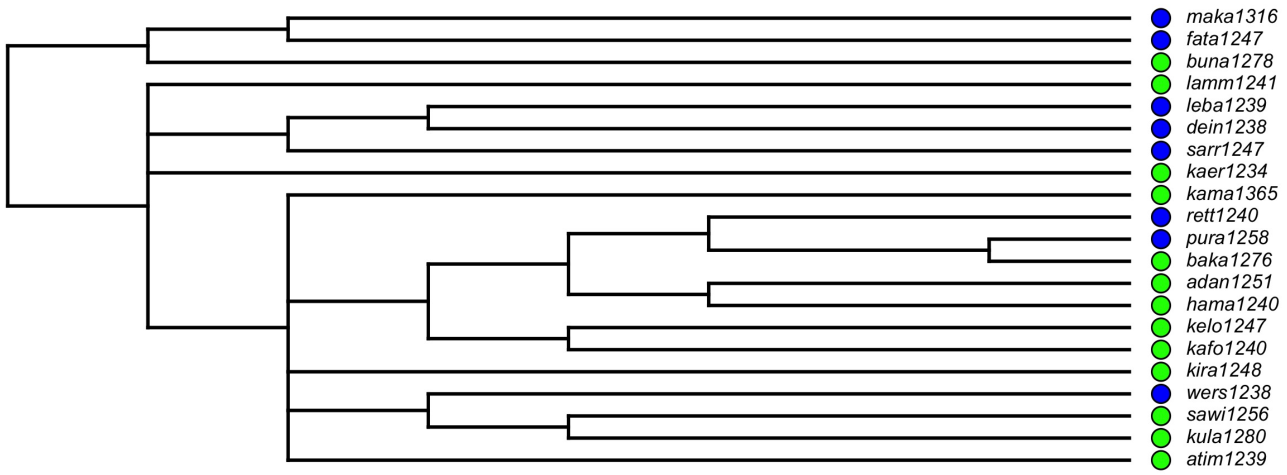

Figure S77. The phylogeny for Timor-Alor-Pantar from Glottolog (ultrametric) (Round, 2021). Conventions as in Figure S52.

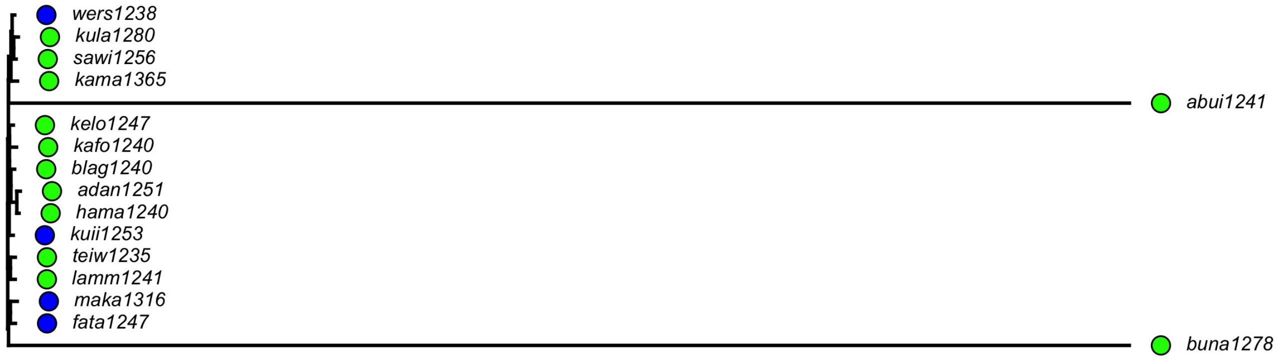

Figure S78. The phylogeny for Timor-Alor-Pantar from Jäger (2018). Conventions as in Figure S52.

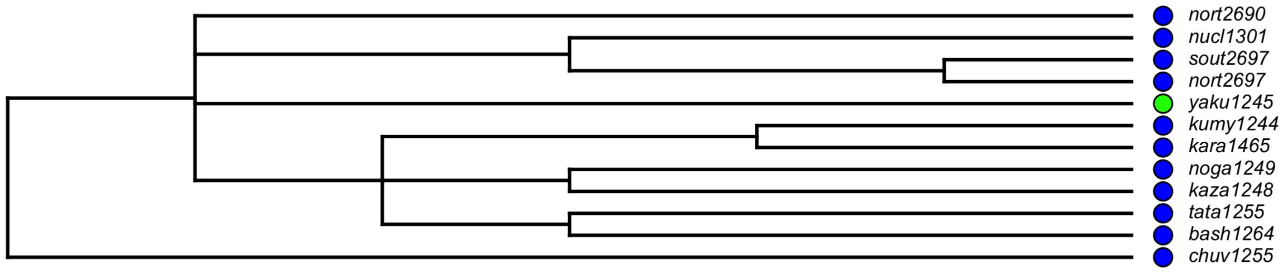

Figure S79. The phylogeny for Turkic from Glottolog (ultrametric) (Round, 2021). Conventions as in Figure S52.

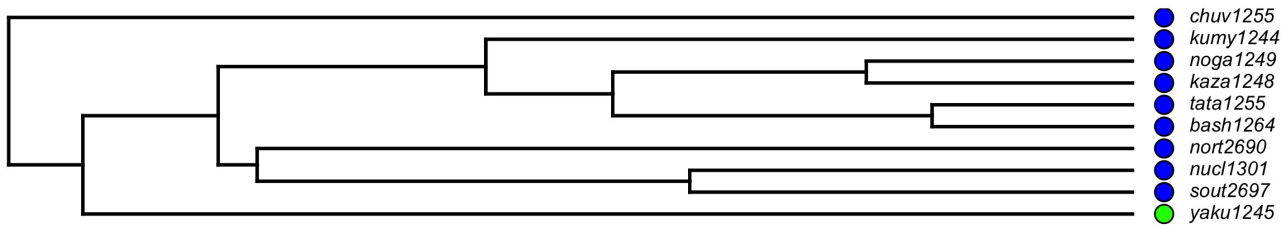

Figure S80. The phylogeny for Turkic (the MCMC summary tree) from Hruschka et al. (2015). Conventions as in Figure S52.

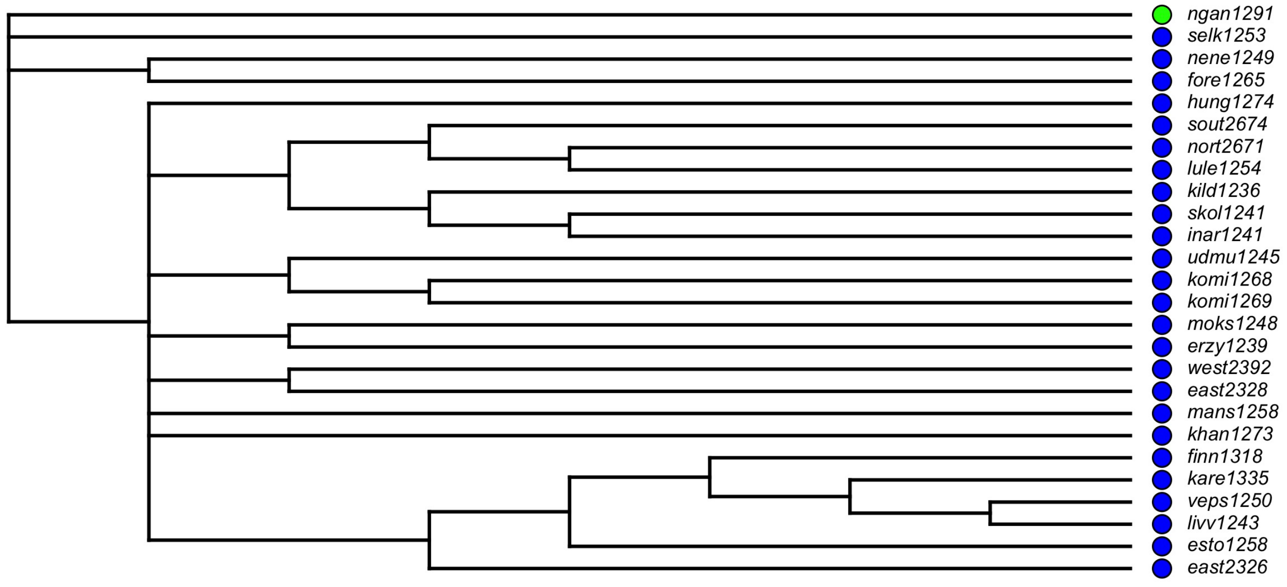

Figure S81. The phylogeny for Uralic from Glottolog (ultrametric) (Round, 2021). Conventions as in Figure S52.

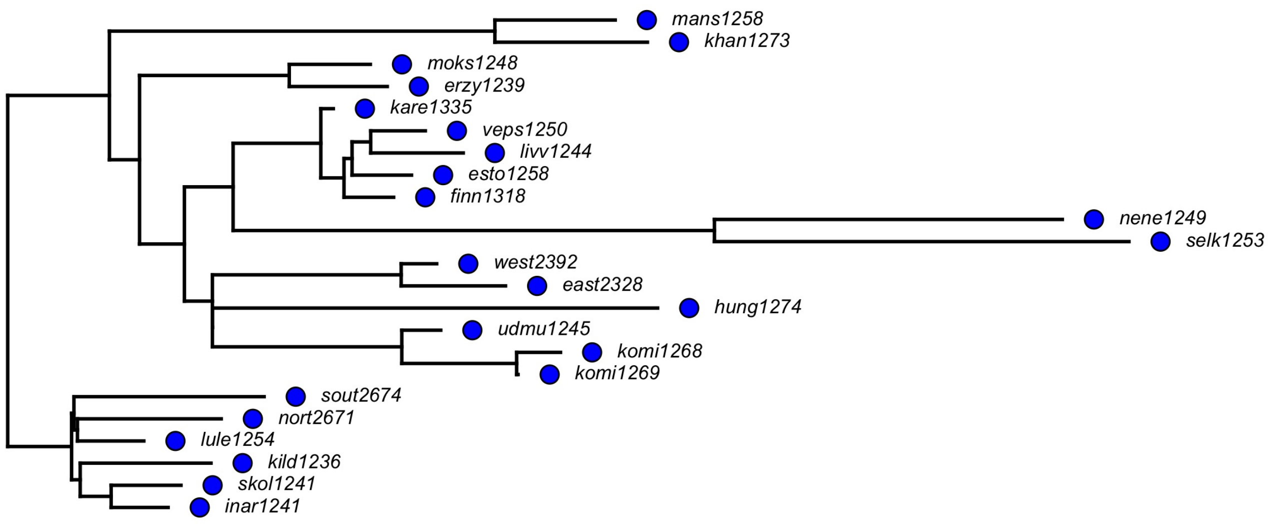

Figure S82. The phylogeny for Uralic from Jäger (2018). Conventions as in Figure S52.

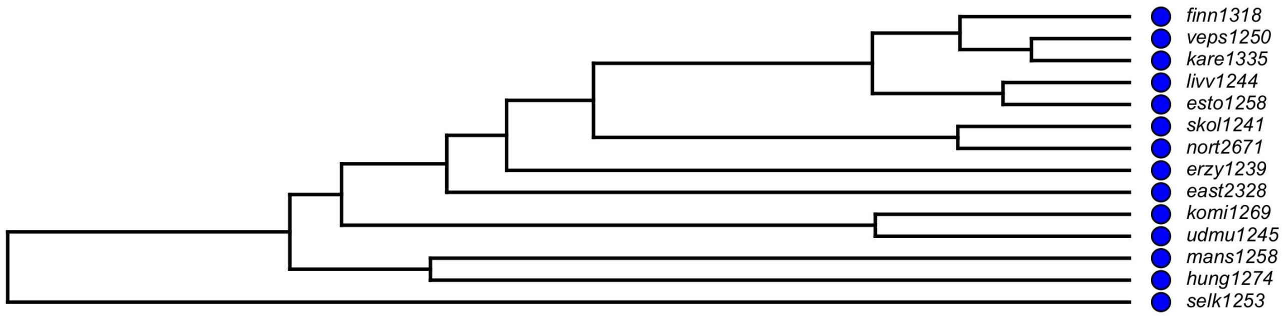

Figure S83. The phylogeny for Uralic (the MCMC summary tree) from Honkola et al. (2013). Conventions as in Figure S52.

Figure S84. Phylogenetic (and non-phylogenetic "control") regressions of *blue* on individual predictors. Please note that **the image is missing on purpose**, as this caption will be referred to from the actual figures below. Each row of plots shows a single family (AA = "Afro-Asiatic", AC/B = "Atlantic-Congo/Bantu", Aas = "Austroasiatic", Aan = "Austronesian", HM = "Hmong-Mien", IE = "Indo-European", ND = "Nakh-Daghestanian", PN = "Pama-Nyungan", ST = "Sino-Tibetan" , TK = "Tai-Kadai", TAP = "Timor-Alor-Pantar", T = "Turkic", and U = "Uralic") or the "global" trees (bottom-most row). Each column of plots shows a single method: GLM = frequentist "standard" logistic regression without controlling for phylogeny, PGLMM = Phylogenetic Generalized Linear Mixed Model for Binary Data as implemented by `binaryPGLMM()` in package `ape`, IG10 = phylogenetic logistic regression of Ives and Garland (2009) as implemented by `phyloglm()` in package `phylolm` which optimizes a GEE approximation to the penalized likelihood of the logistic regression, MPLE = phylogenetic logistic regression of Ives and Garland (2009) as implemented by `phyloglm()` in package `phylolm` which maximizes the penalized likelihood of the logistic regression, BRMSf = "standard" Bayesian logistic regression without controlling for phylogeny (as implemented by `brms`), and BRMS = Bayesian logistic regression controlling for phylogeny (as implemented by `brms` using `gr(glottocode, cov=A)` where `A` is the phylogenetic variance-covariance matrix). The actual trees are shown within each plot on the x-axis, and are abbreviated as: G(o) = "Glottolog (original)", G(x) = "Glottolog (exponential)", G(u) = "Glottolog (ultrametric)", M(s) = "MCMC (summary)", M(p) = "MCMC (posterior)", and J18 = "Jäger (2018)"; for plotting purposes, the Sino-Tibetan MCC tree of Zhang et al. (2019) and the MCC global phylogeny of Bouckaert et al. (2022) are considered as M(s) (i.e., as "MCMC (summary)"). Missing M(s) and M(p) mean that there are no posterior trees available for the family. The y-axis shows, for each family, method and tree, the point estimate (as a dot) of the regression coefficient β, its incertitude (as $\pm$1sd and $\pm$2sd, in darker and lighter colored segments, respectively, for the non-Bayesian methods, and the 89% and 95%HDI for the Bayesian methods, respectively), and its nominal statistical significance (as color; for the Bayesian methods, the posterior probability $p(\beta = 0)$ was used). The M(p) trees were plotted superposed. The dotted red line represents 0.0, and the thick transparent vertical red lines represent cases where *blue* is constant on the tree. Each plot has its own y-axis limits to allow the plotting of the full range without swamping the methods with smaller and tighter estimates by those with bigger or less precise ones, but makes comparing the results between methods and families trickier. Figure generated using `R` version 4.2.3 (2023-03-15) and package `ggplot2` (version 3.4.0).

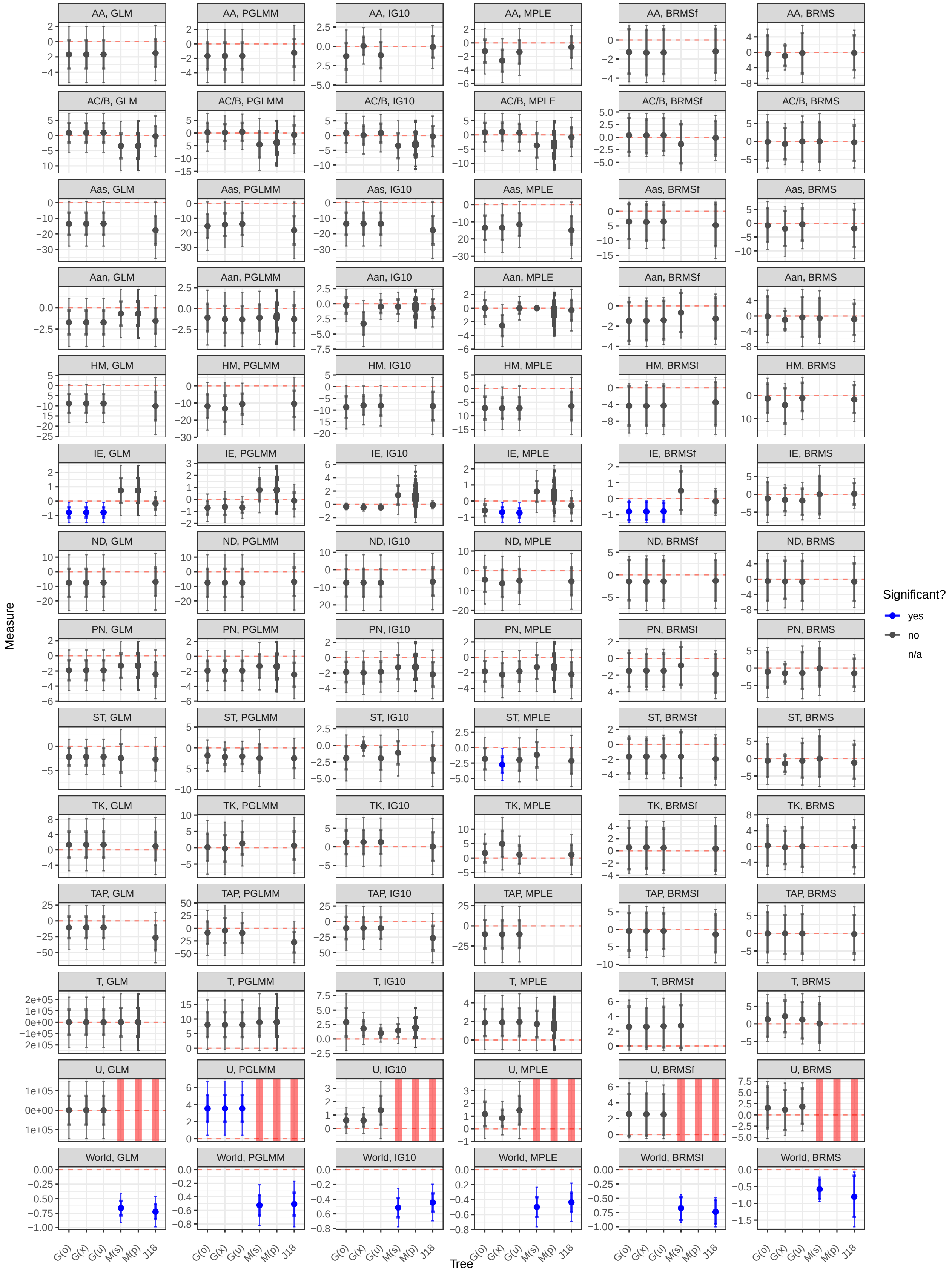

Figure S85. Phylogenetic (and non-phylogenetic "control") regressions of *blue* on *UV-B mean*. Please see Figure S84's caption for explanations.

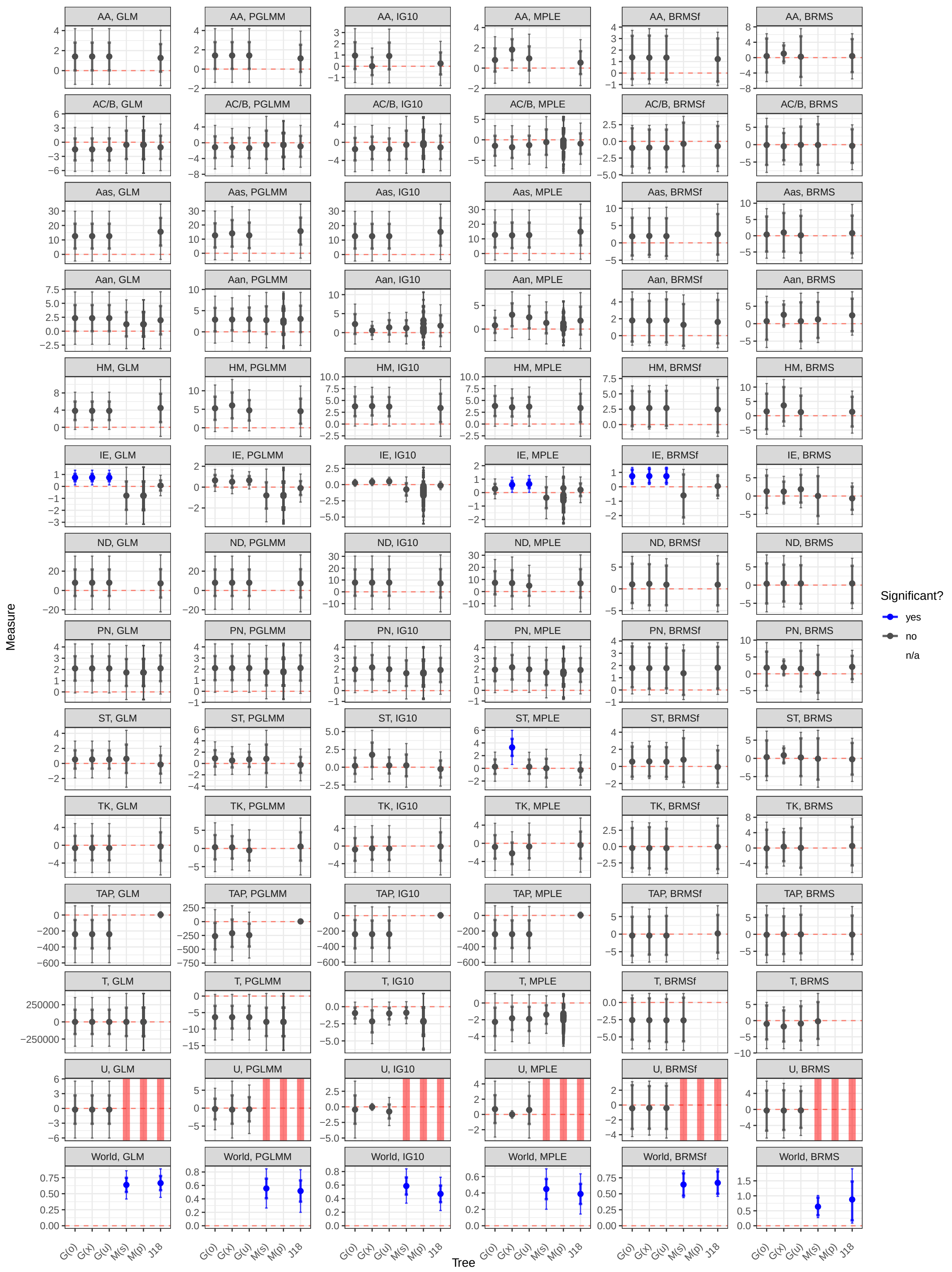

Figure S86. Phylogenetic (and non-phylogenetic "control") regressions of *blue* on *UV-B sd*. Please see Figure S84's caption for explanations.

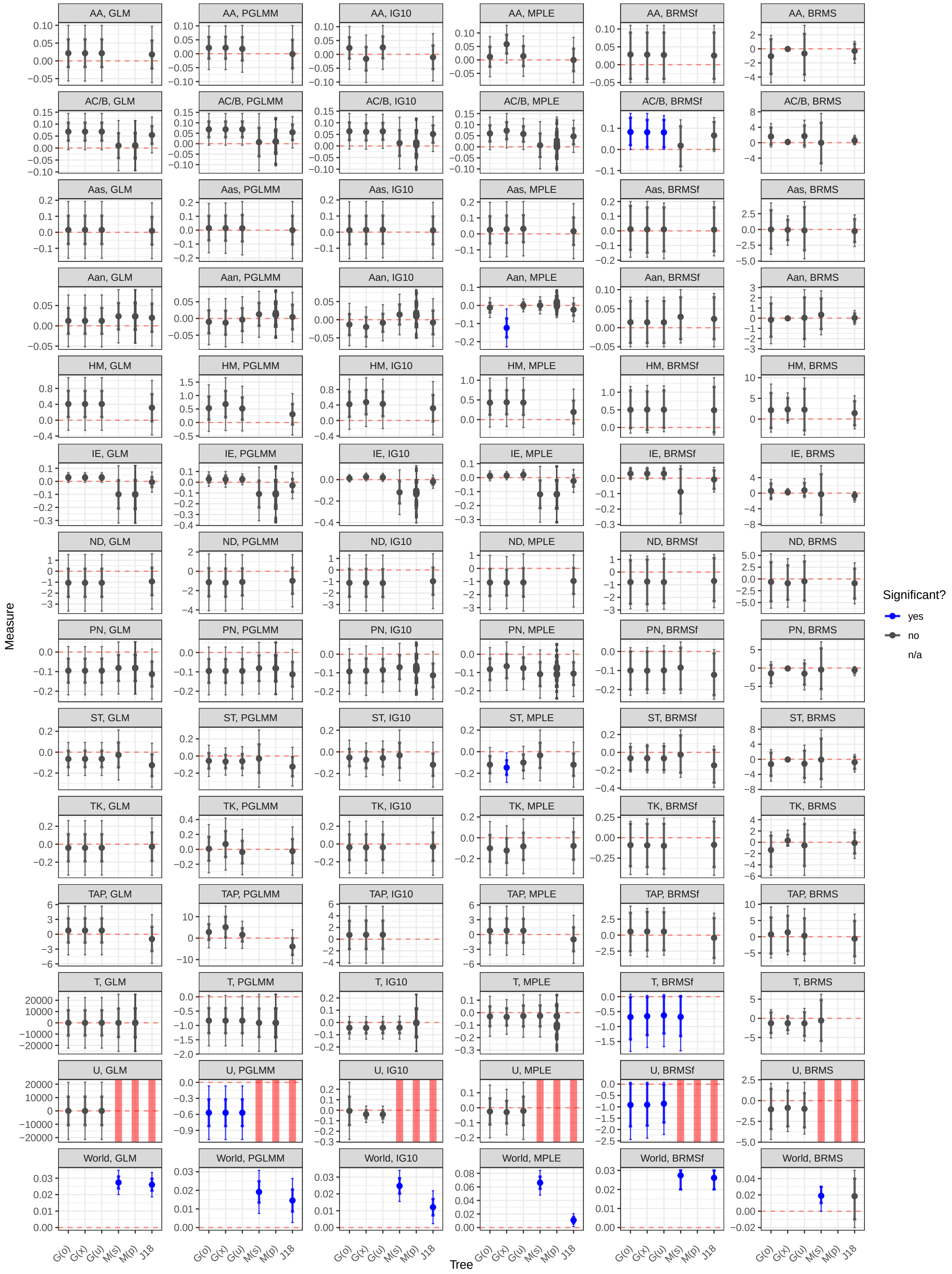

Figure S87. Phylogenetic (and non-phylogenetic "control") regressions of *blue* on *latitude*. Please see Figure S84's caption for explanations.

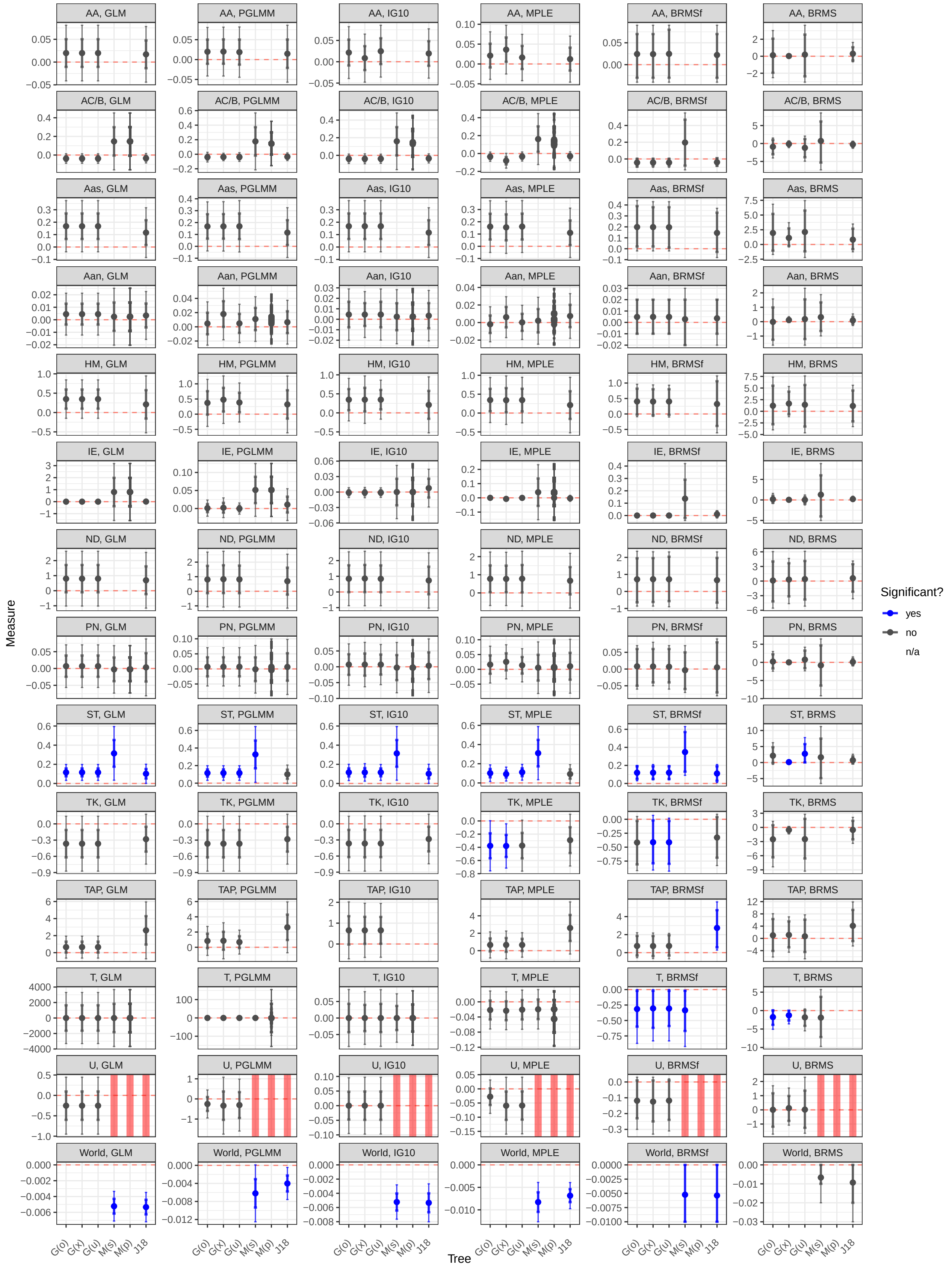

Figure S88. Phylogenetic (and non-phylogenetic "control") regressions of *blue* on *longitude*. Please see Figure S84's caption for explanations.

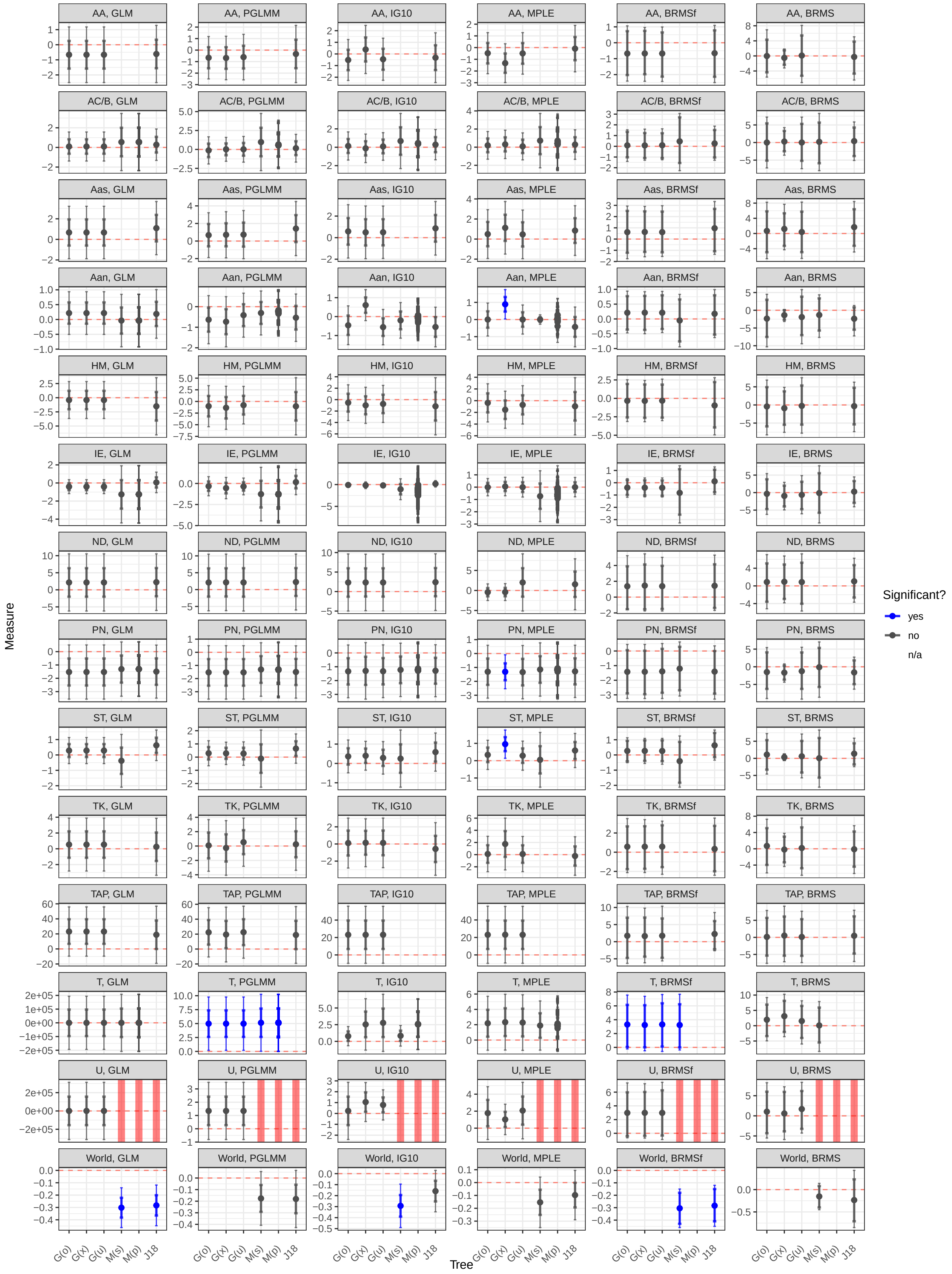

Figure S89. Phylogenetic (and non-phylogenetic "control") regressions of *blue* on *climate PC1*. Please see Figure S84's caption for explanations.

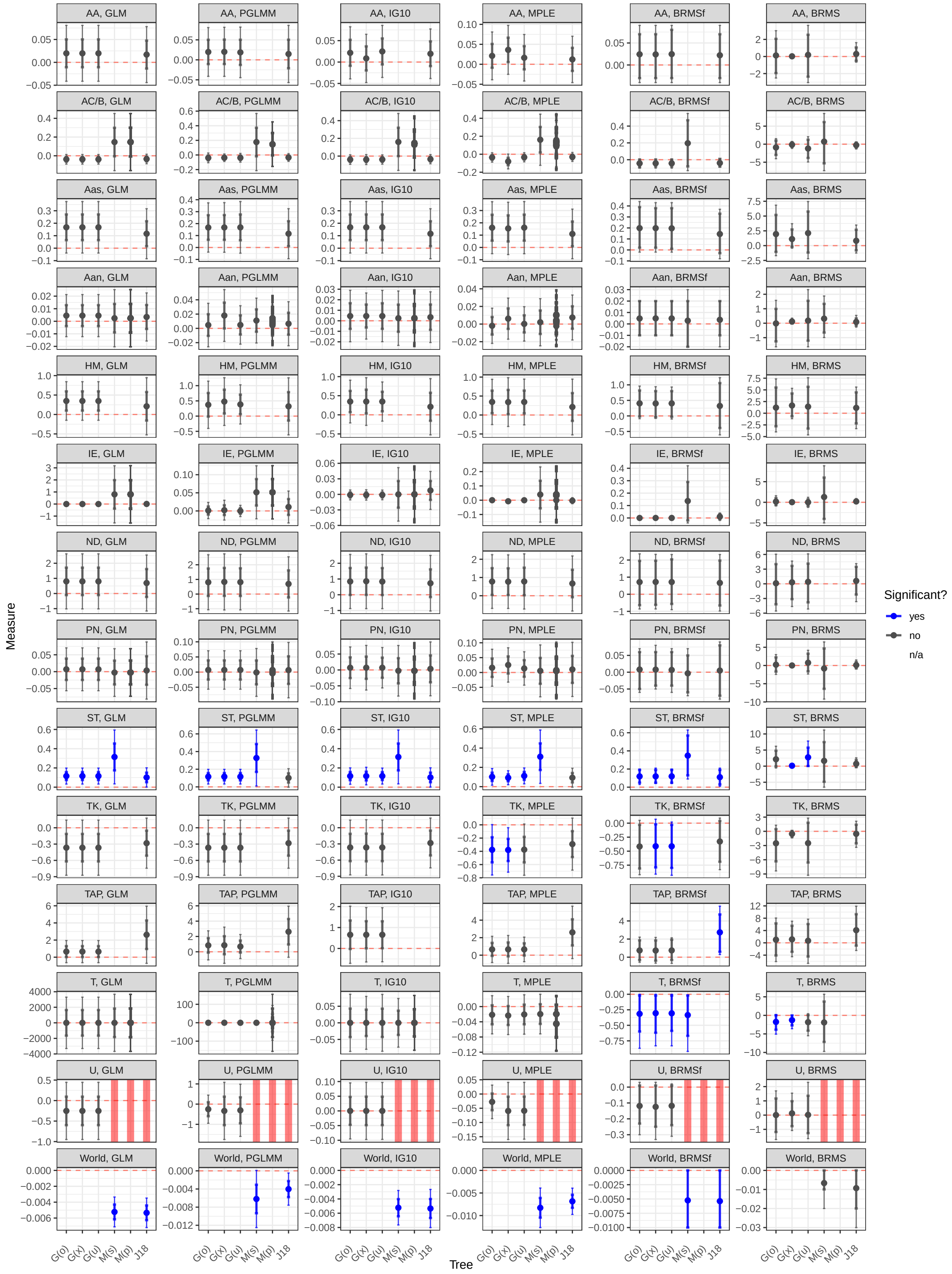

Figure S90. Phylogenetic (and non-phylogenetic "control") regressions of *blue* on *population size*. Please see Figure S84's caption for explanations.

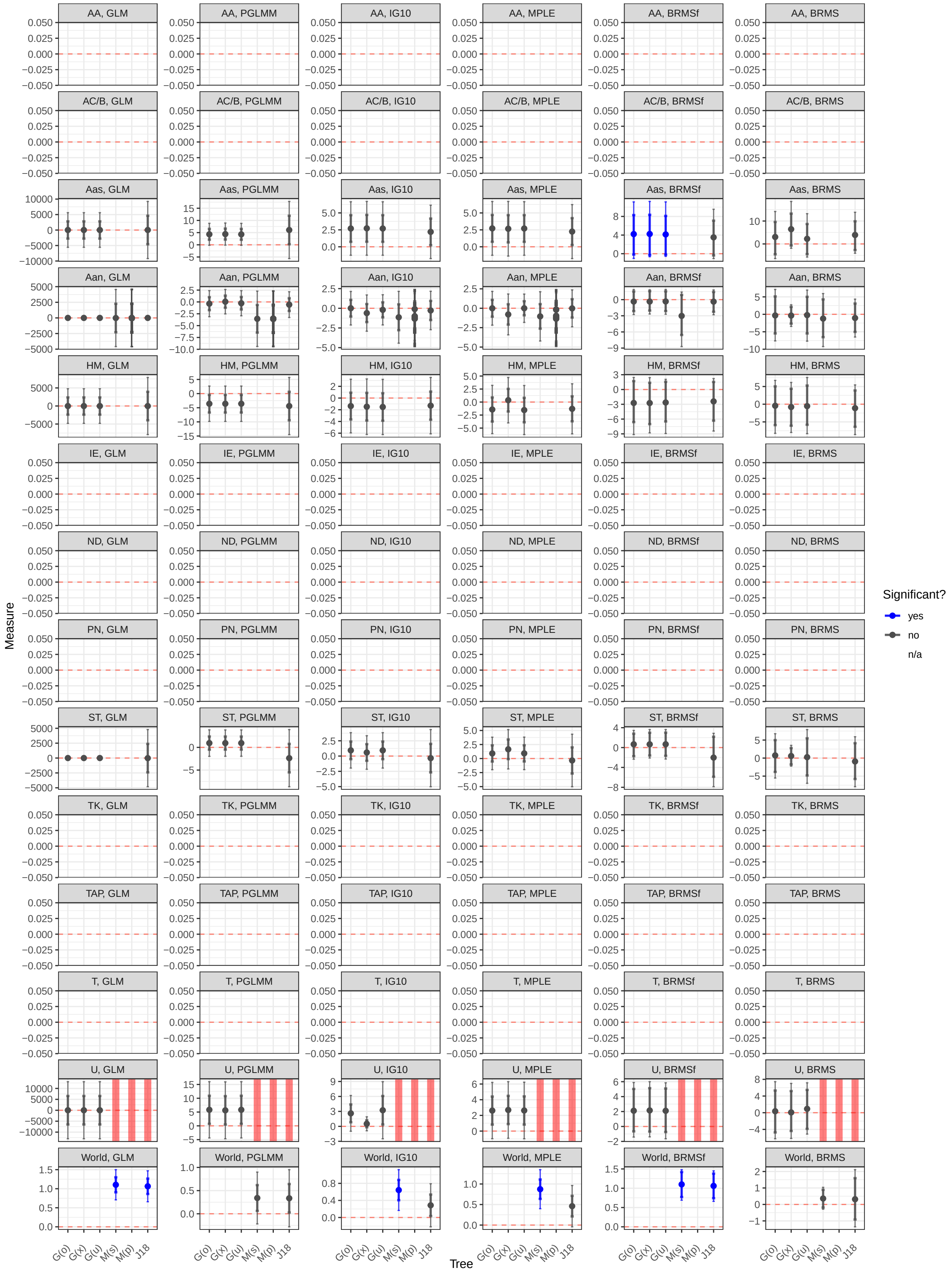

Figure S91. Phylogenetic (and non-phylogenetic "control") regressions of *blue* on *subsistence*. Please see Figure S84's caption for explanations.

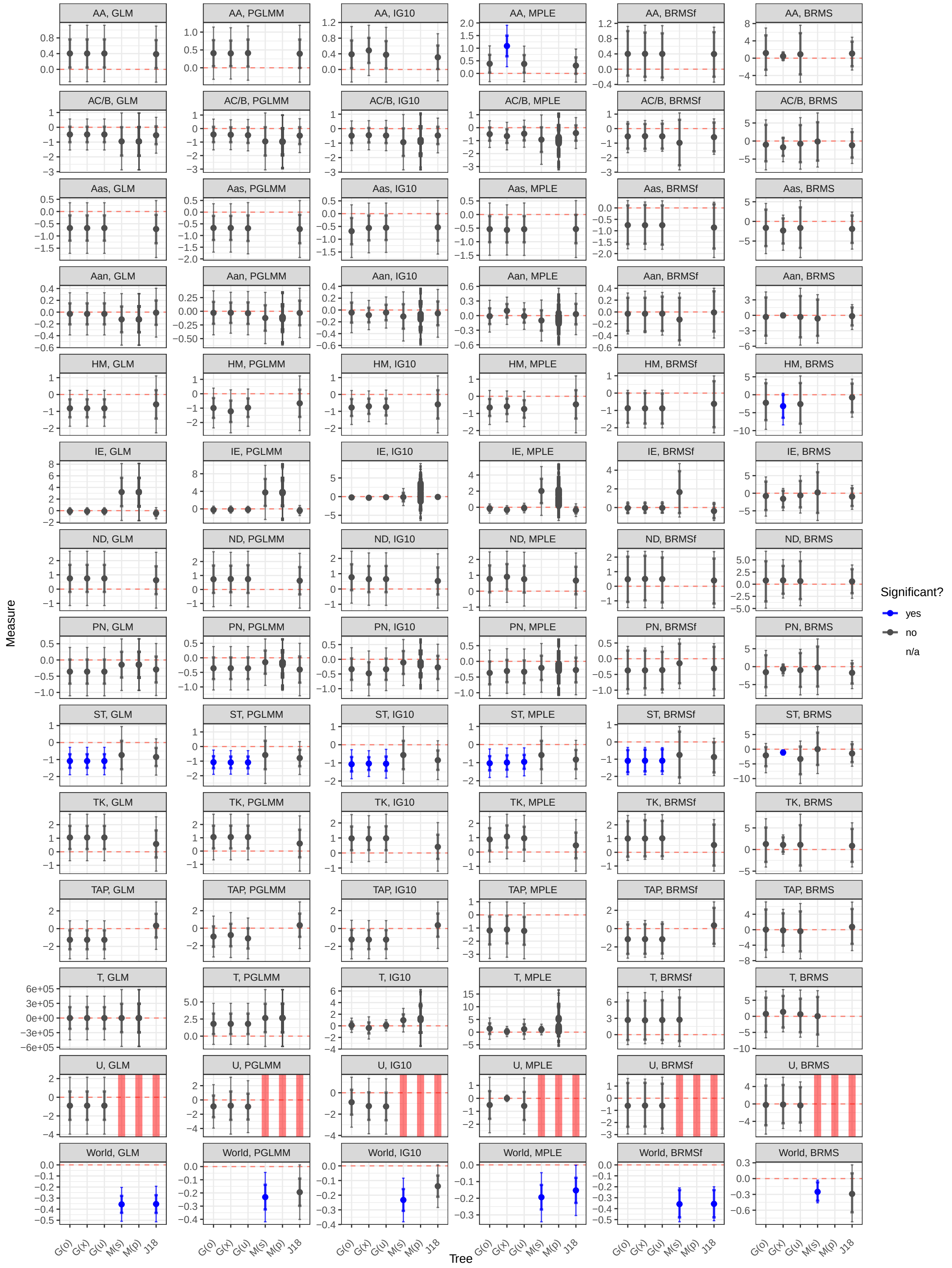

Figure S92. Phylogenetic (and non-phylogenetic "control") regressions of *blue* on *distance to lakes*. Please see Figure S84's caption for explanations.

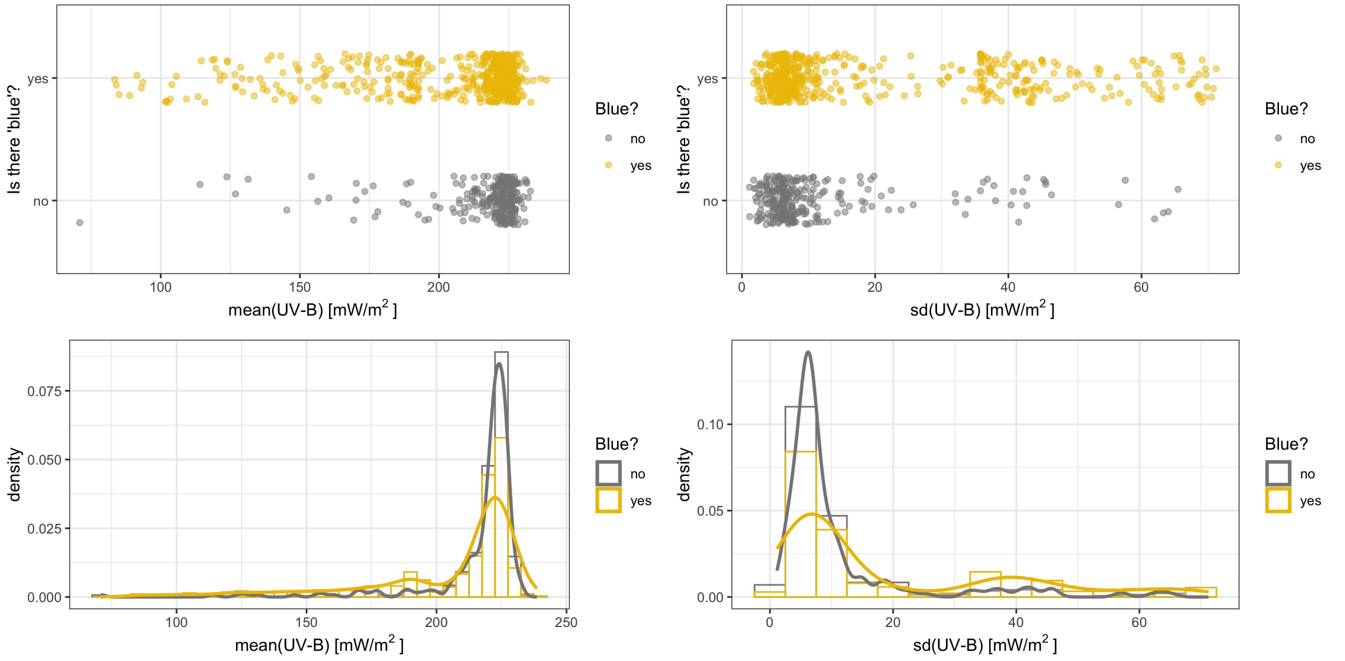

Figure S93. Relationship between the existence of a word for 'blue' and *UV-B mean* (left column) and *UV-B sd* (right column), showing the actual values (top row) and the histogram and density plot (bottom row). Figure generated using `R` version 4.2.3 (2023-03-15) and package `ggplot2` (version 3.4.0).

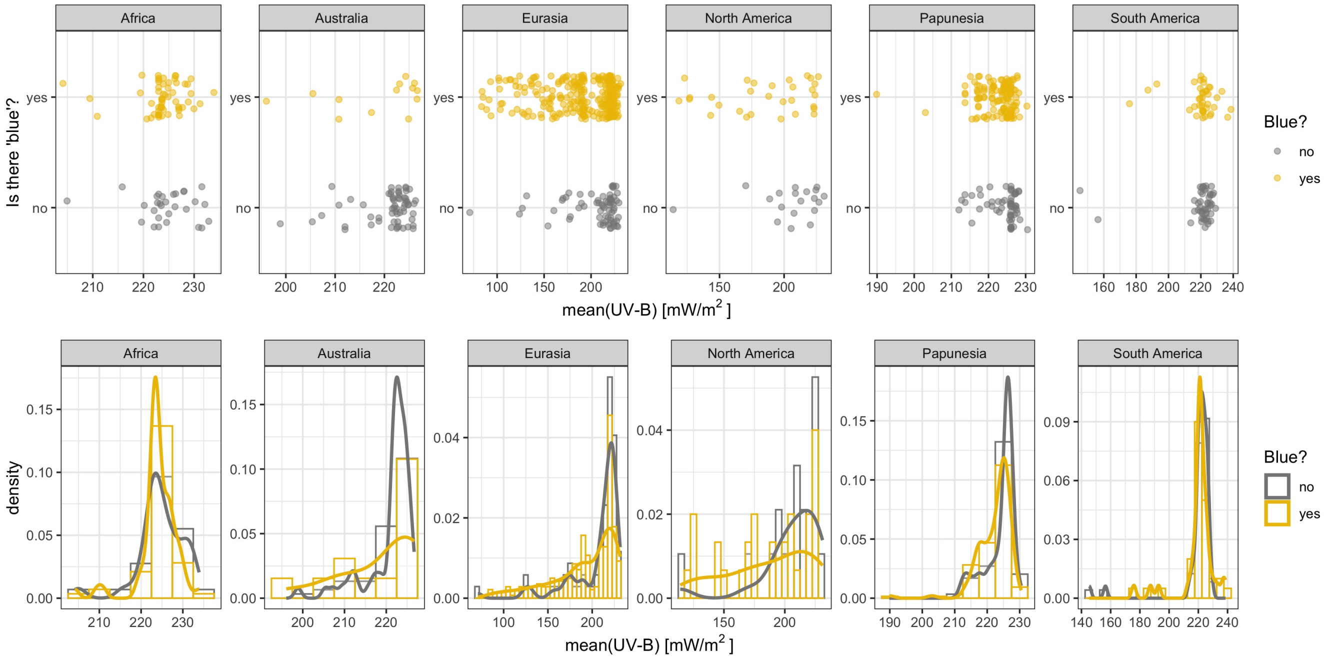

Figure S94. Relationship between the existence of a word for 'blue' and *UV-B mean* by macroarea. Same conventions as in Figure S93.

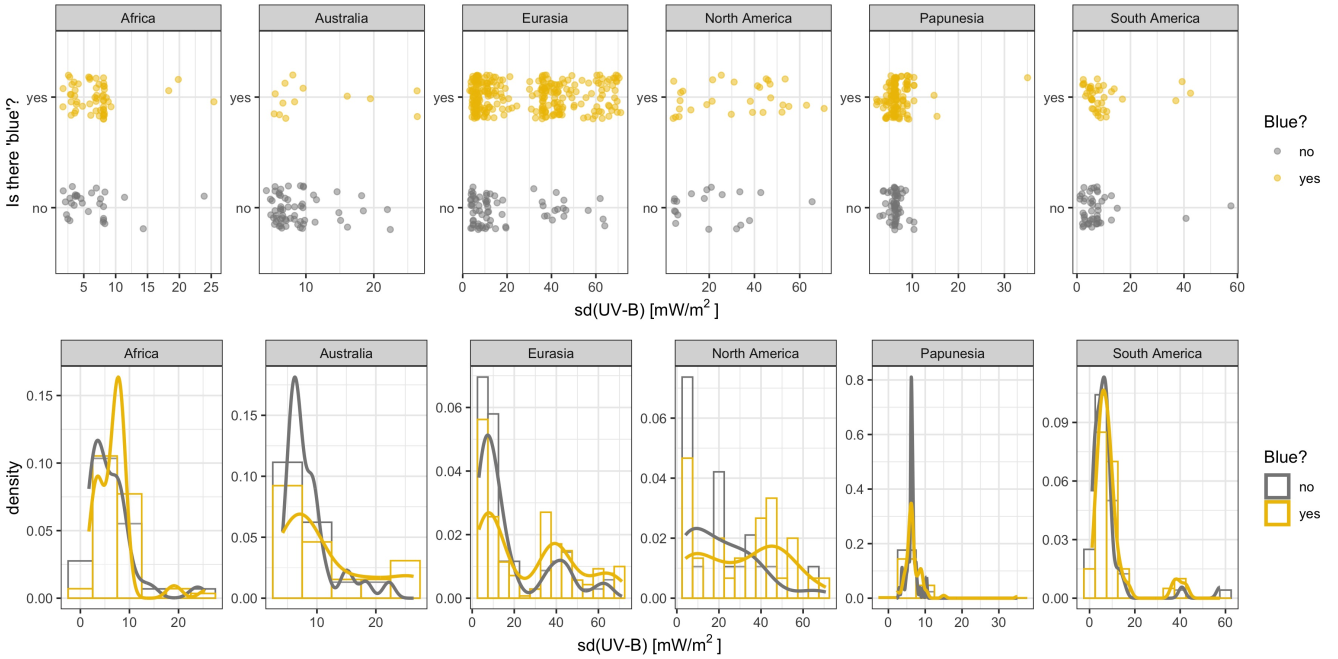

Figure S95. Relationship between the existence of a word for 'blue' and *UV-B sd* by macroarea. Same conventions as in Figure S93.

REFERENCES

Aklif, G. (1999). *Ardiyooloon Bardi Ngaanka : one arm point Bardi dictionary* (Kimberley Language Resource Centre, Halls Creek, W. A)

Alpher, B. (1991). *Yir-Yoront Lexicon: Sketch and Dictionary of an Australian Language*, vol. 6 of *Trends in Linguistics: Documentation* (Berlin, New York: Mouton de Gruyter)

Anderson, B. and Richards, E. (n.d.). *Yindjibarndi Dictionary* (Canberra: Australian Institute of Aboriginal and Torres Strait Islander Studies, Australian Indigenous Languages Collection (ASEDA 0297))

Ash, A., Giacon, J., and Lissarrague, A. (2003). *Gamilaraay, Yuwaalaraay & Yuwaalayaay Dictionary* (Alice Springs, NT, Australia: IAD Press)

Austin, P. (1992a). *A dictionary of Jiwarli, Western Australia* (La Trobe University, Dept. of Linguistics, Bundoora, Vic)

Austin, P. (1992b). *A dictionary of Thalanyji, Western Australia* (La Trobe University, Dept. of Linguistics, Bundoora, Vic)

Austin, P. (1992c). *A dictionary of Tharrgari, Western Australia* (La Trobe University, Dept. of Linguistics, Bundoora, Vic)

Austin, P. (1992d). *A dictionary of Warriyangka* (Canberra: Australian Institute of Aboriginal and Torres Strait Islander Studies, Australian Indigenous Languages Collection (ASEDA 0439))

Austin, P. (n.d.). *Payungu - English Dictionary* (Canberra: Australian Institute of Aboriginal and Torres Strait Islander Studies, Australian Indigenous Languages Collection (ASEDA 0394))

Birch, B. (2006). *A first dictionary of Erre, Mengerrdji and Urningangk : three languages from the Alligator Rivers region of north western Arnhem Land, Northern Territory, Australia* (Gundjeihmi Aboriginal Corporation, Jabiru, N.T)

Black, P. D. and Gilbert, R. (1988). *Kurtjar Dictionary* (Canberra: Australian Institute of Aboriginal and Torres Strait Islander Studies, Australian Indigenous Languages Collection (ASEDA 0026))

Blyth, N. (2001). *Wangka dictionary and grammar* (Canberra: Australian Institute of Aboriginal and Torres Strait Islander Studies, Australian Indigenous Languages Collection (ASEDA 0709))

Bouckaert, R., Bowern, C., and Atkinson, Q. (2018). The origin and expansion of Pama–Nyungan languages across Australia. *Nature Ecology & Evolution* doi:10.1038/s41559-018-0489-3

[Dataset] Bouckaert, R., Redding, D., Sheehan, O., Kyritsis, T., Gray, R., Jones, K. E., et al. (2022). Global language diversification is linked to socio-ecology and threat status. doi:10.31235/osf.io/f8tr6

Bradley, J. (n.d.). *Yanyuwa Dictionary* (Canberra: Australian Institute of Aboriginal and Torres Strait Islander Studies, Australian Indigenous Languages Collection (ASEDA 0382))

Brown, A. and Geytenbeek, B. (1991). *Ngarla-English dictionary* (Wangka Maya, Pilbara Aboriginal Language Centre, Port Hedland, W.A), 2nd interim ed. edn.

Burgman, A. (2007a). *Burduna : Burduna dictionary : English-Burduna wordlist and thematic wordlist 2007* (Wangka Maya Pilbara Aboriginal Language Centre South Hedland, WA)

Burgman, A. (2007b). *Nyamal dictionary : English-Nyamal finderlist and topical wordlist 2007* (Wangka Maya, Pilbara Aboriginal Language Centre, South Hedland, W.A)

Chang, W., Cathcart, C., Hall, D., and Garrett, A. (2015). Ancestry-constrained phylogenetic analysis supports the Indo-European steppe hypothesis. *Language* 91, 194–244. doi:10.1353/lan.2015.0005

Coate, H. H. J. and Elkin, A. P. (1974). *Ngarinjin-English dictionary*. No. 16 in Oceania linguistic monographs (Sydney: University of Sydney)

Eather, B., Kalamirnda, J., and Yurrbukka Community (2005). *A first dictionary of Na-Kara* (Maningrida Arts and Culture, Winnellie, N.T)

Evans, N., Merlan, F., and Tukumba, M. (2004). *A first dictionary of Dalabon (Ngalkbon)* (Maningrida Arts and Culture, Maningrida, N.T.)

Felsenstein, J. (2012). A comparative method for both discrete and continuous characters using the threshold model. *The American Naturalist* 179, 145–156. doi:10.1086/663681

Foote, T. (1992). *Kuuk Thaayorre dictionary : Thaayorre/English* (Jolien Press, Brisbane, Queensland)

Ford, L. J. (1997). *Batjamalh : dictionary and texts* (L.J. Ford, Bungendore, N.S.W.)

Fritz, S. A. and Purvis, A. (2010). Selectivity in mammalian extinction risk and threat types: a new measure of phylogenetic signal strength in binary traits. *Conservation Biology: The Journal of the Society for Conservation Biology* 24, 1042–1051. doi:10.1111/j.1523-1739.2010.01455.x

Geytenbeek, B. and Geytenbeek, H. (1991). *Nyangumarta-English dictionary (interim), with an English-Nyangumarta finder list* (Port Hedland, WA, Australia: Wangka Maya Pilbara Aboriginal Language Centre)

Glasgow, K. (1994). *Burarra – Gun-nartpa dictionary : with English finder list* (Summer Institute of Linguistics, Australian Aborigines and Islanders Branch Darwin)

Gray, R. D., Drummond, A. J., and Greenhill, S. J. (2009). Language phylogenies reveal expansion pulses and pauses in Pacific settlement. *Science* 323, 479–83. doi:10.1126/science.1166858

Green, J. (1992). *Alyawarr to English dictionary* (Institute for Aboriginal Development, Alice Springs, N.T)

Green, R. and Nimbadja, L. (2015). *Gurr-goni to English dictionary* (Batchelor Press, Batchelor, NT)

Grollemund, R., Branford, S., Bostoen, K., Meade, A., Venditti, C., and Pagel, M. (2015). Bantu expansion shows that habitat alters the route and pace of human dispersals. *Proceedings of the National Academy of Sciences* 112, 13296–13301. doi:10.1073/pnas.1503793112

Hale, K. and Leman, N. K. (1997). *Lardil dictionary: a vocabulary of the language of the Lardil people, Mornington Island, Gulf of Carpentaria, Queensland; with English-Lardil finder list* (Gununa, QLD, Australia: Mornington Shire Council)

Hammarström, H. and Forkel, R. (2021). Glottocodes: Identifiers linking families, languages and dialects to comprehensive reference information. *Semantic Web Journal*

Hansen, K. C. and Hansen, L. E. (1992). *Pintupi/Luritja dictionary* (Institute for Aboriginal Development Alice Springs, N.T), 3rd ed. edn.

Harvey, M. (2004). *Larrakia dictionary* (Darwin: ATSIC, Yirra Bandoo Aboriginal Corporation)

Heath, J. (1978). *Ngandi grammar, texts and dictionary* (Australian Institute of Aboriginal Studies ; Humanities Press Canberra, Atlantic Highlands, N.J.)

Heath, J. (1980). *Basic materials in Warndarang : grammar, texts and dictionary* (Dept. of Linguistics, Research School of Pacific Studies, Australian National University for Linguistic Circle of Canberra, Canberra)

Heath, J. (1981). *Basic materials in Mara : grammar, texts, and dictionary* (Dept. of Linguistics, Research School of Pacific Studies, Australian National University. Canberra)

Hercus, L. A. (1992a). *A Nukunu dictionary* (L.A. Hercus, Canberra)

Hercus, L. A. (1992b). *Wembawemba dictionary* (L.A. Hercus, Canberra)

Hercus, L. A. (1993). *Paakantyi dictionary* (L.A. Hercus, Canberra)

Hershberger, H. D. and Hershberger, R. (1982). *Kuku-Yalanji dictionary* (Summer Institute of Linguistics, Australian Aborigines Branch, Darwin)

Honkola, T., Vesakoski, O., Korhonen, K., Lehtinen, J., Syrjänen, K., and Wahlberg, N. (2013). Cultural and climatic changes shape the evolutionary history of the Uralic languages. *Journal of Evolutionary Biology* , 1–10doi:10.1111/jeb.12107

Hruschka, D. J., Branford, S., Smith, E. D., Wilkins, J., Meade, A., Pagel, M., et al. (2015). Detecting regular sound changes in linguistics as events of concerted evolution. *Current Biology* 25, 1–9. doi:10.1016/j.cub.2014.10.064

Hudson, J. and Richards, E. (1993). *Walmajarri Dictionary* (Canberra: Australian Institute of Aboriginal and Torres Strait Islander Studies, Australian Indigenous Languages Collection (ASEDA 0167))

Ives, A. R. and Garland, J., Theodore (2009). Phylogenetic Logistic Regression for Binary Dependent Variables. *Systematic Biology* 59, 9–26. doi:10.1093/sysbio/syp074

James, B. and Milingimbi School Literature Production Centre (2003). *Yan-nhangu dictionary 2003* (B. James, Milingimbi, N.T)

Jäger, G. (2018). Global-scale phylogenetic linguistic inference from lexical resources. *Scientific Data* 5, 180189. doi:10.1038/sdata.2018.189

Kilham, C., Adams, J., Bell, J., and Namponan, G. (1986). *Dictionary and sourcebook of the Wik-Mungkan language* (Summer Institute of Linguistics, Australian Aborigines Branch Darwin)

Kimberley Language Resource Centre (2010). *Bunuba draft dictionary* (Halls Creek, WA, Australia: Kimberley Language Resource Centre)

Laughren, M. (2016). Waanyi dictionary database

Lee, J. (2013). *Tiwi-English Interactive Dictionary*

Lissarrague, A., Ash, A., and Giacon, J. (2003). *Gamilaraay, Yuwaalaraay & Yuwaalayaay dictionary* (IAD Press, Alice Springs, N.T)

Lowe, B. and Lowe, B. (1976). *Temporary Gupapuyngu dictionary* (Milingimbi, NT, Australia)

Mackman, D. (2012). *Wajarri dictionary : the language of the Murchison Region of Western Australia : Wajarri to English, English to Wajarri* (Irra Wangga Language Centre, Geraldton, W.A)

Marmion, D. and Yamadji Language Centre (1995). *Badimaya dictionary* (Canberra: Australian Institute of Aboriginal and Torres Strait Islander Studies, Australian Indigenous Languages Collection (ASEDA 615))

McEntee, J. C. and McKenzie, P. (1992). *Adna-mat-na English dictionary* (J. McEntee, Adelaide), rev. may 1992. edn.

[Dataset] McKelson, K. R. (1989). Studies in Karajarri

Meakins, F., Campbell, L., Nordlinger, R., McConvell, P., Spence, J., Jones, C., et al. (2013). *Bilinarra to English dictionary* (Batchelor, NT, Australia: Batchelor Press)

Meakins, F., McConvell, P., Charola, E., McNair, N., McNair, H., Campbell, L., et al. (2013 2013). *Gurindji to English dictionary* (Batchelor Press, Batchelor, NT)

Merlan, F. and Jacq, P. (2005). *Jawoyn-English dictionary & English finder-list* (Diwurruwurru-jaru Aboriginal Corporation, Katherine, N.T)

Morelli, S. (2012). *Yaygirr dictionary and grammar* (Muurrbay Aboriginal Language and Culture Co-operative, Nambucca Heads, N.S.W)

Nordlinger, R. (1998). *An elementary Wambaya dictionary: with English - Wambaya finder list* (Papulu Aparr-kari Language Centre)

Oates, L. F. (1992). *Muruwari (Moo-roo-warri) dictionary* (produced with the assistance of the Australian Institute of Aboriginal and Torres Strait Islander Studies Albury, NSW)

Pagel, M. (1994). Detecting Correlated Evolution on Phylogenies: A General Method for the Comparative Analysis of Discrete Characters. *Proceedings of the Royal Society of London. Series B: Biological Sciences* 255, 37–45. doi:10.1098/rspb.1994.0006

Peile, A. R. and Valiquette, H. (1993). *A basic Kukatja to English dictionary* (Canberra: Australian Institute of Aboriginal and Torres Strait Islander Studies, Australian Indigenous Languages Collection (ASEDA 0504))

Pym, N. and Larrimore, B. (2011). Iwaidja-English Interactive Dictionary. In *AuSIL Interactive Dictionary Series A-2*, eds. C. E. Grimes and M. Lecompte

Roberson, D., Davidoff, J., Davies, I. R. L., and Shapiro, L. R. (2004). The development of color categories in two languages: A longitudinal study. *Journal of Experimental Psychology: General* 133, 554–571. doi:10.1037/0096-3445.133.4.554

Robertson, C. (1985). *Wangkumara grammar and dictionary* (Sydney: Department of Technical and Further Education, Aboriginal Education Unit)

Robertson, S. and Sommer, B. A. (1997). *Jaabugay dictionary* (Canberra: Australian Institute of Aboriginal and Torres Strait Islander Studies, Australian Indigenous Languages Collection (ASEDA 0013))

Round, E. R. (2021). *glottoTrees: Phylogenetic trees in Linguistics.* R package version 0.1

Saulwick, A., Coleman, C., McKay, G., and Bawinanga Aboriginal Corporation (2003). *A first dictionary of Rembarrnga* (Maningrida Arts & Culture)

Schwartz, S. (1996). Walpiri draft dictionary

Sharpe, M. C. (2001). *Alawa Nanggaya Nindanya Yalanu rugalarra = Alawa-Kriol-English dictionary* (Caitlin Press Prospect, S. Aust), longer ed. edn.

Singer, R., Garidjalalug, N., Hewett, H., Mirwuma, P., and Ambidjambidj, P. (2015). *Mawng dictionary v1.0*

Sutton, P. (1995). *Wik-Ngathan dictionary* (Caitlin Press Prospect, S .Aust)

Turpin, M. and Ross, A. (2012). *Kaytetye to English dictionary* (IAD Press, Alice Springs, N.T)

Wangka Maya Pilbara Aboriginal Language Centre (2008). *Ngajumaya dictionary 2008* (Wangka Maya Pilbara Aboriginal Language Centre, Port Hedland, W.A)

Waters, B. E. (1988). *Djinang dictionary* (Canberra: Australian Institute of Aboriginal and Torres Strait Islander Studies, Australian Indigenous Languages Collection (ASEDA 0009))

Wilson, S. and Harvey, M. (2001). *The Wagiman online dictionary*

Yang, Z., Kumar, S., and Nei, M. (1995). A New Method of Inference of Ancestral Nucleotide and Amino Acid Sequences. *Genetics* 141, 1641–1650

Zhang, M., Yan, S., Pan, W., and Jin, L. (2019). Phylogenetic evidence for Sino-Tibetan origin in northern China in the Late Neolithic. *Nature* 569, 112–115. doi:10.1038/s41586-019-1153-z

Zorc, R. D. and Bowern, C. (2004). *Yolngu Matha dictionary* (Canberra: Australian Institute of Aboriginal and Torres Strait Islander Studies, Australian Indigenous Languages Collection (ASEDA 778))

Zorc, R. D. P. (1996). *Yolngu-Matha dictionary* (Education Technology Unit, Batchelor College Batchelor, N.T)
